# Precise construction of spiro stereocenters *via* enantioselective radical addition through modulating photocatalysis from redox to energy transfer†

**DOI:** 10.1039/d5sc01583a

**Published:** 2025-05-09

**Authors:** Fayu Liu, Yanqi Guo, Weidong Lu, Xiaowei Zhao, Yanli Yin, Zhiyong Jiang

**Affiliations:** a Pingyuan Laboratory, School of Chemistry and Chemical Engineering, Henan Normal University Xinxiang Henan 453007 P. R. China yinzihust@163.com jiangzhiyong@htu.edu.cn; b College of Pharmacy, Henan University Kaifeng Henan 475004 P. R. China

## Abstract

Chiral hydrogen-bonding catalysis has been successfully applied in a wide range of asymmetric photocatalytic radical-based reactions. However, it faces intrinsic challenges in the reactions that rely on oxidative quenching to initiate transformations. A critical issue arises from the formation of anionic side intermediates, which preferentially interact with protons from chiral catalysts, undermining the essential enantiocontrol required for effective product formation. In this study, we demonstrate that creating energy transfer instead of single-electron transfer to trigger these transformations presents a promising solution. As a proof-of-concept, we report the first photocatalytic spirocyclization of olefinic sulfonyl oximes with vinyl azides, furnishing a diverse array of spiroaminals with high yields (up to 94%) and enantioselectivities (up to 99% ee). The success of this method hinges on employing a sulfonyl group as an *N*-protective group for oximes, which facilitates energy transfer as an alternative mechanism to initiate the transformation. This approach not only enhances reactivity and chemoselectivity but also creates an optimal environment for enantiocontrol. The synthetic significance of this work is underscored by the establishment of these products as a novel class of chiral ligands, with preliminary studies indicating their effectiveness in asymmetric alkynylation reactions.

## Abstract 2

Precise chiral hydrogen-bonding catalysis *via* modulating photocatalysis from redox to energy transfer.

## Graphical abstract

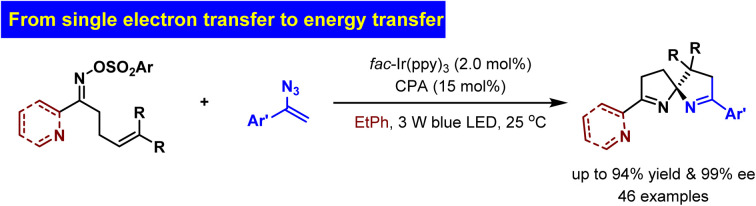

## Introduction

**Fig. 1:**
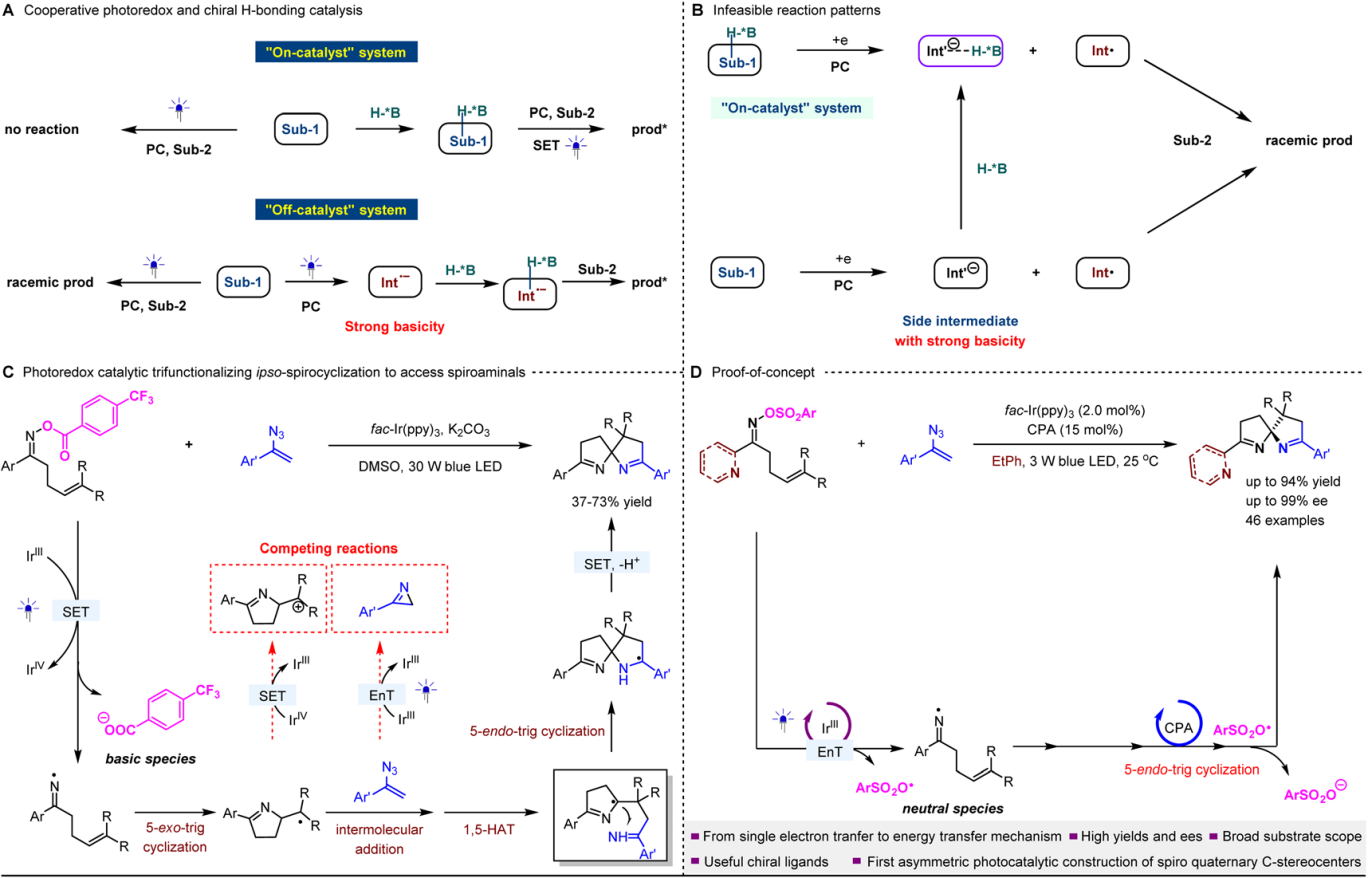
Outline of this work.

Chiral hydrogen-bonding (H-bonding) catalysis^1^ has emerged as a pivotal strategy in asymmetric synthesis, largely driven by the prevalence of electronegative atoms (*e.g.*, O and N) in the molecular frameworks of various feedstock chemicals. Despite the relatively modest energies associated with classical H-bonding interactions, this methodology has found extensive application in chiral radical chemistry through its integration with photoredox catalysis.^2–5^ To achieve precise enantiocontrol, most reactions typically employ an “on-catalyst” system,^6,7^ where chiral H-bonding catalysts, often chiral Brønsted acids, play a crucial role in facilitating transformations (Fig. 1A). These H-bonding interactions effectively lower the reduction potentials of substrates, enabling photocatalytic processes *via* redox mechanisms. In contrast, the “off-catalyst” system—where the chiral acid catalyst is responsible solely for providing stereocontrol during stereocenter formation—has yielded sporadic successes.^6,7^ To mitigate racemic background reactions, the efficacy of H-bonding interactions between key radical anion intermediates and chiral catalysts is paramount. It is anticipated that if the side intermediates formed after single-electron reduction exist as anions, the desired control over stereocenter formation may be compromised—regardless of whether an “on” or “off” system is employed—due to the significantly elevated basicity of these anions (Fig. 1B). This dilemma is particularly pronounced in those photoredox catalytic reactions that depend on oxidative quenching.^8,9^ Therefore, achieving high enantioselectivity poses a significant challenge in these reactions, and to date, there have been no reported successful instances utilizing chiral H-bonding catalysis.

As a striking example, current photoredox catalysis has identified iminyl radicals as pivotal intermediates for intramolecular cyclization, resulting in the formation of diverse and significant nitrogen-containing heterocycles.^10–15^ Typically, *O*-acyl and *O*-aryl oximes serve as precursors, undergoing single-electron reduction to initiate oxidative quenching catalysis and generate iminyl radical species. Notably, oxy anions often function as side intermediates, readily capturing protons within the reaction milieu. This phenomenon may account for the scarcity of reported examples utilizing chiral H-bonding catalysis to achieve enantioselective outcomes, notably involving the trifunctionalization of unactivated olefinic oxime esters *via ipso*-spirocyclization with vinyl azides (Fig. 1C).^12^ Besides the formation of carboxylic anion intermediates that complicate the attainment of precise enantioselectivity in this significant pathway for accessing valuable spiroamino derivatives, the intrinsic complexity of multi-step cascade radical transformations further challenges the efficacy of chiral catalysts in achieving sufficient enantiofacial differentiation. This is especially true given the simultaneous presence of multiple reaction intermediates. Moreover, the unavoidable occurrence of racemic background reactions reduces enantioselectivity within these photocatalytic systems.

To address this challenge, we propose that delaying the formation of unfavorable anion intermediates until after the construction of the stereocenter could represent a promising strategy. However, this approach raises mechanistic concerns, suggesting that the reductive quenching mechanism may be unfeasible, given that iminyl radicals possess the potential for further reduction. This limitation motivates us to explore the feasibility of modulating the photocatalytic process from a redox mechanism to energy transfer (EnT).^16,17^ This alternative approach generates two neutral radical species to initiate transformations (Fig. 1D). A subsequent single-electron transfer (SET) between one radical and the stereocenter-forming radical intermediate generated from the other radical would complete the reaction, yielding enantioenriched products.

Herein, we demonstrate the feasibility of the proposed scenario and successfully develop key asymmetric photocatalytic reactions involving vinyl azides and olefinic oxime esters. In these reactions, imine-containing azaarenes (*e.g.*, pyridines) function as the C-substituents of the oximes, as illustrated in Fig. 1D. Utilizing a dual catalyst system composed of a chiral phosphoric acid (CPA) and *fac*-Ir(ppy)_3_ as the photosensitizer under visible light, we successfully synthesized a diverse array of valuable spiroaminals with high yields and enantiomeric excesses (ees). This study marks the first validation of asymmetric photocatalysis in constructing spiro-quaternary carbon stereocenters *via* an enantioselective radical addition process. The enantioenriched products derived from this methodology hold promise as a novel class of *N*,*N*,*N*-chiral ligands. The success of these reactions is underpinned by the substitution of the traditionally utilized ester group with a sulfonyl group, which protects the nitrogen atom of the oximes. This modification enables the desired EnT mechanism, contrasting with the previously employed SET process. In addition to creating a conducive environment to effectively mitigate the adverse influence of undesired anionic side intermediates on chiral H-bonding catalysis, this strategy considerably enhances reaction efficiency.

## Results and discussion

Of note, the synthesis of spirochiral molecules has garnered considerable attention over recent decades, driven by their prevalence in natural products, bioactive compounds, and chiral ligands.^18–22^ The synthetic advantages offered by photocatalysis involving high reactivity and mild reaction conditions have led to significant advancements in this area.^12,23,24^ As mentioned above, a significant advancement involves the trifunctionalization of unactivated olefinic oxime esters *via ipso*-spirocyclization with vinyl azides.^12^ Notably, in addition to the elusive enantiocontrol, the intramolecular photocycloaddition of vinyl azides *via* EnT can occur at a rapid pace (Fig. 1D).^25^ Meanwhile, the radical intermediates (*vide infra*) generated *via* the 5-*exo*-trig cyclization of iminyl radicals exhibit heightened susceptibility to single-electron oxidation. This poses significant challenges concerning the intrinsic chemoselectivity of this pivotal transformation, which is contingent upon rigorous reaction conditions, thus further complicating the attainment of precise enantioselectivity.

Nonetheless, we undertook the challenge of addressing this reaction. Our preliminary investigation began with the reaction of 2-pyridyl-containing oxime ester 1 and 1-(1-azidovinyl)-4-methylbenzene 5 (Table 1A). Initial attempts under the conditions of 2.0 mol% *fac*-Ir(ppy)_3_, 15 mol% SPINOL-based CPA C1, and 1.5 equivalents of K_2_CO_3_ at 25 °C in *tert*-butylbenzene as the solvent could not yield the desired product 6. Notably, compound 5 was fully consumed, resulting in the formation of the corresponding 3-(*p*-tolyl)-2*H*-azirine, verifying the existence of this competing reaction. This lack of success prompted us to investigate the modulation of *N*-leaving groups in the oxime to improve reactivity. Encouragingly, the use of 4-(trifluoromethyl)benzenesulfonyl oxime 2 led to the formation of 6 with an ee of 31%. This result motivated exploration of different aryl substituents on the sulfonyl groups, such as compounds 3 and 4. Notably, the reaction of 4 with 5 produced product 6 with an improved ee of 61%. This progress underscores the potential of optimizing *N*-leaving groups to enhance enantioselectivity in our photocatalytic system.

**Table 1:** Optimization of the reaction conditions^*a*^

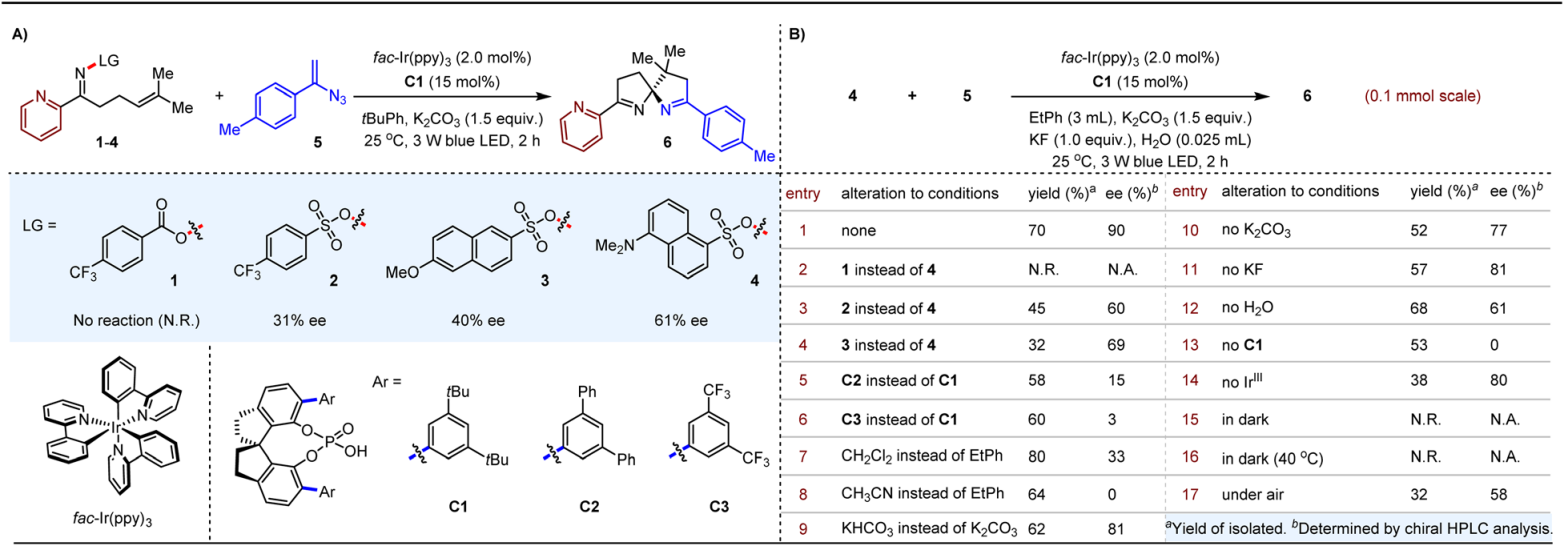

Consequently, we chose the transformation between substrates 4 and 5 to study various reaction parameters with the aim of enhancing enantioselectivity (refer to Table S1 in the ESI†). Remarkably, the addition of 1.0 equivalent of KF and 0.025 mL of H_2_O as additives in ethylbenzene generated product 6 with a yield of 70% and an ee of 90% (entry 1, Table 1B). However, substituting substrate 4 with 1 led to the absence of product 6 under identical conditions (entry 2). Reactions employing substrates 5 with oximes 2 or 3 yielded 6 with moderate enantioselectivities and poor overall yields (entries 3 and 4). We then investigated CPAs C2 and C3, which featured phenyl and trifluoromethyl substituents on the aromatic ring in place of the *tert*-butyl groups of CPA C1 (entries 5 and 6). These modifications, however, resulted in diminished enantioselectivity for product 6, underscoring the sensitivity of enantiocontrol to substituents at the 6,6′-positions of SPINOL. The switch to dichloromethane as the solvent enhanced both reactivity and chemoselectivity, resulting in the formation of 6 with an 80% yield, but the ee decreased to 33% (entry 7). Evaluating more polar solvents, such as acetonitrile, revealed no observable enantioselectivity for product 6 (entry 8). Next, we examined the impact of K_2_CO_3_ by replacing it with KHCO_3_ (entry 9), which led to the formation of product 6 in 62% yield with an ee of 81%. The omission of K_2_CO_3_ resulted in further declines in both yield and ee, highlighting the critical role of this inorganic salt in the catalytic system (entry 10). Moreover, the presence of KF and H_2_O as additives proved essential for maintaining enantioselectivity (entries 11 and 12). Despite the inability to completely exclude H_2_O from the reaction system, the addition of supplemental H_2_O may enhance the solubility of inorganic bases, which are crucial for achieving high enantioselectivity. Notably, when the reaction was conducted without CPA C1, a racemic product 6 was obtained in 53% yield (entry 13), suggesting the existence of a racemic background transformation within the reaction system. Interestingly, product 6 was still produced in 38% yield with an ee of 80% even in the absence of the photosensitizer (entry 14). Further investigations conducted in the dark at both 25 °C and 40 °C revealed no reaction (entries 15 and 16). Ultimately, under an ambient atmosphere, product 6 was obtained in 32% yield with an ee of 58% (entry 17).

With the optimized conditions in place, we proceeded to evaluate the substrate scope of this asymmetric photocatalytic spirocyclization reaction. A diverse array of 1-aryl vinyl azides was subjected to the established reaction conditions alongside substrate 4 (Table 2). This led to the successful synthesis of a series of pyridine-functionalized spiroaminals (6–29), which were obtained in yields ranging from 43% to 94% and ees between 83% and 99%. Notably, the introduction of electron-withdrawing or -donating groups at the *para*-, *meta*-, or *ortho*-positions of the aromatic rings of the vinyl azides (7–13 and 14–25) did not significantly impact enantioselectivity. The relatively lower yields observed for certain products (*e.g.*, 16, 24) can be attributed to the diminished reactivity of radical addition to vinyl azides, as several vinyl azides were consumed to form the corresponding azirines. Importantly, the presence of the trimethylsilyl (TMS) group in compound 16 demonstrated good compatibility with the KF present in the reaction system. Furthermore, the robust tolerance of the allyl group (23) underscores the precise chemoselectivity of this reaction among different olefins. We also explored a selection of vinyl azides featuring fused aromatic (26, 27) and heteroaromatic (28, 29) rings, achieving high yields and ees, thereby underscoring the versatility of this methodology.

**Table 2:** Reactions of 4 with diverse vinyl azides

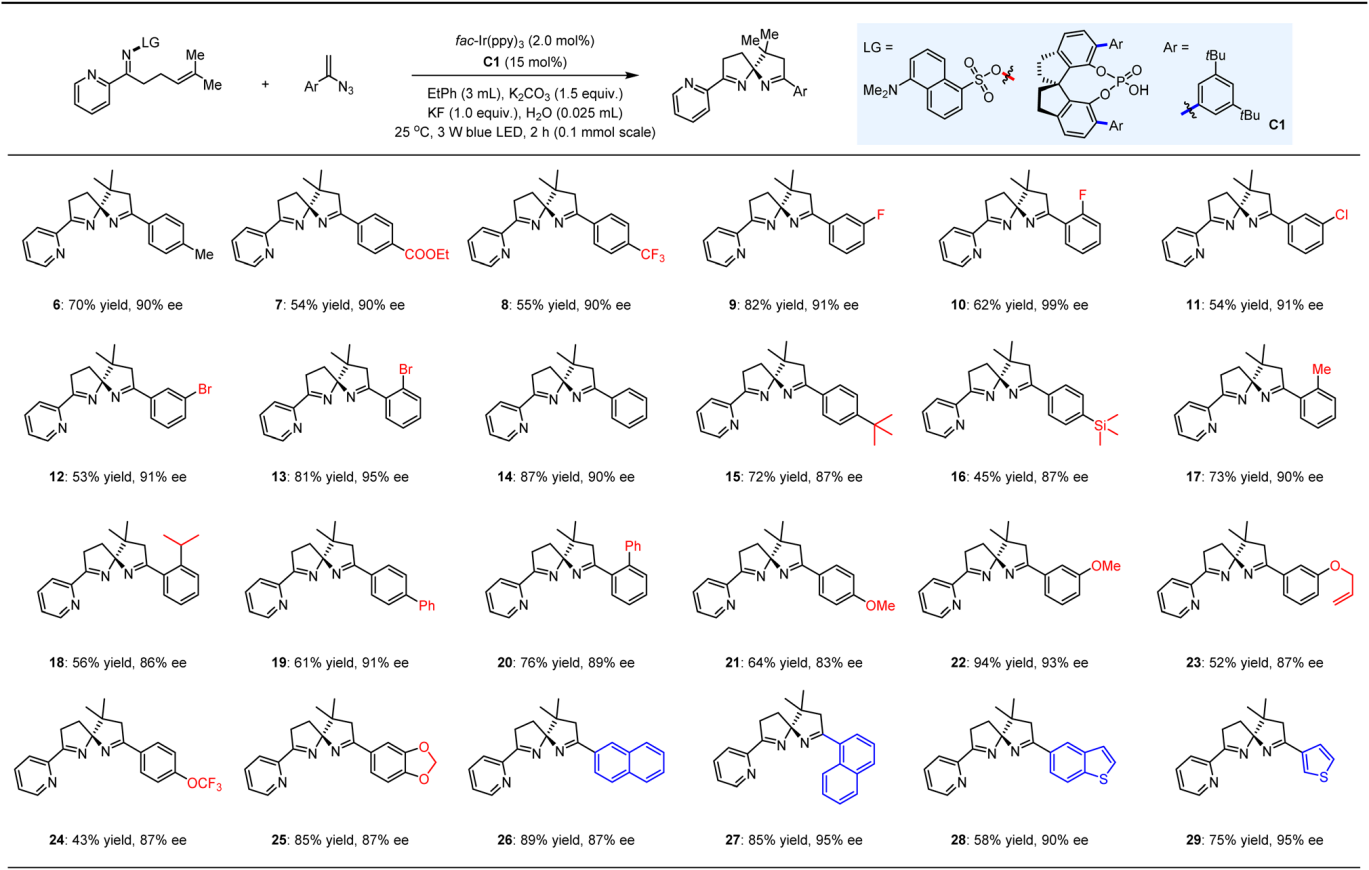

We subsequently investigated the reactions of 1-(1-azidovinyl)-2-bromobenzene with a range of azaarene-substituted 5-(dimethylamino)naphthalene-1-sulfonyl oximes (Table 3). The choice of this vinyl azide was driven by the challenge posed by *ortho*-substitution, which complicates the achievement of satisfactory yields and ees and serves as a useful measure of the synthetic versatility of this strategy. Additionally, the bromide functionality is amenable to various modifications, thus enriching the diversity of these important chiral ligands. Notably, the introduction of substituents with varying electronic properties at the 3-, 4-, 5-, and 6-positions of the 2-pyridyl rings of the oxime substrates did not adversely affect the excellent enantioselectivity, with the corresponding products (30–36) obtained in yields ranging from 56% to 75% and ees exceeding 90%. Importantly, the pyridine moiety in the products was amenable to modulation by quinolines (*e.g.*, 37) and isoquinoline (*e.g.*, 38), thereby further broadening the spectrum of this novel class of potential ligands. Moreover, the substituents on the cyclic rings of the chiral ligands can effectively tune spatial configurations, which is crucial for the broader application of chiral ligands. In this context, the two methyl groups of compound 4 were replaced with various linear alkyl chains (*e.g.*, 39, 40), benzyl groups (*e.g.*, 41), and cyclic structures (*e.g.*, 42–51), yielding a series of structurally appealing spiroaminals. Notably, a diverse range of saturated carbon rings—four-membered (42), five-membered (43, 44), six-membered (45–49), seven-membered (50), and twelve-membered (51)—could be readily constructed on these entities with satisfactory yields and ees.

**Table 3:** Reactions of 1-(1-azidovinyl)-2-bromobenzenes with distinct azaarene-substituted 5-(dimethylamino)naphthalene-1-sulfonyl oximes

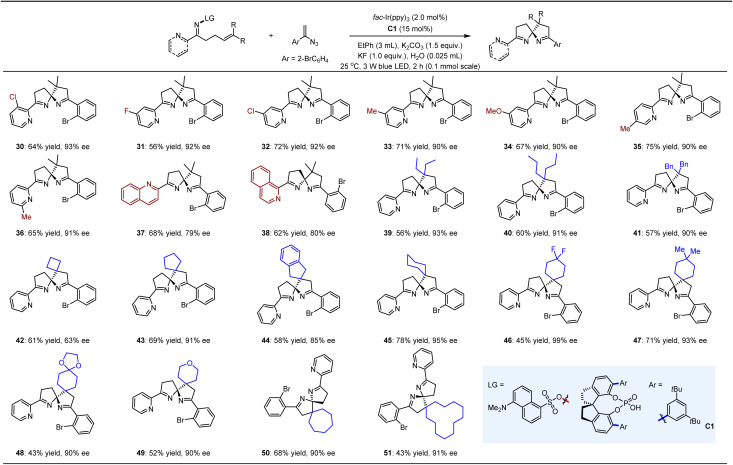

**Fig. 2:**
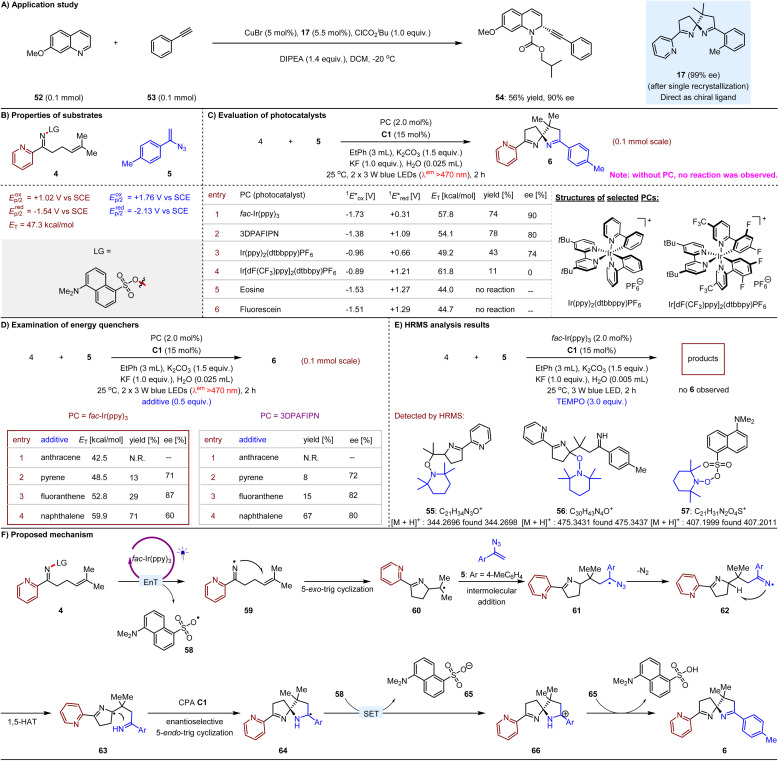
Synthetic utility, mechanistic studies, and the plausible mechanism.

We then assessed the potential of these azaarene-based spiroaminal products as chiral ligands in metal catalysis to demonstrate the synthetic utility of our findings. As illustrated in Fig. 2A, we investigated the asymmetric alkynylation of quinoline 52 with ethynylbenzene (53) in the presence of 5 mol% CuBr,^26^ 1.0 equivalent of isobutyl carbonochloridate, and 1.4 equivalents of *N*,*N*-diisopropylethylamine (DIPEA) in dichloromethane as the solvent at −20 °C, utilizing ligand 17, which exhibited 99% ee following single recrystallization. We were pleased to observe that the desired product 54 was obtained in 56% yield with 90% ee, thereby effectively circumventing the need for the axially chiral P,N ligand, (*S*)-StackPhos,^26^ which necessitates laborious preparation methods.

To elucidate the potential reasons for the reaction's occurrence when the ester groups were replaced with sulfonyl groups, we conducted a series of experiments, using the reaction between substrates 4 and 5 as a representative case. It is worth mentioning that the reaction of 1 and 5 could result in the formation of the desired product by employing a more polar solvent (*i.e.*, dichloromethane) instead of ethylbenzene (see Table S1 in the ESI†). However, the yield was unsatisfactory due to considerably poor chemoselectivity, and we observed poor enantioselectivity despite testing various chiral Brønsted acids.

We initially assessed the UV/Vis spectra of compounds 4, 5, and C1, which excluded the possibility of the formation of new photosensitive species, such as electron donor–acceptor complexes, within the reaction system. The findings also confirmed that compound 4 can be activated by the utilized light source, thereby validating the feasibility of the outcome presented in Table 1B (entry 14). Subsequently, we determined the redox potentials of compounds 4 and 5 (Fig. 2B). The data demonstrated that the photo-excited *fac*-Ir(ppy)_3_ (*E*_ox_ = −1.73 V, *E*_red_ = +0.31 V, *E*_T_ = 57.8 kcal mol^−1^)^27,28^ is capable of facilitating either SET *via* oxidative quenching or EnT with compound 4 (*E*_red_ = −1.54 V, *E*_T_ = 47.3 kcal mol^−1^). However, performing a Stern–Volmer experiment to elucidate the mechanism proved impractical, as *fac*-Ir(ppy)_3_ exhibits a maximum fluorescence emission at 510 nm, whereas substrate 4 emits at 495 nm. Considering that the concentration of substrate 4 was substantially higher than that of *fac*-Ir(ppy)_3_ during the measurements, any potential quenching effects remained undetectable.

In this context, we tested the reaction under light irradiation with emission wavelengths above 470 nm, noting that no reaction occurred in the absence of the photosensitizer (Fig. 2C). We found that product 6 could be obtained in 74% yield with 90% ee in the presence of *fac*-Ir(ppy)_3_ (entry 1). A series of photosensitizers were subsequently examined (entries 2−6), and 3DPAFIPN,^29,30^ Ir(ppy)_2_(dtbbppy)PF_6_,^31^ and Ir[dF(CF_3_)ppy]_2_(dtbbpy)PF_6_ (ref. 31) led to the formation of product 6 (entries 2–4). Importantly, the excited states of these three photosensitizers lacked sufficient reductive ability to facilitate SET with compound 4. While eosine^32^ and fluorescein^32^ are effective photoredox catalysts, they were not suitable for EnT with 4, resulting in no reaction. These findings suggest that the transformation might be initiated by EnT.

We further investigated the use of various polycyclic aromatic hydrocarbons (PAHs) as energy quenchers (Fig. 2D).^33^ Notably, when employing *fac*-Ir(ppy)_3_ as the photosensitizer, the addition of 0.5 equivalents of anthracene completely inhibited the transformation (left table, entry 1). Pyrene and fluoranthene, which possess slightly higher energy than substrate 4 but lower than *fac*-Ir(ppy)_3_ resulted in sluggish reactions, yielding product 6 in 13% and 29% respectively (entries 2 and 3). In contrast, the transformation yielded product 6 in 71% when naphthalene was used as an additive (entry 4). Given that photoactivated *fac*-Ir(ppy)_3_ may undergo SET with 4, we also tested 3DPAFIPN as an alternative photosensitizer, along with the corresponding PAHs as additives (right table). This approach yielded similar results. Notably, due to the slightly lower energy of 3DPAFIPN compared to *fac*-Ir(ppy)_3_ the inhibitory effects of pyrene and fluoranthene were more pronounced (entries 2 and 3). Furthermore, when 2.0 equivalents of these PAHs were introduced into the reaction system, all species, with the exception of naphthalene, were observed to completely inhibit the transformation, regardless of whether *fac*-Ir(ppy)_3_ or 3DPAFIPN was employed as the photosensitizer.

To elucidate the possible intermediates in the reaction system, we investigated the addition of 3.0 equivalents of TEMPO during the reaction of compounds 4 and 5 (Fig. 2E). Notably, the absence of product 6 indicates that radical processes are involved in its formation. High-resolution mass spectrometry (HRMS) analysis revealed the presence of three distinct species (55, 56, and 57), providing crucial insights into the underlying reaction mechanism. Specifically, product 55 confirms the intramolecular addition of an iminyl radical to an olefin, while product 56 supports the occurrence of an intramolecular 1,5-hydrogen atom transfer (HAT) event. Importantly, the detection of species 57 lends further credence to an energy transfer (EnT)-based photocatalytic mechanism.

Based on the experimental results and previous studies,^13^ a plausible mechanism for the complex photocatalytic spirocyclization reaction has been proposed, with the transformation between compounds 4 and 5 selected as a representative example. As shown in Fig. 2F, the reaction is initiated by an EnT between compound 4 and the triplet state *fac*-Ir(ppy)_3_, leading to the formation of radicals 58 and 59. Notably, radical 59 can undergo a 5-*exo*-trig cyclization to yield intermediate 60, which subsequently participates in a radical addition to the olefin moiety of compound 5, forming radical 61. Upon the release of N_2_, the resulting nitrogen atom radical 62 undergoes HAT to generate radical 63. The subsequent 5-*endo*-trig cyclization is crucial, as it results in the construction of a spiro quaternary carbon stereocenter. The basicity of azaarenes and their demonstrated ability to accept protons from CPAs likely enable two H-bonding interactions: one between the CPA C1 and the N–H bond, and another between the CPA C1 and the nitrogen atom of the pyridine moiety in radical 63.^34–43^ These interactions are presumably responsible for allowing the chiral catalyst to exert enantiocontrol during the radical addition process. Consequently, we propose that the EnT process is vital for achieving precise enantioselectivity in this chiral H-bonding catalysis. Since the anionic intermediate (Fig. 1C) will be generated prior to this process in the SET platform, it may possess sufficient basicity to preferentially interact with the chiral Brønsted acid catalyst, thereby diminishing, and potentially disrupting, the essential H-bonding interactions. Following the SET process with radical 58, the resultant radical 64 can undergo oxidation to form cation 66. Concurrently, the anion 65 generated from radical 58 swiftly captures a proton from cation 65, resulting in the formation of the final enantioenriched product 6, alongside an acidic by-product that will be neutralized by the weak inorganic bases present in the reaction. Consequently, the enantioselective control exerted by the chiral acid catalyst remains unaffected by the presence of the formed anions.

## Conclusions

In summary, we have developed a generic and efficient strategy for chiral H-bonding catalysis that achieves precise enantiocontrol in oxidative quenching-engaged photoredox catalytic reactions, which are increasingly prevalent in contemporary organic synthesis. The key innovation of this protocol lies in the modulation of the redox mechanism to an EnT process. This adjustment effectively shifts the generation of formidable anion intermediates in chiral H-bonding catalysis to occur after the formation of stereocenters. This results in enhanced preferential interactions between the acid catalysts and the key radical species. As a prime example, we have successfully executed the first photochemical asymmetric spirocyclization reaction to construct challenging spiro quaternary carbon stereocenters through enantioselective radical addition. By employing a dual catalyst system that integrates a photosensitizer with a chiral Brønsted acid, a diverse range of olefinic sulfonyl oximes can efficiently react with vinyl azides to yield valuable azaarene-functionalized spiroaminals in high yields and excellent enantioselectivities. The incorporation of a sulfonyl protective group for oximes enables these transformations to be initiated *via* EnT, in contrast to the previously utilized SET approach. This innovative reaction mechanism allows for effective transformations under mild conditions while achieving remarkable enantioselectivity. Importantly, the successful application of these products as chiral ligands in asymmetric alkynylation reactions highlights that azaarene-functionalized spiroaminals represent a significant class of chiral ligands. This advancement enriches the toolkit of asymmetric metal catalysis and underscores the considerable practical value of our approach.

## Author contributions

Z. J. conceived and designed the experiments. F. L., Y. G., W. L., and Q. L. performed the experiments. F. L., Y. Y., and X. Z. analysed and interpreted the results. F. L., Y. Y., and Z. J. prepared the ESI.† Z. J. wrote the paper. All authors discussed the results and commented on the manuscript.

## Conflicts of interest

There are no conflicts to declare.

## Supplementary Material

SC-016-D5SC01583A-s001

SC-016-D5SC01583A-s002

## Data Availability

The data that support the findings of this study are available from the corresponding author upon reasonable request.
