# Supplementary material for: Precise construction of spiro stereocenters via enantioselective radical addition through modulating photocatalysis from redox to energy transfer
Source: Chem Sci. 2025 May 9;16(23):10555–62. doi: 10.1039/d5sc01583a (PMC12076215; doi:10.1039/d5sc01583a)
Supplement: SC-016-D5SC01583A-s001 [file SC-016-D5SC01583A-s001.pdf]

Supplementary Information (SI) for Chemical Science.
This journal is © The Royal Society of Chemistry 2025

Supporting Information

Precise construction of spiro stereocenters via enantioselective radical addition through modulating photocatalysis from redox to energy transfer

Fayu Liu,[a] Yanqi Guo,[a] Weidong Lu,[a]
Xiaowei Zhao,[b] Yanli Yin,*[a] and Zhiyong Jiang*[a,b]

[a]*Pingyuan Laboratory, School of Chemistry and Chemical Engineering, Henan Normal University, Xinxiang, Henan, P. R. China 453007*

[b]*College of Pharmacy, Henan University, Kaifeng, Henan, P. R. China 475004*

E-mail: yinzihust@163.com (Y.Y.); jiangzhiyong@htu.edu.cn (Z.J.)

Table of Contents

1. General information

S3-S4

2. Optimization of reaction conditions

S5-S7

3. Experimental procedures

S8-S25

4. Experimental equipment

S26

5. Mechanism studies

S27-S37

6. Determination of the absolute configurations

S38-S42

7. Characterization of adducts

S43-S90

8. Copies of NMR spectra

S91-S166

9. References

S167

1. General information

Experiments involving moisture and/or air sensitive components were performed under a positive pressure of argon in oven-dried glassware equipped with a rubber septum inlet. Dried solvents and liquid reagents were transferred by oven-dried syringes or hypodermic syringe cooled to ambient temperature in a desiccator. Reaction mixtures were stirred in 10 mL Schlenk tube with Teflon-coated magnetic stirring bars unless otherwise stated. Moisture in non-volatile reagents/compounds was removed in high vacuo by means of an oil pump and subsequent purging with nitrogen. Solvents were removed under vacuum and heated with a water bath at 35−40 °C using rotary evaporator with aspirator. The condenser was cooled with running water at 0 °C.

All experiments were monitored by analytical thin layer chromatography (TLC). TLC was performed with pre-coated TLC plates, *silica gel* 60F-254, layer thickness 0.25 mm. After elution, plate was visualized under UV illumination at 254 nm for UV active material. Further visualization was achieved by staining phosphomolybdic acid solution. For those using the aqueous stains, the TLC plates were heated on a hot plate.

Columns for flash chromatography (FC) contained silica gel 200−300 mesh. Columns were packed as slurry of silica gel in petroleum ether and equilibrated solution using the appropriate solvent system. The elution was assisted by applying pressure of about 2 atm with an air pump.

Instrumentations

NMR: ^{1}H NMR spectra were recorded at room temperature on a Bruker AVANCE NEO 400 (400 MHz) or Bruker AVANCE III HD 600 (600 MHz) spectrometer. ^{13}C NMR spectra were recorded on a Bruker AVANCE NEO 400 (100 MHz) or Bruker AVANCE III HD 600 (151 MHz) with complete proton decoupling. ^{19}F NMR spectra were recorded on a Bruker AVANCE NEO 400 (376 MHz) or Bruker AVANCE III HD 600 (565 MHz) spectrometer. Proton nuclear magnetic resonance (^{1}H NMR) and carbon NMR (^{13}C NMR) were recorded in $CDCl_3$ or CD_3OD. Chemical shifts are reported in parts per million (ppm), using the residual solvent signal as an internal standard: $CDCl_3$ (^{1}H NMR: δ 7.26, singlet; ^{13}C NMR: δ 77.16, triplet), CD_3OD (^{1}H NMR: δ 3.31, multiplets; ^{13}C NMR: δ 48.80, multiplets). Multiplicities were given as: *s* (singlet), *d* (doublet), *t* (triplet), *q* (quartet), *quintet*, *m* (multiplets), *dd* (doublet of doublets), *dt* (doublet of triplets), and *br* (broad). Coupling constants (*J*) were recorded in hertz

(Hz). The number of proton atoms (*n*) for a given resonance was indicated by *n*H. The number of carbon atoms (*n*) for a given resonance was indicated by *n*C. High Resolution Mass Spectrometry (HRMS) analysis was obtained using Electrospray Ionization (ESI) and reported in units of mass of charge ratio (m/z). ESI was acquired using a Bruker Compact (ESI-TOF). Mass samples were dissolved in MeOH (HPLC [High Performance Liquid Chromatography] grade) unless otherwise stated. Optical rotations were determined on a INESA SGW®-2. Optical rotations were recorded on a polarimeter with a sodium lamp of wavelength 589 nm and reported as follows; $[\alpha]_{\lambda}^{T°C}$ (*c* = g/100 mL, solvent). Melting points were determined on a melting point apparatus (OptiMelt MPA100). Enantiomeric excesses were determined by chiral HPLC analysis using Thermo Scientific Ultimate 3000. UV detection was monitored at 254 nm and 210 nm at the same time. HPLC samples were dissolved in HPLC grade isopropanol (IPA) unless otherwise stated.

Luminescence Measurements were performed on a Nanolog infrared fluorescence spectrometer (Nanolog FL3-2iHR, US) equipped with a continuous Xe source for steady state measurements and a Xe flashlight source for the observation of phosphorescence spectra. Fluorescence spectra were recorded at ambient conditions in 10×10 mm quartz cuvettes (Hellma, Suprasil). Luminescence spectra at cryogenic conditions (77 K) were recorded in quartz tubes (inner ø = 4 mm) in a small quartz Dewar vessel which was filled with liquid nitrogen. All solutions were handled under dry nitrogen and degassed (15 min in an ultrasound bath) to exclude oxygen as triplet quencher. A longpass filter (Schott Advanced Optics, WG300 or Hebo Spezialglass, UV280) was introduced in the emission-beam path to prevent the scattered excitation light to pass the emission monochromator at higher order wavelengths where necessary.

Materials

All commercial reagents were purchased with the highest purity grade. They were used without further purification unless specified. All solvents used, mainly petroleum ether (PE) and ethyl acetate (EA) were distilled. Anhydrous toluene, $tBuC_6H_5$ and EtC_6H_5 were freshly distilled from sodium/benzophenone before use. All compounds synthesized were stored in a −80 °C freezer and light-sensitive compounds were protected with aluminum foil.

2. Optimization of reaction conditions

Table S1. Optimization of reaction conditions[a]

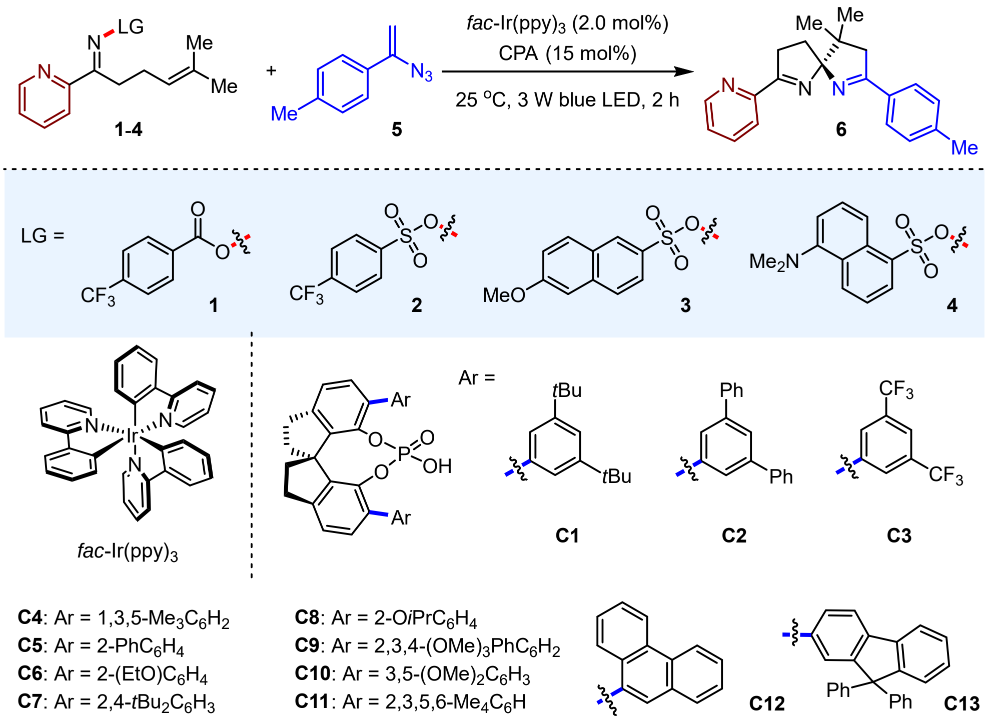

entry	CPA	1-4	additive	solvent (mL)	base (equiv.)	ee (%)[b]
1	**C1**	**1**	-	$CHCl_3$ (0.5)	K_2CO_3 (1.5)	15
2	**C2**	**1**	-	$CHCl_3$ (0.5)	K_2CO_3 (1.5)	10
3	**C3**	**1**	-	$CHCl_3$ (0.5)	K_2CO_3 (1.5)	8
4	**C4**	**1**	-	$CHCl_3$ (0.5)	K_2CO_3 (1.5)	2
5	**C5**	**1**	-	$CHCl_3$ (0.5)	K_2CO_3 (1.5)	4
6	**C6**	**1**	-	$CHCl_3$ (0.5)	K_2CO_3 (1.5)	0
7	**C7**	**1**	-	$CHCl_3$ (0.5)	K_2CO_3 (1.5)	2
8	**C8**	**1**	-	$CHCl_3$ (0.5)	K_2CO_3 (1.5)	4
9	**C9**	**1**	-	$CHCl_3$ (0.5)	K_2CO_3 (1.5)	2
10	**C10**	**1**	-	$CHCl_3$ (0.5)	K_2CO_3 (1.5)	2
11	**C11**	**1**	-	$CHCl_3$ (0.5)	K_2CO_3 (1.5)	0
12	**C12**	**1**	-	$CHCl_3$ (0.5)	K_2CO_3 (1.5)	8
13	**C13**	**1**	-	$CHCl_3$ (0.5)	K_2CO_3 (1.5)	0

14	**C14**	**1**	-	$CHCl_3$ (0.5)	K_2CO_3 (1.5)	14
15	**C15**	**1**	-	$CHCl_3$ (0.5)	K_2CO_3 (1.5)	12
16	**C1**	**1**	-	$^tBuC_6H_5$ (0.5)	K_2CO_3 (1.5)	-
17	**C1**	**2**	-	$^tBuC_6H_5$ (0.5)	K_2CO_3 (1.5)	31
18	**C1**	**3**	-	$^tBuC_6H_5$ (0.5)	K_2CO_3 (1.5)	40
19	**C1**	**4**	-	$^tBuC_6H_5$ (0.5)	K_2CO_3 (1.5)	61
20	**C1**	**4**	H_2O (5 μL)	$^tBuC_6H_5$ (0.5)	K_2CO_3 (1.5)	77
21	**C1**	**4**	H_2O (10 μL)	$^tBuC_6H_5$ (0.5)	K_2CO_3 (1.5)	65
22	**C1**	**4**	H_2O (15 μL)	$^tBuC_6H_5$ (0.5)	K_2CO_3 (1.5)	75
23	**C1**	**4**	H_2O (20 μL)	$^tBuC_6H_5$ (0.5)	K_2CO_3 (1.5)	73
25	**C1**	**4**	H_2O (5 μL)	$^tBuC_6H_5$ (0.6)	K_2CO_3 (1.5)	79
26	**C1**	**4**	H_2O (5 μL)	$^tBuC_6H_5$ (0.7)	K_2CO_3 (1.5)	71
27	**C1**	**4**	H_2O (5 μL)	$^tBuC_6H_5$ (0.8)	K_2CO_3 (1.5)	77
28	**C1**	**4**	H_2O (5 μL)	$^tBuC_6H_5$ (0.9)	K_2CO_3 (1.5)	73
29	**C1**	**4**	H_2O (5 μL)	$^iPrC_6H_5$ (0.6)	K_2CO_3 (1.5)	25
30	**C1**	**4**	H_2O (5 μL)	Toluene (0.6)	K_2CO_3 (1.5)	77
31	**C1**	**4**	H_2O (5 μL)	1-^{t}Bu-3,5-$Me_2C_6H_3$ (0.6)	K_2CO_3 (1.5)	73
32	**C1**	**4**	H_2O (5 μL)	EtC_6H_5 (0.6)	K_2CO_3 (1.5)	81
33	**C1**	**4**	H_2O (5 μL)	EtC_6H_5 (0.6)	$KHCO_3$ (1.5)	73
34	**C1**	**4**	H_2O (5 μL)	EtC_6H_5 (0.6)	K_3PO_4 (1.5)	65
35	**C1**	**4**	H_2O (5 μL)	EtC_6H_5 (0.6)	K_2HPO_4 (1.5)	53
36	**C1**	**4**	H_2O (5 μL)	EtC_6H_5 (0.6)	Na_2CO_3 (1.5)	69
37	**C1**	**4**	H_2O (5 μL)	EtC_6H_5 (0.6)	$NaHCO_3$ (1.5)	67
38	**C1**	**4**	H_2O (5 μL)	EtC_6H_5 (0.6)	Na_3PO_4 (1.5)	37
39	**C1**	**4**	H_2O (5 μL)	EtC_6H_5 (0.6)	Na_2HPO_4 (1.5)	49
40	**C1**	**4**	H_2O (5 μL)	EtC_6H_5 (0.6)	Cs_2CO_3 (1.5)	59
41	**C1**	**4**	H_2O (5 μL)	EtC_6H_5 (0.6)	K_2CO_3 (1.0)	70

42	**C1**	**4**	H_2O (5 μL)	EtC_6H_5 (0.6)	K_2CO_3 (2.0)	75
43	**C1**	**4**	H_2O (5 μL)	EtC_6H_5 (0.6)	K_2CO_3 (2.5)	79
44	**C1**	**4**	H_2O (5 μL)	EtC_6H_5 (0.6)	K_2CO_3 (3.0)	73
45	**C1**	**4**	KF (1.0 equiv.) + H_2O (5 μL)	EtC_6H_5 (0.6)	K_2CO_3 (1.5)	90
46	**C1**	**4**	LiF (1.0 equiv.) + H_2O (5 μL)	EtC_6H_5 (0.6)	K_2CO_3 (1.5)	87
47	**C1**	**4**	NH_4F (1.0 equiv.) + H_2O (5 μL)	EtC_6H_5 (0.6)	K_2CO_3 (1.5)	21

[a]0.02 mmol scale and irradiation distance = 2.0 cm. [b]Determined by chiral HPLC analysis.

3. Experimental procedures

3.1 Synthesis of olefinic sulfonyl oximes

General procedure A

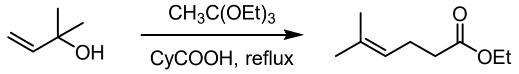

According to a literature procedure:[1] A solution of 1,1-dimethylallyl alcohol (1.0 equiv.), triethyl orthoacetate (10 equiv.) and cyclohexanecarboxylic acid (0.1 equiv.) was stirred under reflux conditions for 3 h. The reaction mixture was then cooled to room temperature and extracted three times with diethyl ether. The combined organic layers were washed with 10 % aqueous HCl, saturated aqueous $NaHCO_3$ solution, water and brine. The organic layer was dried with $MgSO_4$ and concentrated under reduced pressure. The residue was purified by silica gel column chromatography using hexane/ethyl acetate (30:1) as eluent to afford the corresponding ester.

General procedure B

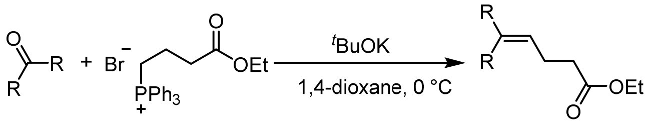

According to a literature procedure:[2] (4-Ethoxy-4-oxobutyl) triphenylphosphonium bromide (1.3 equiv.) was placed in a flame-dried round-bottom flask, and anhydrous THF (0.3 M solution for the ketone) was added under an argon atmosphere. The mixture was cooled to 0 °C and treated with *t*BuOK (1.5 equiv.). After stirring for 1 hour, the ketone (1.0 equiv.) was added dropwise. Upon complete addition, the reaction mixture was gradually warmed to room temperature and stirred for an additional 12 hours. The reaction was then quenched with water and extracted with diethyl ether. The combined organic phase was washed with brine and concentrated under reduced pressure. The residue was purified by silica gel chromatography, eluting with a hexanes/ethyl acetate mixture, to yield the desired product.

General procedure C

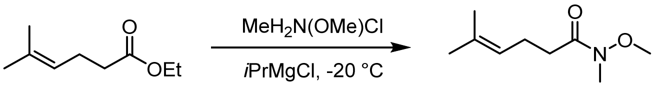

According to a literature procedure:[1] to a solution of ester (1.0 equiv.) in dry THF and *N*,*O*-dimethylhydroxylamide hydrochloride (3.0 equiv.) was slowly added a freshly prepared solution of *i*-PrMgCl in THF (1.0 M, 4.0 equiv.) at –20 °C. The mixture was stirred for 30 min at –10 °C and then hydrolyzed with NH_4Cl solution (20 wt % in H_2O, 100 mL), followed by three times extraction with diethyl ether (100 mL). The combined organic layers were dried with $MgSO_4$ and concentrated under reduced pressure. The resulting residue was purified by flash column chromatography to afford the desired Weinreb amide.

General procedure D

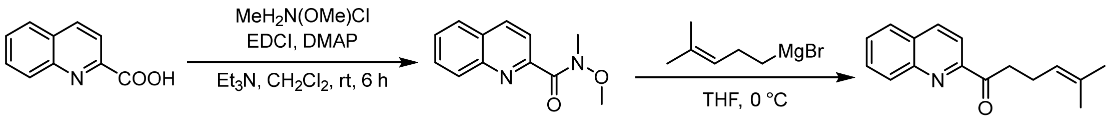

According to a literature procedure:[3,4] Picolinic acid (1.0 equiv.), DMAP (0.02 equiv.), EDCI (1.2 equiv.), and *N*,*O*-dimethyl hydroxylamine hydrochloride (1.1 equiv.) were weighed into a flame-dried round-bottom flask, which was subsequently sealed with a septum. Anhydrous dichloromethane (CH_2Cl_2) was added via syringe, and the resulting solution was cooled to 0 °C using an ice bath. Triethylamine (3.0 equiv.) was then introduced through a syringe, and the mixture was stirred at 0 °C for 15 minutes. The ice bath was removed, and stirring was continued for an additional 6 hours at room temperature. The reaction was quenched by the addition of water. After separating the organic layer, the aqueous phase was neutralized with a saturated aqueous solution of sodium bicarbonate ($NaHCO_3$) until a pH of 7−8 was achieved. The aqueous layer was then washed with CH_2Cl_2. All organic layers were combined and washed successively with saturated aqueous $NaHCO_3$ and saturated aqueous sodium chloride (NaCl), followed by drying over magnesium sulfate ($MgSO_4$). The mixture was filtered and concentrated under reduced pressure to yield the amide as a white solid.

To a flame-dried round-bottom flask equipped with a condenser and stir bar, magnesium turnings and a catalytic amount of 1,2-dibromoethane were added. A solution of 5-bromo-2-methylpent-2-ene (1.2 equiv.) in anhydrous THF was then introduced gradually, first in portions and subsequently dropwise. The reaction mixture was stirred at room temperature for 1 hour. Following this, the reaction was cooled to 0 °C, and a solution of amide (1.0 equiv.) in THF was added dropwise. The mixture was allowed to stir under a nitrogen atmosphere overnight. After completion, the reaction was cooled to 0 °C and quenched with a saturated

aqueous solution of NH_4Cl. The organic layer was extracted with diethyl ether, washed with saturated aqueous NH_4Cl and brine, and then dried over anhydrous Na_2SO_4. The solvent was removed under reduced pressure to yield a clear oil. The crude product was subsequently purified by silica gel chromatography to afford the desired compound.

General procedure E

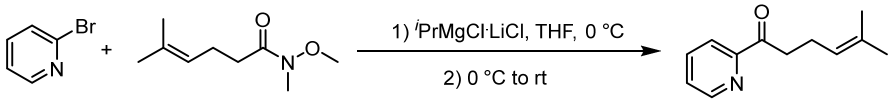

According to a literature procedure:[5] A dry Schlenk tube equipped with a stirring bar was charged with 2-bromopyridine (1.0 equiv.) and dry THF. The solution was cooled to 0 °C, and *i*-PrMgCl•LiCl (1 equiv., 1.3 M in THF) was added dropwise. The mixture was stirred at 0 °C for 4 hours, after which a solution of Weinreb amide (1.0 equiv.) in THF was introduced. The reaction was allowed to warm to room temperature and stirred overnight. Subsequently, 5 mL of water and diethyl ether were added to the mixture. The layers were separated, and the aqueous phase was extracted with diethyl ether. The combined organic layers were dried, filtered, and evaporated. Purification by column chromatography on silica gel yielded the ketone as an oil.

General procedure F

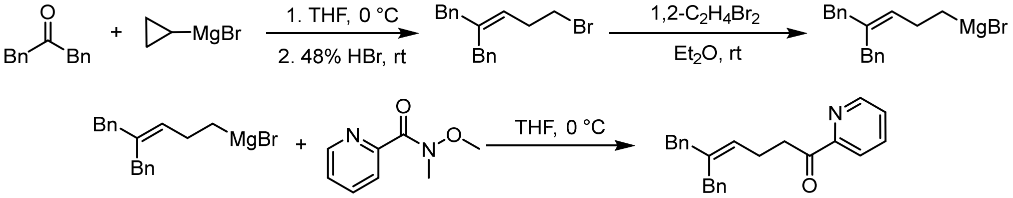

According to a literature procedure:[6] The freshly prepared Grignard reagent was cooled in an ice bath and a solution of 1,3-diphenylpropan-2-one (1.0 equiv) in Et_2O was added dropwise over 10 minutes, which was then stirred overnight at room temperature. After cooling to 0 ºC, it was quenched by saturated NH_4Cl. The separated aqueous layer was extracted with Et_2O, and the combined organic layers were washed with water (10 mL), brine (10 mL), dried over Na_2SO_4, filtered, and concentrated. Crude product was obtained as a pale-yellow oil, which was directly used in next step without further purification.

To the above crude product at room temperature was added dropwise 48% aq HBr (1.6 equiv) over 20 minutes with vigorous stirring, which was then stirred for an additional two hours. Water and Et_2O were added and the resulting mixture was stirred for 5 minutes. The separated aqueous layer was extracted with Et_2O, and the combined organic layers were washed sequentially with water and brine. It was then dried over Na_2SO_4, filtered, concentrated to give crude as a pale-yellow oil, The crude product was purified via silica gel chromatography to give the titled compound.

To a flame-dried 100-mL round bottom flask equipped with stir bar was added magnesium turnings (1.4 equiv.) and a catalytic amount of 1,2-dibromoethane. To the mixture was added (2-(3-bromopropylidene)propane-1,3-diyl)dibenzene (1.2 equiv.) dissolved in anhydrous THF, first portion-wise and then dropwise. The reaction mixture was stirred at rt for 1 hours. The reaction was cooled to room temperature and then 0 °C, after which Weinreb amide (1.0 equiv.) dissolved in THF was added dropwise. The reaction was allowed to stir under nitrogen overnight. The reaction was cooled to 0 °C and quenched with saturated aqueous NH_4Cl, extracted with Et_2O, washed with saturated aqueous NH_4Cl and brine, dried over anhydrous Na_2SO_4, and concentrated in vacuo to afford a clear oil. The crude product was purified via silica gel chromatography to give the titled compound.

General procedure G

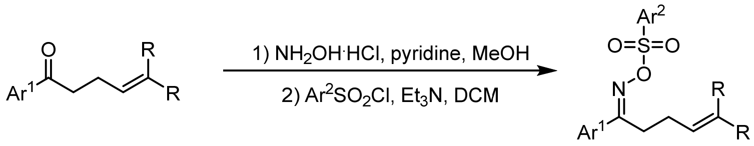

According to a literature procedure:[7] A solution of the ketone (1.0 equiv.), hydroxylamine hydrochloride (1.2 equiv.), and pyridine (1.5 equiv.) in methanol was stirred at room temperature for 6 hours. Following this reaction period, the solvent was removed under reduced pressure, and the residue was dissolved in ethyl acetate for extraction. The organic phase was washed twice with saturated sodium chloride NaCl, dried over Na_2SO_4, and concentrated under vacuum. The resulting residue was then dissolved in dry CH_2Cl_2, and triethylamine (1.5 equiv.) was added. Benzoyl chloride (1.2 equiv.) was introduced dropwise while maintaining the

temperature at 0 °C, and the mixture was stirred at this temperature for an additional 2 hours. Subsequently, the reaction mixture was extracted with CH_2Cl_2. The combined organic phases were dried over Na_2SO_4 and concentrated under vacuum. Further purification was achieved through column chromatography on silica gel, yielding the olefinic sulfonyl oximes.

Characterization data for representative olefinic sulfonyl oximes

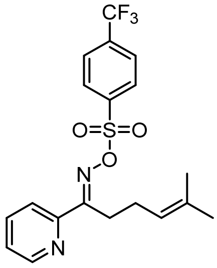

(*E*)-5-methyl-1-(pyridin-2-yl)hex-4-en-1-one *O*-((4-(trifluoromethyl)phenyl)sulfonyl) oxime (2) was synthesized according to general procedure **A, C, E** and **G**. White solid; Mp 95.7−96.5 °C; **^{1}H NMR** (400 MHz, $CDCl_3$) δ 8.62 (d, *J* = 4.7 Hz, 1H), 8.18 (d, *J* = 8.2 Hz, 2H), 7.83 (d, *J* = 8.3 Hz, 2H), 7.76 – 7.60 (m, 2H), 7.34 (dd, *J* = 8.8, 4.7 Hz, 1H), 5.08 (t, *J* = 7.3 Hz, 1H), 3.05 (t, *J* = 7.7 Hz, 2H), 2.22 (q, *J* = 7.5 Hz, 2H), 1.59 (s, 3H), 1.48 (s, 3H); **^{13}C NMR** (101 MHz, $CDCl_3$) δ 168.3, 151.0, 149.5, 139.4, 136.8, 135.7 (q, *J* = 33.3 Hz), 133.6, 129.6, 126.29 (q, *J* = 3.7 Hz), 125.4, 124.6 (q, *J* = 222.7 Hz), 122.4, 122.2, 26.9, 25.7, 25.2, 17.7; **^{19}F NMR** (376 MHz, $CDCl_3$) δ -63.27; **HRMS (ESI)** 413.1140 m/z (M + H^+), calc. for $C_{19}H_{20}F_3N_2O_3S^+$ 413.1141.

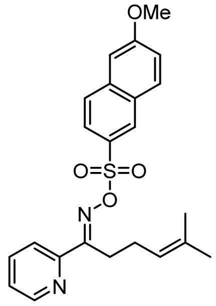

(*E*)-5-methyl-1-(pyridin-2-yl)hex-4-en-1-one *O*-((6-methoxynaphthalen-2-yl)sulfonyl) oxime (3) was synthesized according to general procedure **A**, **C**, **E** and **G**. White solid; Mp 90.5−91.3 °C; **^{1}H NMR** (400 MHz, $CDCl_3$) δ 8.70 (d, *J* = 4.5 Hz, 1H), 8.64 (s, 1H), 8.06 (dd, *J* = 8.7, 1.5 Hz, 1H), 7.98 (dd, *J* = 13.3, 8.9 Hz, 2H), 7.83 (d, *J* = 7.9 Hz, 1H), 7.77 (dd, *J* = 10.8, 4.5 Hz, 1H), 7.39 (dt, *J* = 11.1, 4.5 Hz, 2H), 7.29 (s, 1H), 5.20 (t, *J* = 7.1 Hz, 1H), 4.06 (s, 3H), 3.15 (t, *J* = 7.8 Hz, 2H), 2.31 (dd, *J* = 15.0, 7.4 Hz, 2H), 1.68 (s, 3H), 1.58 (s, 3H); **^{13}C**

NMR (101 MHz, $CDCl_3$) δ 167.2, 160.5, 151.3, 149.1, 137.4, 136.7, 133.4, 131.0, 130.7, 130.1, 127.8, 127.4, 125.1, 124.2, 122.5, 122.2, 120.8, 105.9, 55.6, 26.7, 25.6, 25.1, 17.6. **HRMS** (ESI) 425.1531 m/z (M + H^+), calc. for $C_{23}H_{25}N_2O_4S^+$ 425.1530.

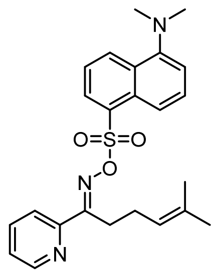

(*E*)-5-methyl-1-(pyridin-2-yl)hex-4-en-1-one *O*-((5-(dimethylamino)naphthalen-1-yl)sulfonyl) oxime (4) was synthesized according to general procedure **A**, **C**, **E** and **G**. Kelly solid; Mp 89.4–90.2 °C; **^{1}H NMR** (600 MHz, $CDCl_3$) δ 8.62 (d, *J* = 8.5 Hz, 1H), 8.56 – 8.50 (m, 1H), 8.44 – 8.37 (m, 2H), 7.62 – 7.53 (m, 4H), 7.23 (m, 1H), 7.19 (d, *J* = 7.5 Hz, 1H), 5.12 – 5.04 (m, 1H), 3.06 – 3.01 (m, 2H), 2.88 (s, 6H), 2.18 (m, 2H), 1.56 (s, 3H), 1.45 (s, 3H); **^{13}C NMR** (151 MHz, $CDCl_3$) δ 167.2, 151.2, 149.1, 136.3, 133.2, 131.8, 131.4, 130.1, 129.8, 128.7, 124.9, 123.1, 122.6, 122.1, 119.5, 115.5, 45.4, 26.6, 25.5, 25.2, 17.5; **HRMS** (ESI) 438.1844 (M + H^+), calc. for $C_{24}H_{28}N_3O_3S^+$ 438.1846.

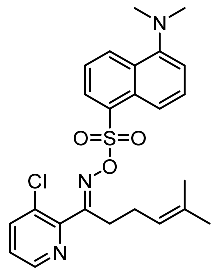

(*E*)-1-(3-chloropyridin-2-yl)-5-methylhex-4-en-1-one *O*-((5-(dimethylamino)naphthalen-1-yl)sulfonyl) oxime (4a) was synthesized according to general procedure **A**, **C**, **E** and **G**. Kelly solid; Mp 102.5 - 103.4 °C; **^{1}H NMR** (600 MHz, $CDCl_3$) δ 8.67 (d, *J* = 5.3 Hz, 1H), 8.45 (d, *J* = 7.4 Hz, 1H), 8.43 (d, *J* = 5.3 Hz, 2H), 7.62 (m, 2H), 7.54 (d, *J* = 1.8 Hz, 1H), 7.24 (dd, *J* = 5.2, 1.9 Hz, 1H), 7.22 (d, *J* = 6.6 Hz, 1H), 5.06 (t, *J* = 7.2 Hz, 1H), 3.03 – 2.97 (m, 2H), 2.90 (s, 6H), 2.16 (m, 2H), 1.56 (s, 3H), 1.45 (s, 3H); **^{13}C NMR** (151 MHz, $CDCl_3$) δ 166.4, 152.9, 149.9, 144.6, 133.6, 132.1, 132.0, 131.3, 130.3, 129.8, 128.8, 125.3, 123.4, 122.5, 122.4, 119.8, 115.8, 77.4, 77.2, 76.9, 45.6, 26.7, 25.6, 25.3, 17.6; **HRMS** (ESI) 472.1452 m/z (M +H^+), calc. for $C_{24}H_{27}ClN_3O_3S^+$ 472.1456.

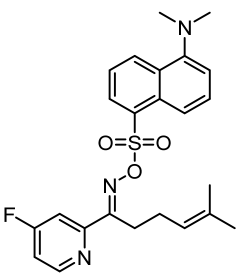

(*E*)-1-(4-fluoropyridin-2-yl)-5-methylhex-4-en-1-one *O*-((5-(dimethylamino)naphthalen-1-yl)sulfonyl) oxime (4b) was synthesized according to general procedure **A**, **C**, **E** and **G**. Kelly solid; 96.2 - 97.0 °C; **^{1}H NMR** (400 MHz, CD_3OD) δ 8.69 (d, *J* = 8.5 Hz, 1H), 8.55 (dd, *J* = 7.9, 5.4 Hz, 1H), 8.42 (dd, *J* = 7.4, 1.2 Hz, 1H), 8.37 (d, *J* = 8.7 Hz, 1H), 7.66 (m, 2H), 7.30 (d, *J* = 7.6 Hz, 1H), 7.18 (m, 2H), 5.02 – 4.93 (m, 1H), 3.00 (t, *J* = 7.6 Hz, 2H), 2.87 (s, 6H), 2.14 (q, *J* = 7.5 Hz, 2H), 1.47 (s, 3H), 1.34 (s, 3H); **^{13}C NMR** (101 MHz, $CDCl_3$) δ 168.6 (d, *J* = 262.7 Hz), 166.4 (d, *J* = 3.6 Hz), 154.5, 151.5 (d, *J* = 7.1 Hz), 133.5, 132.0, 131.3, 130.2, 129.9, 128.9, 123.4, 122.5, 119.6, 115.7, 113.0 (d, *J* = 16.7 Hz), 109.8 (d, *J* = 18.5 Hz), 45.6, 26.7, 25.6, 25.3, 17.6; **^{19}F NMR** (376 MHz, $CDCl_3$) δ -101.67. **HRMS** (ESI) 456.1750 m/z (M + H^+), calc. for $C_{24}H_{27}FN_3O_3S^+$ 456.1752.

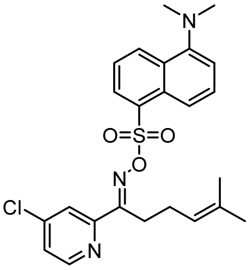

(*E*)-1-(4-chloropyridin-2-yl)-5-methylhex-4-en-1-one *O*-((5-(dimethylamino)naphthalen-1-yl)sulfonyl) oxime (4c) was synthesized according to general procedure **A**, **C**, **E** and **G**. Kelly solid; Mp 103.3−104.1 °C; **^{1}H NMR (**600 MHz, $CDCl_3$) δ 8.66 (d, *J* = 7.5 Hz, 1H), 8.45 (dd, *J* = 7.4, 1.2 Hz, 1H), 8.43 – 8.36 (m, 2H), 7.61 (m, 2H), 7.56 – 7.52 (m, 1H), 7.24 (dd, *J* = 5.3, 2.0 Hz, 1H), 7.21 (d, *J* = 7.3 Hz, 1H), 5.10 – 4.88 (m, 1H), 3.07 – 2.94 (m, 2H), 2.89 (s, 6H), 2.16 (m, 2H), 1.56 (s, 3H), 1.45 (s, 3H); **^{13}C NMR** (151 MHz, $CDCl_3$) δ 166.4, 152.8, 149.9, 144.5, 133.5, 132.0, 131.2, 130.2, 129.8, 128.8, 125.2, 123.4, 122.4, 122.3, 115.7, 77.3, 77.1, 76.9, 45.6, 26.6, 25.6, 25.2, 17.6; **HRMS** (ESI) 472.1454 m/z (M + H^+), calc. for $C_{24}H_{27}ClN_3O_3S^+$ 472.1456.

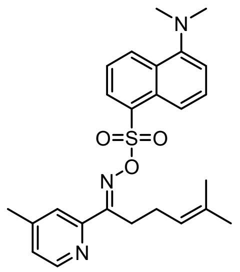

(*E*)-5-methyl-1-(4-methylpyridin-2-yl)hex-4-en-1-one *O*-((5-(dimethylamino)naphthalen-1-yl)sulfonyl) oxime (4d) was synthesized according to general procedure **A**, **C**, **E** and **G**. Kelly solid; Mp 93.9–94.8 °C; **^{1}H NMR** (600 MHz, $CDCl_3$) δ 8.64 (d, *J* = 7.8 Hz, 1H), 8.47 – 8.39 (m, 2H), 8.38 (d, *J* = 4.9 Hz, 1H), 7.65 – 7.50 (m, 2H), 7.39 – 7.36 (m, 1H), 7.20 (d, *J* = 7.4 Hz, 1H), 7.06 (m, 1H), 5.10 – 5.05 (m, 1H), 3.06 – 2.96 (m, 2H), 2.89 (s, 6H), 2.27 (s, 3H), 2.16 (m, 2H), 1.57 (s, 3H), 1.46 (s, 3H); **^{13}C NMR** (151 MHz, $CDCl_3$) δ 167.5, 151.0, 148.8, 147.6, 133.2, 131.7, 131.5, 130.2, 129.7, 128.6, 125.9, 123.2, 122.9, 122.6, 115.6, 45.5, 26.7, 25.5, 25.2, 20.9, 17.6; **HRMS** (ESI) 452.2001 m/z (M + H^+), calc. for $C_{25}H_{30}N_3O_3S^+$ 452.2002.

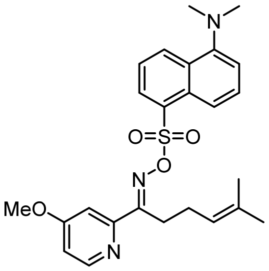

(*E*)-1-(4-methoxypyridin-2-yl)-5-methylhex-4-en-1-one *O*-((5-(dimethylamino)naphthalen-1-yl)sulfonyl) oxime (4e) was synthesized according to general procedure **A**, **C**, **E** and **G**. Kelly solid; Mp 89.5–90.3 °C; **^{1}H NMR** (600 MHz, $CDCl_3$) δ 8.63 (d, *J* = 8.2 Hz, 1H), 8.47 – 8.37 (m, 2H), 8.34 (d, *J* = 5.7 Hz, 1H), 7.59 (m, 2H), 7.20 (d, *J* = 7.5 Hz, 1H), 7.02 (d, *J* = 2.5 Hz, 1H), 6.76 (dd, *J* = 5.7, 2.6 Hz, 1H), 5.14 – 5.00 (m, 1H), 3.76 (s, 3H), 3.05 – 2.95 (m, 2H), 2.89 (s, 6H), 2.16 (m, 2H), 1.57 (s, 3H), 1.47 (s, 3H); **^{13}C NMR** (151 MHz, $CDCl_3$) δ 167.3, 165.9, 152.9, 150.3, 133.3, 131.9, 131.8, 131.5, 130.3, 129.9, 128.8, 127.2, 123.2, 122.7, 115.7, 111.7, 107.7, 77.4, 77.2, 76.9, 55.4, 45.6, 26.9, 25.7, 25.3, 17.7; **HRMS** (ESI) 468.1956 m/z (M + H^+), calc. for $C_{25}H_{30}N_3O_4S^+$ 468.1952.

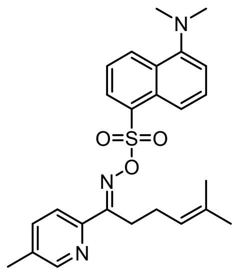

(*E*)-5-methyl-1-(5-methylpyridin-2-yl)hex-4-en-1-one *O*-((5-(dimethylamino)naphthalen-1-yl)sulfonyl) oxime (4f) was synthesized according to general procedure **A**, **C**, **E** and **G**. Kelly solid; Mp 68.7−69.7 °C; **^{1}H NMR** (600 MHz, $CDCl_3$) δ 8.62 (d, *J* = 8.4 Hz, 1H), 8.41 (m, 2H), 8.35 (s, 1H), 7.62 – 7.52 (m, 2H), 7.46 (d, *J* = 8.1 Hz, 1H), 7.36 (dd, *J* = 8.1, 1.7 Hz, 1H), 7.19 (d, *J* = 7.5 Hz, 1H), 5.09 (t, *J* = 7.2 Hz, 1H), 3.00 (m, 2H), 2.88 (s, 6H), 2.29 (s, 3H), 2.16 (m, 2H), 1.58 (s, 3H), 1.47 (s, 3H); **^{13}C NMR** (151 MHz, $CDCl_3$) δ 167.3, 149.6, 148.6, 136.9, 135.1, 133.3, 131.9, 131.8, 131.6, 130.2, 129.9, 128.8, 123.3, 122.8, 121.7, 115.6, 45.6, 26.7, 25.7, 25.4, 18.5, 17.7; **HRMS** (ESI) 452.2000 m/z (M + H^+), calc. for $C_{25}H_{30}N_3O_3S^+$ 452.2002.

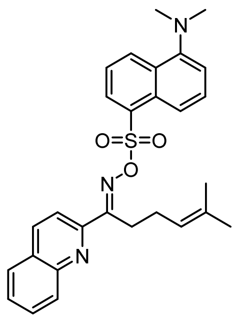

(*E*)-5-methyl-1-(quinolin-2-yl)hex-4-en-1-one *O*-((5-(dimethylamino)naphthalen-1-yl)sulfonyl) oxime (4g) was synthesized according to general procedure **D** and **G**. Yellow solid; Mp 77.3−78.1 °C; **^{1}H NMR** (600 MHz, $CDCl_3$) δ 8.63 (d, *J* = 7.3 Hz, 1H), 8.47 (m, 2H), 8.00 (m, 2H), 7.81 – 7.48 (m, 6H), 7.19 (d, *J* = 6.7 Hz, 1H), 5.19 (m, 1H), 3.25 – 3.09 (m, 2H), 2.87 (s, 6H), 2.27 (m, 2H), 1.60 (s, 3H), 1.54 (s, 3H); **^{13}C NMR** (151 MHz, $CDCl_3$) δ 167.3, 151.1, 147.6, 136.2, 133.4, 131.9, 131.9, 131.5, 130.2, 130.1, 129.9, 129.8, 128.8, 128.5, 127.7, 127.6, 123.3, 122.9, 119.6, 119.1, 115.6, 45.6, 26.6, 25.7, 25.6, 17.7; **HRMS** (ESI) 488.2004 m/z (M + H^+), calc. for $C_{28}H_{30}N_3O_3S^+$ 488.2002.

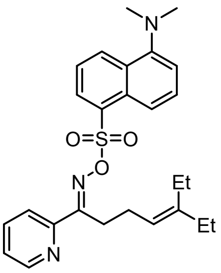

(*E*)-5-ethyl-1-(pyridin-2-yl)hept-4-en-1-one *O*-((5-(dimethylamino)naphthalen-1-yl)sulfonyl) oxime (4h) was synthesized according to general procedure **A**, **C**, **E** and **G**. Kelly solid; Mp 82.9−83.7 °C; **^{1}H NMR** (600 MHz, $CDCl_3$) δ 8.62 (d, *J* = 8.5 Hz, 1H), 8.53 (d, *J* = 4.7 Hz, 1H), 8.47 – 8.32 (m, 2H), 7.61 – 7.57 (m, 2H), 7.57 – 7.52 (m, 2H), 7.23 (m, 1H), 7.19 (d, *J* = 7.5 Hz, 1H), 5.03 (t, *J* = 7.2 Hz, 1H), 3.09 – 2.99 (m, 2H), 2.21 (m, 2H), 1.92 – 1.81 (m, 4H), 0.86 (t, *J* = 6.0 Hz, 3H), 0.83 (t, *J* = 6.2 Hz, 3H); **^{13}C NMR** (151 MHz, $CDCl_3$) δ 167.4, 151.4, 149.2, 144.5, 136.4, 131.9, 131.9, 131.5, 130.2, 129.9, 128.8, 125.0, 123.3, 122.2, 120.7, 119.6, 115.6, 45.6, 29.1, 26.98, 24.8, 23.2, 13.3, 12.8; **HRMS** (ESI) 466.2160 m/z (M + H^+), calc. for $C_{26}H_{32}N_3O_3S^+$ 466.2159.

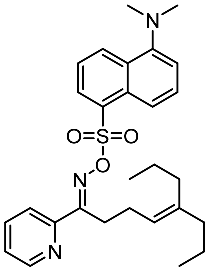

(*E*)-5-propyl-1-(pyridin-2-yl)oct-4-en-1-one *O*-((5-(dimethylamino)naphthalen-1-yl)sulfonyl) oxime (4i) was synthesized according to general procedure **A**, **C**, **E** and **G**. Kelly oil; **^{1}H NMR** (600 MHz, $CDCl_3$) δ 8.61 (d, *J* = 8.5 Hz, 1H), 8.54 – 8.51 (m, 1H), 8.45 – 8.37 (m, 2H), 7.57 (m, 4H), 7.23 – 7.20 (m, 1H), 7.18 (d, *J* = 7.5 Hz, 1H), 5.11 (t, *J* = 7.2 Hz, 1H), 3.11 – 2.98 (m, 2H), 2.86 (s, 6H), 2.22 (m, 2H), 1.91 – 1.82 (m, 4H), 1.30 (m, 4H), 0.82 (m, 6H); **^{13}C NMR** (151 MHz, $CDCl_3$) δ 167.3, 151.9, 151.3, 149.1, 141.1, 136.4, 131.9, 131.8, 131.5, 130.2, 129.9, 128.8, 125.0, 123.2, 122.8, 122.2, 119.5, 115.6, 45.5, 38.9, 32.1, 27.0, 25.0, 21.6, 21.2, 14.2, 14.0; **HRMS** (ESI) 494.2472 m/z (M + H^+), calc. for $C_{28}H_{36}N_3O_3S^+$ 494.2472.

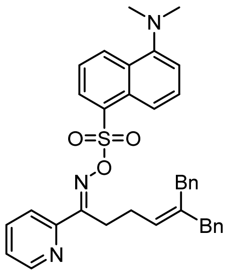

(*E)*-5-benzyl-6-phenyl-1-(pyridin-2-yl)hex-4-en-1-one *O*-((5-(dimethylamino)naphthalen-1-yl)sulfonyl) oxime (4j) was synthesized according to general procedure **F** and **G**. Kelly oil; **^{1}H NMR** (600 MHz, $CDCl_3$) δ8.45 (d, *J* = 8.5 Hz, 1H), 8.35 (d, *J* = 4.8 Hz, 1H), 8.29 (dd, *J* = 7.3, 0.8 Hz, 1H), 8.23 (d, *J* = 8.7 Hz, 1H), 7.46 – 7.35 (m, 3H), 7.33 – 7.27 (m, 1H), 7.13 – 7.02 (m, 7H), 6.95 (d, *J* = 7.5 Hz, 1H), 6.91 (d, *J* = 7.2 Hz, 2H), 6.81 (d, *J* = 6.5 Hz, 2H), 5.20 (t, *J* = 7.3 Hz, 1H), 3.06 – 2.99 (m, 2H), 2.94 (s, 2H), 2.90 (s, 2H), 2.67 (s, 6H), 2.27 (m, 2H); **^{13}C NMR** (151 MHz, $CDCl_3$) δ 166.8, 151.8, 150.9, 149.1, 139.7, 139.7, 139.1, 136.4, 131.9, 131.8, 131.3, 130.1, 129.8, 129.1, 128.8, 128.7, 128.4, 128.2, 126.2, 126.0, 125.9, 125.1, 123.1, 122.1, 119.3, 115.5, 77.4, 77.2, 76.9, 45.4, 42.8, 35.1, 26.6, 25.4; **HRMS** (ESI) 590.2470 m/z (M + H^+), calc. for $C_{36}H_{36}N_3O_3S^+$ 590.2472.

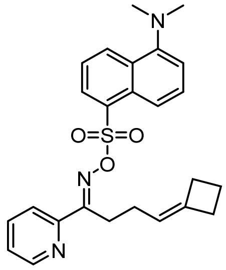

(*E*)-4-cyclobutylidene-1-(pyridin-2-yl)butan-1-one *O*-((5-(dimethylamino)naphthalen-1-yl)sulfonyl) oxime (4k) was synthesized according to general procedure **A**, **C**, **E** and **G**. Grayish green solid; Mp 105.3−106.1 °C; **^{1}H NMR** (400 MHz, $CDCl_3$) δ 8.86 (d, *J* = 8.4 Hz, 1H), 8.76 (d, *J* = 4.7 Hz, 1H), 8.65 (m, 2H), 7.87 – 7.75 (m, 4H), 7.53 – 7.34 (m, 2H), 5.20 (m, 1H), 3.28 (t, *J* = 7.7 Hz, 2H), 2.68 (t, *J* = 7.5 Hz, 2H), 2.61 (t, *J* = 7.6 Hz, 2H), 2.31 (m, 2H), 2.07 – 1.96 (m, 2H); **^{13}C NMR** (101 MHz, $CDCl_3$) δ 167.3, 156.8, 151.4, 149.2, 141.8, 136.4, 131.9, 131.8, 131.5, 130.2, 129.9, 128.8, 125.0, 123.3, 122.2, 119.7, 118.3, 115.6, 45.6, 30.8, 29.1, 26.6, 25.2, 16.9; **HRMS** (ESI) 450.1848 m/z (M + H^+), calc. for $C_{25}H_{28}N_3O_3S^+$ 450.1846.

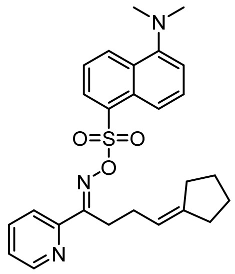

(*E*)-4-cyclopentylidene-1-(pyridin-2-yl)butan-1-one *O*-((5-(dimethylamino)naphthalen-1-yl)sulfonyl) oxime (4l) was synthesized according to general procedure **A**, **C**, **E** and **G**. Grayish green solid; Mp 70.3−71.0 °C; **^{1}H NMR** (600 MHz, $CDCl_3$) δ 8.79 – 8.30 (m, 4H), 7.60 (d, *J* = m, 4H), 7.26 (s, 2H), 5.19 (s, 1H), 3.10 (s, 2H), 2.92 (s, 6H), 2.20 (s, 2H), 2.02 (m, 4H), 1.54 (s, 4H); **^{13}C NMR** (151 MHz, $CDCl_3$) δ 167.4, 151.9, 151.4, 149.2, 145.0, 136.4, 131.9, 131.5, 130.2, 129.9, 128.8, 125.0, 123.2, 122.2, 119.6, 118.1, 115.6, 45.6, 33.5, 28.5, 26.8, 26.5, 26.4, 26.4; **HRMS** (ESI) 464.2000 m/z (M + H^+), calc. for $C_{26}H_{30}N_3O_3S^+$ 464.2002.

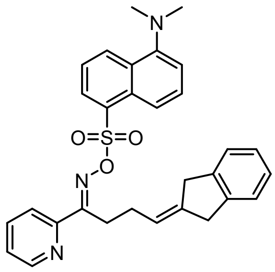

(*E*)-4-(1,3-dihydro-2*H*-inden-2-ylidene)-1-(pyridin-2-yl)butan-1-one *O*-((5-(dimethylami neo)naphthalen-1-yl)sulfonyl) oxime (4m) was synthesized according to general procedure **A**, **C**, **E** and **G**. White solid; Mp 122.0−122.9 °C; **^{1}H NMR** (600 MHz, $CDCl_3$) δ 8.71 (s, 1H), 8.55 (m, 1H), 8.48 (d, *J* = 7.1 Hz, 1H), 8.45 (d, *J* = 7.3 Hz, 1H), 7.62 (t, *J* = 8.1 Hz, 2H), 7.52 (m, 2H), 7.24 (m, 2H), 7.13 (m, 4H), 5.38 (m, 1H), 3.47 (s, 2H), 3.35 (s, 2H), 3.14 (t, *J* = 7.7 Hz, 2H), 2.93 (s, 6H), 2.30 (m, 2H); **^{13}C NMR** (151 MHz, $CDCl_3$) δ 167.4, 151.3 149.2, 142.2, 142.0, 140.3, 136.5, 132.1, 131.8, 131.5, 130.2, 128.8, 126.4, 126.4, 125.1, 124.8, 124.5, 123.7, 122.2, 121.2, 115.9, 45.8, 39.2, 35.7, 26.8, 26.3; **HRMS** (ESI) 512.2000 m/z (M + H^+), calc. for $C_{30}H_{30}N_3O_3S^+$ 512.2002.

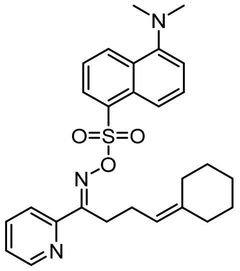

(*E*)-4-cyclohexylidene-1-(pyridin-2-yl)butan-1-one *O*-((5-(dimethylamino)naphthalen-1-yl)sulfonyl) oxime (4n) was synthesized according to general procedure **A**, **C**, **E** and **G**. Kelly solid; Mp 64.3−65.1 °C; **^{1}H NMR** (600 MHz, $CDCl_3$) δ 8.63 (d, *J* = 8.1 Hz, 1H), 8.55 – 8.51 (m, 1H), 8.45 – 8.39 (m, 2H), 7.65 – 7.55 (m, 4H), 7.23 (m, 1H), 7.19 (d, *J* = 7.4 Hz, 1H), 5.01 (t, *J* = 7.4 Hz, 1H), 3.05 (t, *J* = 7.7 Hz, 2H), 2.88 (s, 6H), 2.20 (m, 2H), 1.93 – 1.85 (m, 4H), 1.44 (m, 2H), 1.33 (m, 4H); **^{13}C NMR** (151 MHz, $CDCl_3$) δ 167.3, 151.4, 149.1, 141.4, 136.4, 131.9, 131.5, 130.2, 129.9, 128.8, 125.0, 123.3, 122.2, 119.7, 119.3, 115.7, 45.6, 37.0, 28.6, 28.5, 27.8, 26.9, 26.9, 24.4; **HRMS** (ESI) 478.2157 m/z (M + H^+), calc. for $C_{27}H_{32}N_3O_3S^+$ 478.2159.

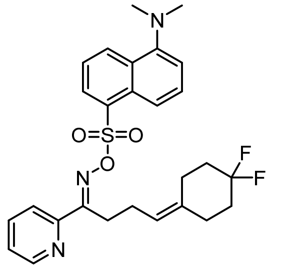

(*E*)-4-(4,4-difluorocyclohexylidene)-1-(pyridin-2-yl)butan-1-one *O*-((5-(dimethylamino) naphthalen-1-yl)sulfonyl) oxime (4o) was synthesized according to general procedure **A**, **C**, **E** and **G**. Kelly solid; Mp 103.9−104.8 °C; **^{1}H NMR** (600 MHz, $CDCl_3$) δ 8.66 (d, *J* = 5.8 Hz, 1H), 8.53 (m, 1H), 8.44 (m, 1H), 8.42 (d, *J* = 8.6 Hz, 1H), 7.63 – 7.58 (m, 4H), 7.26 – 7.24 (m, 1H), 7.21 (d, *J* = 6.8 Hz, 1H), 5.18 (t, *J* = 7.5 Hz, 1H), 3.09 (t, *J* = 7.6 Hz, 2H), 2.90 (s, 6H), 2.27 (m, 2H), 2.05 – 2.01 (m, 2H), 2.01 – 1.97 (m, 2H), 1.69 (m, 2H), 1.61 (m, 2H); **^{13}C NMR** (151 MHz, $CDCl_3$) δ 166.8, 151.2, 149.2, 136.5, 136.0, 132.1, 131.9, 131.4, 130.2, 128.8, 125.2, 123.4 (dd, J=249.1, 232.2 Hz), 122.8, 121.9, 115.7, 45.6, 34.9 (t, *J* = 23.2 Hz), 34.3 (t, *J* = 23.7 Hz), 32.0 (t, *J* = 5.0 Hz), 26.4, 24.8, 23.6 (t, *J* = 5.2 Hz); **^{19}F NMR** (376 MHz, $CDCl_3$) δ -97.32; **HRMS** (ESI) 514.1972 m/z (M + H^+), calc. for $C_{27}H_{30}F_2N_3O_3S^+$ 514.1970.

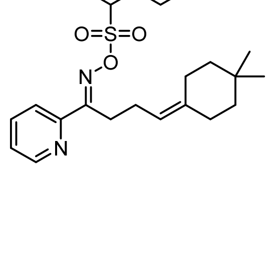

(*E*)-4-(4,4-dimethylcyclohexylidene)-1-(pyridin-2-yl)butan-1-one *O*-((5-(dimethylamino) naphthalen-1-yl)sulfonyl) oxime (4p) was synthesized according to general procedure **A**, **C**, **E** and **G**. Kelly solid; Mp 118.8−119.7 ºC; **^{1}H NMR** (600 MHz, $CDCl_3$) δ 8.62 (d, *J* = 8.3 Hz, 1H), 8.53 (d, *J* = 4.7 Hz, 1H), 8.42 (m, 2H), 7.64 – 7.51 (m, 4H), 7.32 – 7.09 (m, 2H), 5.02 (t, *J* = 7.3 Hz, 1H), 3.05 (t, *J* = 7.7 Hz, 2H), 2.88 (s, 6H), 2.20 (m, 2H), 1.93 – 1.85 (m, 4H), 1.17 – 1.04 (m, 4H), 0.85 (s, 6H); **^{13}C NMR** (151 MHz, $CDCl_3$) δ 167.3, 151.4, 149.1, 141.1, 136.4, 131.9, 131.9, 131.5, 130.2, 129.9, 128.8, 125.0, 123.3, 122.2, 119.6, 119.3, 115.6, 45.6, 40.8, 40.5, 32.7, 30.6, 28.3, 26.9, 24.5, 24.3; **HRMS** (ESI) 506.2467 m/z (M + H^+), calc. for $C_{29}H_{36}N_3O_3S^+$ 506.2472.

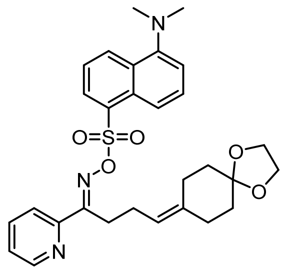

(*E*)-1-(pyridin-2-yl)-4-(1,4-dioxaspiro[4.5]decan-8-ylidene)butan-1-one *O*-((5-(dimethyllll amino)naphthalen-1-yl)sulfonyl) oxime (4q) was synthesized according to general procedure **B**, **C**, **E** and **G**. Kelly solid; Mp 116.6−117.5 ºC; **^{1}H NMR** (400 MHz, $CDCl_3$) δ 8.74 (d, *J* = 8.2 Hz, 1H), 8.63 (d, *J* = 4.2 Hz, 1H), 8.52 (m, 2H), 7.69 (m, 4H), 7.40 – 7.25 (m, 2H), 5.19 (t, *J* = 7.1 Hz, 1H), 4.03 (s, 4H), 3.18 (m, 2H), 2.99 (s, 6H), 2.34 (m, 2H), 2.13 (s, 4H), 1.63 – 1.47 (m, 4H); **^{13}C NMR** (101 MHz, $CDCl_3$) δ 167.1, 151.3, 149.1 138.5, 136.5, 131.95, 131.9, 131.4, 130.2, 129.9, 128.8, 125.1, 123.3, 122.2, 120.9, 119.6, 115.7, 109.0, 64.4, 45.7, 36.1, 35.3, 33.4, 26.7, 24.9, 24.8; **HRMS** (ESI) 536.2213 m/z (M + H^+), calc. for $C_{29}H_{34}N_3O_5S^+$ 536.2214.

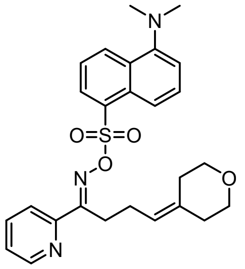

(*E*)-1-(pyridin-2-yl)-4-(tetrahydro-4*H*-pyran-4-ylidene)butan-1-one *O*-((5-(dimethylami neo)naphthalen-1-yl)sulfonyl) oxime (4r) was synthesized according to general procedure **B**, **C**, **E** and **G**. Yellow solid; Mp 74.4−75.1 ºC; **^{1}H NMR** (600 MHz, $CDCl_3$) δ 8.62 (d, *J* = 8.4

Hz, 1H), 8.53 (d, J = 4.5 Hz, 1H), 8.43 (d, J = 7.2 Hz, 1H), 8.39 (d, J = 8.6 Hz, 1H), 7.65 – 7.53 (m, 4H), 7.25 – 7.21 (m, 1H), 7.19 (d, J = 7.5 Hz, 1H), 5.12 (t, J = 7.4 Hz, 1H), 3.44 (t, J = 5.4 Hz, 2H), 3.36 (t, J = 5.4 Hz, 2H), 3.08 (t, J = 7.5 Hz, 2H), 2.88 (s, 6H), 2.25 (m, 2H), 1.96 (m, 4H); **^{13}C NMR** (151 MHz, $CDCl_3$) δ 166.9, 151.3, 149.2, 136.5, 135.9, 131.9, 131.4, 130.2, 129.9, 129.9, 128.8, 125.1, 123.3, 122.1, 121.3, 119.5, 115.6, 45.6, 36.8, 29.5, 26.6, 24.2; **HRMS** (ESI) 480.1956 m/z (M + H^+), calc. for $C_{26}H_{30}N_3O_4S^+$ 480.1952.

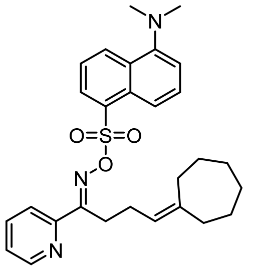

(*E*)-4-cycloheptylidene-1-(pyridin-2-yl)butan-1-one *O*-((5-(dimethylamino)naphthalen-1-yl)sulfonyl) oxime (4s) was synthesized according to general procedure **A**, **C**, **E** and **G**. Kelly solid; Mp 88.4−89.2 ºC; **^{1}H NMR** (600 MHz, $CDCl_3$) δ 8.63 (d, J = 8.3 Hz, 1H), 8.53 (d, J = 4.7 Hz, 1H), 8.47 – 8.31 (m, 2H), 7.63 – 7.51 (m, 4H), 7.24 (dd, J = 8.8, 4.6 Hz, 1H), 7.19 (d, J = 7.5 Hz, 1H), 5.07 (t, J = 7.1 Hz, 1H), 3.09 – 3.01 (m, 2H), 2.88 (s, 6H), 2.21 – 2.15 (m, 2H), 2.03 (m, 4H), 1.40 (s, 8H); **^{13}C NMR** (151 MHz, $CDCl_3$) δ 167.4, 151.4, 149.2, 144.5, 136.4, 131.9, 131.9, 131.9, 130.2, 129.9, 128.8, 125.0, 123.3, 122.2, 120.7, 119.6, 115.6, 45.6, 29.1, 26.9, 24.9, 23.2, 13.3, 12.8; **HRMS** (ESI) 492.2314 m/z (M + H^+), calc. for $C_{28}H_{34}N_3O_3S^+$ 492.2315.

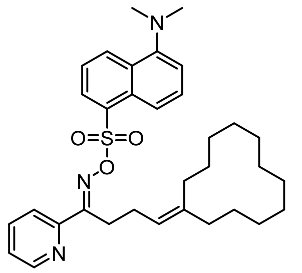

(*E*)-4-cyclododecylidene-1-(pyridin-2-yl)butan-1-one *O*-((5-(dimethylamino)naphthalen-1-yl)sulfonyl) oxime (4t) was synthesized according to general procedure **A**, **C**, **E** and **G**. White solid; Mp 81.9−82.8 ºC; **^{1}H NMR** (400 MHz, $CDCl_3$) δ 8.62 (d, J = 8.5 Hz, 1H), 8.53 (m, 1H), 8.42 (m, 2H), 7.58 (m, 4H), 7.24 (m, 1H), 7.19 (d, J = 7.6 Hz, 1H), 5.17 (t, J = 7.2 Hz, 1H), 3.09 – 2.97 (m, 2H), 2.88 (s, 6H), 2.24 (m, 2H), 1.91 (t, J = 6.6 Hz, 4H), 1.41 – 1.18 (m, 18H);

^{13}C NMR (101 MHz, $CDCl_3$) δ 167.4, 151.4, 149.2, 139.2, 136.4, 131.9, 131.5, 130.2, 129.9, 128.8, 125.0, 123.2, 123.2, 122.2, 119.6, 115.6, 45.6, 31.6, 28.6, 26.9, 25.3, 25.2, 24.9, 24.2, 24.2, 24.0, 23.9, 23.5, 23.3, 22.4; **HRMS** (ESI) 584.2920 m/z (M + Na^+), calc. for $C_{33}H_{43}N_3NaO_3S^+$ 584.2917.

3.2 Synthesis of vinyl azides

Vinyl azides were synthesized following established literature procedures. To a Schlenk flask equipped with a stir bar, terminal alkyne (3.0 mmol, 1.0 equiv.), $TMSN_3$ (6.0 mmol, 2.0 equiv.), and H_2O (6.0 mmol, 2.0 equiv.) were added to 3 mL of dimethyl sulfoxide (DMSO) and heated to 80 °C. Subsequently, Ag_2CO_3 (10 mol%) was introduced into the mixture. After stirring, the reaction was allowed to cool to room temperature, then diluted with water and extracted with ethyl acetate three times. The combined organic layers were washed with brine, dried over anhydrous Na_2SO_4, filtered, and concentrated under reduced pressure. The crude product was further purified by flash column chromatography on silica gel using a petroleum ether/ethyl acetate eluent, yielding the desired vinyl azide.

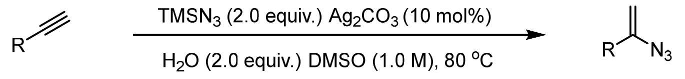

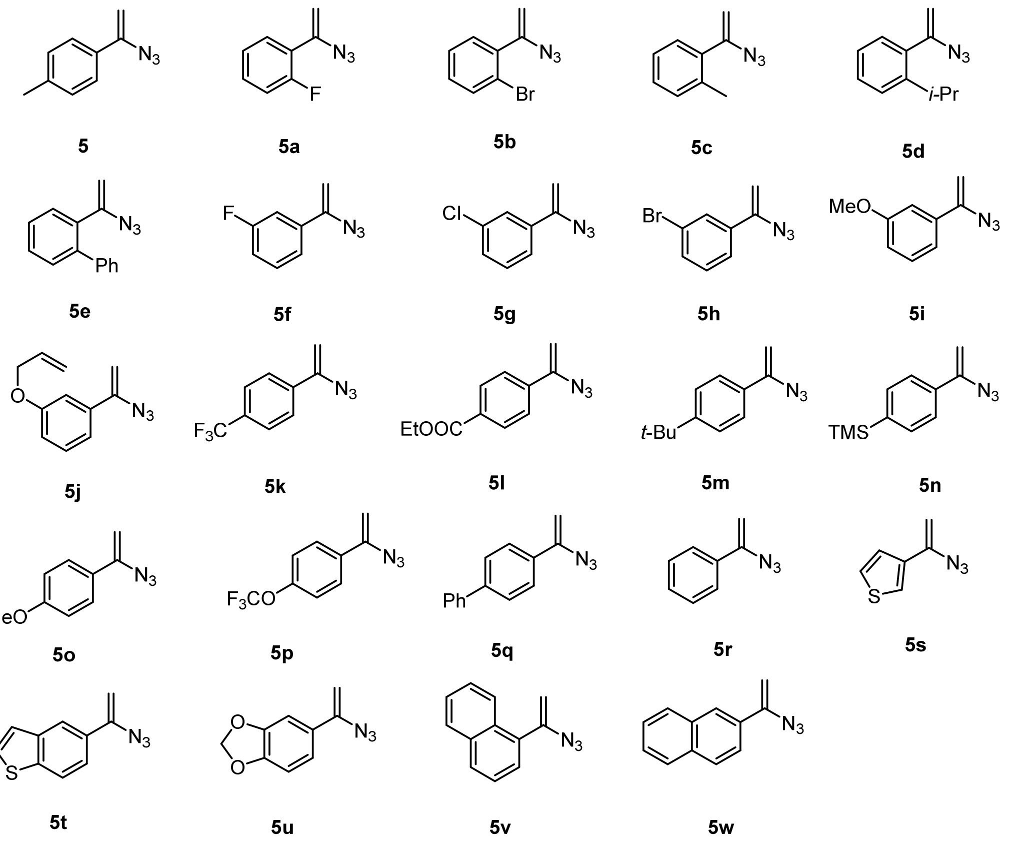

The experimental data are in accordance with the literature reports: **5**[8], **5b**[8], **5k-5r**[8], **5v**[8], **5t**[8], **5v**[8], **5a**[9], **5d**[9], **5f-5j**[9], **5s**[9], **5u**[9], **5c**[10], **5e**[11]

3.3 General experimental procedures for accessing enantioenriched products

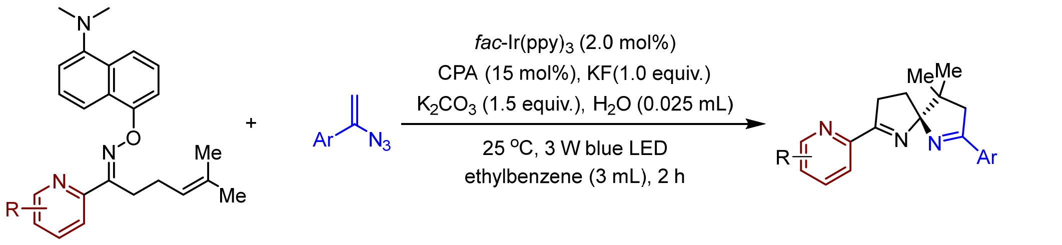

An oven-dried Schlenk tube (10 mL) equipped with a stirring bar was charged with olefinic sulfonyl oximes (0.1 mmol), **C**1 (0.015 mmol, 15 mol%), K_2CO_3 (0.015 mmol, 1.5 equiv.), KF (0.1 mmol, 1.0 equiv.), H_2O (0.025 mL), a photocatalyst (2.0 mol%), vinyl azide (0.3 mmol, 3.0 equiv.), and ethylbenzene (3.0 mL). The reaction mixture was degassed three times using the freeze-pump-thaw method. The tube was then positioned approximately 2.0 cm from 3 W blue LEDs and stirred at room temperature for 2 hours. Upon completion, the residue was purified by flash chromatography on silica gel using a gradient elution of petroleum ether/ethyl acetate/tetrahydrofuran (15:1:1 to 10:1:1) to yield the desired product.

3.4 Application of *N*, *N*, *N*-ligand[12]

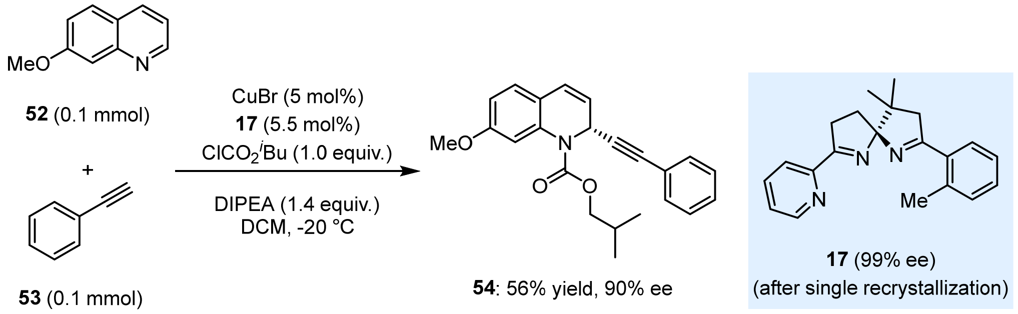

CuBr (0.005 mmol, 5 mol%) was added to a flask inside a glove box, which was subsequently fitted with a septum before being removed and placed under a dry nitrogen atmosphere. To this flask, a solution of compound **17** (0.0055 mmol, 5.5 mol%) in dichloromethane (1.0 mL) was added, and the mixture was stirred for 30 minutes at room temperature. Following this, the mixture was cooled to 0 °C, compound **53** (0.1 mmol, 1.0 equiv.) was introduced into the flask. During the stirring period, isobutyl chloroformate (0.1 mmol, 1.0 equiv.) was added to a solution of 7-methoxyquinoline (0.1 mmol, 1.0 equiv.) in DCM (1.0 mL) and stirred for 5 minutes at room temperature. The resulting quinolinium salt was then transferred to the flask

containing the copper catalyst and alkyne, followed by the addition of DIPEA (0.14 mmol, 1.4 equiv.) via syringe, the mixture was cooled to -20°C. The reaction mixture was stirred for the appropriate duration, as monitored by TLC. Upon completion, the reaction mixture was subjected directly to flash column chromatography on silica gel, using an eluent of ethyl acetate/petroleum ether (10:1), to yield the desired product **54**.

4. Experimental equipment

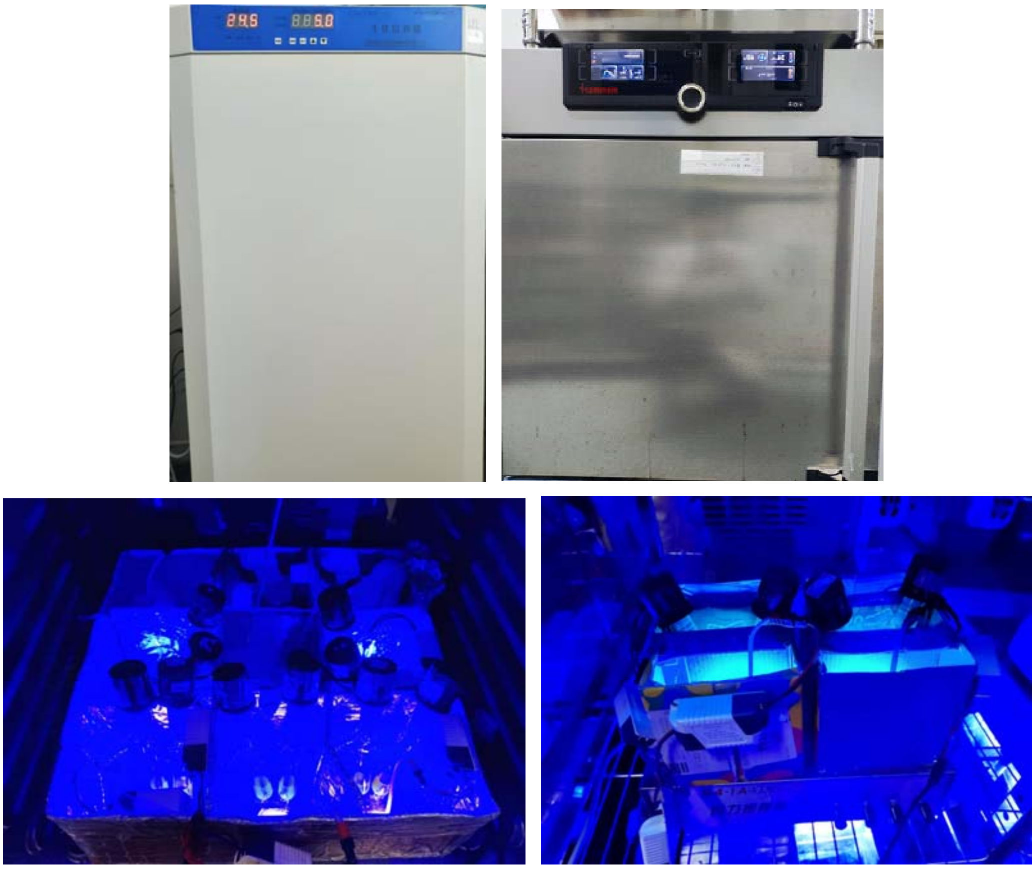

5. Mechanism studies

5.1 UV/Visible absorption spectroscopy

UV-vis absorption spectroscopy was performed on a PERSEE TU-1901 spectrophotometer, equipped with a temperature control unit at 25 °C. The samples were measured in a 3 mL quartz cuvette fitted with a PTFE stopper. **4**, **5**, **C1 and *fac*-Ir(ppy)₃** were prepared as a 0.1 mM solution with fresh DCM as the solvent for measurement.

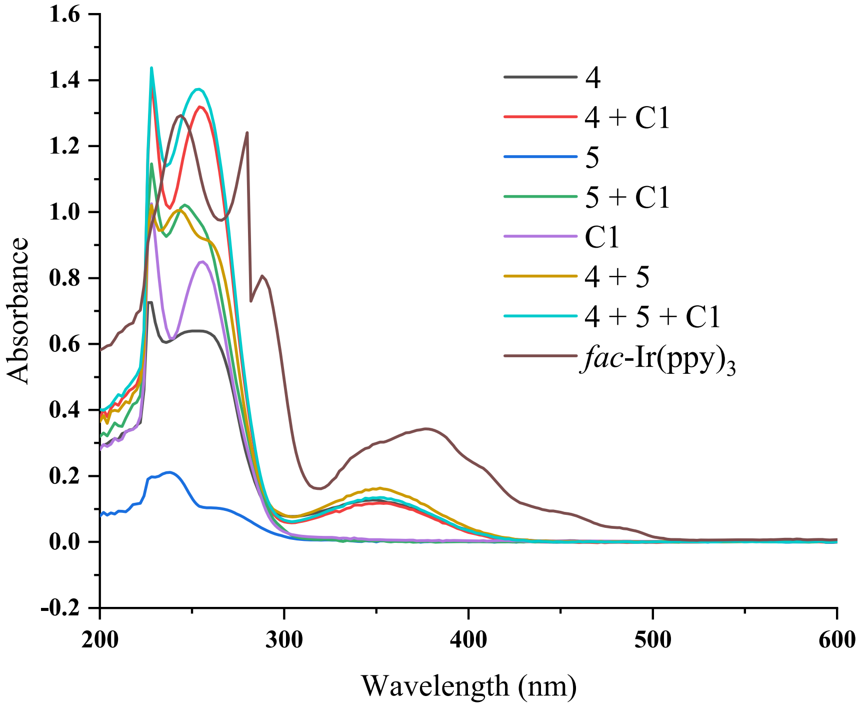

Fig. S1. UV-vis absorption spectra of **4**, **5**, **C1, 4 + C1**, **5 + C1**, **4 + 5**, **4 + 5 + C1 and *fac*-Ir(ppy)$_3$**.

Comments: The results can rule out the production of new photo-sensitive species, such as EDA complexes, in the reaction system.

5.2 Cyclic voltammetry studies

Electrochemical measurements were conducted using a CHI 660E electrochemical analyzer (CH Instruments). Electrochemical potentials were determined under standardized conditions to maintain internal consistency. Cyclic voltammograms were collected with a potentiostat. Samples, consisting of 0.01 mmol of compounds **4**, **5**, **4** + **C1**, and **5** + **C1**, were prepared in 10 mL of 0.1 M tetrabutylammonium hexafluorophosphate dissolved in anhydrous acetonitrile. The measurements utilized a radially configured glassy carbon working electrode, a platinum wire counter electrode, and a saturated KCl silver/silver chloride reference electrode, with all values referenced to Ag/AgCl.

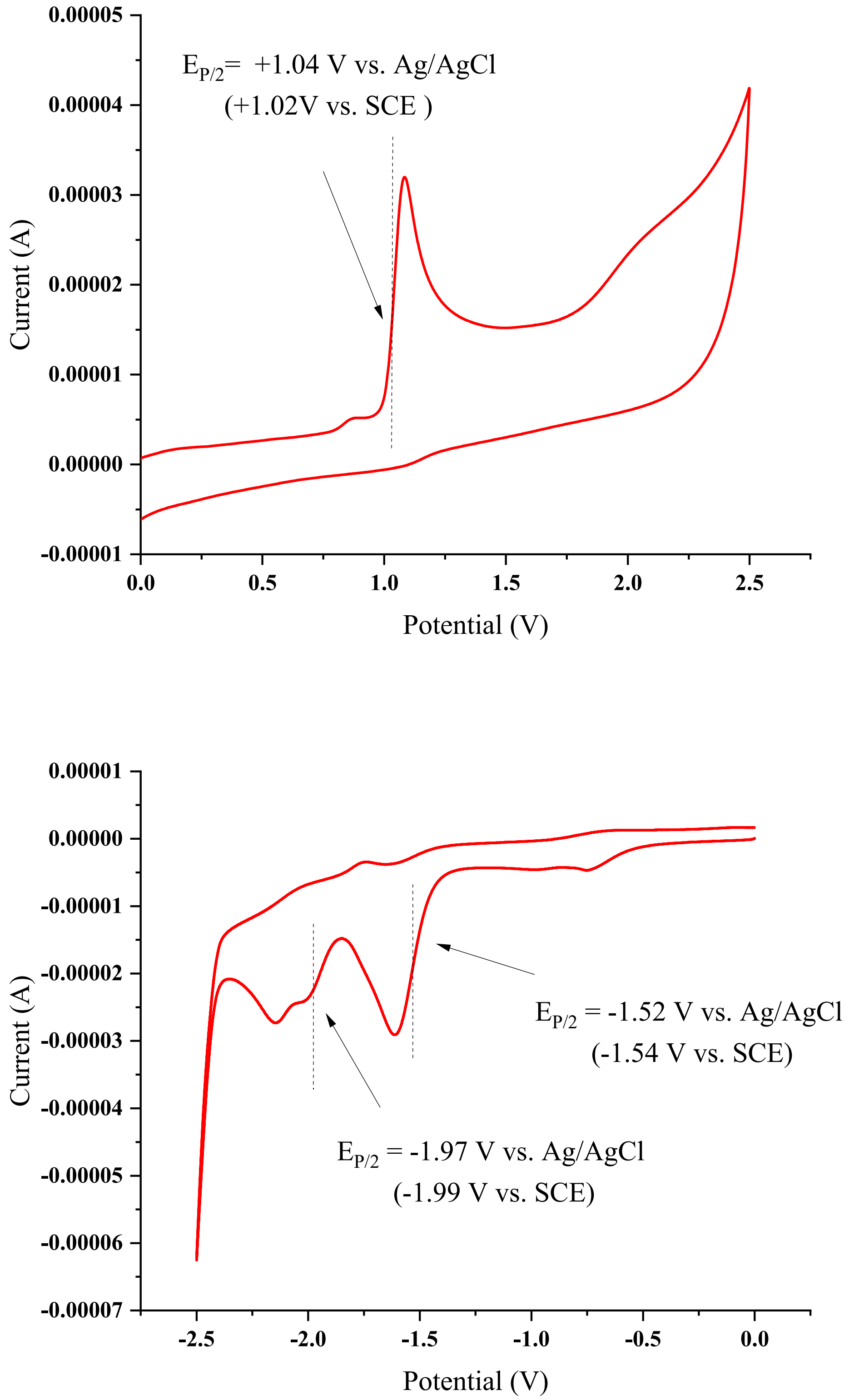

Fig. S2 Cyclic voltammogram of **4** in MeCN.

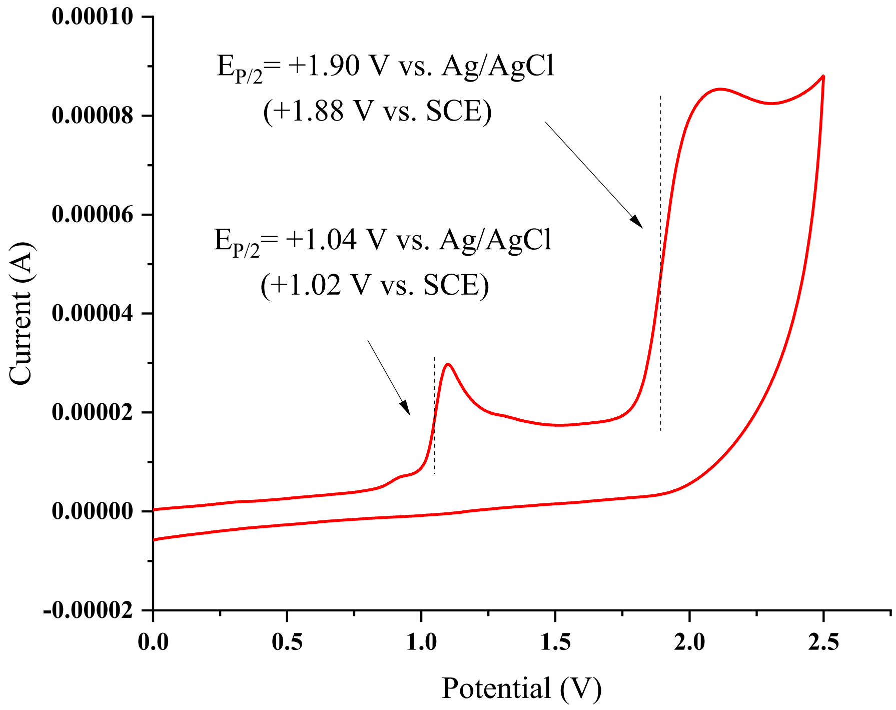

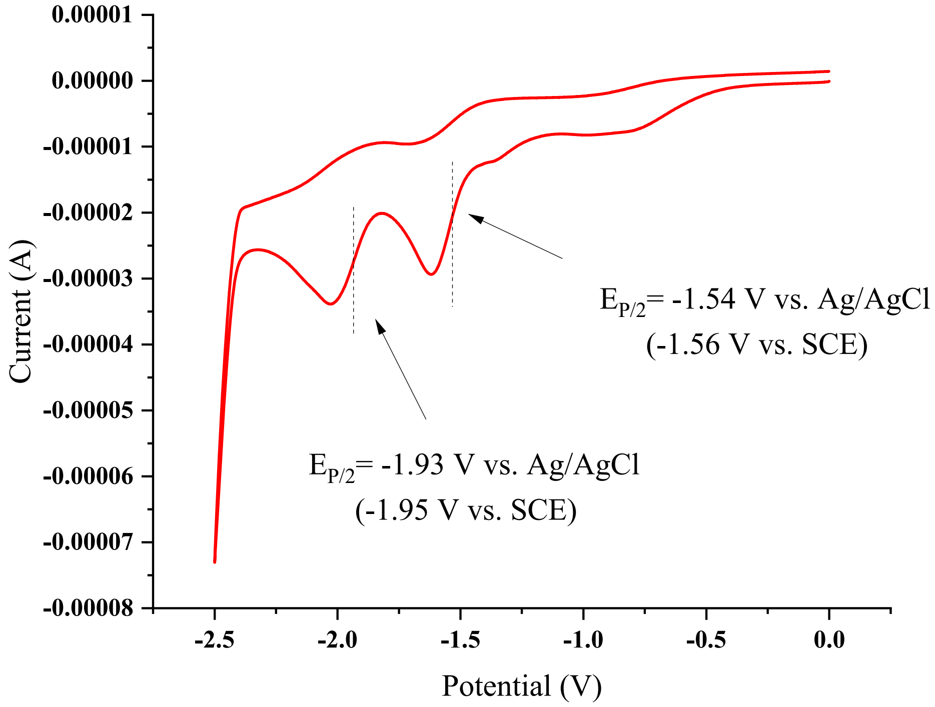

Fig. S3 Cyclic voltammogram of **4** + **C1** in MeCN.

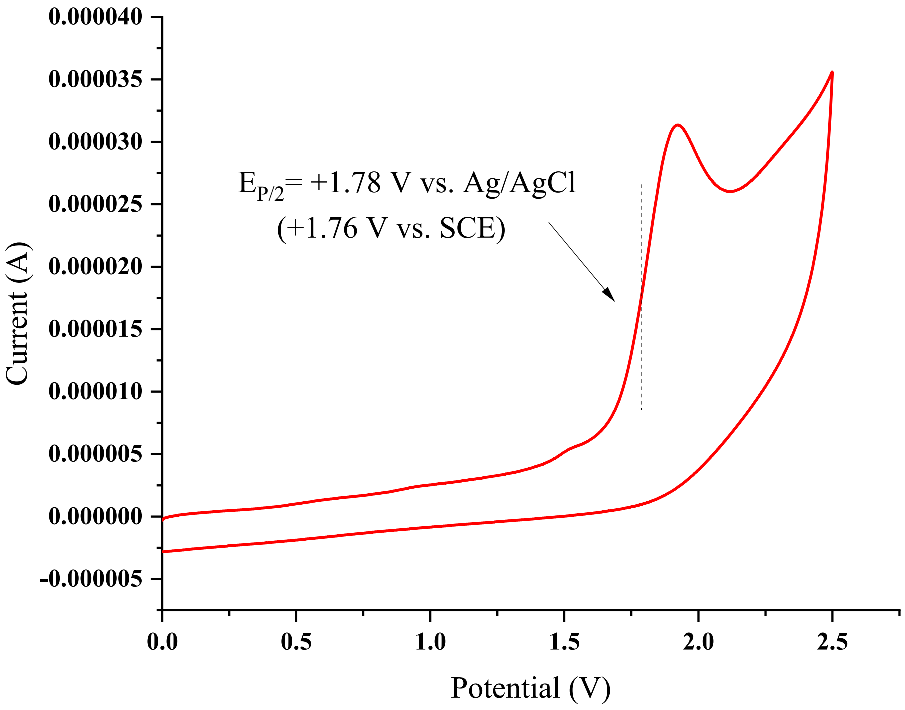

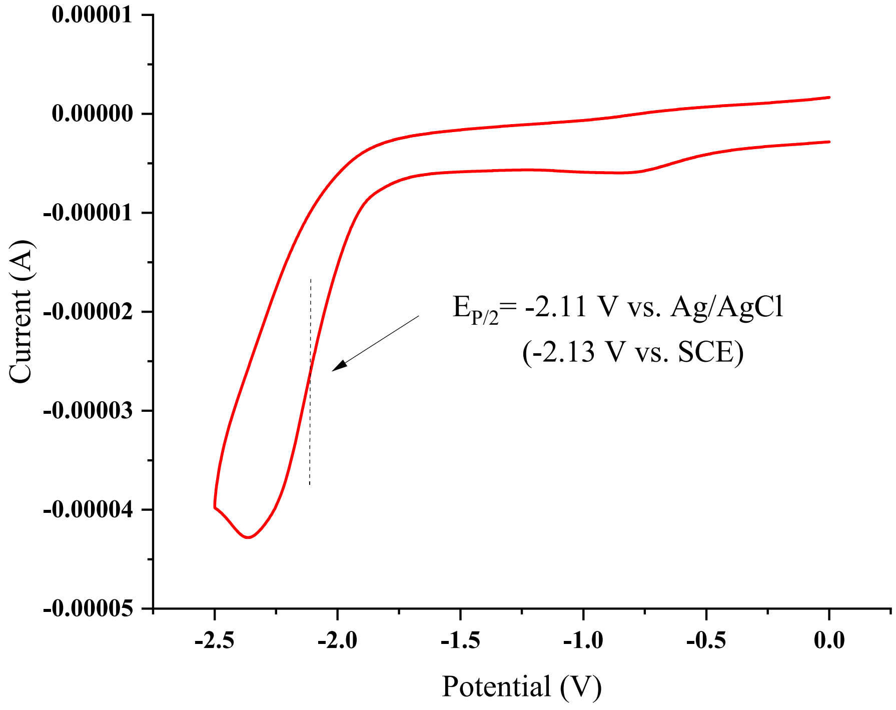

Fig. S4 Cyclic voltammogram of **5** in MeCN.

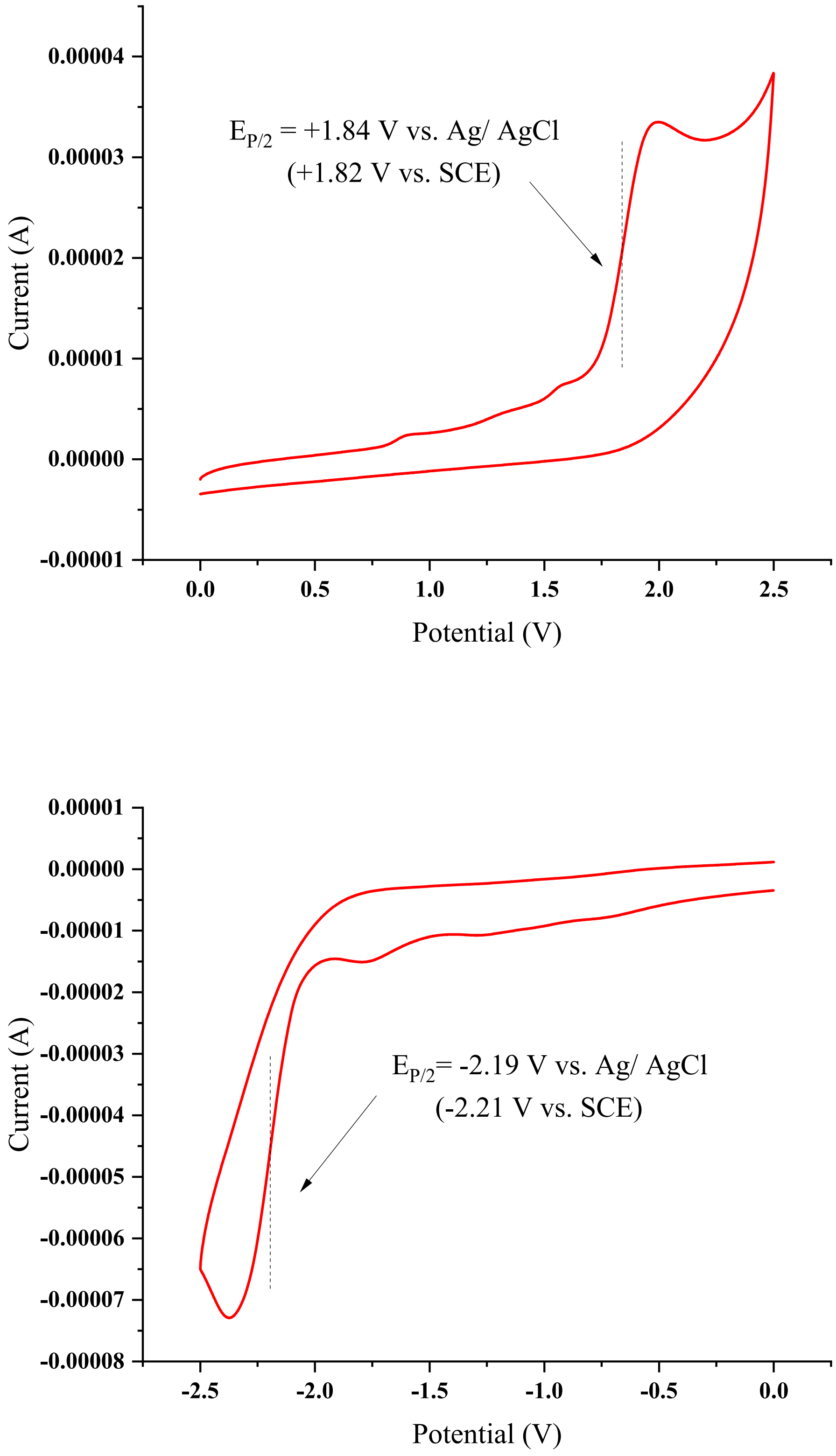

Fig. S5 Cyclic voltammogram of **5** + **C1** in MeCN.

5.3 Phosphorescence emission spectra at 77K of 4

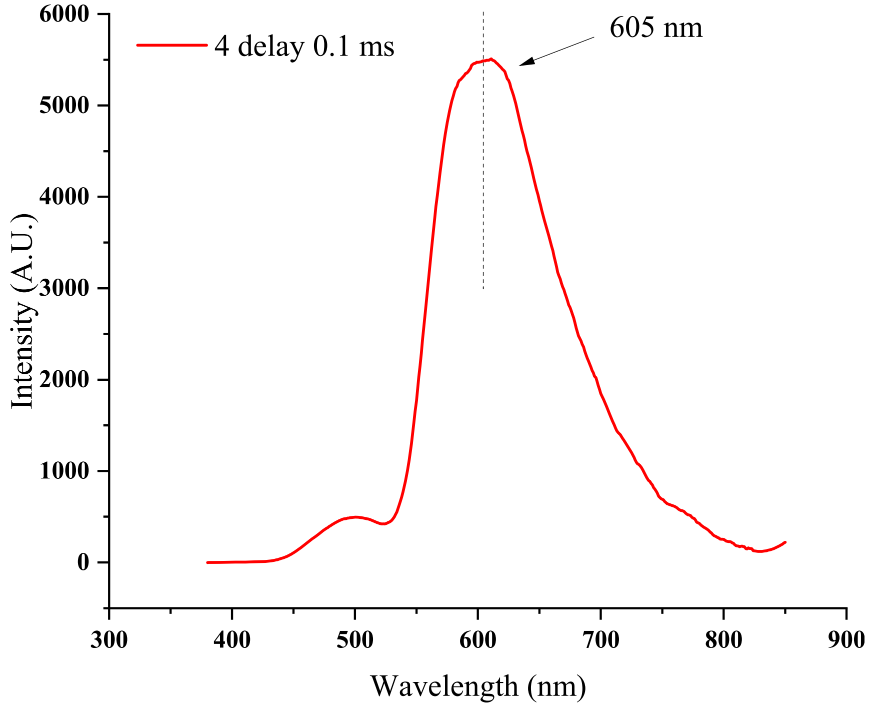

Fig. S6 The phosphorescence emission data for **4** with concentration of 1.0 mM in a DCM matrix at 77 K. The maxima was obtained at 605 nm. E_T = 47.3 kcal/mol.

5.4 Fluorescence emission spectra of 4 and *fac*-Ir(ppy)₃

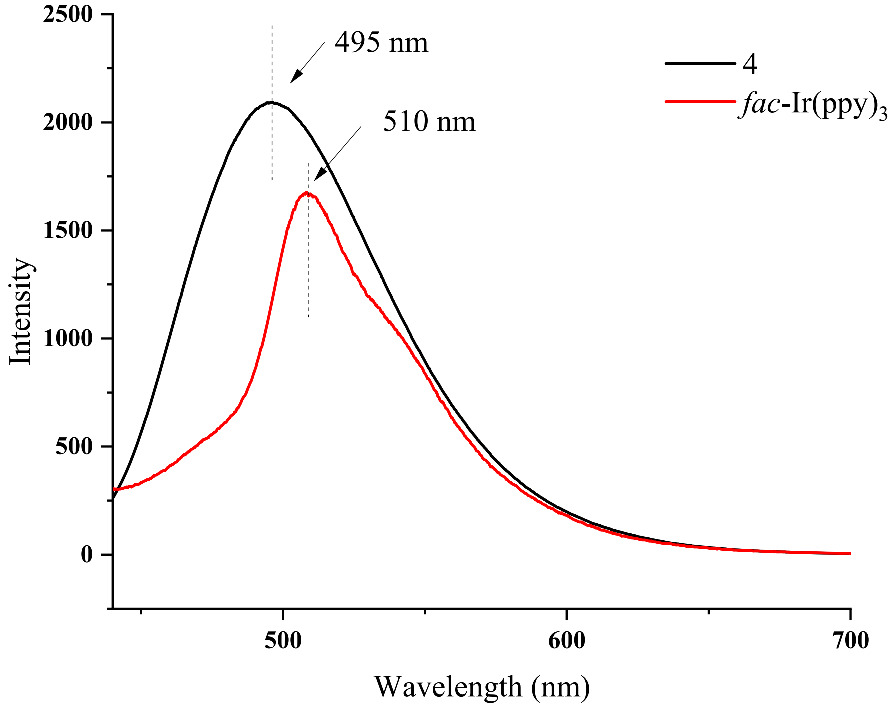

Fig. S7 The fluorescence emission spectrum for **4** and *fac*-Ir(ppy)$_3$ with concentration of 1.0 mM in a DCM, the maxima was obtained at 495 nm and 510 nm, respectively.

5.5 Stern-Volmer fluorescence quenching experiments and grating experiment

Emission intensities were recorded on a spectrofluorometer, *fac*-Ir(ppy)$_3$ solution was excited at 370 nm and the emission intensity at 510 nm was observed. A solution of *fac*-Ir(ppy)$_3$ (1.0 × 10^{-5} M) in EtPh was added to the appropriate amount of quencher in 3.0 mL volumetric flask under N_2. The solution was transferred to a 3.0 mL quartz cell and the emission spectrum of the sample was collected.

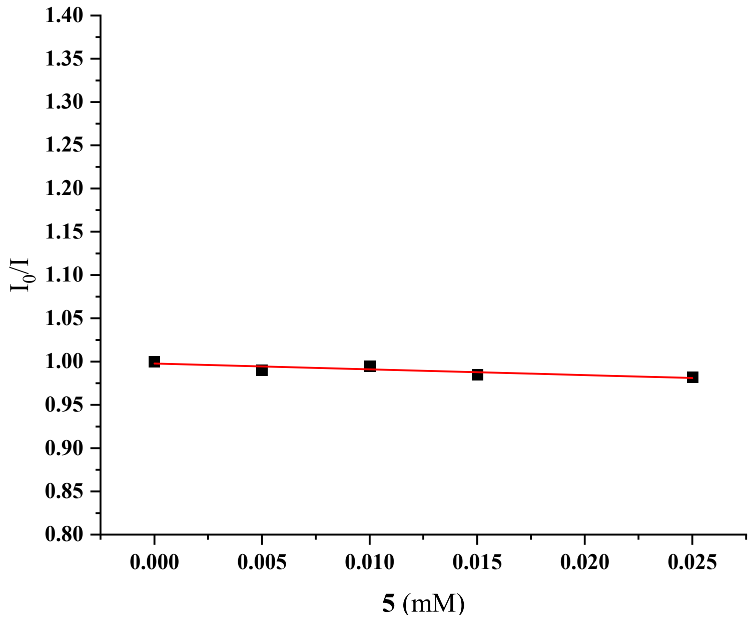

Fig. S8 Luminescence quenching experminent: Stern-Volmer plots of the photosensitizer *fac*-Ir(ppy)$_3$ using varying concentrations of **5** in EtPh.

5.6 Linear effect experiments

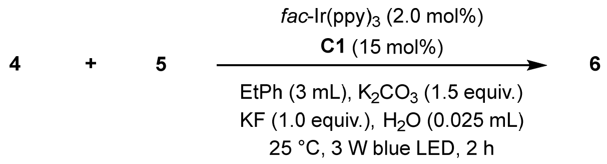

The ee value of **C1** was determined by HPLC analysis: CHIRALPAK QD-AX (4.6 mm i.d. x 150 mm); methanol/acetonitrile/acetic acid/triethylamine = 50/50/2/0.2; flow 0.5 mL/min; 25 °C; 254 nm. The ee value of **6** was determined by HPLC analysis: CHIRALPAK IE (4.6 mm i.d. x 250 nm); hexane/2-propanol = 80/20; flow rate 1.0 mL/min; 20 °C, 254 nm.

entry	1	2	3	4	5	6
ee (%) of **C1**	3	27	45	66	81	99
ee (%) of **6**	0	18	40	60	72	90

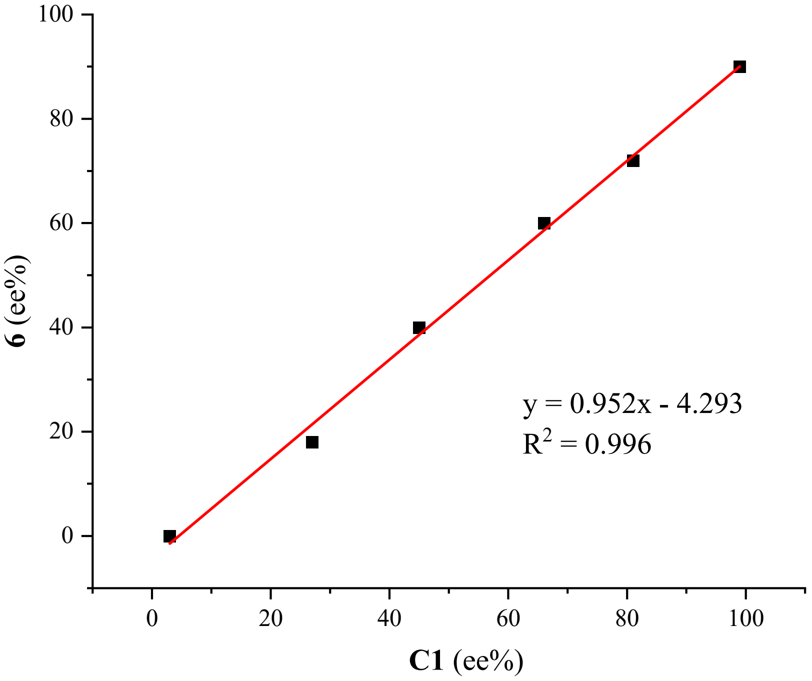

Fig. S9 Relationship between ee values of **C1** and **6.**

HPLC spectra:

C1: 3% ee

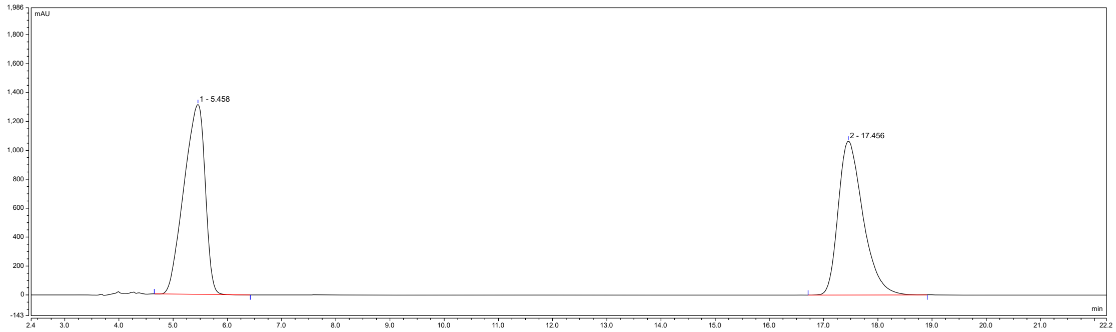

Entry	Retention Time	Area	Height	%Area
1	5.458	594.8593	1313.39	51.36
2	17.456	563.3493	1064.01	48.64

C1: 27% ee

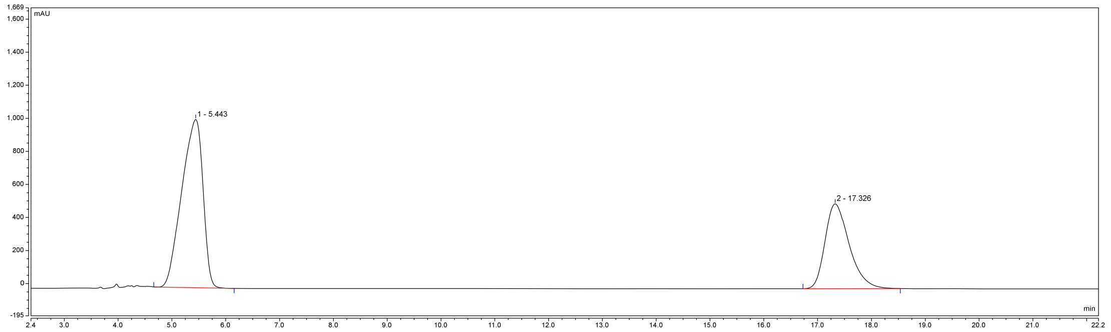

Entry	Retention Time	Area	Height	%Area
1	5.443	465.1247	1017.41	63.51
2	17.326	267.2776	512.38	36.49

C1：45% ee

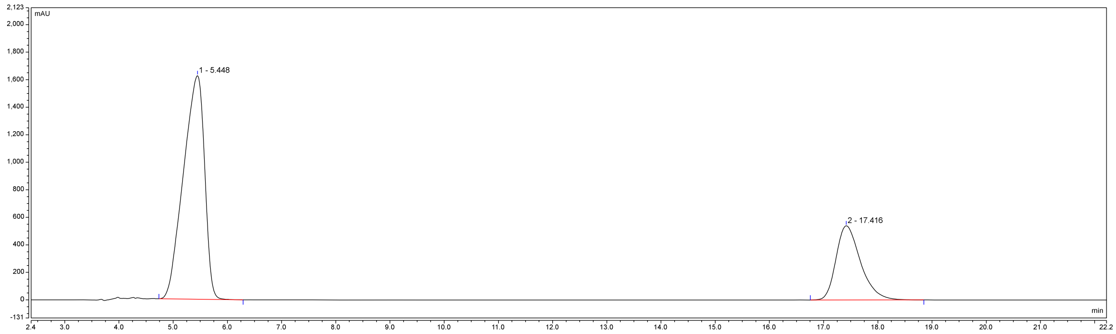

Entry	Retention Time	Area	Height	%Area
1	5.448	734.9608	1624.47	72.28
2	17.416	281.8033	538.59	27.72

C1：66% ee

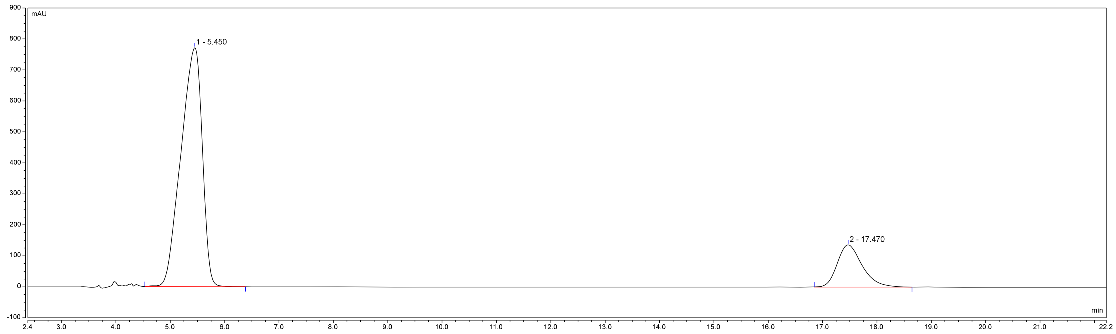

Entry	Retention Time	Area	Height	%Area
1	5.450	350.5317	771.17	82.96
2	17.470	72.0007	135.99	17.04

C1: 81%

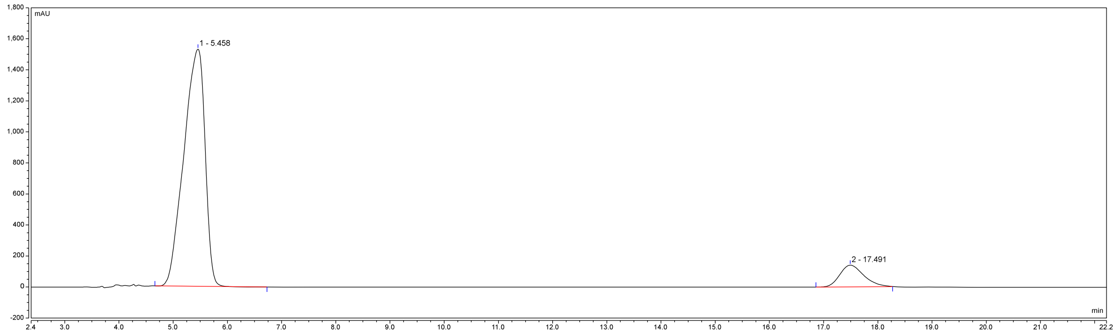

Entry	Retention Time	Area	Height	%Area
1	5.458	691.4495	1528.72	90.58
2	17.491	71.8996	140.24	9.42

C1: 99% ee

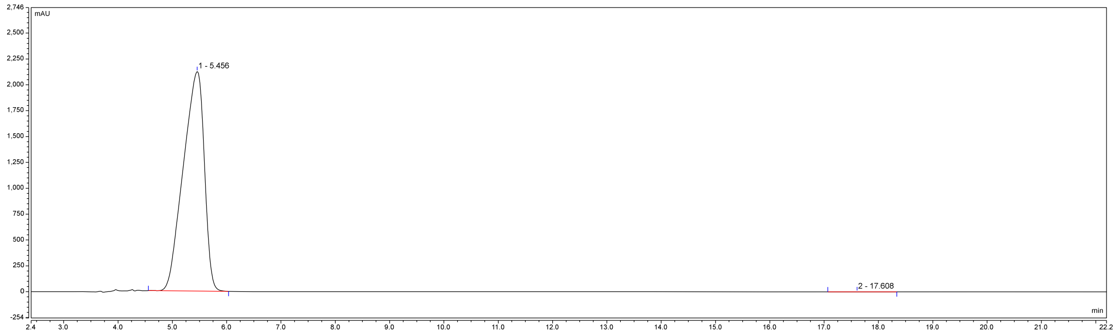

Entry	Retention Time	Area	Height	%Area
1	5.456	950.4685	2122.53	99.99
2	17.608	0.1202	0.25	0.01

6a: 0% ee

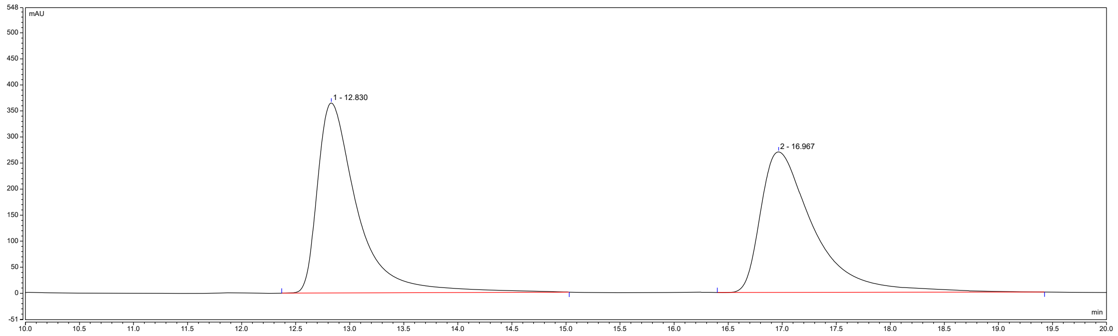

Entry	Retention Time	Area	Height	%Area
1	12.830	156.0018	364.68	50.37
2	16.967	153.7055	269.96	49.63

6a: 18% ee

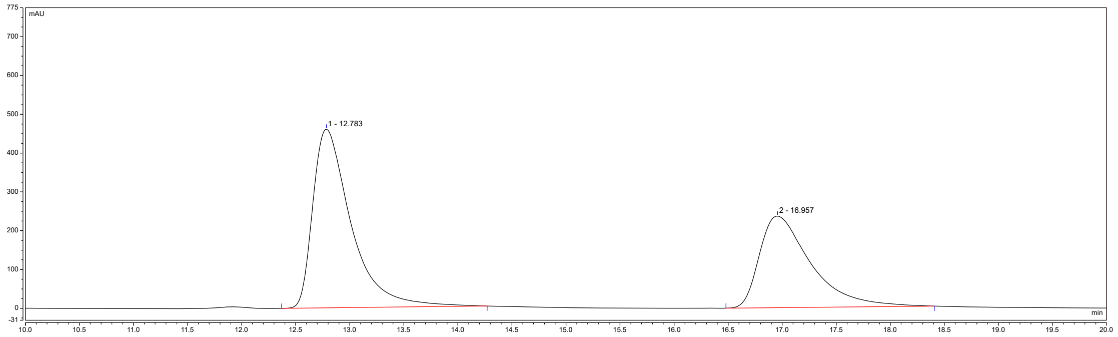

Entry	Retention Time	Area	Height	%Area
1	12.783	188.6594	460.27	59.21
2	16.957	129.9564	236.20	40.79

6a: 40% ee

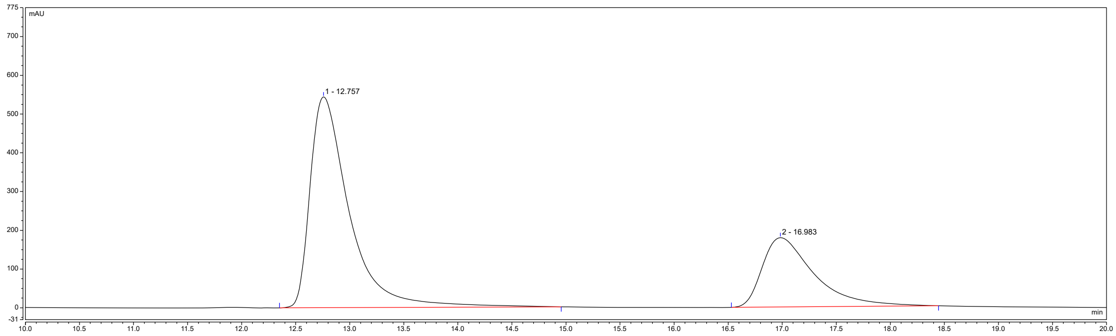

Entry	Retention Time	Area	Height	%Area
1	12.757	227.9025	543.68	69.65
2	16.983	99.3121	179.03	30.35

6a: 60% ee

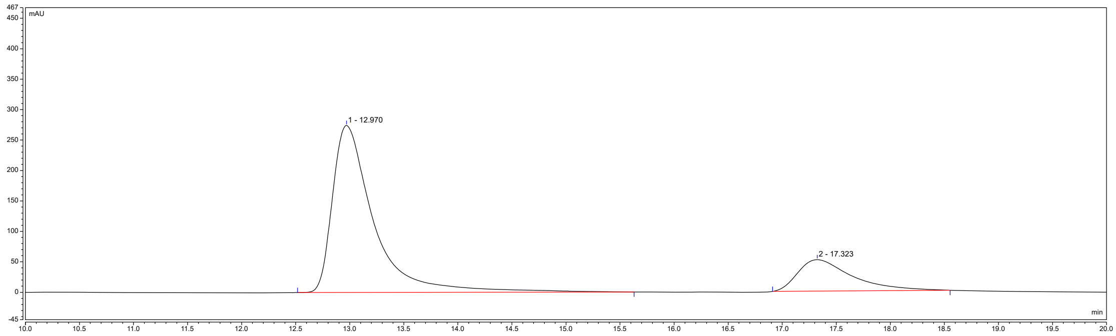

Entry	Retention Time	Area	Height	%Area
1	12.970	121.1727	274.29	80.22
2	17.323	29.8721	51.67	19.78

6a: 72% ee

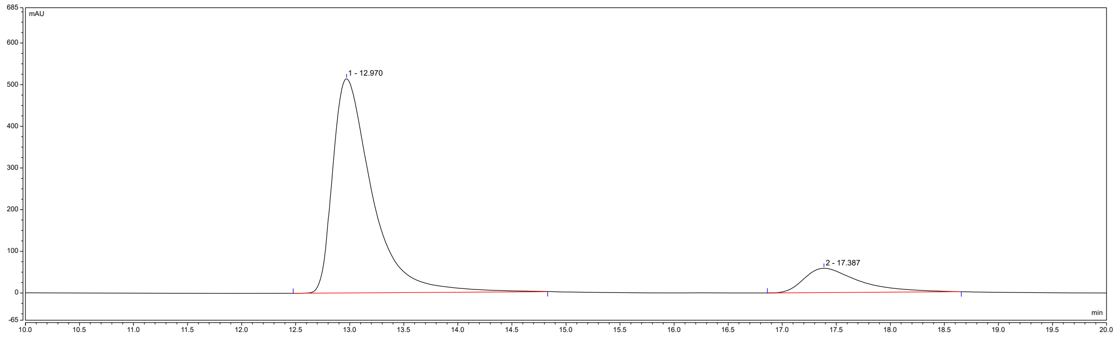

Entry	Retention Time	Area	Height	%Area
1	12.970	216.3354	514.25	86.25
2	17.387	34.4827	58.49	13.75

6a: 90% ee

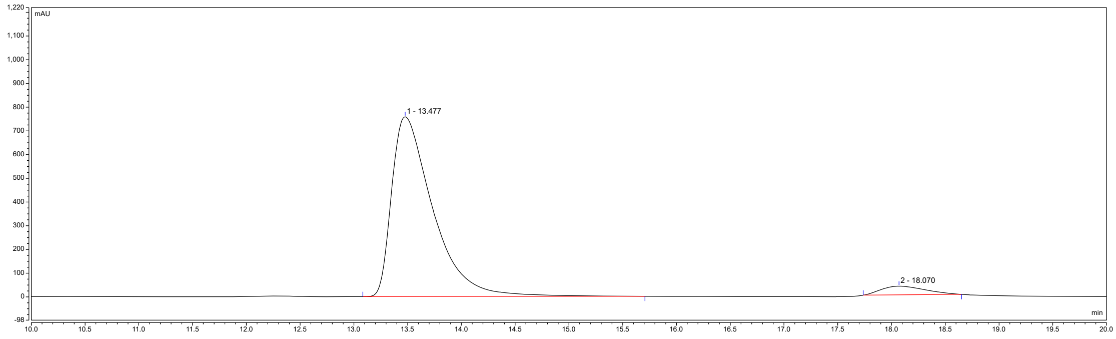

Entry	Retention Time	Area	Height	%Area
1	13.477	336.3840	757.81	95.07
2	18.070	17.4559	36.80	4.93

6. Determination of the absolute configurations

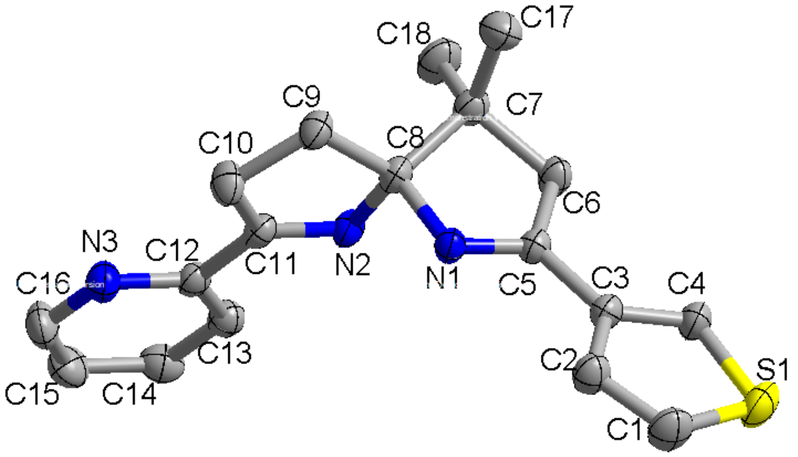

Fig. S10 X-ray **s**ingle-crystal structure of **29** (CCDC: 2414232).

Table S2 Crystal data and structure refinement for 29.

Identification code	**29**
Empirical formula	$C_{18}H_{19}N_3S$
Formula weight	309.42
Temperature/K	293(2)
Crystal system	monoclinic
Space group	C2
a/Å	20.1236(5)
b/Å	7.0187(3)
c/Å	12.0117(4)
α/°	90
β/°	97.820(3)
γ/°	90
Volume/Å^3	1680.77(9)
Z	4
ρ_{calc}g/cm^3	1.223
μ/mm^{-1}	1.694
F(000)	656.0
Crystal size/mm^3	0.21 × 0.11 × 0.1
Radiation	CuKα (λ = 1.54184)
2Θ range for data collection/°	7.428 to 141.706
Index ranges	$-24 \leq h \leq 24$, $-8 \leq k \leq 6$, $-14 \leq l \leq 14$
Reflections collected	11325
Independent reflections	2804 [R_{int} = 0.0345, R_{sigma} = 0.0285]
Data/restraints/parameters	2804/1/201
Goodness-of-fit on F^2	1.071

Final R indexes [I>=2σ (I)]	$R_1 = 0.0419$, $wR_2 = 0.1138$
Final R indexes [all data]	$R_1 = 0.0465$, $wR_2 = 0.1186$
Largest diff. peak/hole / e Å^{-3}	0.17/-0.22
Flack parameter	0.025(13)

Table S3. Fractional Atomic Coordinates (×10^4) and Equivalent Isotropic Displacement Parameters (Å^2×10^3) for 29. U_{eq} is defined as 1/3 of of the trace of the orthogonalised U_{IJ} tensor.

Atom	*x*	*y*	*z*	U(eq)
S1	7950.3(4)	4541(2)	8455.3(11)	80.9(4)
N1	5498.4(11)	4997(4)	8285(2)	42.9(6)
N2	4655.7(11)	5098(4)	6710(2)	46.7(6)
N3	3185.1(14)	2283(5)	6060(3)	57.7(7)
C1	7516(2)	3026(7)	9190(4)	69.7(11)
C2	6839.2(16)	3466(6)	9045(3)	55.8(8)
C3	6691.9(14)	5054(5)	8331(2)	43.2(7)
C4	7250.2(15)	5776(6)	7953(3)	54.6(8)
C5	6011.9(13)	5818(5)	7994(2)	41.6(7)
C6	5877.3(15)	7582(5)	7294(3)	50.6(8)
C7	5164.9(15)	8098(5)	7529(3)	51.3(8)
C8	4900.8(13)	6082(5)	7777(2)	44.4(7)
C9	4323.2(15)	5963(6)	8495(3)	53.1(8)
C10	3861.8(15)	4445(7)	7923(3)	58.4(9)
C11	4104.8(13)	4269(5)	6795(2)	43.4(7)
C12	3738.4(14)	3180(5)	5847(3)	45.1(7)
C13	3963.2(16)	3135(5)	4806(3)	50.3(8)
C14	3605.7(19)	2088(6)	3951(3)	60.3(9)
C15	3036(2)	1150(7)	4158(3)	67.1(10)
C16	2845.2(18)	1287(7)	5226(4)	68.6(11)
C17	5225(2)	9361(7)	8573(4)	69.1(10)
C18	4739.7(19)	9092(7)	6549(4)	74.0(12)

Table S4 Anisotropic Displacement Parameters (Å^2×10^3) for 29. The Anisotropic displacement factor exponent takes the form: $-2\pi^2[h^2a^{*2}U_{11}+2hka^*b^*U_{12}+\ldots]$.

Atom	U_{11}	U_{22}	U_{33}	U_{23}	U_{13}	U_{12}
S1	38.1(4)	90.0(9)	114.9(8)	7.7(7)	11.7(4)	12.8(5)
N1	34.7(11)	44.4(16)	49.5(12)	4.1(12)	6.1(9)	-0.9(11)
N2	32.6(11)	59.3(19)	48.3(12)	-3.0(12)	6.2(9)	0.5(11)
N3	45.2(13)	63(2)	65.4(15)	-5.1(15)	10.7(12)	-8.2(13)
C1	59(2)	67(3)	80(2)	19(2)	0.1(17)	15(2)
C2	47.3(16)	58(2)	61.4(17)	10.8(17)	2.9(13)	4.2(16)

C3	35.7(13)	44.5(19)	49.0(14)	-0.8(13)	4.0(10)	1.3(12)
C4	37.3(14)	57(2)	70.2(19)	2.2(18)	10.8(13)	0.8(15)
C5	34.4(13)	46.2(19)	44.1(13)	0.1(13)	4.5(10)	0.7(12)
C6	42.4(15)	51(2)	59.6(17)	13.1(15)	9.8(13)	-1.1(14)
C7	43.2(15)	51(2)	59.9(17)	6.5(16)	8.1(13)	5.9(15)
C8	33.8(13)	51(2)	48.4(14)	0.7(14)	7.0(11)	2.5(13)
C9	39.1(14)	67(2)	54.7(16)	-5.0(16)	11.6(12)	1.2(15)
C10	49.3(15)	73(3)	55.3(16)	-5.5(18)	16.4(13)	-14.7(19)
C11	34.7(12)	48.4(19)	47.4(14)	3.4(13)	6.9(10)	7.2(13)
C12	36.4(12)	46.5(19)	52.1(15)	3.8(14)	4.8(11)	5.2(13)
C13	48.0(15)	50(2)	53.3(16)	3.5(15)	7.2(12)	2.8(15)
C14	72(2)	57(2)	51.6(17)	-1.4(17)	6.1(15)	4.0(19)
C15	67(2)	65(3)	66(2)	-16(2)	-0.9(17)	-6(2)
C16	52.8(19)	74(3)	79(2)	-13(2)	9.8(17)	-24(2)
C17	72(2)	55(3)	83(2)	-8(2)	17.9(18)	-2(2)
C18	56.9(19)	73(3)	90(3)	26(2)	3.4(18)	16(2)

Table S5 Bond Lengths for 29.

Atom	Atom	Length/Å	Atom	Atom	Length/Å
S1	C1	1.698(5)	C6	C7	1.542(4)
S1	C4	1.694(3)	C7	C8	1.555(5)
N1	C5	1.273(4)	C7	C17	1.527(5)
N1	C8	1.483(4)	C7	C18	1.527(5)
N2	C8	1.481(4)	C8	C9	1.541(4)
N2	C11	1.268(4)	C9	C10	1.515(5)
N3	C12	1.334(4)	C10	C11	1.506(4)
N3	C16	1.332(5)	C11	C12	1.482(4)
C1	C2	1.384(5)	C12	C13	1.386(4)
C2	C3	1.412(5)	C13	C14	1.383(5)
C3	C4	1.365(4)	C14	C15	1.374(6)
C3	C5	1.474(4)	C15	C16	1.392(6)
C5	C6	1.501(5)			

Table S6 Bond Angles for 29.

Atom	Atom	Atom	Angle/°	Atom	Atom	Atom	Angle/°
C4	S1	C1	92.50(19)	C18	C7	C17	110.0(4)
C5	N1	C8	107.3(3)	N1	C8	C7	105.5(2)
C11	N2	C8	109.6(2)	N1	C8	C9	111.9(3)
C16	N3	C12	117.4(3)	N2	C8	N1	105.8(2)
C2	C1	S1	111.2(3)	N2	C8	C7	109.9(2)

C1	C2	C3	112.0(3)	N2	C8	C9	105.8(2)
C2	C3	C5	124.3(3)	C9	C8	C7	117.4(3)
C4	C3	C2	112.4(3)	C10	C9	C8	104.3(3)
C4	C3	C5	123.3(3)	C11	C10	C9	102.2(3)
C3	C4	S1	111.9(3)	N2	C11	C10	115.4(3)
N1	C5	C3	121.2(3)	N2	C11	C12	121.6(3)
N1	C5	C6	115.8(3)	C12	C11	C10	123.0(3)
C3	C5	C6	123.0(3)	N3	C12	C11	115.9(3)
C5	C6	C7	101.1(3)	N3	C12	C13	123.1(3)
C6	C7	C8	99.9(3)	C13	C12	C11	121.0(3)
C17	C7	C6	108.4(3)	C14	C13	C12	118.8(3)
C17	C7	C8	111.2(3)	C15	C14	C13	118.9(3)
C18	C7	C6	113.8(3)	C14	C15	C16	118.4(3)
C18	C7	C8	113.2(3)	N3	C16	C15	123.5(4)

Table S7 Torsion Angles for 29.

A	B	C	D	Angle/°	A	B	C	D	Angle/°
S1	C1	C2	C3	-0.2(5)	C7	C8	C9	C10	138.4(3)
N1	C5	C6	C7	18.4(4)	C8	N1	C5	C3	-178.0(3)
N1	C8	C9	C10	-99.3(3)	C8	N1	C5	C6	1.8(4)
N2	C8	C9	C10	15.4(4)	C8	N2	C11	C10	-1.6(4)
N2	C11	C12	N3	176.3(3)	C8	N2	C11	C12	179.5(3)
N2	C11	C12	C13	-4.3(5)	C8	C9	C10	C11	-15.5(4)
N3	C12	C13	C14	-1.1(5)	C9	C10	C11	N2	11.5(4)
C1	S1	C4	C3	-0.1(3)	C9	C10	C11	C12	-169.6(3)
C1	C2	C3	C4	0.1(5)	C10	C11	C12	N3	-2.6(5)
C1	C2	C3	C5	178.1(3)	C10	C11	C12	C13	176.8(3)
C2	C3	C4	S1	0.0(4)	C11	N2	C8	N1	109.9(3)
C2	C3	C5	N1	-4.2(5)	C11	N2	C8	C7	-136.6(3)
C2	C3	C5	C6	176.0(3)	C11	N2	C8	C9	-9.0(4)
C3	C5	C6	C7	-161.8(3)	C11	C12	C13	C14	179.6(3)
C4	S1	C1	C2	0.2(4)	C12	N3	C16	C15	-0.4(6)
C4	C3	C5	N1	173.5(3)	C12	C13	C14	C15	0.8(6)
C4	C3	C5	C6	-6.3(5)	C13	C14	C15	C16	-0.4(6)
C5	N1	C8	N2	95.0(3)	C14	C15	C16	N3	0.1(7)
C5	N1	C8	C7	-21.5(3)	C16	N3	C12	C11	-179.8(3)
C5	N1	C8	C9	-150.3(3)	C16	N3	C12	C13	0.9(6)
C5	C3	C4	S1	-177.9(2)	C17	C7	C8	N1	-83.3(3)
C5	C6	C7	C8	-28.3(3)	C17	C7	C8	N2	163.1(3)
C5	C6	C7	C17	88.2(3)	C17	C7	C8	C9	42.2(4)

C5	C6	C7	C18	-149.2(3)	C18	C7	C8	N1	152.4(3)
C6	C7	C8	N1	31.0(3)	C18	C7	C8	N2	38.7(4)
C6	C7	C8	N2	-82.6(3)	C18	C7	C8	C9	-82.2(4)
C6	C7	C8	C9	156.5(3)					

Table S8 Hydrogen Atom Coordinates (Å×10^4) and Isotropic Displacement Parameters (Å^2×10^3) for 29.

**Atom**	***x***	***y***	***z***	**U(eq)**
H1	7708	2030	9633	84
H2	6519	2796	9377	67
H4	7246	6820	7476	66
H6A	5887	7322	6503	61
H6B	6196	8584	7538	61
H9A	4489	5599	9262	64
H9B	4092	7175	8503	64
H10A	3910	3252	8333	70
H10B	3397	4847	7844	70
H13	4347	3796	4686	60
H14	3749	2019	3247	72
H15	2784	441	3598	80
H16	2460	650	5365	82
H17A	5510	8754	9175	104
H17B	4788	9556	8791	104
H17C	5414	10569	8409	104
H18A	4940	10295	6408	111
H18B	4296	9299	6733	111
H18C	4716	8308	5890	111

7. Characterization of adducts

(*R*)-4,4-dimethyl-7-(pyridin-2-yl)-2-(*p*-tolyl)-1,6-diazaspiro[4.4]nona-1,6-diene (6)

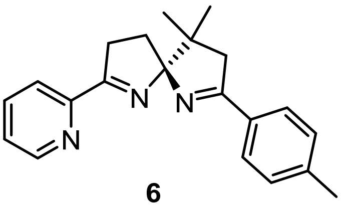

6

White solid; Mp 130.2−131.1 °C; 22.2 mg, 70% yield, 90% ee; $[\alpha]_D^{22}$ −36.1 (*c* 1.0, CH_3OH); **^{1}H NMR** (400 MHz, $CDCl_3$) δ 8.66 – 8.62 (m, 1H), 8.16 (d, *J* = 7.9 Hz, 1H), 7.76 (d, *J* = 8.1 Hz, 2H), 7.70 (td, *J* = 7.7, 1.8 Hz, 1H), 7.30 (m, 1H), 7.19 (d, *J* = 8.0 Hz, 2H), 3.39 (m, 1H), 3.22 (m, 2H), 2.87 (d, *J* = 15.9 Hz, 1H), 2.37 (s, 3H), 2.30 (m, 1H), 2.15 (m, 1H), 1.15 (s, 3H), 1.06 (s, 3H); **^{13}C NMR** (101 MHz, $CDCl_3$) δ 174.6, 174.4, 153.6, 149.1, 140.9, 136.2, 132.3, 129.1, 127.9, 124.7, 122.7, 111.2, 50.1, 45.0, 35.3, 28.5, 25.1, 22.0, 21.6; **HRMS** (ESI) m/z 317.1892 (M +H$^+$), calc. for $C_{21}H_{23}N_3^+$ 317.1892.

The ee was determined by HPLC analysis: CHIRALPAK IE (4.6 mm i.d. x 250 mm); hexane/2-propanol = 80/20; flow rate 1.0 mL/min; 25 °C; 254 nm; retention time: 12.0 min (major) and 15.8 min (minor).

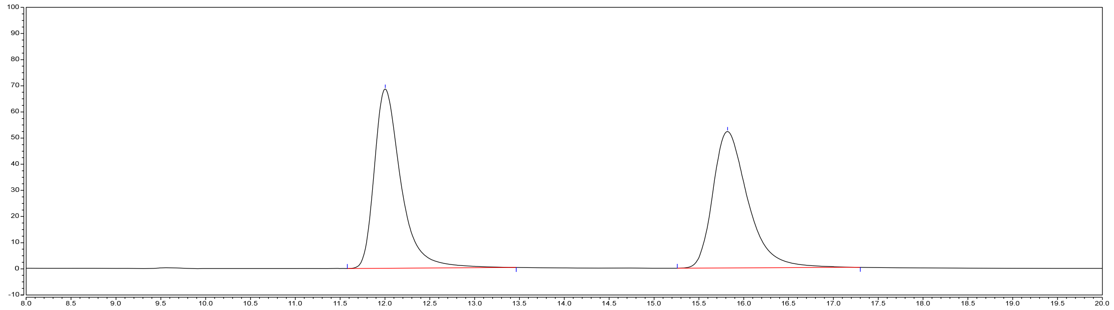

Entry	Retention Time	Area	Height	%Area
1	12.003	23.6889	68.55	50.02
2	15.820	23.6721	52.20	49.98

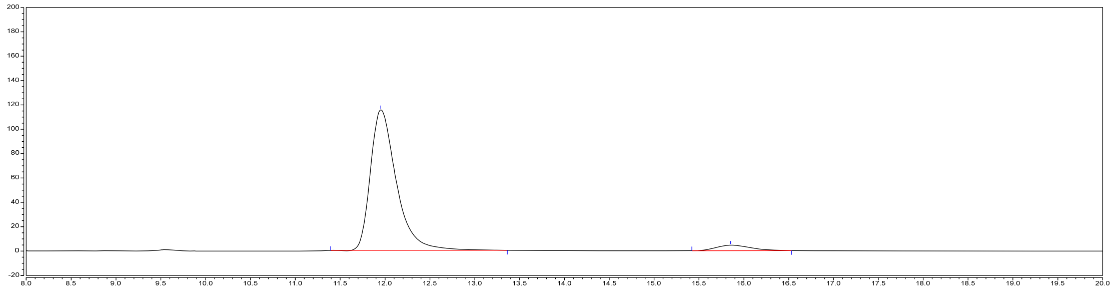

Entry	Retention Time	Area	Height	%Area
1	11.953	38.6644	115.39	95.00
2	15.853	2.0343	4.55	5.00

(*R*)-methyl-4-(4,4-dimethyl-7-(pyridin-2-yl)-1,6-diazaspiro[4.4]nona-1,6-dien-2-yl) benzoate (7)

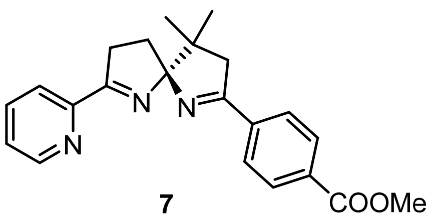

7

Yellow solid; Mp 116.3−117.1 °C; 19.6 mg, 54% yield, 90% ee; $[\alpha]_D^{22}$ +218.9 (*c* 1.0, CH_3OH); **^{1}H NMR** (400 MHz, CD_2Cl_2) δ 8.62 (d, *J* = 4.8 Hz, 1H), 8.13 (d, *J* = 7.9 Hz, 1H), 8.05 (d, *J* = 8.4 Hz, 2H), 7.93 (d, *J* = 8.3 Hz, 2H), 7.72 (td, *J* = 7.7, 1.7 Hz, 1H), 7.36 – 7.28 (m, 1H), 3.90 (s, 3H), 3.28 (m, 2H), 3.23 (d, *J* = 15.8 Hz, 1H), 2.90 (d, *J* = 16.1 Hz, 1H), 2.31 – 2.22 (m, 1H), 2.13 (m, 1H), 1.17 (s, 3H), 1.06 (s, 3H); **^{13}C NMR** (101 MHz, CD_2Cl_2) δ 170.4, 168.6, 161.8, 148.7, 144.4, 134.3, 131.4, 127.2, 124.8, 123.0, 120.1, 117.4, 106.7, 47.4, 45.2, 40.1, 30.5, 23.6, 20.1, 16.8; **HRMS** (ESI) m/z 362.1862 (M +H$^+$), calc. for $C_{22}H_{24}N_3O_2^+$ 362.1863.

The ee was determined by HPLC analysis: CHIRALPAK IC (4.6 mm i.d. x 250 mm); hexane/2-propanol = 80/20; flow rate 1.0 mL/min; 25 °C; 254 nm; retention time: 16.5 min (minor) and 23.6 min (major).

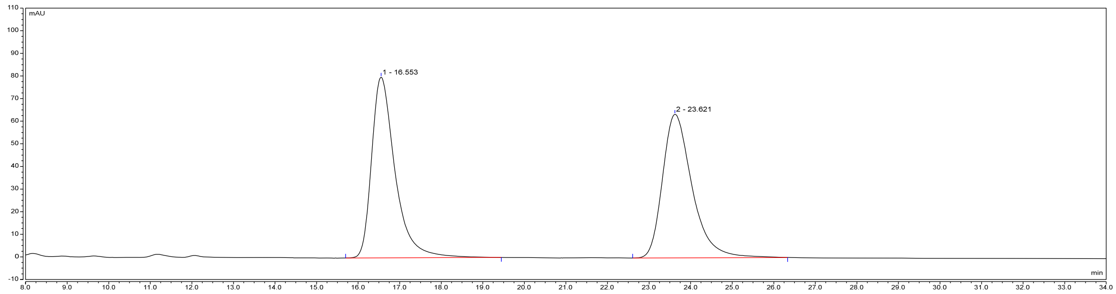

Entry	Retention Time	Area	Height	%Area
1	16.553	79.95	52.3562	50.05
2	23.621	63.52	52.2507	49.95

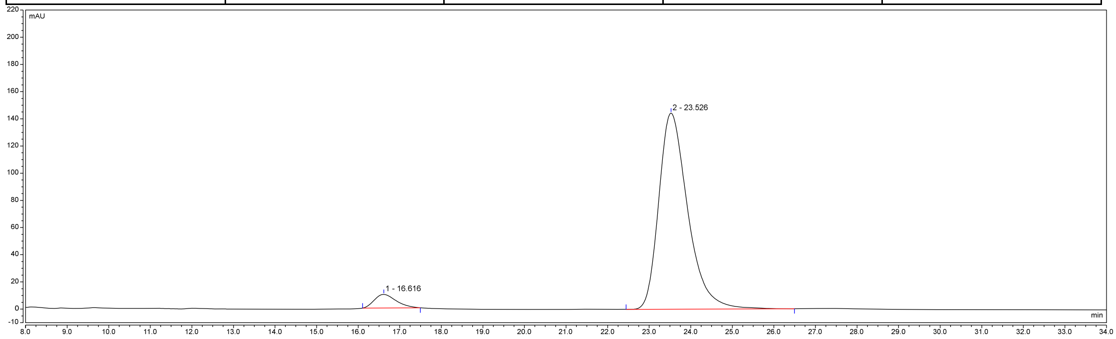

Entry	Retention Time	Area	Height	%Area
1	16.616	6.0873	10.00	4.97
2	23.526	116.4727	144.26	95.03

(*R*)-4,4-dimethyl-7-(pyridin-2-yl)-2-(4-(trifluoromethyl)phenyl)-1,6-diazaspiro[4.4]nona-1,6-diene (8)

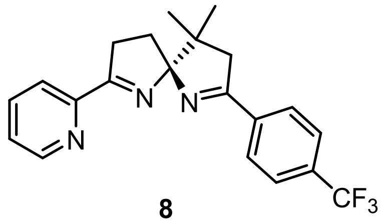

8

Yellow solid; Mp 112.8−113.6 °C; 20.5 mg, 55% yield, 90% ee; $[\alpha]_D^{22}$ −39.6 (*c* 1.0, CH_3OH); **^{1}H NMR** (400 MHz, $CDCl_3$) δ 8.68 – 8.61 (m, 1H), 8.15 (d, *J* = 7.9 Hz, 1H), 7.97 (d, *J* = 8.1 Hz, 2H), 7.69 (td, *J* = 7.7, 1.7 Hz, 1H), 7.64 (d, *J* = 8.2 Hz, 2H), 7.30 (m, 1H), 3.40 (m, 1H), 3.32 – 3.20 (m, 2H), 2.87 (d, *J* = 16.1 Hz, 1H), 2.36 – 2.27 (m, 1H), 2.15 (m, 1H), 1.17 (s, 3H), 1.07 (s, 3H); **^{13}C NMR** (151 MHz, $CDCl_3$) δ 175.4, 173.6, 153.3, 149.2, 138.3, 136.3, 132.4 (q, *J* = 32.7 Hz), 128.3, 125.4 (q, *J* = 3.6 Hz), 124.9, 124.1 (q, *J* = 270.7 Hz), 111.5, 107.8,50.1, 45.2, 35.4, 28.4, 25.1, 21.8; **^{19}F NMR** (376 MHz, CD_3OD) δ -64.33; **HRMS** (ESI) 372.1680 m/z (M +H$^+$), calc. for $C_{21}H_{21}F_3N_3^+$ 372.1682.

The ee was determined by HPLC analysis: CHIRALPAK IE (4.6 mm i.d. x 250 mm); hexane/2-propanol = 80/20; flow rate 1.0 mL/min; 25 °C; 254 nm; retention time: 9.2 min (major) and 12.9 min (minor).

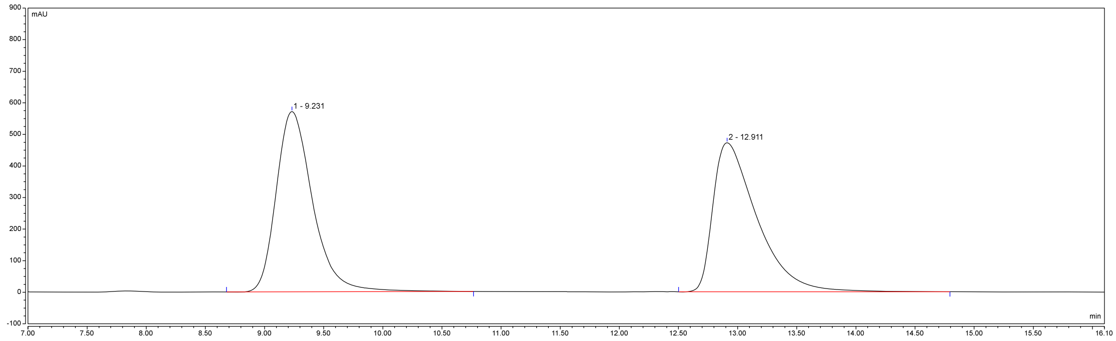

Entry	Retention Time	Area	Height	%Area
1	9.231	203.7416	570.71	50.05
2	12.911	203.3037	472.14	49.95

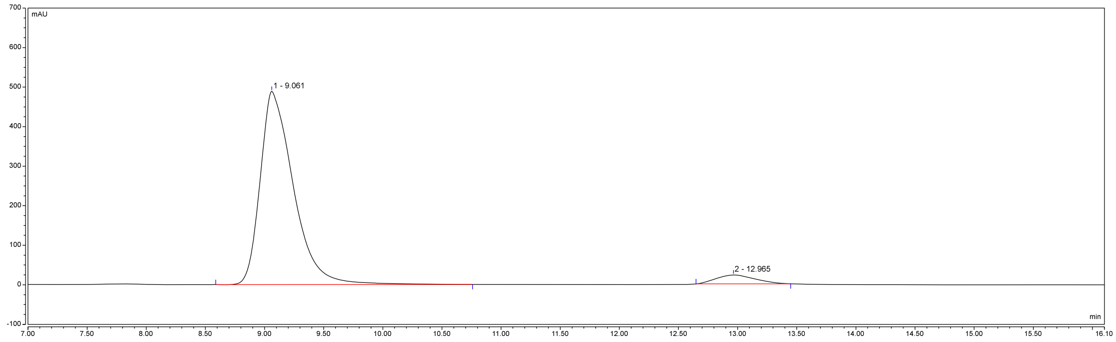

Entry	Retention Time	Area	Height	%Area

1	9.061	158.7658	489.03	95.00
2	12.965	8.3636	22.14	5.00

(*R*)-2-(3-fluorophenyl)-4,4-dimethyl-7-(pyridin-2-yl)-1,6-diazaspiro[4.4]nona-1,6-diene (9)

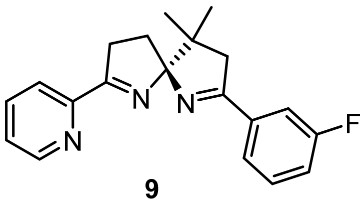

9

Yellow solid; Mp 149.3−150.1 °C; 26.4 mg, 82% yield, 91% ee; $[\alpha]_D^{22}$ +51.6 (*c* 1.0, CH_3OH); **^{1}H NMR** (600 MHz, $CDCl_3$) δ 8.64 (d, *J* = 4.8 Hz, 1H), 8.16 (d, *J* = 7.9 Hz, 1H), 7.70 (td, *J* = 7.8, 1.6 Hz, 1H), 7.60 (m, 2H), 7.35 (m, 1H), 7.33 – 7.27 (m, 1H), 7.11 (td, *J* = 8.3, 2.3 Hz, 1H), 3.38 (m, 1H), 3.29 – 3.20 (m, 2H), 2.84 (d, *J* = 16.0 Hz, 1H), 2.30 (m, 1H), 2.21 – 2.08 (m, 1H), 1.17 (s, 3H), 1.07 (s, 3H); **^{13}C NMR** (151 MHz, $CDCl_3$) δ 175.1, 173.6 (d, *J* = 2.6 Hz), 163.7, 162.1, 153.5, 149.2, 137.4 (d, *J* = 7.5 Hz), 136.3, 129.9 (d, *J* = 7.8 Hz), 124.9, 123.7 (d, *J* = 2.3 Hz), 122.7, 117.6 (d, *J* = 21.7 Hz), 114.7 (d, *J* = 22.2 Hz), 111.4, 50.2, 45.2, 35.4, 28.5, 25.2, 21.9; **^{19}F NMR** (376 MHz, CD_3OD) δ -114.24; **HRMS** (ESI) 322.1714 m/z ($M + H^+$), calc. for $C_{20}H_{21}FN_3^+$ 322.1714.

The ee was determined by HPLC analysis: CHIRALPAK IE (4.6 mm i.d. x 250 mm); hexane/2-propanol = 80/20; flow rate 1.0 mL/min; 25 °C; 254 nm; retention time: 9.2 min (major) and 13.6 min (minor).

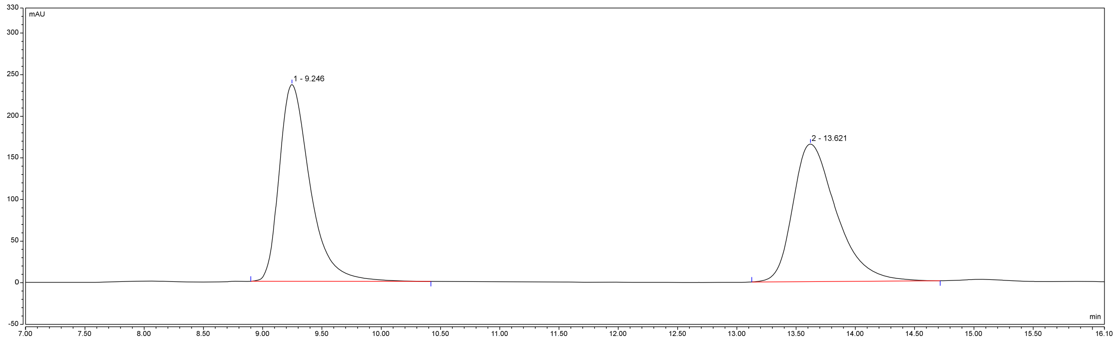

Entry	Retention Time	Area	Height	%Area
1	9.246	71.2050	236.37	50.35
2	13.621	70.2168	165.42	49.65

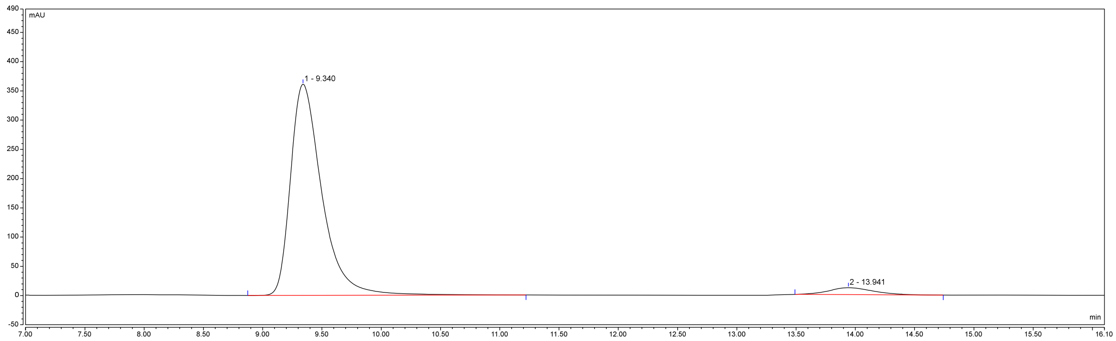

Entry	Retention Time	Area	Height	%Area
1	9.340	110.5999	360.89	95.63
2	13.941	5.0538	11.71	4.37

(*R*)-2-(2-fluorophenyl)-4,4-dimethyl-7-(pyridin-2-yl)-1,6-diazaspiro[4.4]nona-1,6-diene (10)

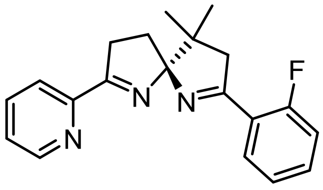

10

White solid; Mp 145.1−145.9 °C; 20.0 mg, 62% yield, 99% ee; $[\alpha]_D^{22}$ +54.7 (*c* 1.0, CH_3OH); **^{1}H NMR** (600 MHz, $CDCl_3$) δ 8.63 (d, J = 4.2 Hz, 1H), 8.18 (d, J = 7.9 Hz, 1H), 7.98 (td, J = 7.7, 1.6 Hz, 1H), 7.73 – 7.65 (m, 1H), 7.40 – 7.33 (m, 1H), 7.31 – 7.26 (m, 1H), 7.13 (t, J = 7.6 Hz, 1H), 7.06 (dd, J = 11.2, 8.4 Hz, 1H), 3.39 – 3.30 (m, 2H), 3.24 (m, 1H), 2.92 (dd, J = 16.7, 3.5 Hz, 1H), 2.28 (m, 1H), 2.15 (m, 1H), 1.15 (s, 3H), 1.06 (s, 3H); **^{13}C NMR** (101 MHz, $CDCl_3$) δ 175.0, 172.1 (d, *J* = 1.8 Hz), 161.9 (d, *J* = 252.8 Hz), 153.5, 149.1, 136.2, 132.2 (d, *J* = 8.7 Hz), 130.1 (d, *J* = 3.5 Hz), 124.8, 124.2 (d, *J* = 3.3 Hz), 123.3 (d, *J* = 11.8 Hz), 122.7, 116.2 (d, *J* = 22.6 Hz), 110.2, 53.23 (d, *J* = 6.0 Hz), 45.1 (d, *J* = 1.4 Hz), 35.3, 28.3, 24.9, 21.8; **^{19}F NMR** (565 MHz, $CDCl_3$) δ -112.7; **HRMS** (ESI) 322.1714 m/z (M + H^+), calc. for $C_{20}H_{21}FN_3^+$ 322.1714.

The ee was determined by HPLC analysis: CHIRALPAK IE (4.6 mm i.d. x 250 mm); hexane/2-propanol = 80/20; flow rate 1.0 mL/min; 25 °C; 254 nm; retention time: 9.0 min (major) and 11.2 min (minor).

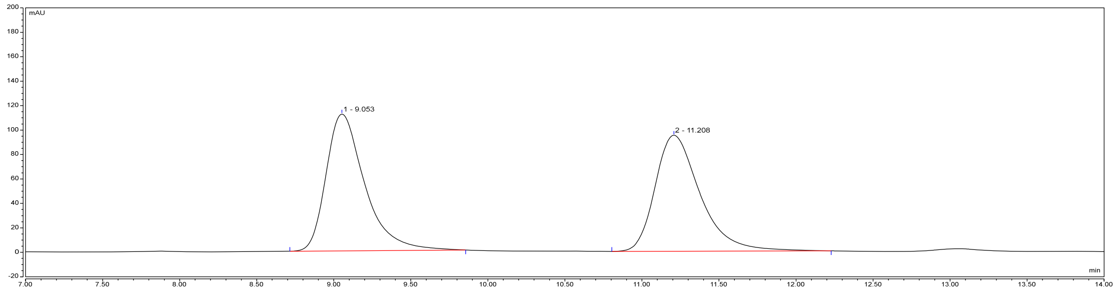

Entry	Retention Time	Area	Height	%Area

1	9.053	32.6268	112.00	50.42
2	11.208	32.0861	94.88	49.58

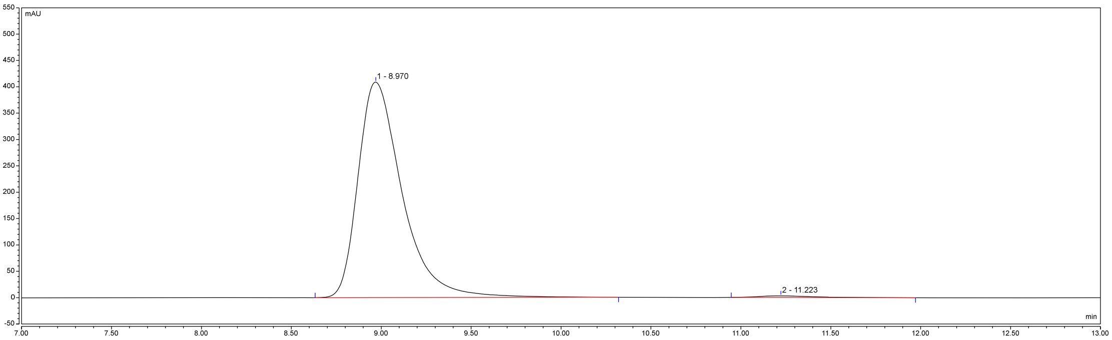

Entry	Retention Time	Area	Height	%Area
1	8.970	115.8794	408.87	99.07
2	11.223	1.0863	3.19	0.93

(*R*)-2-(3-chlorophenyl)-4,4-dimethyl-7-(pyridin-2-yl)-1,6-diazaspiro[4.4]nona-1,6-diene (11)

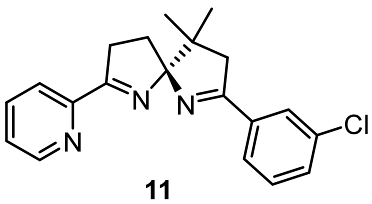

11

Yellow solid; Mp 61.2−62.0 °C; 18.3 mg, 54% yield, 91% ee; $[\alpha]_D^{22}$ +315.5 (*c* 1.0, CH_3OH); **^{1}H NMR** (600 MHz, $CDCl_3$) δ 8.64 (d, *J* = 4.1 Hz, 1H), 8.16 (d, *J* = 7.9 Hz, 1H), 7.88 (s, 1H), 7.76 – 7.65 (m, 2H), 7.38 (d, *J* = 7.9 Hz, 1H), 7.33 – 7.27 (m, 2H), 3.38 (dd, *J* = 17.4, 8.7 Hz, 1H), 3.25 (m, 2H), 2.83 (d, *J* = 16.0 Hz, 1H), 2.34 – 2.25 (m, 1H), 2.14 (m, 1H), 1.16 (s, 3H), 1.06 (s, 3H); **^{13}C NMR** (151 MHz, $CDCl_3$) δ 175.1, 173.6, 153.5, 149.2, 136.3, 134.6, 130.75, 129.7, 127.9, 126.2, 124.9, 122.7, 111.4, 50.1, 45.2, 35.4, 28.4, 25.2, 21.9; **HRMS** (ESI) 338.1419 m/z (M + H^+), calc. for $C_{20}H_{21}ClN_3^+$ 338.1419.

The ee was determined by HPLC analysis: CHIRALPAK IE (4.6 mm i.d. x 250 mm); hexane/2-propanol = 80/20; flow rate 1.0 mL/min; 25 °C; 254 nm; retention time: 9.5 min (major) and 14.8 min (minor).

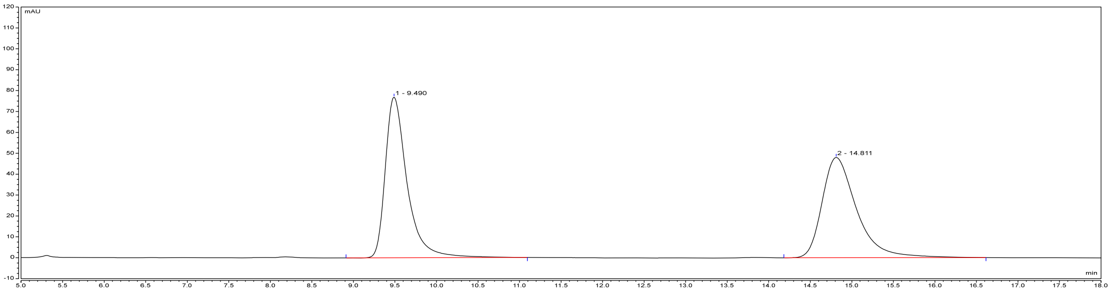

Entry	Retention Time	Area	Height	%Area
1	9.490	23.4172	76.86	50.32
2	14.811	23.1219	47.97	49.68

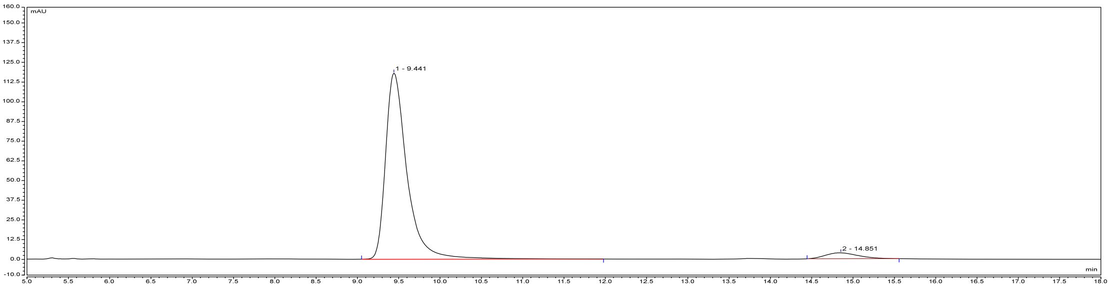

Entry	Retention Time	Area	Height	%Area
1	9.441	35.5181	118.01	95.35
2	14.851	1.7321	3.78	4.65

(*R*)-2-(3-bromophenyl)-4,4-dimethyl-7-(pyridin-2-yl)-1,6-diazaspiro[4.4]nona-1,6-diene (12)

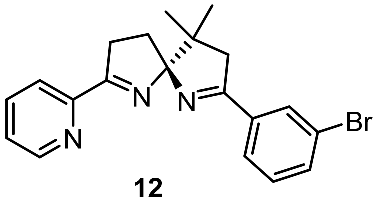

12

White solid; Mp 140.0−140.9 °C; 20.2 mg, 53% yield, 91% ee; $[\alpha]_D^{22}$ +236.3 (*c* 1.0, CH_3OH); **^{1}H NMR** (600 MHz, $CDCl_3$) δ 8.66 (d, *J* = 4.4 Hz, 1H), 8.18 (d, *J* = 7.9 Hz, 1H), 8.06 (s, 1H), 7.78 (d, *J* = 7.6 Hz, 1H), 7.71 (td, *J* = 7.8, 1.5 Hz, 1H), 7.56 (d, *J* = 7.9 Hz, 1H), 7.32 (dd, *J* = 6.5, 5.1 Hz, 1H), 7.28 (d, *J* = 7.6 Hz, 1H), 3.41 (m, 1H), 3.31 – 3.21 (m, 2H), 2.85 (d, *J* = 16.0 Hz, 1H), 2.35 – 2.27 (m, 1H), 2.16 (m, 1H), 1.18 (s, 3H), 1.08 (s, 3H); **^{13}C NMR** (151 MHz, $CDCl_3$) δ 175.1, 173.4, 153.4, 149.2, 137.1, 136.2, 133.6, 130.8, 129.9, 126.5, 124.8, 122.7, 122.7, 111.3, 50.0, 45.1, 35.3, 28.4, 25.1, 21.9; **HRMS** (ESI) 382.0915 m/z (M + H^+), calc. for $C_{20}H_{21}BrN_3^+$ 382.0913.

The ee was determined by HPLC analysis: CHIRALPAK IE (4.6 mm i.d. x 250 mm); hexane/2-propanol = 80/20; flow rate 1.0 mL/min; 25 °C; 254 nm; retention time: 9.9 min (major) and 15.7 min (minor).

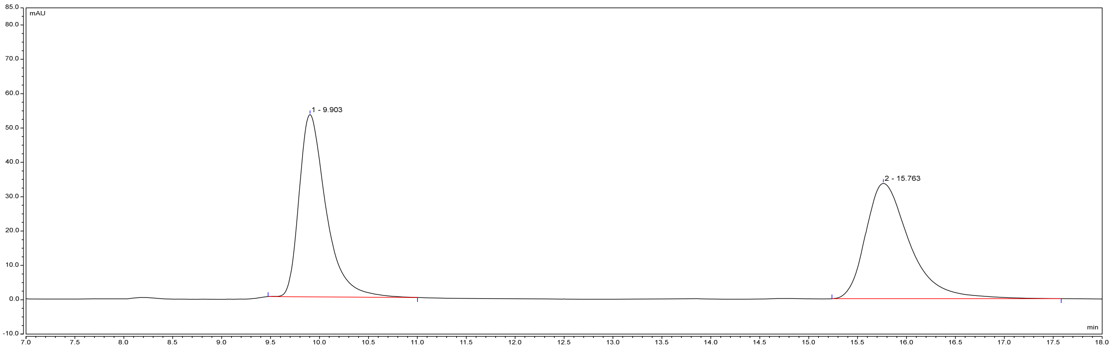

Entry	Retention Time	Area	Height	%Area

1	9.903	16.9639	53.04	49.22
2	15.763	17.4990	33.57	50.78

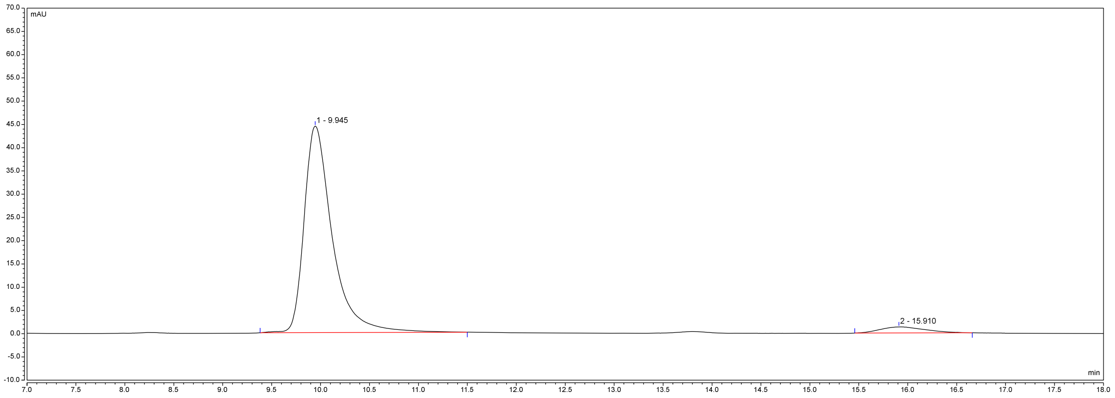

Entry	Retention Time	Area	Height	%Area
1	9.945	14.8335	44.40	95.58
2	15.910	0.6852	1.31	4.42

(*R*)-2-(2-bromophenyl)-4,4-dimethyl-7-(pyridin-2-yl)-1,6-diazaspiro[4.4]nona-1,6-diene (13)

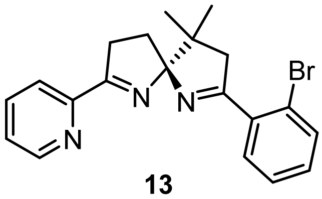

13

White solid; Mp 128.4−129.2 °C; 30.9 mg, 81% yield, 95% ee; $[\alpha]_D^{22}$ −27.1 (*c* 1.0, CH_3OH); **^{1}H NMR** (400 MHz, $CDCl_3$) δ 8.64 (d, *J* = 4.4 Hz, 1H), 8.21 (d, *J* = 7.9 Hz, 1H), 7.72 (td, *J* = 7.7, 1.6 Hz, 1H), 7.57 (d, *J* = 7.9 Hz, 1H), 7.50 (dd, *J* = 7.6, 1.5 Hz, 1H), 7.34 – 7.26 (m, 2H), 7.20 (td, *J* = 7.8, 1.6 Hz, 1H), 3.40 – 3.27 (m, 3H), 2.98 (d, *J* = 16.4 Hz, 1H), 2.37 – 2.27 (m, 1H), 2.13 (m, 1H), 1.17 (s, 3H), 1.15 (s, 3H); **^{13}C NMR** (151 MHz, $CDCl_3$) δ 176.2, 174.3, 152.5, 148.1, 136.9, 135.3, 132.3, 129.6, 129.5, 126.4, 123.8, 121.7, 120.3, 109.9, 76.4, 76.2, 75.9, 52.2, 44.6, 34.2, 27.2, 23.8, 20.7; **HRMS** (ESI) 382.0912 m/z ($M + H^+$), calc. for $C_{20}H_{21}BrN_3^+$ 382.0913.

The ee was determined by HPLC analysis: CHIRALPAK IE (4.6 mm i.d. x 250 mm); hexane/2-propanol = 80/20; flow rate 1.0 mL/min; 25 °C; 254 nm; retention time: 10.4 min (minor) and 12.1 min (major).

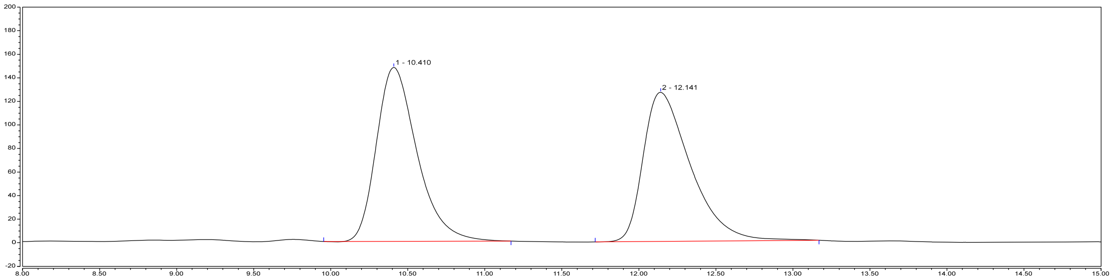

Entry	Retention Time	Area	Height	%Area

1	10.410	45.1504	147.62	50.07
2	12.141	45.0170	126.62	49.93

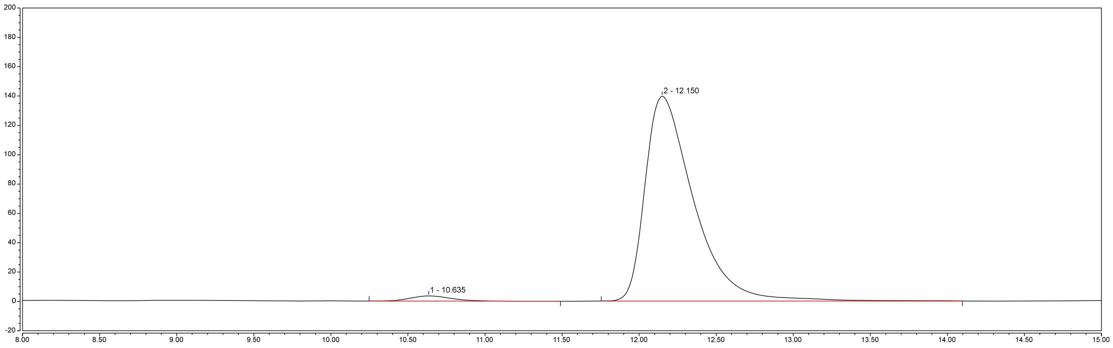

Entry	Retention Time	Area	Height	%Area
1	10.635	1.1155	3.59	2.14
2	12.150	51.0606	139.65	97.86

(*R*)-4,4-dimethyl-2-phenyl-7-(pyridin-2-yl)-1,6-diazaspiro[4.4]nona-1,6-diene (14)

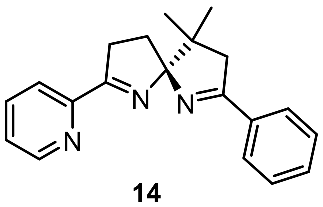

14

White solid; Mp 99.8−100.6 °C; 26.5 mg, 87% yield, 90% ee; $[\alpha]_D^{22}$ +169.6 (*c* 1.0, CH_3OH); **^{1}H NMR** (600 MHz, $CDCl_3$) δ 8.64 (d, *J* = 4.4 Hz, 1H), 8.16 (d, *J* = 7.9 Hz, 1H), 7.87 (d, *J* = 7.0 Hz, 2H), 7.68 (td, *J* = 7.7, 1.7 Hz, 1H), 7.43 – 7.36 (m, 3H), 7.31 – 7.26 (m, 1H), 3.40 (m, 1H), 3.24 (m, 2H), 2.88 (d, *J* = 15.9 Hz, 1H), 2.36 – 2.27 (m, 1H), 2.15 (m, 1H), 1.17 (s, 3H), 1.07 (s, 3H); **^{13}C NMR** (101 MHz, $CDCl_3$) δ 174.8, 174., 153.5, 149.1, 136.2, 135.0, 130.7, 128.4, 127.9, 124.7, 122.7, 111.3, 50.1, 45.0, 35.3, 28.5, 25.1, 21.9; **HRMS** (ESI) 304.1808 m/z (M +H$^+$), calc. for $C_{20}H_{22}N_3^+$ 304.1808.

The ee was determined by HPLC analysis: CHIRALPAK IE (4.6 mm i.d. x 250 mm); hexane/2-propanol = 80/20; flow rate 1.0 mL/min; 25 °C; 254 nm; retention time: 11.1 min (major) and 15.6 min (minor).

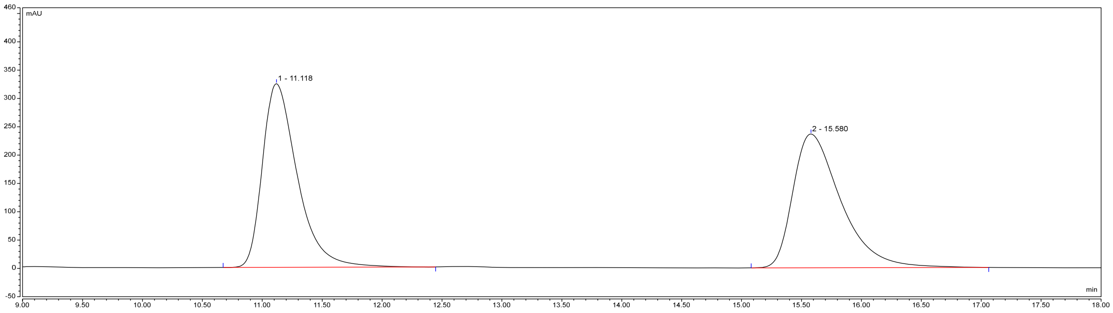

Entry	Retention Time	Area	Height	%Area
1	11.118	109.6161	324.15	50.02
2	15.580	109.5438	236.12	49.98

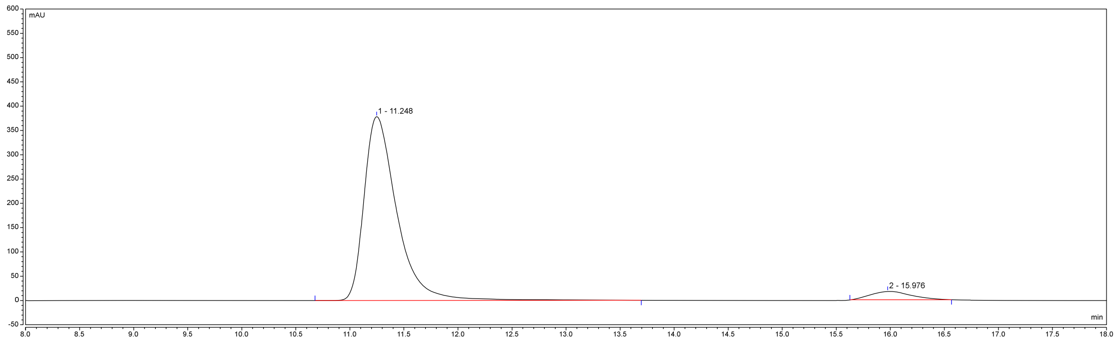

Entry	Retention Time	Area	Height	%Area
1	11.248	133.0498	378.38	94.72
2	15.976	7.4211	17.13	5.28

(*R*)-2-(4-(*tert*-butyl)phenyl)-4,4-dimethyl-7-(pyridin-2-yl)-1,6-diazaspiro[4.4]nona-1,6-diene (15)

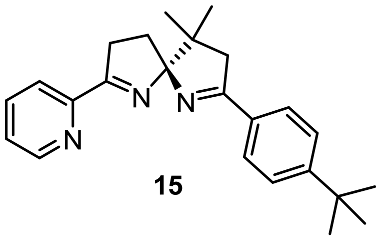

Yellow solid; Mp 87.0−87.9 °C; 25.9 mg, 72% yield, 87% ee; $[\alpha]^{22}_{D}$ +347.9 (*c* 1.0, CH_3OH); **^{1}H NMR** (600 MHz, $CDCl_3$) δ 8.64 (d, *J* = 4.0 Hz, 1H), 8.16 (d, *J* = 7.9 Hz, 1H), 7.82 (d, *J* = 8.0 Hz, 2H), 7.68 (t, *J* = 7.1 Hz, 1H), 7.41 (d, *J* = 8.2 Hz, 2H), 7.31 – 7.26 (m, 1H), 3.43 – 3.33 (m, 1H), 3.28 – 3.15 (m, 2H), 2.87 (d, *J* = 15.9 Hz, 1H), 2.33 – 2.26 (m, 1H), 2.15 (m, 1H), 1.32 (s, 9H), 1.16 (s, 3H), 1.06 (s, 3H); **^{13}C NMR** (151 MHz, $CDCl_3$) δ 174.6 174.4, 154.2, 153.6, 149.1, 136.2, 132.2, 127.7, 125.4, 124.7, 122.7, 111.2, 50.0, 45.0, 35.3, 34.9, 31.3, 28.4, 25.0, 22.0; **HRMS** (ESI) 360.2434 m/z (M + H^+), calc. for $C_{24}H_{30}N_3^+$ 360.2434.

The ee was determined by HPLC analysis: CHIRALPAK IE (4.6 mm i.d. x 250 mm); hexane/2-propanol = 80/20; flow rate 1.0 mL/min; 25 °C; 254 nm; retention time: 13.1 min (major) and 16.5 min (minor).

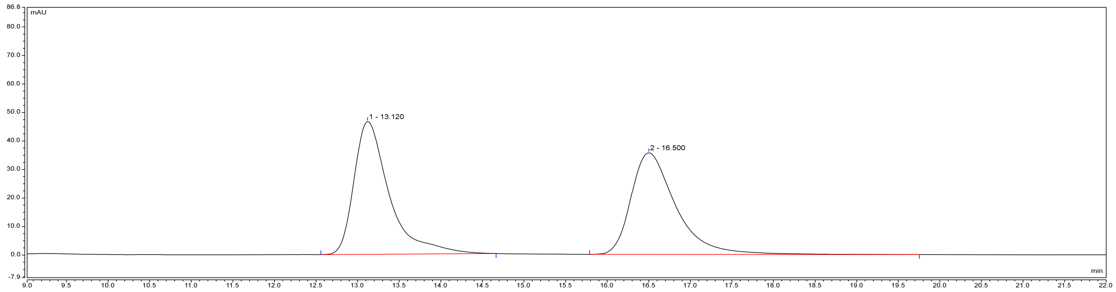

Entry	Retention Time	Area	Height	%Area
1	13.120	22.8950	46.56	50.78
2	16.500	22.1930	35.61	49.22

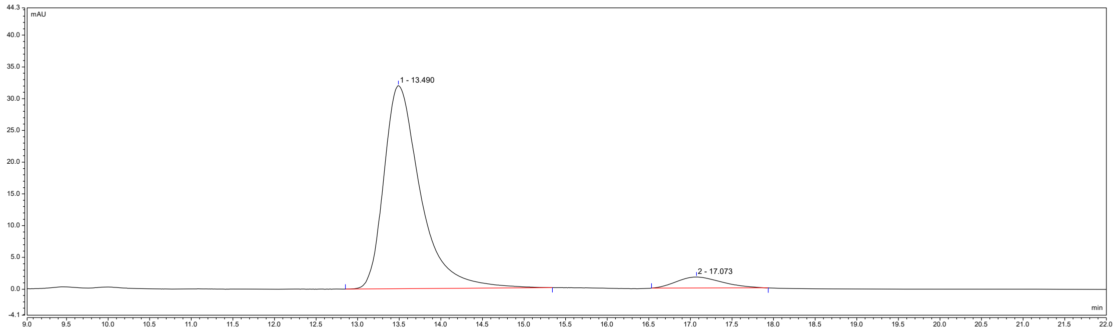

Entry	Retention Time	Area	Height	%Area
1	13.490	16.3046	31.99	93.73
2	17.073	1.0906	1.75	6.27

(*R*)-4,4-dimethyl-7-(pyridin-2-yl)-2-(4-(trimethylsilyl)phenyl)-1,6-diazaspiro[4.4]nona-1,6-diene (16)

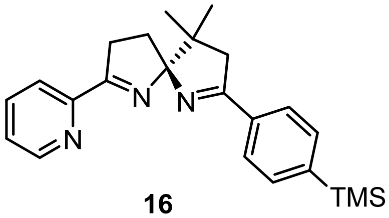

16

Yellow solid; Mp 121.2−122.0 °C; 16.9 mg, 45% yield, 86% ee; $[\alpha]_D^{22}$ +27.3 (*c* 1.0, CH_3OH); **^{1}H NMR** (600 MHz, $CDCl_3$) δ 8.64 (d, *J* = 4.8 Hz, 1H), 8.16 (d, *J* = 7.9 Hz, 1H), 7.85 (d, *J* = 8.0 Hz, 2H), 7.69 (td, *J* = 7.7, 1.7 Hz, 1H), 7.55 (d, *J* = 8.1 Hz, 2H), 7.29 (m, 1H), 3.39 (m, 1H), 3.30 – 3.18 (m, 2H), 2.88 (d, *J* = 15.9 Hz, 1H), 2.30 (m, 1H), 2.16 (m, 1H), 1.16 (s, 4H), 1.06 (s, 4H), 0.27 (s, 9H); **^{13}C NMR** (151 MHz, $CDCl_3$) δ 174.8, 174.7, 153.5, 149.1, 144.0, 136.2, 135.2, 133.4, 127.0, 124.8, 122.7, 111.2, 50.0, 45.0, 35.3, 28.4, 25.0, 22.0, -1.1; **HRMS** (ESI) 376.2204 m/z (M + H^+), calc. for $C_{23}H_{30}N_3Si^+$ 376.2204.

The ee was determined by HPLC analysis: CHIRALPAK IE (4.6 mm i.d. x 250 mm); hexane/2-propanol = 80/20; flow rate 1.0 mL/min; 25 °C; 254 nm; retention time: 8.3 min (major) and 10.6 min (minor).

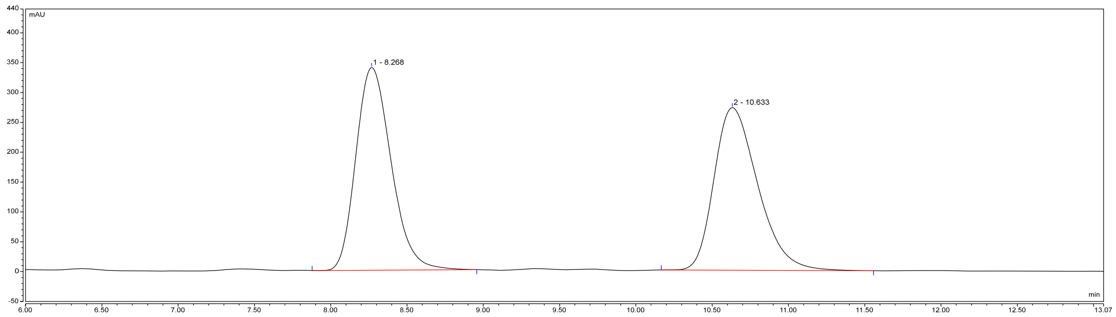

Entry	Retention Time	Area	Height	%Area
1	8.268	90.9800	339.35	50.15
2	10.633	90.4203	272.18	49.85

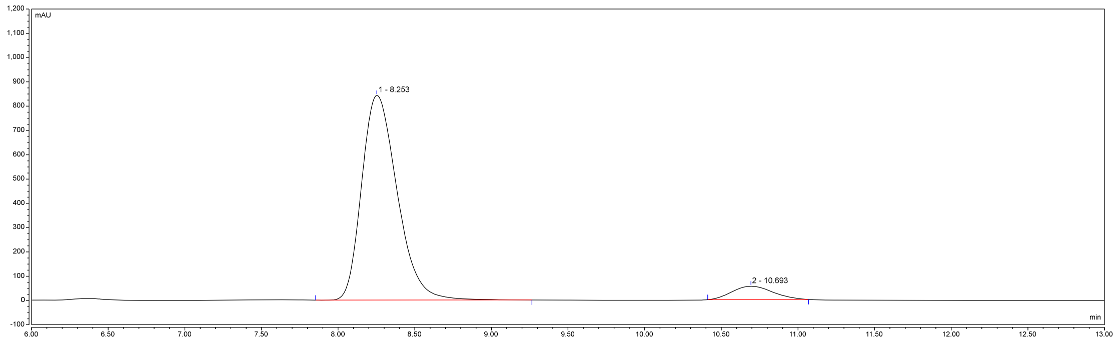

Entry	Retention Time	Area	Height	%Area
1	8.253	226.2281	842.65	93.08
2	10.693	16.8216	55.07	6.92

(*R*)-4,4-dimethyl-7-(pyridin-2-yl)-2-(*o*-tolyl)-1,6-diazaspiro[4.4]nona-1,6-diene (17)

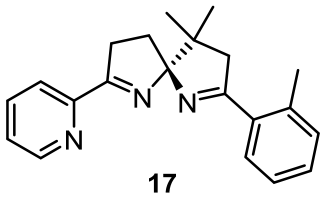

17

White solid; Mp 117.9−118.7 °C; 23.2 mg, 73% yield, 90% ee; $[\alpha]_D^{22}$ −33.7 (*c* 1.0, CH_3OH); **^{1}H NMR** (600 MHz, $CDCl_3$) δ 8.61 – 8.44 (m, 1H), 8.11 (d, *J* = 7.8 Hz, 1H), 7.63 (td, *J* = 7.7, 1.7 Hz, 1H), 7.37 (d, *J* = 7.4 Hz, 1H), 7.22 (m, 1H), 7.20 – 7.15 (m, 1H), 7.12 (m, 2H), 3.29 – 3.15 (m, 3H), 2.66 (d, *J* = 16.1 Hz, 1H), 2.42 (s, 3H), 2.30 – 2.19 (m, 1H), 2.05 (m, 1H), 1.10 (s, 3H), 1.02 (s, 3H); **^{13}C NMR** (151 MHz, $CDCl_3$) δ 177.2, 174.9, 153.7, 149.1, 137.0, 136.2, 135.7, 131.1, 129.2, 128.7, 125.6, 124.8, 122.7, 111.5, 53.7, 44.8, 35.2, 28.5, 25.1, 21.5, 21.2; **HRMS** (ESI) 318.1965 m/z (M + H^+), calc. for $C_{21}H_{24}N_3^+$ 318.1965.

The ee was determined by HPLC analysis: CHIRALPAK IC (4.6 mm i.d. x 250 mm); hexane/2-propanol = 80/20; flow rate 1.0 mL/min; 25 °C; 254 nm; retention time: 8.3 min (minor) and 9.2 min (major).

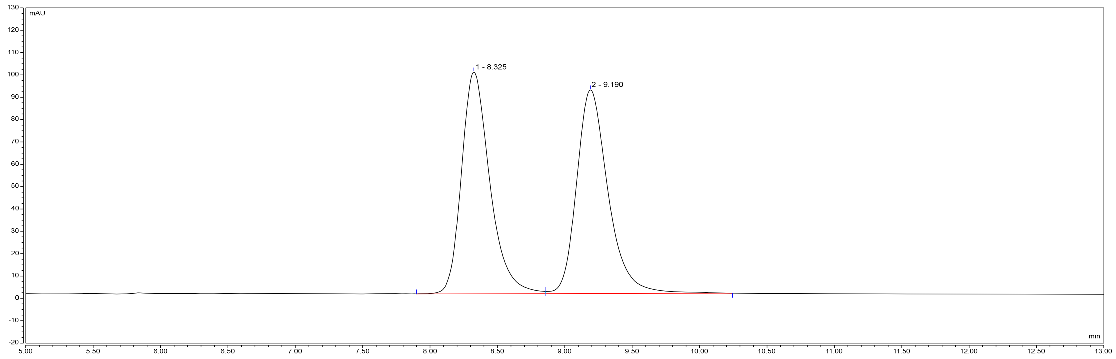

Entry	Retention Time	Area	Height	%Area
1	8.325	24.2549	99.23	49.59
2	9.190	24.6521	91.11	50.41

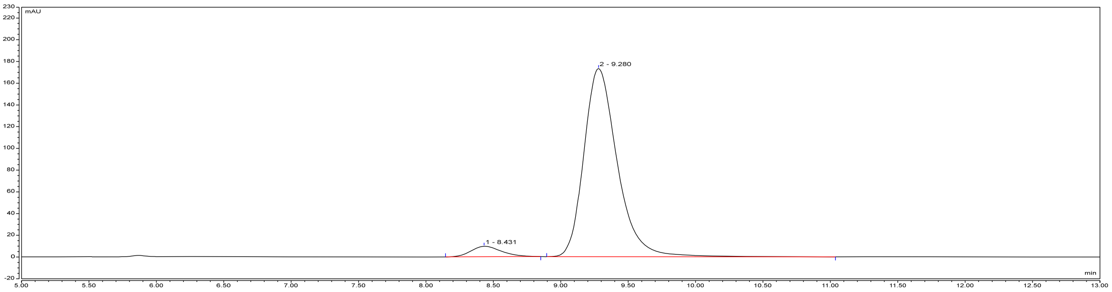

Entry	Retention Time	Area	Height	%Area
1	8.431	2.5219	9.68	4.83
2	9.280	49.7100	173.08	95.17

(*R*)-2-(2-isopropylphenyl)-4,4-dimethyl-7-(pyridin-2-yl)-1,6-diazaspiro[4.4]nona-1,6-diene (18)

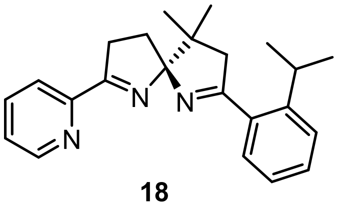

18

White solid; Mp 113.3 - 114.2 °C; 19.4 mg, 56% yield, 86% ee; $[\alpha]_D^{22}$ −32.7 (*c* 1.0, CH_3OH); **^{1}H NMR** (600 MHz, $CDCl_3$) δ 8.75 – 8.50 (m, 1H), 8.19 (d, *J* = 7.9 Hz, 1H), 7.71 (td, *J* = 7.7, 1.7 Hz, 1H), 7.36 – 7.27 (m, 4H), 7.22 – 7.11 (m, 1H), 3.56 – 3.45 (m, 1H), 3.32 – 3.28 (m, 2H), 2.70 (d, *J* = 16.3 Hz, 1H), 2.33 (m, 1H), 2.11 (m, 1H), 1.24 (d, *J* = 6.9 Hz, 3H), 1.20 (d, *J* = 6.9 Hz, 3H), 1.18 (s, 3H), 1.13 (s, 3H); **^{13}C NMR** (151 MHz, $CDCl_3$) δ 177.8, 174.9, 153.7, 149.1, 147.1 136.3, 135.6, 129.3, 128.1, 125.7, 125.5, 124.8, 122.6, 111.6, 54.9, 44.8, 35.1, 29.5, 28.3, 25.0, 24.1, 24.2, 21.5; **HRMS** (ESI) 346.2278 m/z (M + H^+), calc. for $C_{23}H_{28}N_3^+$ 346.2278.

The ee was determined by HPLC analysis: CHIRALPAK IE (4.6 mm i.d. x 250 mm); hexane/2-propanol = 85/15; flow rate 1.0 mL/min; 25 °C; 254 nm; retention time: 7.6 min (minor) and 8.7 min (major).

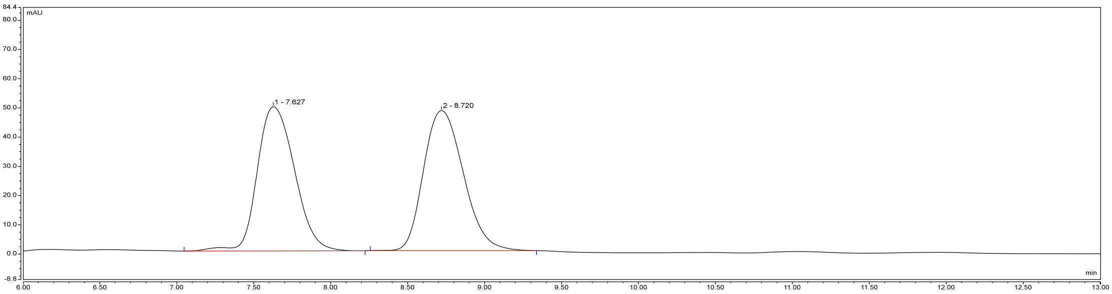

Entry	Retention Time	Area	Height	%Area
1	7.627	13.8441	49.31	49.08
2	8.720	14.3635	47.91	50.92

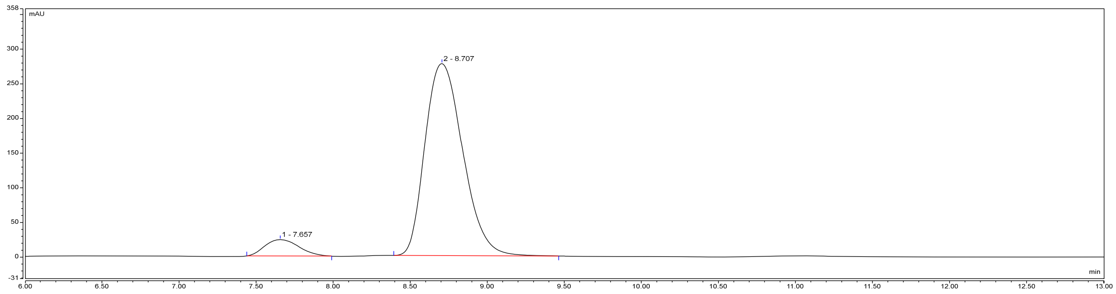

Entry	Retention Time	Area	Height	%Area
1	7.657	6.0145	23.50	7.10
2	8.707	78.6801	276.98	92.90

(*R*)-2-([1,1'-biphenyl]-4-yl)-4,4-dimethyl-7-(pyridin-2-yl)-1,6-diazaspiro[4.4]nona-1,6-diene (19)

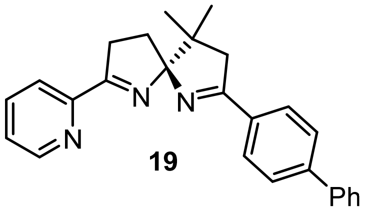

White solid; Mp 173.3−174.1 °C; 23.2 mg, 61% yield, 90% ee; $[\alpha]_D^{22}$ +83.2 (*c* 1.0, CH_3OH); **^{1}H NMR** (600 MHz, $CDCl_3$) δ 8.65 (d, *J* = 4.7 Hz, 1H), 8.18 (d, *J* = 7.9 Hz, 1H), 7.95 (d, *J* = 8.3 Hz, 2H), 7.70 (td, *J* = 7.7, 1.7 Hz, 1H), 7.63 (dd, *J* = 10.1, 7.9 Hz, 4H), 7.44 (t, *J* = 7.7 Hz, 2H), 7.36 (t, *J* = 7.4 Hz, 1H), 7.30 (m, 1H), 3.42 (m, 1H), 3.32 – 3.21 (m, 2H), 2.92 (d, *J* = 15.9 Hz, 1H), 2.38 – 2.27 (m, 1H), 2.17 (m, 1H), 1.19 (s, 3H), 1.09 (s, 3H); **^{13}C NMR** (151 MHz, $CDCl_3$) δ 174.8, 174.3, 153.5, 149.1, 143.5, 140.5, 136.3, 133.9, 128.9, 128.5, 127.8, 127.2, 127.1, 124.8, 122.7, 111.3, 50.1, 45.1, 35.3, 28.5, 25.2, 22.0; **HRMS** (ESI) 380.2121 (M + H^+), calc. for $C_{26}H_{26}N_3^+$ 380.2121.

The ee was determined by HPLC analysis: CHIRALPAK IE (4.6 mm i.d. x 250 mm); hexane/2-propanol = 80/20; flow rate 1.0 mL/min; 25 °C; 254 nm; retention time: 19.7 min (major) and 24.5 min (minor).

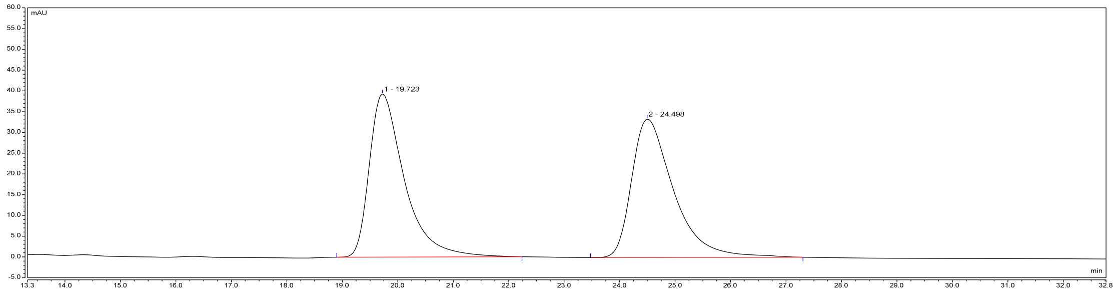

Entry	Retention Time	Area	Height	%Area
1	19.723	28.7281	39.27	49.67
2	24.498	29.1076	33.33	50.33

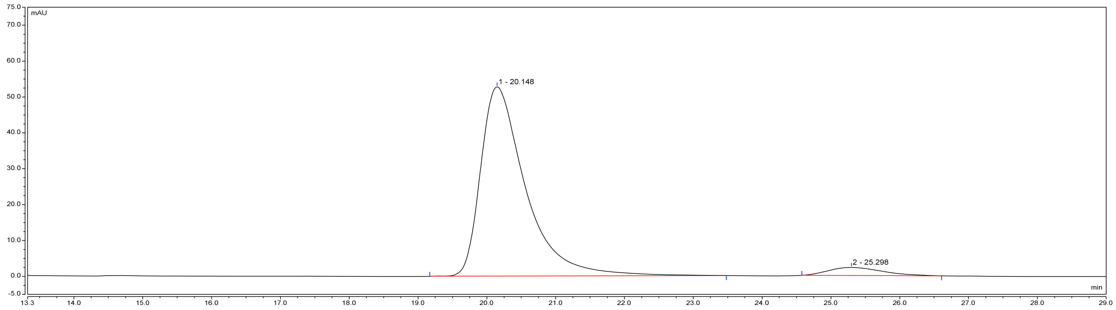

Entry	Retention Time	Area	Height	%Area
1	20.148	39.9711	52.74	95.17
2	25.298	2.0290	2.28	4.83

(*R*)-2-([1,1'-biphenyl]-2-yl)-4,4-dimethyl-7-(pyridin-2-yl)-1,6-diazaspiro [4.4] nona-1,6-diene (20)

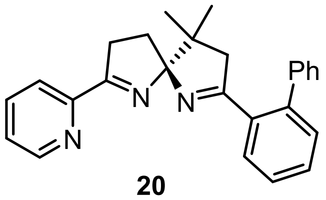

20

White solid; Mp 122.9−123.8 °C; 28.9 mg, 76% yield, 88% ee; $[\alpha]_D^{22}$ +288.3 (*c* 1.0, CH_3OH); **^{1}H NMR** (400 MHz, $CDCl_3$) δ 8.65 (d, *J* = 4.8 Hz, 1H), 8.22 (d, *J* = 7.9 Hz, 1H), 7.74 (t, *J* = 7.7 Hz, 1H), 7.66 (d, *J* = 7.4 Hz, 1H), 7.45 – 7.31 (m, 9H), 3.38 – 3.17 (m, 2H), 2.58 (d, *J* = 16.5 Hz, 1H), 2.31 – 2.22 (m, 1H), 2.10 – 1.98 (m, 2H), 0.94 (s, 3H), 0.85 (s, 3H); ^{13}C NMR (151 MHz, $CDCl_3$) δ 178.5, 174.7, 153.6, 149.1, 141.2, 141.1, 136.2, 130.1, 129.5, 129.3, 128.3, 127.5, 127.4, 124.8, 122.6, 110.8, 52.8, 45.1, 35.1, 28.2, 24.6, 21.8; **HRMS** (ESI) 380.2121 m/z (M + H^+), calc. for $C_{26}H_{26}N_3^+$ 380.2121.

The ee was determined by HPLC analysis: CHIRALPAK IE (4.6 mm i.d. x 250 mm); hexane/2-propanol = 80/20; flow rate 1.0 mL/min; 25 °C; 254 nm; retention time: 10.6 min (minor) and 12.9 min (major).

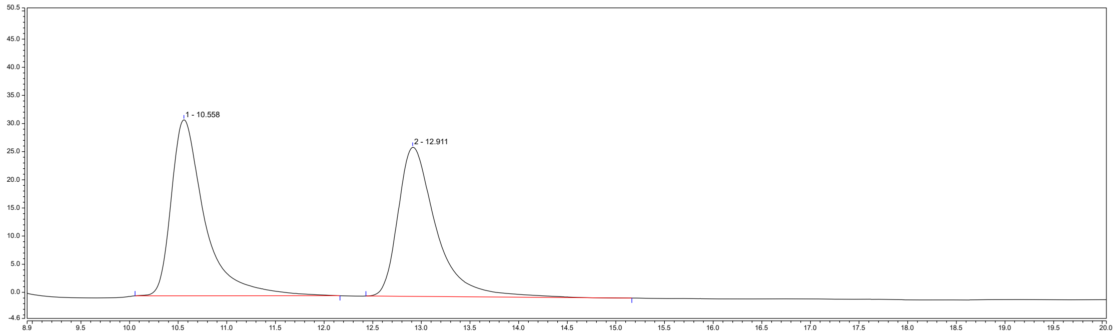

Entry	Retention Time	Area	Height	%Area
1	10.558	12.4890	31.25	50.90
2	12.911	12.0471	26.50	49.10

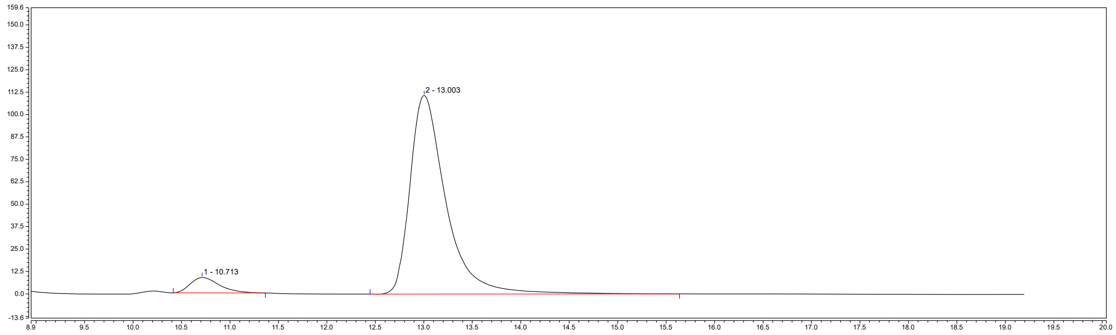

Entry	Retention Time	Area	Height	%Area
1	10.713	3.0019	8.60	5.93
2	13.003	47.5936	110.74	94.07

(*R*)-2-(4-methoxyphenyl)-4,4-dimethyl-7-(pyridin-2-yl)-1,6-diazaspiro[4.4]nona-1,6-diene (21)

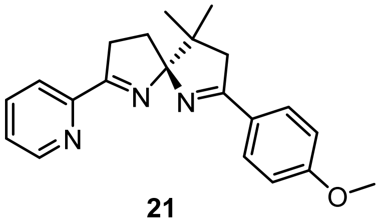

21

White solid; Mp 123.0−123.9 °C; 21.4 mg, 64% yield, 83% ee; $[\alpha]_D^{22}$ −167.4 (*c* 1.0, CH_3OH); **^{1}H NMR** (400 MHz, CD_3OD) δ 8.63 (d, *J* = 4.4 Hz, 1H), 8.11 (d, *J* = 7.9 Hz, 1H), 7.87 (d, *J* = 7.7 Hz, 1H), 7.83 (d, *J* = 8.8 Hz, 2H), 7.46 (dd, *J* = 6.7, 5.5 Hz, 1H), 6.98 (d, *J* = 8.8 Hz, 2H), 3.83 (s, 3H), 3.41 (m, 1H), 3.33 – 3.21 (m, 3H), 2.98 (d, *J* = 16.5 Hz, 1H), 2.30 – 2.15 (m, 2H), 1.16 (s, 3H), 1.07 (s, 3H); **^{13}C NMR** (151 MHz, CD_3OD) δ 177.5, 177.0, 163.7, 153.5, 150.1, 138.0, 130.7, 127.6, 126.5, 123.5, 114.8, 112.1, 55.7, 50.7, 45.3, 36.2, 28.6, 25.3, 21.5; **HRMS** (ESI) 334.1914 m/z (M + H^+), calc. for $C_{21}H_{24}N_3O^+$ 334.1914.

The ee was determined by HPLC analysis: CHIRALPAK IE (4.6 mm i.d. x 250 mm); hexane/2-propanol = 70/30; flow rate 1.0 mL/min; 25 °C; 254 nm; retention time: 13.8 min (major) and 19.2 min (minor).

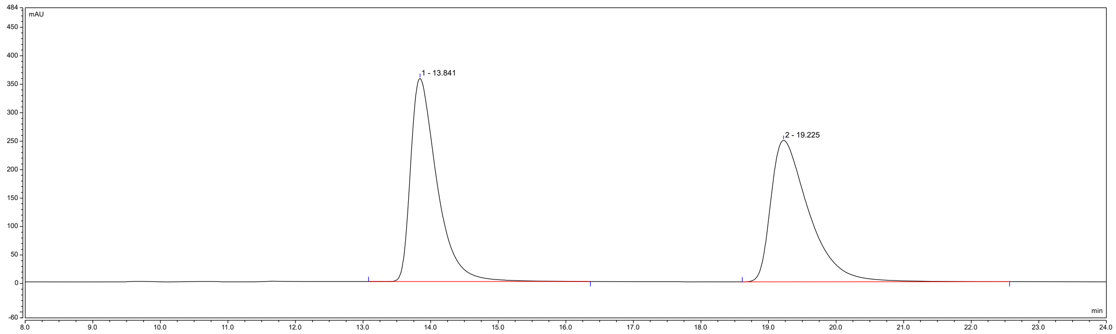

Entry	Retention Time	Area	Height	%Area
1	13.841	161.3460	356.58	49.91
2	19.225	161.9124	248.36	50.09

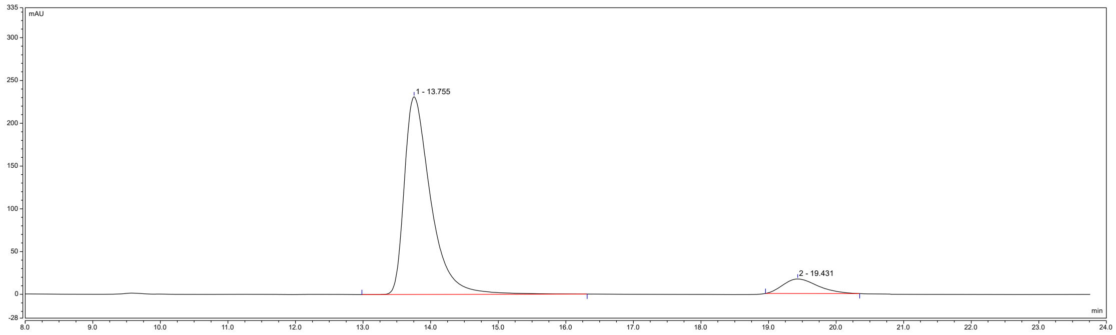

Entry	Retention Time	Area	Height	%Area
1	13.755	104.4886	231.01	91.20
2	19.431	10.0825	16.98	8.80

(*R*)-2-(3-methoxyphenyl)-4,4-dimethyl-7-(pyridin-2-yl)-1,6-diazaspiro[4.4]nona-1,6-diene (22)

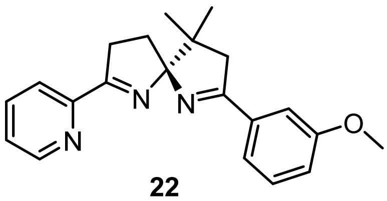

22

White solid; Mp 87.2−88.1 °C; 31.4 mg, 94% yield, 92% ee; $[\alpha]_D^{22}$ −108.4 (*c* 1.0, CH_3OH); **^{1}H NMR** (400 MHz, $CDCl_3$) δ 8.63 (d, *J* = 4.8 Hz, 1H), 8.16 (d, *J* = 7.9 Hz, 1H), 7.67 (m, 1H), 7.45 (m, 1H), 7.38 (d, *J* = 7.7 Hz, 1H), 7.31 – 7.25 (m, 2H), 6.96 (dd, *J* = 8.1, 2.5 Hz, 1H), 3.81 (s, 3H), 3.44 – 3.31 (m, 1H), 3.26 – 3.15 (m, 2H), 2.85 (d, *J* = 16.0 Hz, 1H), 2.28 (m, 1H), 2.13 (m, 1H), 1.14 (s, 3H), 1.05 (s, 3H); **^{13}C NMR** (101 MHz, $CDCl_3$) δ 174.7, 174.4, 159.6, 153.5, 149.1, 136.5, 136.2, 129.3, 124.7, 122.6, 120.6, 117.2, 112.2, 111.2, 77.5, 77.1, 76.8, 55.4, 50.2, 45.0, 35.2, 28.5, 25.0, 22.0; **HRMS** (ESI) 334.1914 m/z (M + H^+), calc. for $C_{21}H_{24}N_3O^+$ 334.1914.

The ee was determined by HPLC analysis: CHIRALPAK IE (4.6 mm i.d. x 250 mm); hexane/2-propanol = 80/20; flow rate 1.0 mL/min; 25 °C; 254 nm; retention time: 15.8 min (major) and 24.8 min (minor).

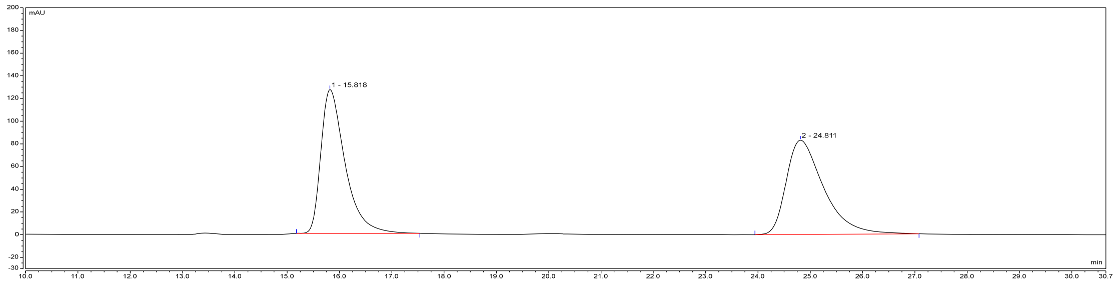

Entry	Retention Time	Area	Height	%Area
1	15.818	67.8241	126.64	49.66
2	24.811	68.7576	83.09	50.34

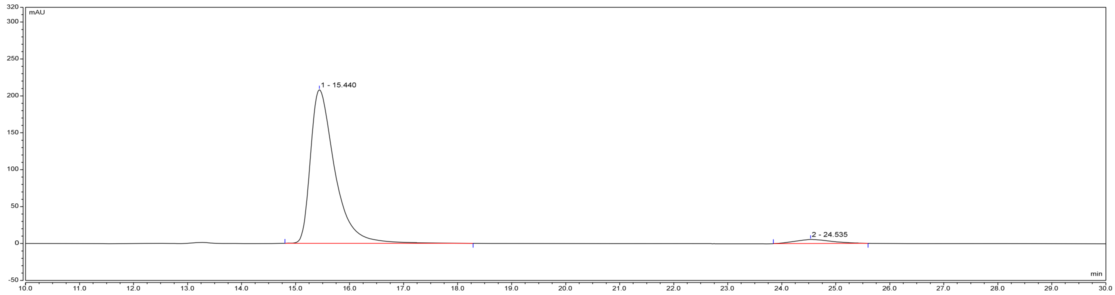

Entry	Retention Time	Area	Height	%Area
1	15.440	108.1700	207.79	96.30
2	24.535	4.1587	5.38	3.70

(*R*)-2-(3-(allyloxy)phenyl)-4,4-dimethyl-7-(pyridin-2-yl)-1,6-diazaspiro[4.4]nona-1,6-diene (23)

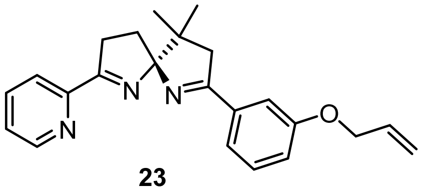

23

White solid; Mp 129.3−130.1 °C; 18.7 mg, 52% yield, 87% ee; $[\alpha]_D^{22}$ −38.1 (*c* 1.0, CH_3OH); **^{1}H NMR** (600 MHz, $CDCl_3$) δ 8.63 (d, *J* = 4.8 Hz, 1H), 8.16 (d, *J* = 7.9 Hz, 1H), 7.68 (td, *J* = 7.7, 1.7 Hz, 1H), 7.47 (s, 1H), 7.40 (d, *J* = 7.6 Hz, 1H), 7.28 (m, 2H), 6.98 (dd, *J* = 8.1, 2.4 Hz, 1H), 6.04 (m, 1H), 5.40 (dd, *J* = 17.3, 1.5 Hz, 1H), 5.26 (dd, *J* = 10.5, 1.3 Hz, 1H), 4.56 (d, *J* = 5.3 Hz, 2H), 3.38 (m, 1H), 3.23 (m, 2H), 2.85 (d, *J* = 15.9 Hz, 1H), 2.29 (m, 1H), 2.14 (m, 1H), 1.15 (s, 3H), 1.06 (s, 3H); **^{13}C NMR** (151 MHz, $CDCl_3$) δ 174.8, 174.5, 158.7, 153.5, 149.1, 136.4, 136.2, 133.2, 129.3, 124.8, 122.7, 120.8, 117.8, 117.7, 113.3, 111.2, 68.9, 50.2, 45.0, 35.3, 28.5, 25.1, 22.0; **HRMS** (ESI) 360.2070 m/z (M + H^+), calc. for $C_{23}H_{26}N_3O^+$ 360.2070.

The ee was determined by HPLC analysis: CHIRALPAK IE (4.6 mm i.d. x 250 mm); hexane/2-propanol = 80/20; flow rate 1.0 mL/min; 25 °C; 254 nm; retention time: 12.2 min (major) and 25.1 min (minor).

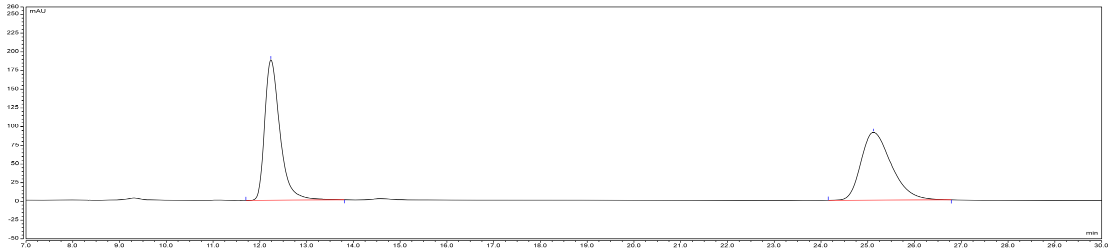

Entry	Retention Time	Area	Height	%Area
1	12.237	70.7692	187.85	50.95
2	25.123	68.1214	90.70	49.05

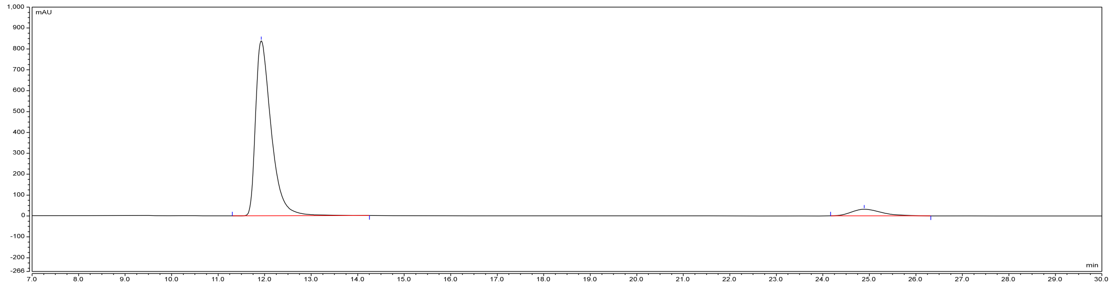

Entry	Retention Time	Area	Height	%Area
1	11.927	324.5224	838.10	93.79
2	24.890	21.4765	30.67	6.21

(*R*)-4,4-dimethyl-7-(pyridin-2-yl)-2-(4-(trifluoromethoxy)phenyl)-1,6-diazaspiro[4.4] nona-1,6-diene (24)

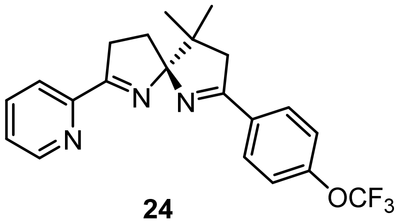

24

White solid; Mp 118.0−119.0 °C; 16.7 mg, 43% yield, 87% ee; $[\alpha]_D^{22}$ +47.3 (*c* 1.0, CH_3OH); **^{1}H NMR** (600 MHz, $CDCl_3$) δ 8.64 (d, *J* = 4.6 Hz, 1H), 8.15 (d, *J* = 7.9 Hz, 1H), 7.90 (d, *J* = 8.7 Hz, 2H), 7.68 (td, *J* = 7.7, 1.6 Hz, 1H), 7.34 – 7.27 (m, 1H), 7.22 (d, *J* = 8.3 Hz, 2H), 3.38 (m, 1H), 3.29 – 3.16 (m, 2H), 2.84 (d, *J* = 15.9 Hz, 1H), 2.29 (m, 1H), 2.20 – 2.03 (m, 1H), 1.16 (s, 3H), 1.06 (s, 3H); **^{13}C NMR** (151 MHz, $CDCl_3$) δ 175.1, 173.3, 153.4, 151.0, 149.2, 136.3, 133.6, 129.6, 124.8, 123.0, 120.64, 120.5 (q, *J* = 257.8 Hz), 111.4, 50.1, 45.2, 35.3, 28.4, 25.1, 21.9; **^{19}F NMR** (565 MHz, $CDCl_3$) δ -57.7; **HRMS** (ESI) 388.1631 m/z (M + H^+), calc. for $C_{21}H_{21}F_3N_3O^+$ 388.1631.

The ee was determined by HPLC analysis: CHIRALPAK IE (4.6 mm i.d. x 250 mm); hexane/2-propanol = 80/20; flow rate 1.0 mL/min; 25 °C; 254 nm; retention time: 6.2 min (major) and 7.9 min (minor).

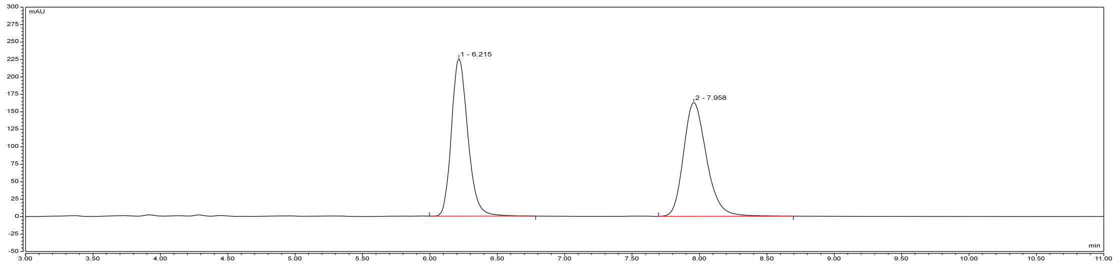

Entry	Retention Time	Area	Height	%Area
1	6.215	31.7399	225.18	49.68
2	7.958	32.1456	162.76	50.32

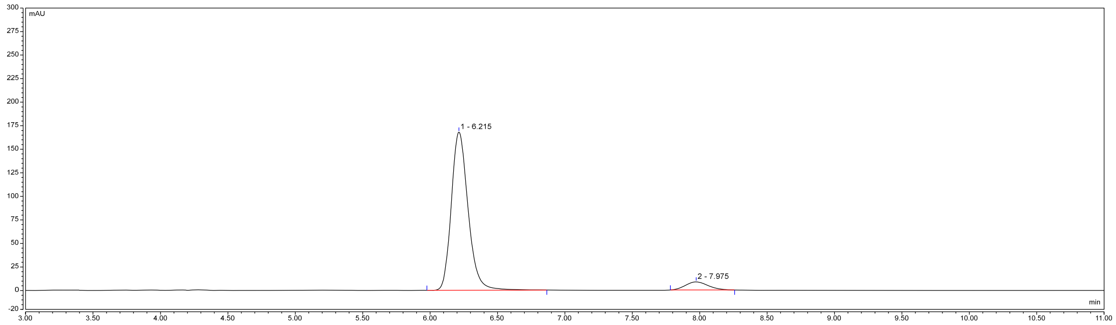

Entry	Retention Time	Area	Height	%Area
1	6.215	24.2024	167.93	93.73
2	7.975	1.6196	8.47	6.27

(*R*)-2-(benzo[*d*][1,3]dioxol-5-yl)-4,4-dimethyl-7-(pyridin-2-yl)-1,6-diazaspiro[4.4]nona-1,6-diene (25)

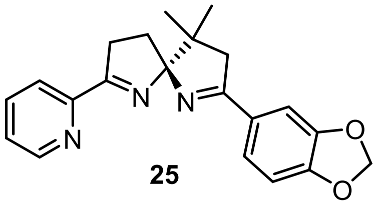

25

White solid; Mp 146.7−147.5 °C; 29.6 mg, 85% yield, 86% ee; $[\alpha]_{D}^{22}$ −48.7 (*c* 1.0, CH_3OH); **^{1}H NMR** (400 MHz, CD_3OD) δ 8.70 – 8.50 (m, 1H), 8.12 (d, J = 7.9 Hz, 1H), 7.93 – 7.81 (m, 1H), 7.48 (m, 1H), 7.41 (m, 1H), 7.41 – 7.37 (m, 1H), 6.89 (d, *J* = 8.0 Hz, 1H), 6.02 (m, 2H), 3.40 (m, 1H), 3.30 (d, *J* = 3.1 Hz, 1H), 3.27 (t, *J* = 5.9 Hz, 1H), 2.97 (d, *J* = 16.5 Hz, 1H), 2.25 (dd, *J* = 8.2, 6.4 Hz, 2H), 1.16 (s, 3H), 1.08 (s, 3H); **^{13}C NMR** (151 MHz, CD_3OD) δ 177.3, 177.1, 153.5, 151.8, 150.1, 149.4, 138.0, 129.4, 126.5, 124.7, 123.5, 112.1, 108.8, 107.9, 102.9, 50.9, 45.4, 36.2, 28.6, 25.3, 21.5; **HRMS** (ESI) 348.1706 m/z (M +H$^+$), calc. for $C_{21}H_{21}F_3N_3O^+$ 348.1707.

The ee was determined by HPLC analysis: CHIRALPAK IE (4.6 mm i.d. x 250 mm); hexane/2-propanol = 70/30; flow rate 1.0 mL/min; 25 °C; 254 nm; retention time:14.1 min (major) and 22.5 min (minor).

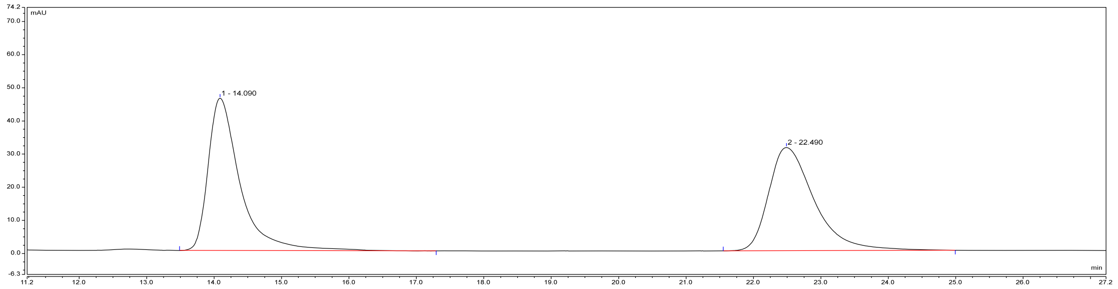

Entry	Retention Time	Area	Height	%Area
1	14.090	25.0992	45.92	50.77
2	22.490	24.3365	31.18	49.23

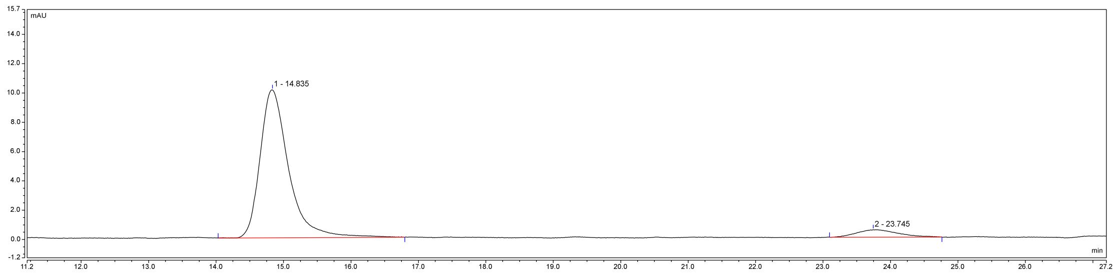

Entry	Retention Time	Area	Height	%Area
1	14.835	4.9381	10.11	93.22
2	23.745	0.3589	0.52	6.78

(*R*)-4,4-dimethyl-2-(naphthalen-2-yl)-7-(pyridin-2-yl)-1,6-diazaspiro[4.4]nona-1,6-diene (26)

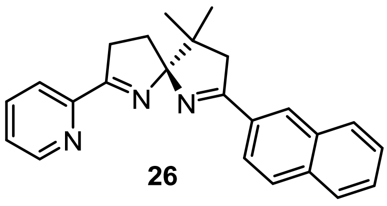

26

White solid; Mp 155.9−156.8 °C; 31.5 mg, 89% yield, 87% ee; $[\alpha]_D^{22}$ −29.6 (*c* 1.0, CH_3OH); **^{1}H NMR** (600 MHz, CD_3OD) δ 8.64 (d, *J* = 4.6 Hz, 1H), 8.34 (s, 1H), 8.13 (d, *J* = 8.0 Hz, 1H), 8.03 (dd, *J* = 8.6, 1.5 Hz, 1H), 7.98 (d, *J* = 7.6 Hz, 1H), 7.92 – 7.88 (m, 2H), 7.86 (dd, *J* = 7.8, 1.5 Hz, 1H), 7.55 (m, 2H), 7.50 – 7.42 (m, 1H), 3.49 – 3.40 (m, 2H), 3.31 – 3.27 (m, 1H), 3.17 (d, *J* = 16.4 Hz, 1H), 2.30 (m, 2H), 1.22 (s, 3H), 1.13 (s, 3H); **^{13}C NMR** (151 MHz, CD_3OD) δ 178.1, 177.3, 153.5, 150.2, 138.1, 136.1, 134.2, 132.6, 130.3, 129.8, 129.2, 128.6,128.5, 127.6, 126.6, 124.8, 123.5, 112.4, 50.9, 45.4, 36.3, 28.7, 25.3, 21.5; **HRMS** (ESI) 354.1965 m/z (M + H^+), calc. for $C_{24}H_{24}N_3^+$ 354.1965.

The ee was determined by HPLC analysis: CHIRALPAK IE (4.6 mm i.d. x 250 mm); hexane/2-propanol = 80/20; flow rate 1.0 mL/min; 25 °C; 254 nm; retention time: 14.6 min (major) and 20.0 min (minor).

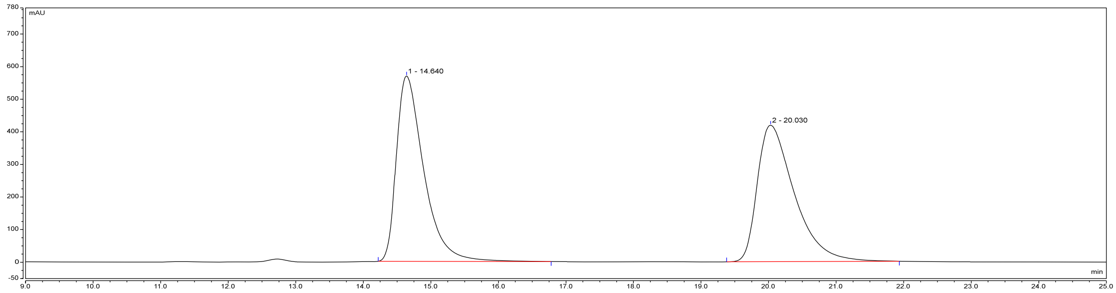

Entry	Retention Time	Area	Height	%Area
1	14.640	266.4490	568.46	50.06
2	20.030	265.8514	418.78	49.94

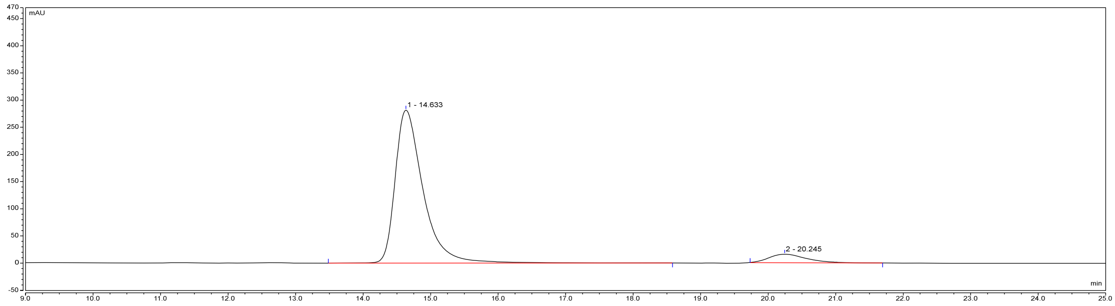

Entry	Retention Time	Area	Height	%Area
1	14.633	134.8512	281.58	93.13
2	20.245	9.9473	15.63	6.87

(*R*)-4,4-dimethyl-2-(naphthalen-1-yl)-7-(pyridin-2-yl)-1,6-diazaspiro[4.4]nona-1,6-diene (27)

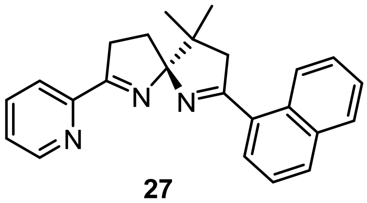

27

White solid; Mp 147.1−147.9 °C; 30.1 mg, 85% yield, 95% ee; $[\alpha]_D^{22}$ −41.6 (*c* 1.0, CH_3OH); **^{1}H NMR** (400 MHz, $CDCl_3$) δ 8.86 (d, *J* = 8.2 Hz, 1H), 8.66 (dd, *J* = 4.8, 0.7 Hz, 1H), 8.25 (d, *J* = 7.5 Hz, 1H), 7.90 – 7.79 (m, 2H), 7.77 – 7.66 (m, 2H), 7.56 – 7.41 (m, 3H), 7.32 (m, 1H), 3.57 (d, *J* = 16.0 Hz, 1H), 3.48 – 3.29 (m, 2H), 2.93 (d, *J* = 16.0 Hz, 1H), 2.52 – 2.42 (m, 1H), 2.21 (m, 1H), 1.24 (s, 3H), 1.19 (s, 3H); **^{13}C NMR** (151 MHz, $CDCl_3$) δ 176.4, 175.1 153.6, 149.2, 136.3, 134.0, 133.0, 131.0, 130.5, 128.3, 127.5, 127.2, 126.5, 126.1, 124.8, 124.8, 122.8, 112.1, 53.9, 44.6, 35.3, 28.6, 25.2, 21.6; **HRMS** (ESI) 354.1965 m/z (M + H^+), calc. for $C_{24}H_{24}N_3^+$ 354.1965.

The ee was determined by HPLC analysis: CHIRALPAK IE (4.6 mm i.d. x 250 mm); hexane/2-propanol = 95/5; flow rate 1.0 mL/min; 25 °C; 254 nm; retention time: 21.6 min (minor) and 23.9 min (major).

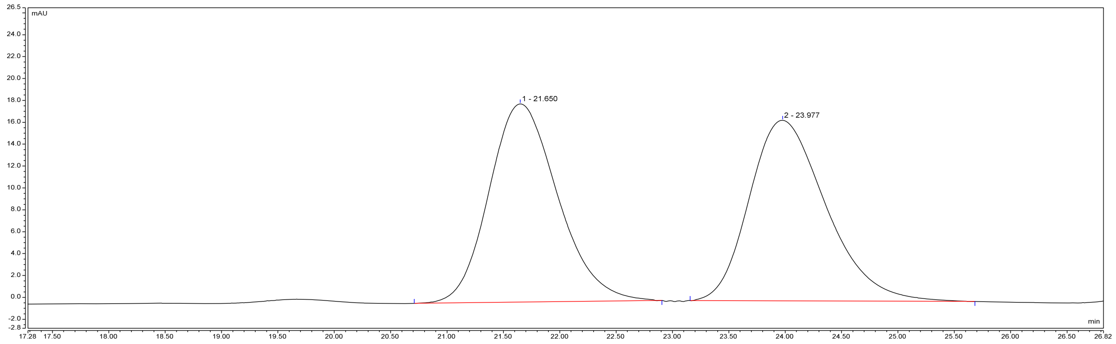

Entry	Retention Time	Area	Height	%Area
1	21.650	12.7943	18.12	49.59
2	23.977	13.0052	16.49	50.41

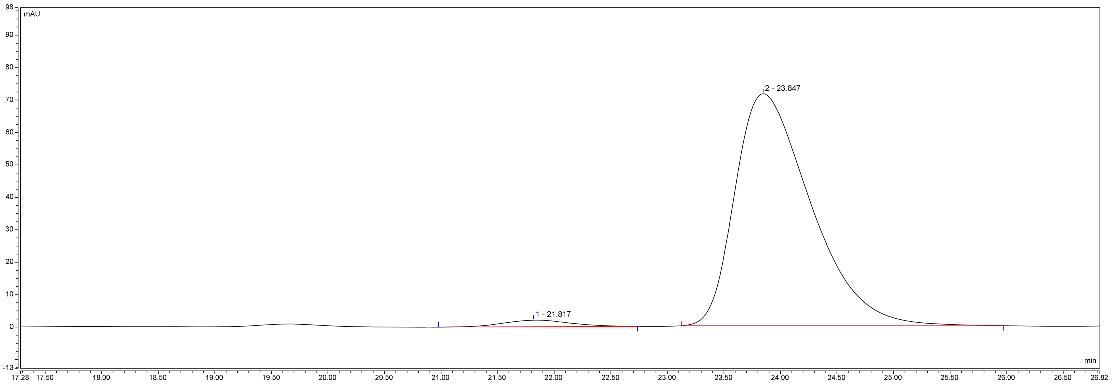

Entry	Retention Time	Area	Height	%Area
1	21.817	1.3686	2.03	2.40
2	23.847	55.6272	71.54	97.60

(*R*)-2-(benzo[*b*]thiophen-5-yl)-4,4-dimethyl-7-(pyridin-2-yl)-1,6-diazaspiro[4.4]nona-1,6-diene (28)

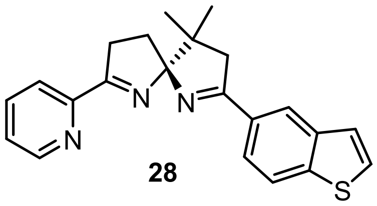

28

Yellow solid; Mp 117.2−118.0 °C; 20.9 mg, 58% yield, 90% ee; $[\alpha]_D^{22}$ −49.6 (*c* 1.0, CH_3OH); **^{1}H NMR** (600 MHz, CD_3OD) δ 8.68 – 8.60 (m, 1H), 8.33 (s, 1H), 8.12 (dd, *J* = 8.0, 0.9 Hz, 1H), 7.96 (dd, *J* = 8.5, 2.6 Hz, 1H), 7.90 (dd, *J* = 8.5, 1.2 Hz, 1H), 7.89 – 7.83 (m, 1H), 7.64 (dd, *J* = 5.4, 2.6 Hz, 1H), 7.46 (dd, *J* = 5.3, 1.6 Hz, 2H), 3.48 – 3.38 (m, 2H), 3.31 – 3.26 (m, 1H), 3.10 (dd, *J* = 16.4, 4.9 Hz, 1H), 2.28 (m, 2H), 1.20 (s, 3H), 1.11 (s, 3H); **^{13}C NMR** (151 MHz, CD_3OD) δ 178.3, 177.2, 153.5, 150.2, 143.9, 141.0, 138.0, 131.6, 128.7, 126.6, 125.2, 124.8, 123.9, 123.5, 123.4, 112.3, 51.0, 45.4, 36.2, 28.7, 25.3, 21.5; **HRMS** (ESI) 360.1528 m/z (M + H^+), calc. for $C_{22}H_{22}N_3S^+$ 360.1529.

The ee was determined by HPLC analysis: CHIRALPAK IE (4.6 mm i.d. x 250 mm); hexane/2-propanol = 80/20; flow rate 1.0 mL/min; 25 °C; 254 nm; retention time:16.4 min (major) and 22.7 min (minor).

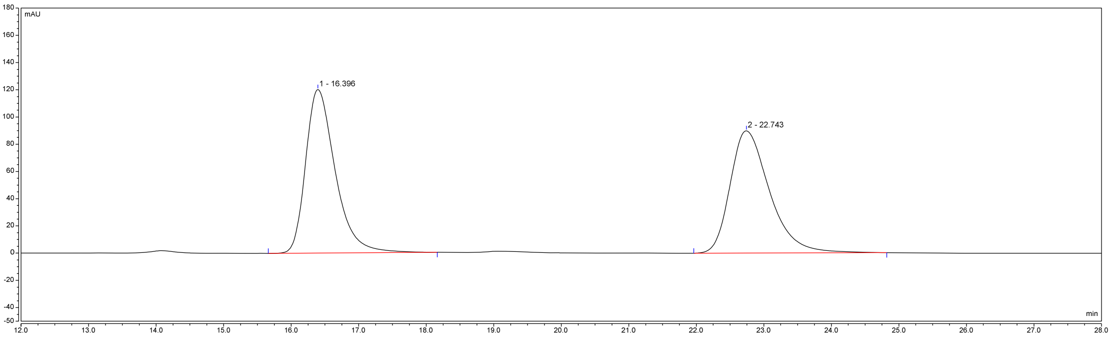

Entry	Retention Time	Area	Height	%Area
1	16.396	60.8735	120.13	50.05

2	22.743	60.7590	89.83	49.95

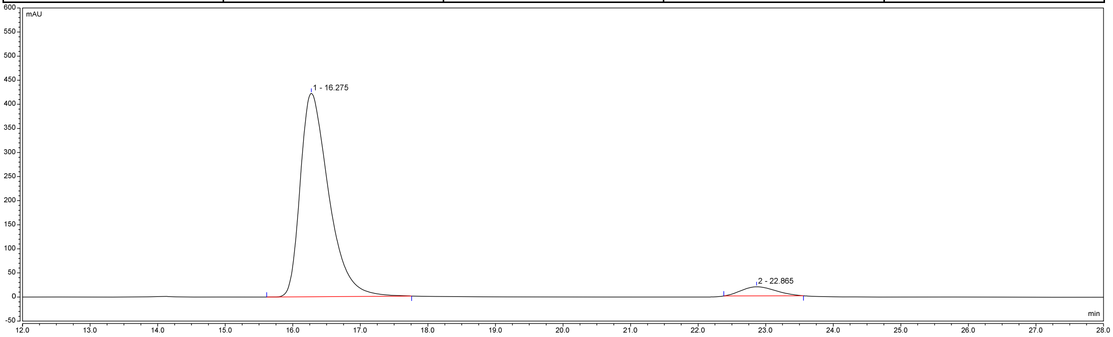

Entry	Retention Time	Area	Height	%Area
1	16.275	422.31	205.9780	94.88
2	22.865	18.89	11.1160	5.12

(*R*)-4,4-dimethyl-7-(pyridin-2-yl)-2-(thiophen-3-yl)-1,6-diazaspiro[4.4]nona-1,6-diene (29)

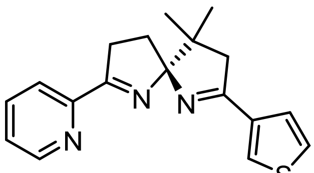

29

White solid; Mp 147.5−148.3 °C; 23.3 mg, 75% yield, 95% ee; $[\alpha]_D^{22}$ −292.0 (*c* 1.0, CH_3OH); **^{1}H NMR** (400 MHz, $CDCl_3$) δ 8.64 (d, *J* = 3.8 Hz, 1H), 8.17 (d, *J* = 7.8 Hz, 1H), 7.78 – 7.64 (m, 2H), 7.59 (d, *J* = 4.2 Hz, 1H), 7.30 (m, 2H), 3.37 (m, 1H), 3.22 (d, *J* = 15.7 Hz, 2H), 2.81 (d, *J* = 15.8 Hz, 1H), 2.29 (m, 1H), 2.19 – 2.06 (m, 1H), 1.15 (s, 3H), 1.07 (s, 3H); **^{13}C NMR** (151 MHz, CD_3OD) δ 175.9, 172.1, 152.3, 148.9, 137.0, 136.8, 129.9, 126.5, 125.8, 125.3, 122.3, 110.9, 50.3, 44.0, 35.0, 27.4, 24.1, 20.2; **HRMS** (ESI) 310.1372 m/z (M + H^+), calc. for $C_{18}H_{20}N_3S^+$ 310.1372.

The ee was determined by HPLC analysis: CHIRALPAK IE (4.6 mm i.d. x 250 mm); hexane/2-propanol = 80/20; flow rate 1.0 mL/min; 25 °C; 254 nm; retention time: 17.9 min (major) and 27.4 min (minor).

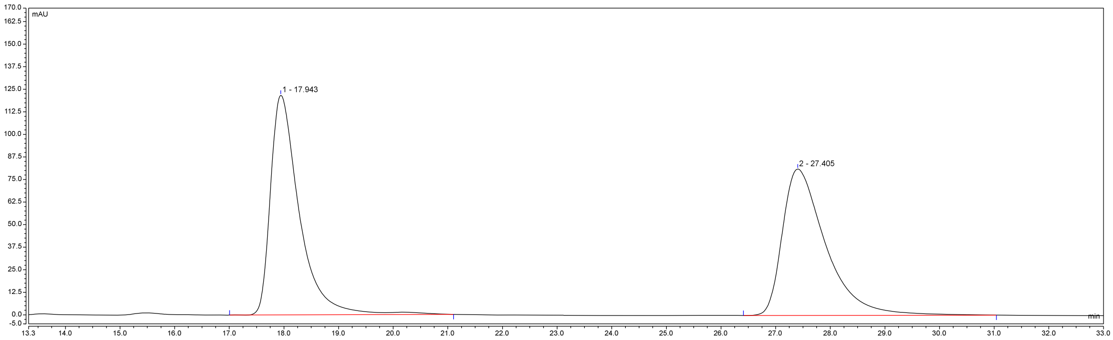

Entry	Retention Time	Area	Height	%Area
1	17.943	74.7485	121.69	50.12
2	27.405	74.3949	81.12	49.88

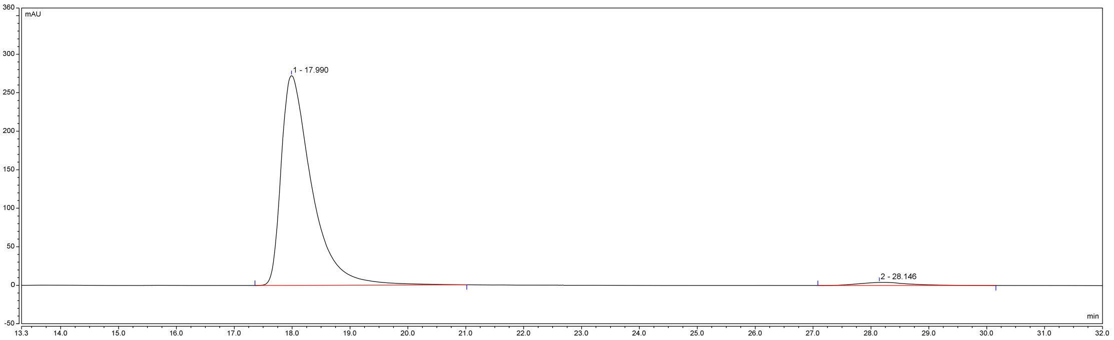

Entry	Retention Time	Area	Height	%Area
1	17.990	272.29	166.2436	97.63
2	28.146	4.01	4.0311	2.37

(*R*)-2-(2-bromophenyl)-7-(3-fluoropyridin-2-yl)-4,4-dimethyl-1,6-diazaspiro[4.4]nona-1,6-diene (30)

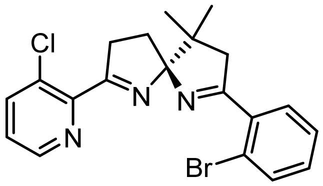

30

Yellow solid; Mp 87.3−88.1 °C; 26.6 mg, 64% yield, 92% ee; $[\alpha]_D^{22}$ –30.3 (*c* 1.0, CH_3OH); **^{1}H NMR** (600 MHz, $CDCl_3$) δ 8.51 (d, *J* = 5.3 Hz, 1H), 8.18 (dd, *J* = 48.8, 1.9 Hz, 1H), 7.62 – 7.51 (m, 1H), 7.54 – 7.44 (m, 1H), 7.32 – 7.27 (m, 2H), 7.20 (td, *J* = 7.8, 1.7 Hz, 1H), 3.34 (d, *J* = 16.4 Hz, 1H), 3.31 – 3.19 (m, 2H), 2.98 (d, *J* = 16.4 Hz, 1H), 2.32 (m, 1H), 2.11 (m, 1H), 1.16 (s, 3H), 1.14 (s, 3H); ^{13}C **NMR** (151 MHz, $CDCl_3$) δ 177.4, 174.4, 154.9, 149.9, 144.4, 137.8, 133.3, 130.7, 130.4, 127.4, 125.0, 122.9, 121.3, 110.9, 53.3, 45.6, 35.2, 28.2, 24.8, 21.6; **HRMS** (ESI) 416.0521 m/z (M + H^+), calc. for $C_{20}H_{20}BrClN_3^+$ 416.0524. The ee was determined by HPLC analysis: CHIRALPAK IE (4.6 mm i.d. x 250 mm); hexane/2-propanol = 80/20; flow rate 1.0 mL/min; 25 °C; 254 nm; retention time: 7.3 min (minor) and 9.1 min (major).

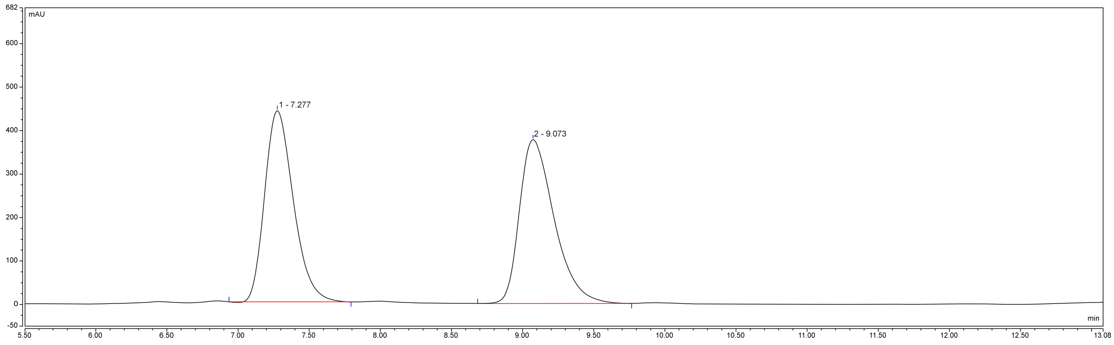

Entry	Retention Time	Area	Height	%Area
1	7.277	102.8317	439.58	49.12
2	9.073	106.5133	376.68	50.88

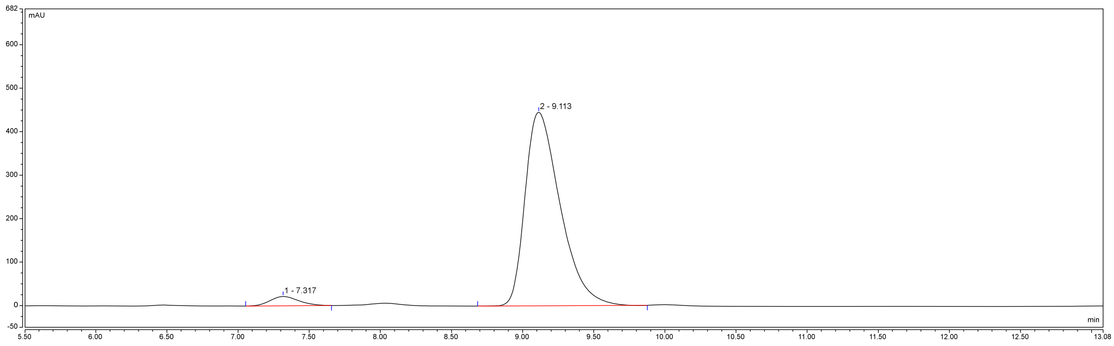

Entry	Retention Time	Area	Height	%Area
1	7.317	4.9989	21.38	3.79
2	9.113	126.9549	445.04	96.21

(*R*)-2-(2-bromophenyl)-7-(4-fluoropyridin-2-yl)-4,4-dimethyl-1,6-diazaspiro[4.4]nona-1,6-diene (31)

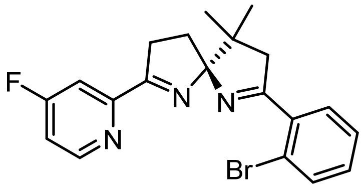

31

Yellow solid; Mp 120.7−121.5 °C; 22.4 mg, 56% yield, 92% ee; $[\alpha]_D^{22}$ −42.3 (*c* 1.0, CH_3OH); **^{1}H NMR** (600 MHz, CD_3OD) δ 8.66 (dd, *J* = 8.3, 5.7 Hz, 1H), 7.92 (dd, *J* = 9.8, 2.5 Hz, 1H), 7.67 (dd, *J* = 8.0, 0.6 Hz, 1H), 7.46 – 7.39 (m, 2H), 7.36 – 7.34 (m, 1H), 7.33 – 7.29 (m, 1H), 3.37 (d, *J* = 16.9 Hz, 1H), 3.30 (d, *J* = 8.6 Hz, 2H), 3.01 (d, *J* = 16.9 Hz, 1H), 2.28 (t, *J* = 7.3 Hz, 2H), 1.21 (s, 3H), 1.20 (s, 3H); **^{13}C NMR** (101 MHz, CD_3OD) δ 180.5, 176.7(d, *J* = 3.6 Hz), 170.2 (d, *J* = 261.7 Hz), 156.9 (d, *J* = 7.0 Hz), 153.0 (d, *J* = 7.2 Hz), 138.2, 134.2, 132.1, 130.7, 128.6, 121.4, 114.1 (d, *J* = 17.1 Hz), 111.9, 110.8 (d, *J* = 18.4 Hz), 54.3, 46.3, 36.1, 28.6, 24.9, 21.4; **^{19}F NMR** (565 MHz, CD_3OD) δ -103.67; **HRMS** (ESI) 400.0819 m/z (M + H^+), calc. for $C_{20}H_{20}BrFN_3^+$ 400.0819.

The ee was determined by HPLC analysis: CHIRALPAK IE (4.6 mm i.d. x 250 mm); hexane/2-propanol = 80/20; flow rate 1.0 mL/min; 25 °C; 254 nm; retention time: 7.1 min (minor) and 8.5 min (major).

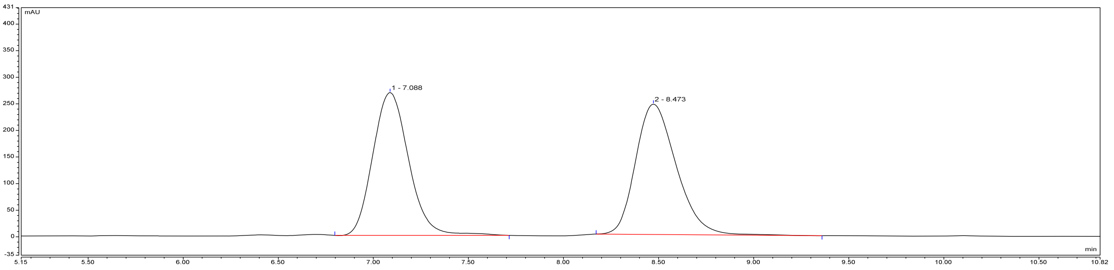

Entry	Retention Time	Area	Height	%Area
1	7.088	58.9000	268.46	49.22

2	8.473	60.7684	245.01	50.78

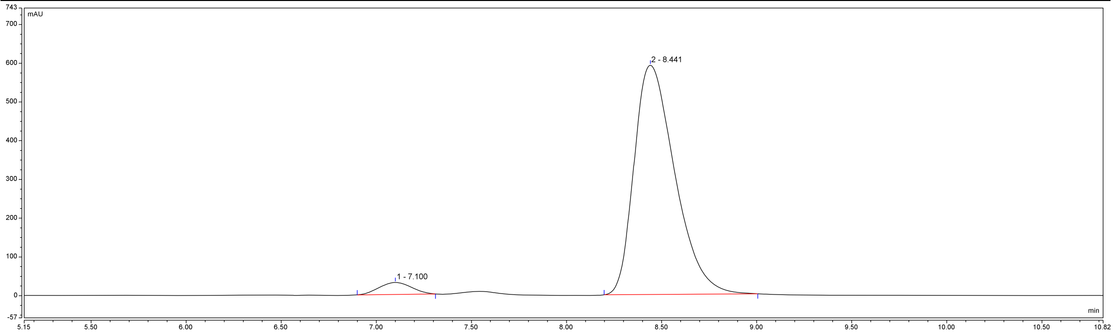

Entry	Retention Time	Area	Height	%Area
1	7.100	6.1243	30.97	4.00
2	8.441	147.0176	592.42	96.00

(*R*)-2-(2-bromophenyl)-7-(4-chloropyridin-2-yl)-4,4-dimethyl-1,6-diazaspiro[4.4]nona-1,6-diene (32)

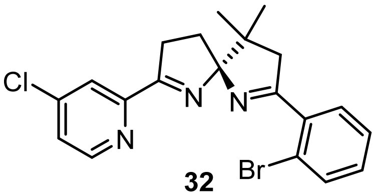

32

Yellow solid; Mp 126.0−126.8 °C; 29.9 mg, 72% yield, 92% ee; $[\alpha]_D^{22}$ −16.8 (*c* 1.0, CH_3OH); **^{1}H NMR** (400 MHz, $CDCl_3$) δ 8.52 (d, *J* = 5.3 Hz, 1H), 8.23 (d, *J* = 1.9 Hz, 1H), 7.57 (dd, *J* = 7.9, 0.8 Hz, 1H), 7.50 (dd, *J* = 7.6, 1.6 Hz, 1H), 7.35 – 7.27 (m, 2H), 7.21 (td, *J* = 7.7, 1.7 Hz, 1H), 3.39 – 3.32 (m, 1H), 3.26 (m, 2H), 2.99 (d, *J* = 16.4 Hz, 1H), 2.33 (m, 1H), 2.12 (m, 1H), 1.16 (s, 3H), 1.14 (s, 3H); **^{13}C NMR** (101 MHz, $CDCl_3$) δ 177.5, 174.4, 154.9, 150.0, 144.5, 137.8, 133.4, 130.7, 130.5, 127.4, 125.0, 122.9, 121.3, 110.9, 53.3, 45.7, 35.2, 28.2, 24.8, 21.6; **HRMS** (ESI) 416.0520 m/z (M + H$^+$), calc. for $C_{20}H_{20}BrClN_3^+$ 416.0524.

The ee was determined by HPLC analysis: CHIRALPAK IE (4.6 mm i.d. x 250 mm); hexane/2-propanol = 80/20; flow rate 1.0 mL/min; 25 °C; 254 nm; retention time: 7.0 min (minor) and 8.6 min (major).

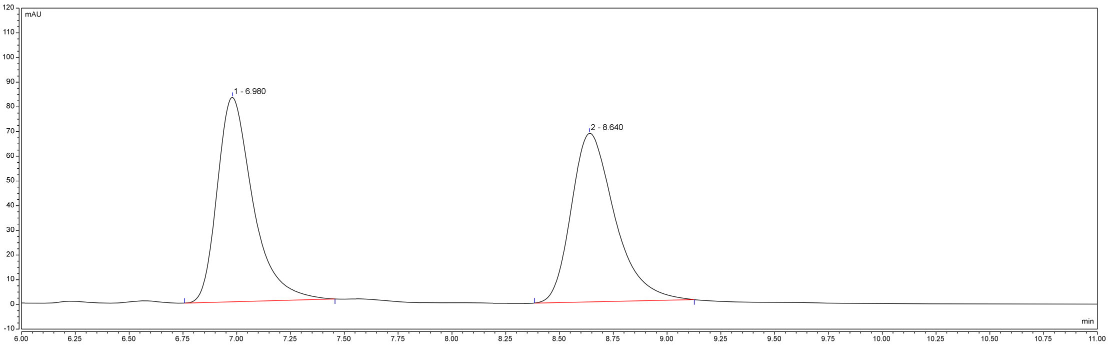

Entry	Retention Time	Area	Height	%Area
1	6.980	15.9218	82.76	49.85

2	8.640	16.0206	68.27	50.15

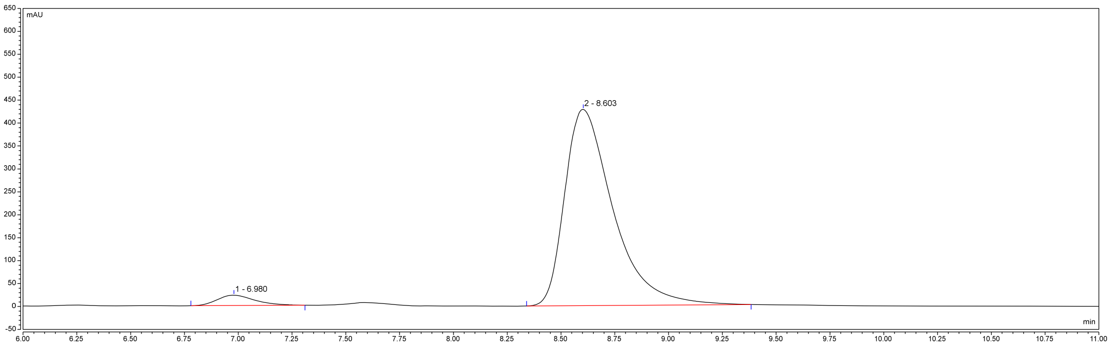

Entry	Retention Time	Area	Height	%Area
1	6.980	4.5374	22.63	3.91
2	8.603	111.5857	427.89	96.09

(*R*)-2-(2-bromophenyl)-4,4-dimethyl-7-(4-methylpyridin-2-yl)-1,6-diazaspiro[4.4]nona-1,6-diene (33)

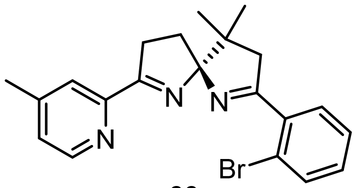

33

White solid; Mp 96.4−97.2 °C; 28.1 mg, 71% yield, 93% ee; $[\alpha]_D^{22}$ −20.3 (*c* 1.0, CH_3OH); **^{1}H NMR** (600 MHz, CD_3OD) δ 8.49 (d, *J* = 5.0 Hz, 1H), 8.01 (s, 1H), 7.67 (d, *J* = 8.0 Hz, 1H), 7.46 (dd, *J* = 7.6, 1.7 Hz, 1H), 7.42 (m, 1H), 7.34 (m, 2H), 3.37 (d, *J* = 16.9 Hz, 1H), 3.33 – 3.26 (m, 2H), 3.01 (d, *J* = 16.9 Hz, 1H), 2.44 (s, 3H), 2.31 – 2.22 (m, 2H), 1.21 (s, 3H), 1.20 (s, 3H); **^{13}C NMR** (151 MHz, CD_3OD) δ 180.3, 177.7, 153.3, 149.9, 138.2, 134.2, 132.1, 130.8, 128.5, 127.5, 124.4, 121.4, 111.9, 54.2, 46.2, 36.1, 28.6, 25.0, 21.5, 20.8; **HRMS** (ESI) 396.1070 m/z (M + H^+), calc. for $C_{21}H_{23}BrN_3^+$396.1070.

The ee was determined by HPLC analysis: CHIRALPAK IE (4.6 mm i.d. x 250 mm); hexane/2-propanol = 80/20; flow rate 1.0 mL/min; 25 °C; 254 nm; retention time: 12.1 min (minor) and 16.9 min (major).

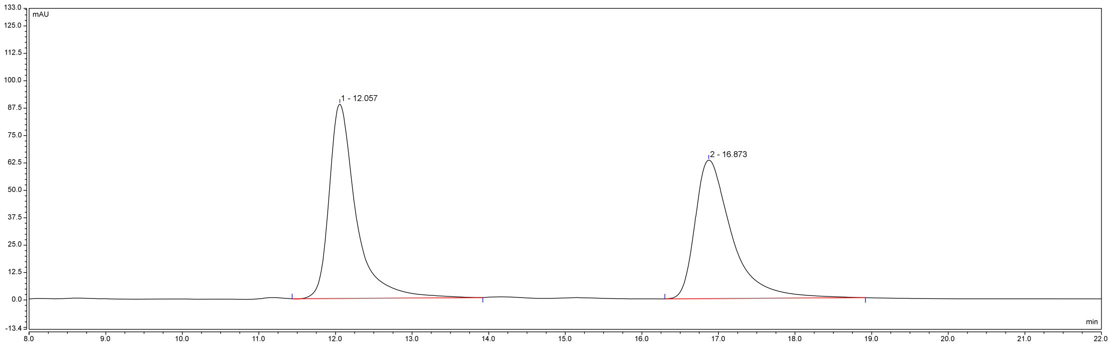

Entry	Retention Time	Area	Height	%Area
1	12.057	36.0942	88.60	50.77

2	16.873	34.9945	63.20	49.23

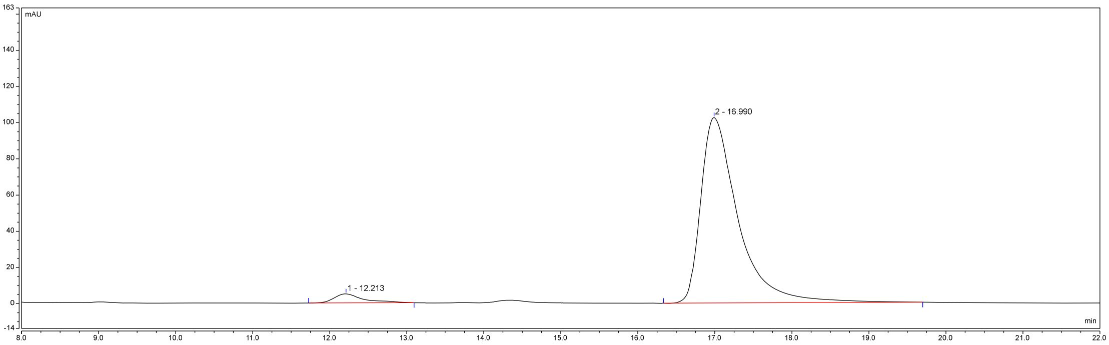

Entry	Retention Time	Area	Height	%Area
1	12.213	2.1926	4.99	3.68
2	16.990	57.3287	102.46	96.32

(*R*)-2-(2-bromophenyl)-7-(4-methoxypyridin-2-yl)-4,4-dimethyl-1,6-diazaspiro[4.4]nona-1,6-diene (34)

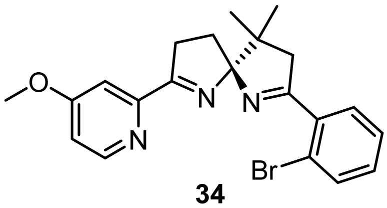

34

White solid; Mp 106.4−107.2 °C; 27.6 mg, 67% yield, 91% ee; $[\alpha]_D^{22}$ −26.5 (*c* 1.0, CH_3OH); **^{1}H NMR** (400 MHz, $CDCl_3$) δ 8.44 (d, *J* = 5.7 Hz, 1H), 7.74 (d, *J* = 2.6 Hz, 1H), 7.57 (dd, *J* = 8.0, 0.9 Hz, 1H), 7.52 (dd, *J* = 7.6, 1.7 Hz, 1H), 7.30 (td, *J* = 7.5, 1.0 Hz, 1H), 7.21 (td, *J* = 7.7, 1.7 Hz, 1H), 6.85 (dd, *J* = 5.7, 2.6 Hz, 1H), 3.89 (s, 3H), 3.34 (d, *J* = 16.4 Hz, 1H), 3.31 – 3.22 (m, 2H), 2.97 (d, *J* = 16.4 Hz, 1H), 2.35 – 2.23 (m, 1H), 2.13 (m, 1H), 1.16 (s, 3H), 1.14 (s, 3H); **^{13}C NMR** (151 MHz, $CDCl_3$) δ 177.1, 175.3, 165.9, 155.3, 150.3, 137.9, 133.4 130.7, 130.5, 127.4, 121.3, 111.9, 110.9, 107.87, 55.5, 53.3, 45.8, 35.4, 28.3, 24.8, 21.8; **HRMS** (ESI) 412.1019 m/z (M + H^+), calc. for $C_{21}H_{23}BrN_3O^+$ 412.1019. The ee was determined by HPLC analysis: CHIRALPAK IE (4.6 mm i.d. x 250 mm); hexane/2-propanol = 80/20; flow rate 1.0 mL/min; 25 °C; 254 nm; retention time: 17.8 min (minor) and 22.6 min (major).

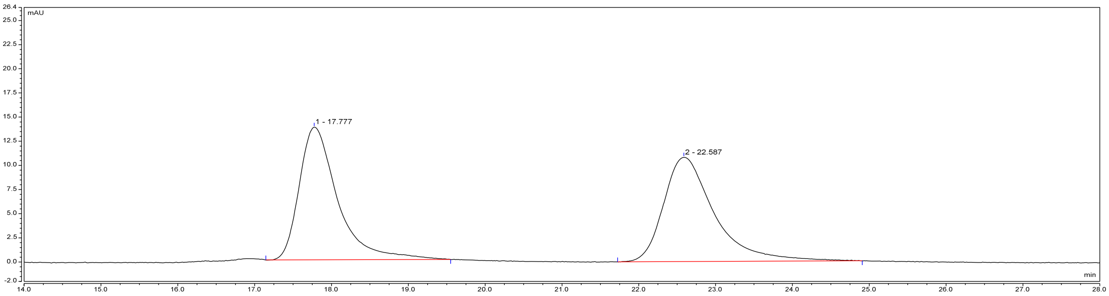

Entry	Retention Time	Area	Height	%Area
1	17.777	7.9726	13.73	49.17
2	22.587	8.2432	10.82	50.83

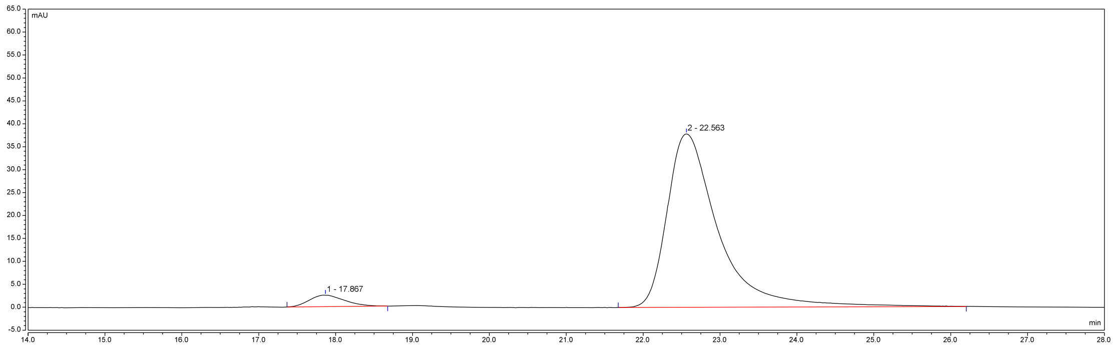

Entry	Retention Time	Area	Height	%Area
1	17.867	1.3272	2.53	4.36
2	22.563	29.1262	37.86	95.64

(*R*)-2-(2-bromophenyl)-4,4-dimethyl-7-(5-methylpyridin-2-yl)-1,6-diazaspiro[4.4]nona-1,6-diene (35)

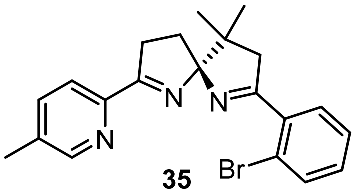

35

White solid; Mp 96.7−97.5 °C; 29.7 mg, 75% yield, 90% ee; $[\alpha]_D^{22}$ −36.9 (*c* 1.0, CH_3OH); **^{1}H NMR** (600 MHz, CD_3OD) δ 8.48 (dd, *J* = 1.4, 0.7 Hz, 1H), 8.05 (d, *J* = 8.0 Hz, 1H), 7.72 (d, *J* = 8.0 Hz, 1H), 7.65 (d, *J* = 8.0 Hz, 1H), 7.45 (dd, *J* = 7.7, 1.8 Hz, 1H), 7.41 (td, *J* = 7.5, 1.0 Hz, 1H), 7.33 (m, 1H), 3.35 (d, *J* = 16.9 Hz, 1H), 3.32 – 3.27 (m, 2H), 2.99 (d, *J* = 16.9 Hz, 1H), 2.40 (s, 3H), 2.27 – 2.21 (m, 2H), 1.19 (s, 3H), 1.19 (s, 3H); **^{13}C NMR** (151 MHz, CD_3OD) δ 178.9, 176.3, 149.7, 149.3, 137.2, 137.1, 136.0, 133.0, 130.8, 129.5, 127.3, 122.1, 120.2, 110.7, 53.0, 45.0, 34.9, 27.4, 23.8, 20.3, 17.0; **HRMS** (ESI) 396.1069 m/z (M + H^+), calc. for $C_{21}H_{23}BrN_3^+$ 396.1070.

The ee was determined by HPLC analysis: CHIRALPAK IE (4.6 mm i.d. x 250 mm); hexane/2-propanol = 80/20; flow rate 1.0 mL/min; 25 °C; 254 nm; retention time: 9.0 min (minor) and 11.2 min (major).

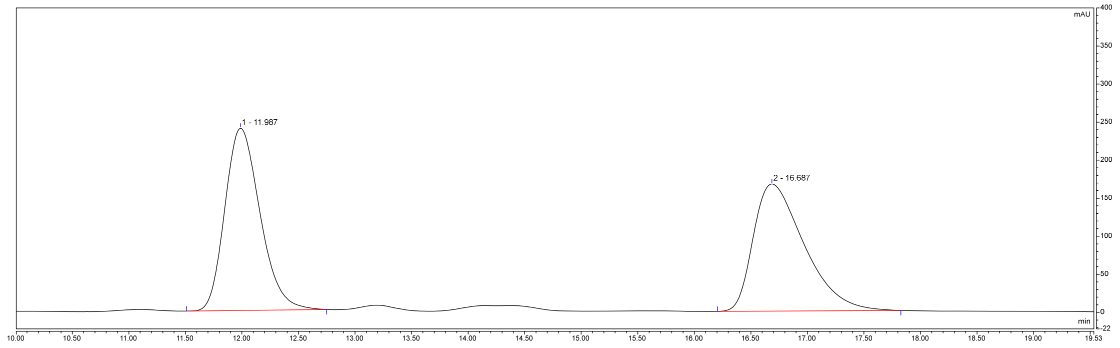

Entry	Retention Time	Area	Height	%Area
1	11.987	86.3239	239.32	49.83

2	16.687	86.9137	167.03	50.17

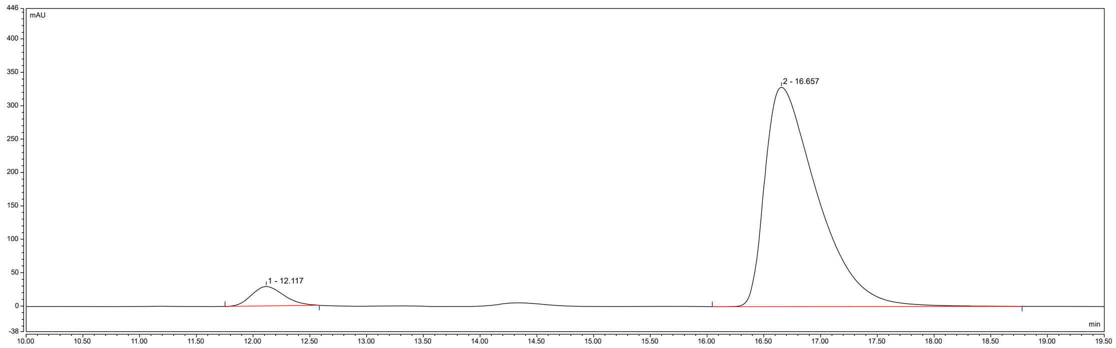

Entry	Retention Time	Area	Height	%Area
1	12.117	9.6115	29.11	5.19
2	16.657	175.6218	328.41	94.81

(*R*)-2-(2-bromophenyl)-4,4-dimethyl-7-(6-methylpyridin-2-yl)-1,6-diazaspiro[4.4]nona-1,6-diene (36)

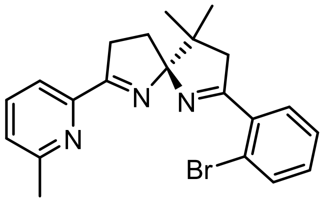

36

White solid; Mp 98.4−99.3 °C; 25.7 mg, 65% yield, 91% ee; $[\alpha]_D^{22}$ −38.0 (*c* 1.0, CH_3OH); **^{1}H NMR** (600 MHz, CD_3OD) δ 7.92 (d, *J* = 7.7 Hz, 1H), 7.76 (t, *J* = 7.7 Hz, 1H), 7.67 (d, *J* = 8.0 Hz, 1H), 7.47 (dd, *J* = 7.6, 1.7 Hz, 1H), 7.42 (m, 1H), 7.35 (d, *J* = 7.6 Hz, 2H), 3.36 (d, *J* = 16.9 Hz, 1H), 3.32 – 3.30 (m, 1H), 3.01 (d, *J* = 16.9 Hz, 1H), 2.59 (s, 3H), 2.56 (d, *J* = 5.6 Hz, 1H), 2.26 (t, *J* = 7.0 Hz, 2H), 1.21 (s, 3H), 1.20 (s, 3H); **^{13}C NMR** (151 MHz, CD_3OD) δ 180.2, 178.3, 159.6, 153.1, 138.3, 138.0, 134.2, 132.1, 130.8, 128.5, 126.0, 121.4, 120.6, 111.9, 54.2, 46.2, 36.2, 28.6, 25.1, 24.0, 21.5; **HRMS** (ESI) 396.1070 m/z (M + H^+), calc. for $C_{21}H_{23}BrN_3^+$ 396.1070.

The ee was determined by HPLC analysis: CHIRALPAK IE (4.6 mm i.d. x 250 mm); hexane/2-propanol = 80/20; flow rate 1.0 mL/min; 25 °C; 254 nm; retention time: 13.3 min (minor) and 18.8 min (major).

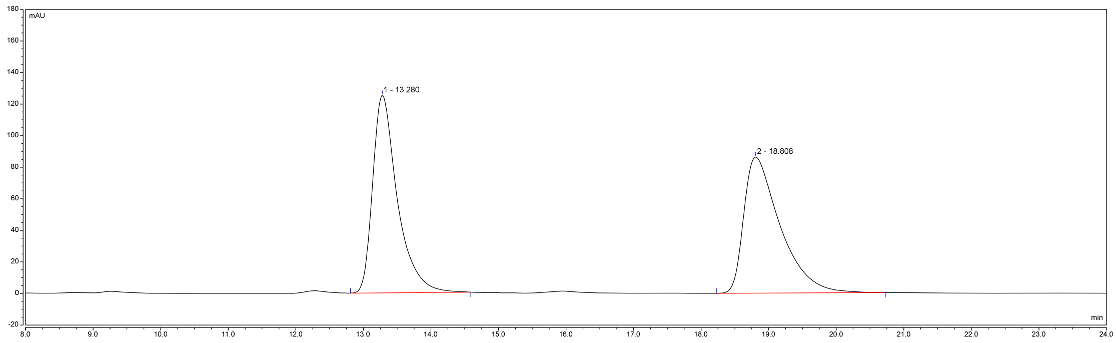

Entry	Retention Time	Area	Height	%Area
1	13.280	52.8715	125.10	49.63

2	18.808	53.6494	86.19	50.37

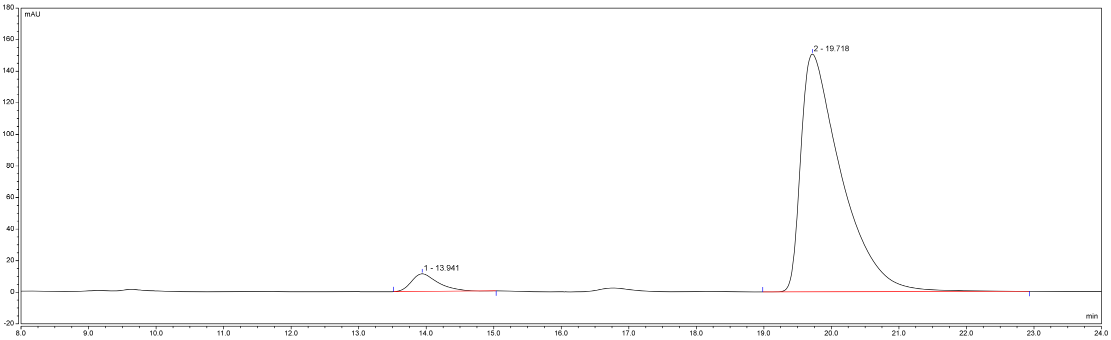

Entry	Retention Time	Area	Height	%Area
1	13.941	5.0326	11.15	4.60
2	19.718	104.2851	150.57	95.40

(*R*)-2-(7-(2-bromophenyl)-9,9-dimethyl-1,6-diazaspiro[4.4]nona-1,6-dien-2-yl)quinoline (37)

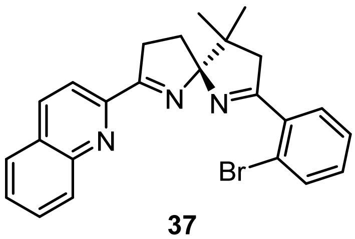

37

White solid; Mp 96.3−97.1 °C; 29.4 mg, 68% yield, 77% ee; $[\alpha]_D^{22}$ +30.7 (*c* 1.0, CH_3OH); **^{1}H NMR** (400 MHz, $CDCl_3$) δ 8.34 (d, *J* = 8.5 Hz, 1H), 8.15 (d, *J* = 8.4 Hz, 2H), 7.82 (dd, *J* = 8.1, 1.1 Hz, 1H), 7.72 (m, 1H), 7.62 – 7.50 (m, 3H), 7.31 (td, *J* = 7.5, 1.2 Hz, 1H), 7.25 – 7.18 (m, 1H), 3.55 – 3.42 (m, 2H), 3.42 – 3.33 (m, 1H), 3.01 (d, *J* = 16.4 Hz, 1H), 2.37 (m, 1H), 2.19 (m, 1H), 1.20 (s, 3H), 1.18 (s, 3H); **^{13}C NMR** (151 MHz, $CDCl_3$) δ 177.2, 175.9, 153.8, 147.9, 137.9, 135.9, 133.4, 130.6, 130.5, 130.1, 129.5, 128.7, 127.7, 127.4, 127.4, 121.3, 120.2, 111.2, 53.4, 45.8, 35.2, 28.3, 24.8, 21.8; **HRMS** (ESI) 432.1070 m/z (M + H^+), calc. for $C_{24}H_{23}BrN_3^+$ 432.1070.

The ee was determined by HPLC analysis: CHIRALPAK IE (4.6 mm i.d. x 250 mm); hexane/2-propanol = 80/20; flow rate 1.0 mL/min; 25 °C; 254 nm; retention time: 9.0 min (major) and 11.2 min (minor).

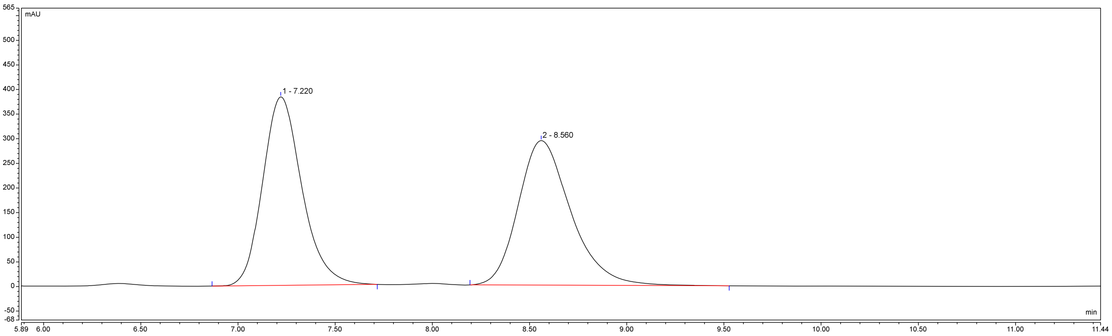

Entry	Retention Time	Area	Height	%Area
1	7.220	89.8899	382.99	49.43

2	8.560	91.9658	293.54	50.57

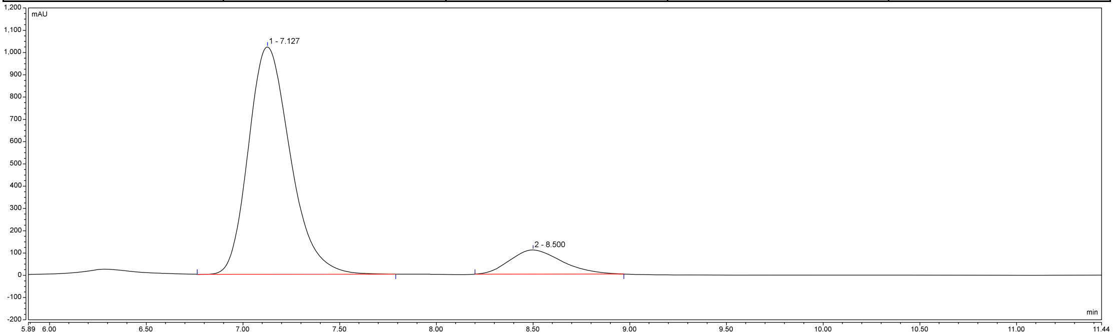

Entry	Retention Time	Area	Height	%Area
1	7.127	255.8833	1020.00	88.31
2	8.500	33.8698	108.37	11.69

(*R*)-1-(7-(2-bromophenyl)-9,9-dimethyl-1,6-diazaspiro[4.4]nona-1,6-dien-2-yl) isoquinoline (38)

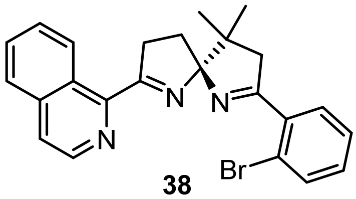

38

White solid; Mp 132.4−133.2 °C; 26.8 mg, 62% yield, 80% ee; $[\alpha]_D^{22}$ +57.3 (*c* 1.0, CH_3OH); **^{1}H NMR** (600 MHz, CD_3OD) δ 9.04 (d, *J* = 8.6 Hz, 1H), 8.55 (d, *J* = 5.6 Hz, 1H), 7.97 (d, *J* = 8.2 Hz, 1H), 7.87 (d, *J* = 5.6 Hz, 1H), 7.81 – 7.74 (m, 1H), 7.72 – 7.64 (m, 2H), 7.46 – 7.42 (m, 2H), 7.35 (m, 1H), 3.49 (m, 1H), 3.45 – 3.37 (m, 2H), 3.01 (d, *J* = 17.0 Hz, 1H), 2.35 – 2.28 (m, 2H), 1.31 (s, 3H), 1.27 (s, 3H); **^{13}C NMR** (151 MHz, CD_3OD) δ 180.7, 178.3, 154.1, 142.2, 138.5, 138.1, 134.2, 132.0, 131.7, 130.5, 129.2, 128.6, 128.4, 127.9, 127.7, 123.7, 121.3, 113.4, 54.4, 46.1, 39.2, 28.2, 24.9, 21.7; **HRMS** (ESI) 432.1069 m/z (M + H^+), calc. for $C_{24}H_{23}BrN_3^+$ 432.1070.

The ee was determined by HPLC analysis: CHIRALPAK IE (4.6 mm i.d. x 250 mm); hexane/2-propanol = 80/20; flow rate 1.0 mL/min; 25 °C; 254 nm; retention time: 9.6 min (minor) and 12.5 min (major).

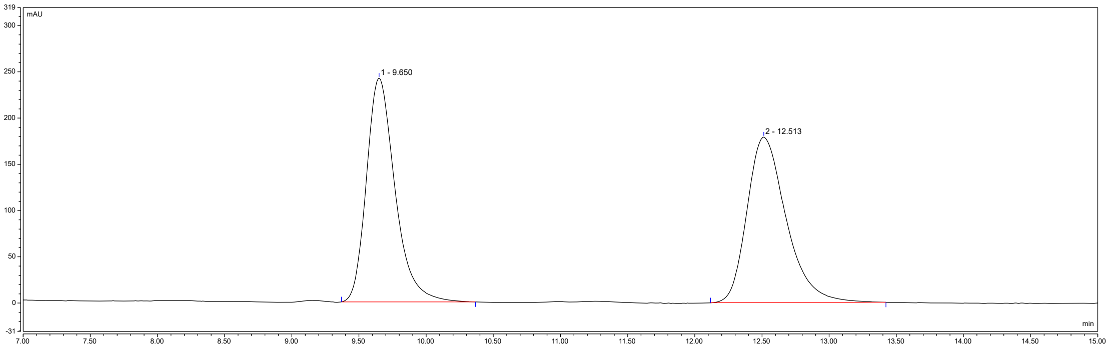

Entry	Retention Time	Area	Height	%Area

1	9.650	59.7291	241.86	49.70
2	12.513	60.4520	178.98	50.30

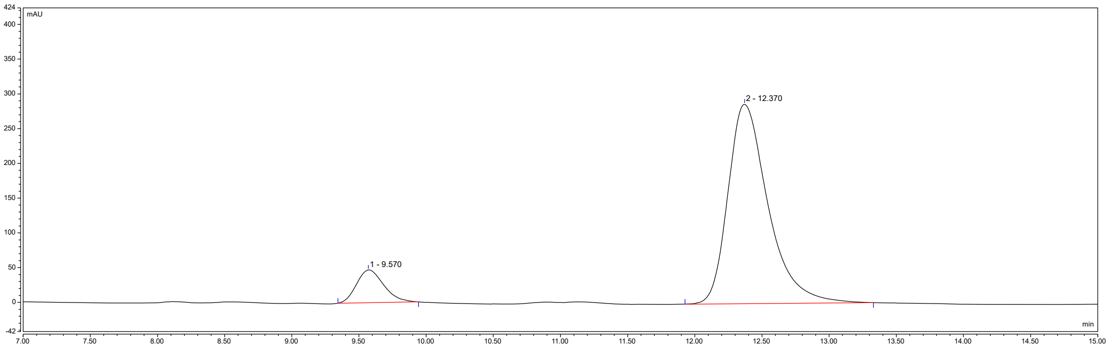

Entry	Retention Time	Area	Height	%Area
1	9.570	11.0719	47.11	10.03
2	12.370	99.3123	287.15	89.97

(*R*)-2-(2-bromophenyl)-4,4-diethyl-7-(pyridin-2-yl)-1,6-diazaspiro[4.4]nona-1,6-diene (39)

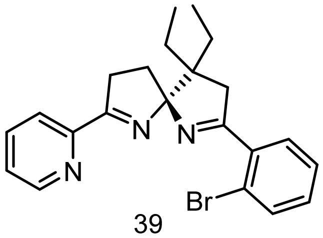

39

White solid; Mp 89.0−89.9 °C; 22.9 mg, 56% yield, 93% ee; $[\alpha]_D^{22}$ −32.9 (*c* 1.0, CH_3OH); **^{1}H NMR** (600 MHz, CD_3OD) δ 8.71 – 8.50 (m, 1H), 8.17 (d, *J* = 7.9 Hz, 1H), 7.94 – 7.80 (m, 1H), 7.65 (d, *J* = 8.0 Hz, 1H), 7.52 – 7.43 (m, 2H), 7.40 (m, 1H), 7.36 – 7.24 (m, 1H), 3.32 (m, 2H), 3.25 (d, *J* = 17.3 Hz, 1H), 3.09 (d, *J* = 17.3 Hz, 1H), 2.38 – 2.26 (m, 2H), 1.84 (m, 1H), 1.79 – 1.71 (m, 2H), 1.59 (m, 1H), 0.93 (t, *J* = 7.4 Hz, 3H), 0.83 (t, *J* = 7.5 Hz, 3H); **^{13}C NMR** (151 MHz, CD_3OD) δ 179.2, 177.1, 153.6, 150.1, 138.0, 137.9, 134.3, 132.1, 131.0, 128.5, 126.6, 123.6, 121.5, 112.2, 52.7, 50.5, 35.9, 30.1, 26.1, 25.8, 9.6, 9.5; **HRMS** (ESI) 410.1226 m/z (M + H^+), calc. for $C_{22}H_{25}BrN_3^+$ 410.1226.

The ee was determined by HPLC analysis: CHIRALPAK IE (4.6 mm i.d. x 250 mm); hexane/2-propanol = 90/10; flow rate 1.0 mL/min; 25 °C; 254 nm; retention time: 15.3 min (minor) and 16.8 min (major).

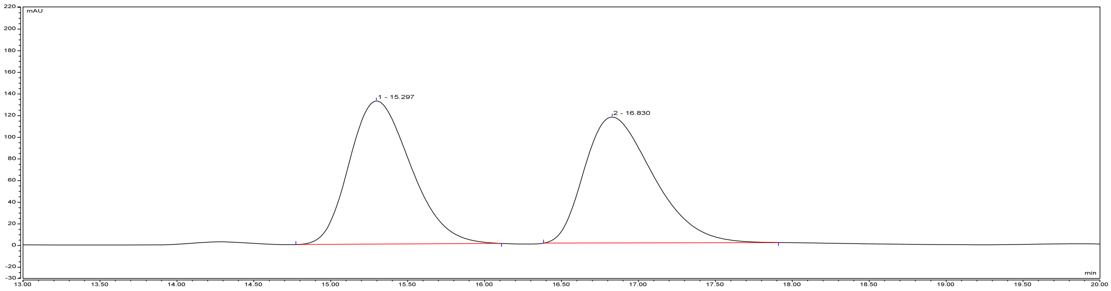

Entry	Retention Time	Area	Height	%Area
1	15.297	61.2781	132.23	50.42

2	16.830	60.2531	116.30	49.58

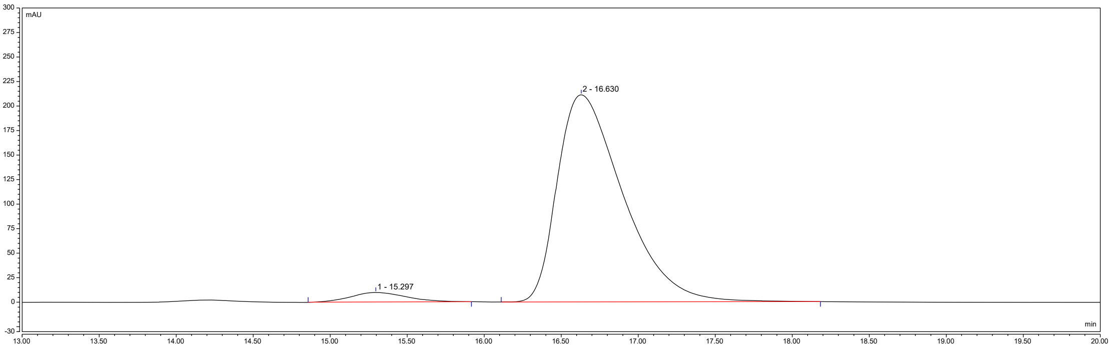

Entry	Retention Time	Area	Height	%Area
1	15.297	4.0545	9.71	3.74
2	16.630	104.3055	211.15	96.26

(*R*)-2-(2-bromophenyl)-4,4-dipropyl-7-(pyridin-2-yl)-1,6-diazaspiro[4.4]nona-1,6-diene (40)

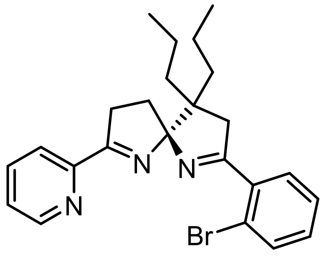

40

White solid; Mp 109.8−110.6 °C; 26.1 mg, 60% yield, 90% ee; $[\alpha]_D^{22}$ −36.6 (*c* 1.0, CH_3OH); **^{1}H NMR** (400 MHz, CD_3OD) δ 8.64 (dd, *J* = 4.8, 0.7 Hz, 1H), 8.16 (d, *J* = 7.9 Hz, 1H), 7.90 (m, 1H), 7.66 (d, *J* = 8.0 Hz, 1H), 7.51 – 7.43 (m, 2H), 7.41 (m, 1H), 7.36 – 7.29 (m, 1H), 3.43 – 3.31 (m, 1H), 3.27 (d, *J* = 17.3 Hz, 1H), 3.11 (d, *J* = 17.2 Hz, 1H), 2.42 – 2.29 (m, 2H), 1.79 – 1.62 (m, 3H), 1.56 – 1.46 (m, 1H), 1.38 – 1.24 (m, 3H), 1.22 – 1.11 (m, 1H), 0.96 (t, *J* = 7.2 Hz, 3H), 0.85 (t, *J* = 7.2 Hz, 3H); **^{13}C NMR** (151 MHz, CD_3OD) δ 179.3, 177.1, 153.6, 150.2, 138.1, 137.9, 134.4, 132.1, 131.1, 128.5, 126.6, 123.6, 121.5, 112.3, 52.5, 51.2, 36.9, 36.8, 35.9, 30.0, 19.4, 19.0, 15.2, 15.2; **HRMS** (ESI) 438.1539 m/z (M + H^+), calc. for $C_{24}H_{29}BrN_3^+$ 438.1539.

The ee was determined by HPLC analysis: CHIRALPAK IC (4.6 mm i.d. x 250 mm); hexane/2-propanol = 80/20; flow rate 1.0 mL/min; 25 °C; 254 nm; retention time: 10.4 min (minor) and 13.4 min (major).

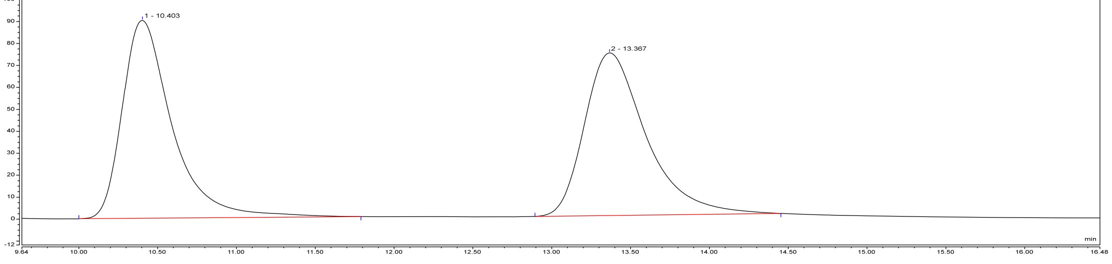

Entry	Retention Time	Area	Height	%Area

1	10.403	33.0103	90.12	49.64
2	13.367	33.4931	74.03	50.36

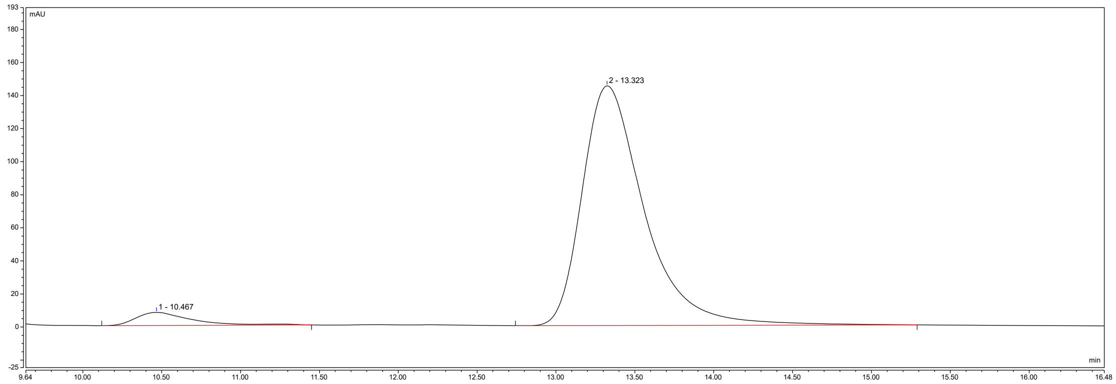

Entry	Retention Time	Area	Height	%Area
1	10.467	3.2124	7.92	4.63
2	13.323	66.1171	144.97	95.37

(*R*)-4,4-dibenzyl-2-(2-bromophenyl)-7-(pyridin-2-yl)-1,6-diazaspiro[4.4]nona-1,6-diene (41)

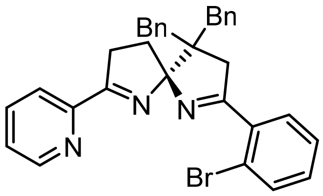

41

Yellow oil; 30.4 mg, 57% yield, 90% ee; $[\alpha]_D^{22}$ +47.2 (*c* 1.0, CH_3OH); **^{1}H NMR** (600 MHz, CD_3OD) δ 8.68 – 8.64 (m, 1H), 8.28 (d, *J* = 7.9 Hz, 1H), 7.93 (m, 1H), 7.56 (d, *J* = 8.0 Hz, 1H), 7.54 – 7.49 (m, 1H), 7.28 (d, *J* = 7.7 Hz, 2H), 7.25 – 7.20 (m, 4H), 7.16 – 7.13 (m, 4H), 7.10 (d, *J* = 7.1 Hz, 2H), 6.52 (dd, *J* = 7.7, 1.4 Hz, 1H), 3.84 (d, *J* = 16.8 Hz, 1H), 3.38 – 3.33 (m, 1H), 3.27 (d, *J* = 13.1 Hz, 1H), 3.24 (s, 1H), 3.05 (d, *J* = 16.8 Hz, 1H), 2.93 (d, *J* = 14.0 Hz, 1H), 2.90 – 2.83 (m, 2H), 2.56 – 2.49 (m, 1H), 2.38 – 2.31 (m, 1H); **^{13}C NMR** (151 MHz, CD_3OD) δ 179.1, 176.8, 153.7, 150.2, 140.2, 139.8, 138.1, 137.3, 134.1, 132.0, 131.6, 131.4, 129.2, 128.9, 127.9, 127.2, 126.6, 123.8, 121.3, 112.5, 56.0, 40.9, 40.9, 36.2, 30.7, 29.5; **HRMS** (ESI) 534.1539 m/z (M + H$^+$), calc. for $C_{32}H_{29}BrN_3^+$ 534.1539.

The ee was determined by HPLC analysis: CHIRALPAK IE (4.6 mm i.d. x 250 mm); hexane/2-propanol = 80/20; flow rate 1.0 mL/min; 25 °C; 254 nm; retention time: 11.7 min (minor) and 13.1 min (major).

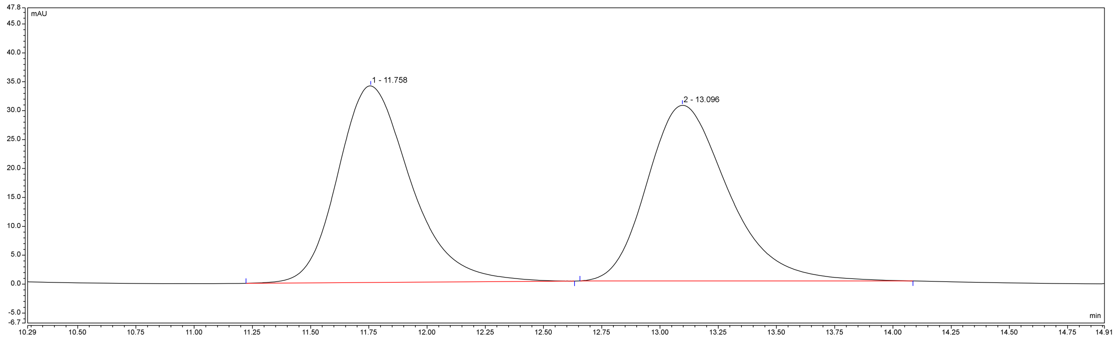

Entry	Retention Time	Area	Height	%Area
1	11.758	12.3496	33.99	50.14
2	13.096	12.2806	30.38	49.86

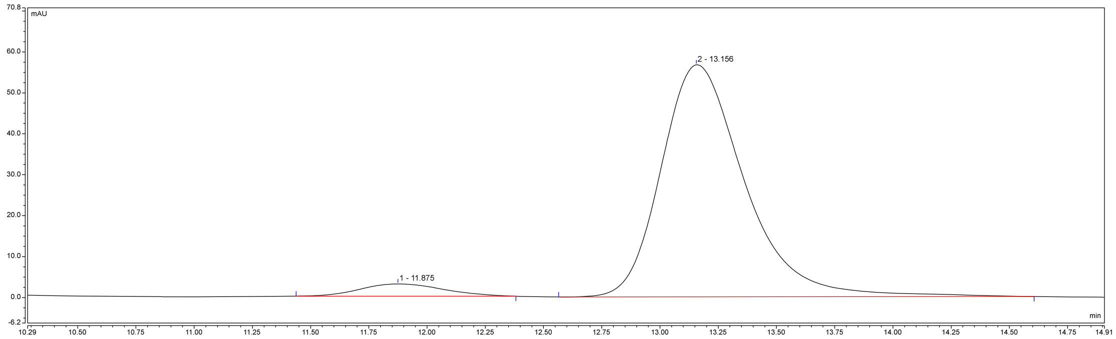

Entry	Retention Time	Area	Height	%Area
1	11.875	1.2308	3.00	4.96
2	13.156	23.5759	56.66	95.04

(*R*)-11-(2-bromophenyl)-7-(pyridin-2-yl)-6,10-diazadispiro $[3.0.4^5.3^4]$ dodeca-6,10-diene (42)

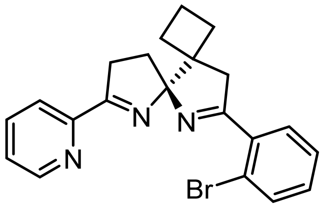

42

White solid; Mp 102.5−103.3 °C; 24.0 mg, 61% yield, 63% ee; $[\alpha]_D^{22}$ −70.7 (*c* 1.0, CH_3OH); **^{1}H NMR** (600 MHz, CD_3OD) δ 8.66 (dd, *J* = 4.8, 0.6 Hz, 1H), 8.20 (d, *J* = 7.9 Hz, 1H), 7.90 (td, *J* = 7.8, 1.7 Hz, 1H), 7.65 (d, *J* = 8.0 Hz, 1H), 7.52 – 7.47 (m, 1H), 7.45 (dd, *J* = 7.6, 1.7 Hz, 1H), 7.42 (m, 1H), 7.33 (td, *J* = 7.9, 1.8 Hz, 1H), 3.54 (d, *J* = 17.2 Hz, 1H), 3.41 (d, *J* = 17.2 Hz, 1H), 3.39 – 3.29 (m, 2H), 2.47 (m, 1H), 2.37 – 2.26 (m, 1H), 2.26 – 2.19 (m, 1H), 2.16 (m, 1H), 2.04 – 1.93 (m, 3H), 1.76 (m, 1H); **^{13}C NMR** (151 MHz, CD_3OD) δ 178.9, 178.7, 153.3, 150.2, 138.2, 138.1, 134.1, 132.1, 130.6, 128.6, 126.7, 123.7, 121.4, 110.7, 53.4, 52.9, 36.5, 30.4, 29.6, 28.5, 16.8; **HRMS** (ESI) 394.0914 m/z (M + H^+), calc. for $C_{21}H_{21}BrN_3^+$ 394.0913.

The ee was determined by HPLC analysis: CHIRALPAK IE (4.6 mm i.d. x 250 mm); hexane/2-propanol = 80/20; flow rate 1.0 mL/min; 25 °C; 254 nm; retention time: 15.1 min (major) and

17.3 min (minor).

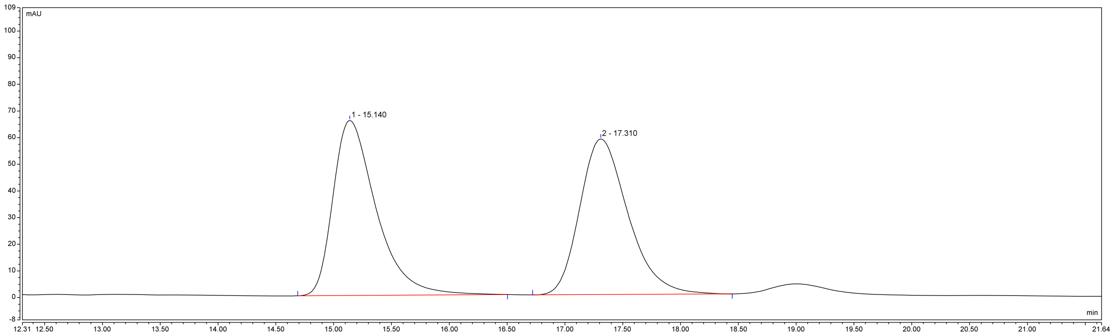

Entry	Retention Time	Area	Height	%Area
1	15.140	28.3819	65.65	50.19
2	17.310	28.1718	58.35	49.81

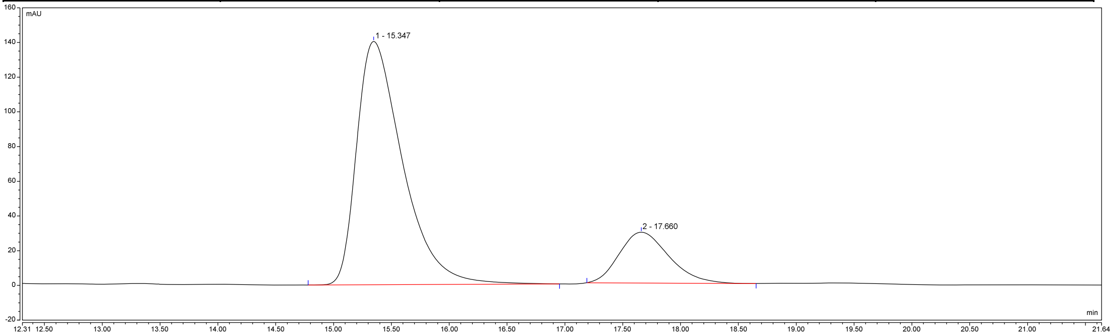

Entry	Retention Time	Area	Height	%Area
1	15.347	64.4327	140.21	81.58
2	17.660	14.5477	29.29	18.42

(*R*)-12-(2-bromophenyl)-2-(pyridin-2-yl)-1,13-diazadispiro [4.0.4^{6}.3^{5}]trideca-1,12-diene (43)

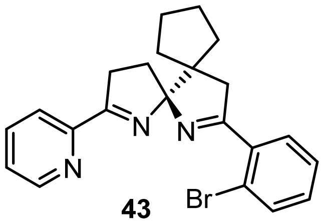

43

White solid; Mp 110.2−111.0 °C; 28.1 mg, 69% yield, 90% ee; $[\alpha]_D^{22}$ −78.7 (*c* 1.0, CH_3OH); **^{1}H NMR** (400 MHz, CD_3OD) δ 8.65 (d, *J* = 4.8 Hz, 1H), 8.15 (d, *J* = 7.9 Hz, 1H), 7.90 (m, 1H), 7.66 (d, *J* = 8.0 Hz, 1H), 7.49 (m, 1H), 7.47 – 7.38 (m, 2H), 7.33 (m, 1H), 3.31 (m, 3H), 3.13 (d, *J* = 16.9 Hz, 1H), 2.35 – 2.17 (m, 2H), 1.76 (m, 8H); **^{13}C NMR** (151 MHz, CD_3OD) δ 180.1, 177.9, 153.5, 150.2, 138.3, 138.1, 134.2, 132.1, 130.7, 128.6, 126.6, 123.7, 121.4, 111.6, 58.5, 53.1, 36.0, 35.3, 33.2, 29.9, 24.9, 24.9; **HRMS** (ESI) 408.1072 m/z (M + H$^+$), calc. for $C_{22}H_{23}BrN_3^+$ 408.1070.

The ee was determined by HPLC analysis: CHIRALPAK IE (4.6 mm i.d. x 250 mm); hexane/2-propanol = 85/15; flow rate 1.0 mL/min; 25 °C; 254 nm; retention time: 20.2 min (minor) and 22.6 min (major).

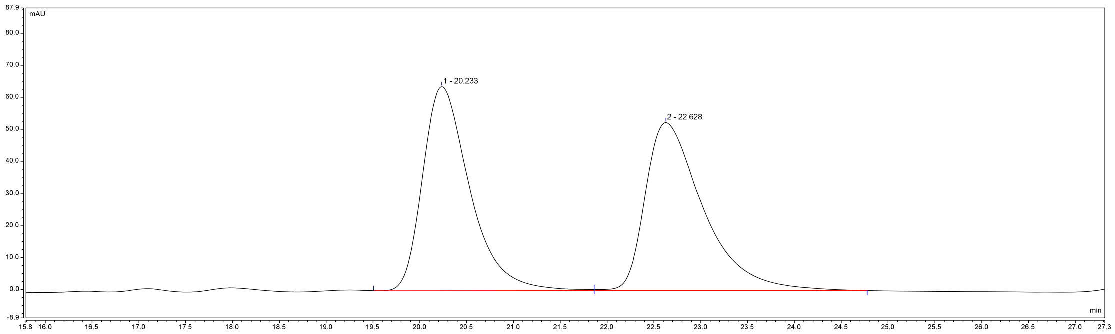

Entry	Retention Time	Area	Height	%Area
1	20.233	37.2799	63.74	49.71
2	22.628	37.7167	52.49	50.29

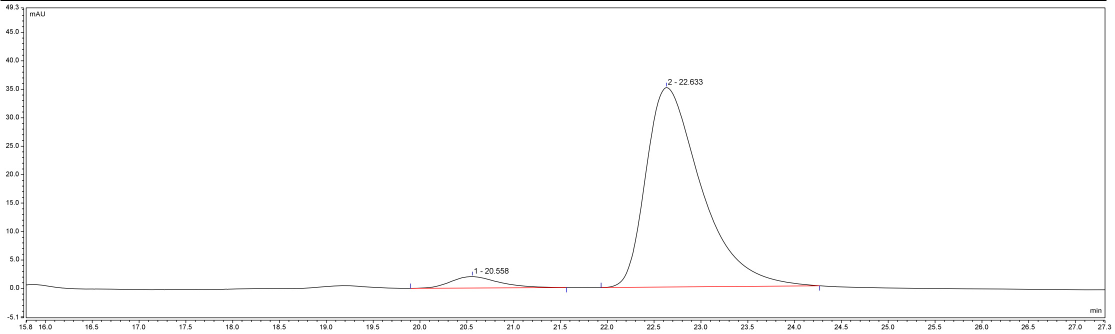

Entry	Retention Time	Area	Height	%Area
1	20.558	1.1840	2.01	4.69
2	22.633	24.0835	35.05	95.31

(*R*)-5'-(2-bromophenyl)-5''-(pyridin-2-yl)-1,3,3'',4''-tetrahydro-4'H-dispiro[indene-2,3'-pyrrole-2',2''-pyrrole] (44)

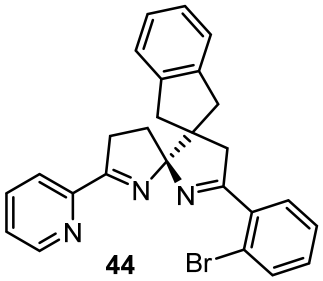

44

White solid; Mp 144.4−145.2 °C; 26.4 mg, 58% yield, 85% ee; $[\alpha]_D^{22}$ −41.0 (*c* 1.0, CH_3OH); **^{1}H NMR** (400 MHz, CD_3OD) δ 8.64 (d, *J* = 4.7 Hz, 1H), 8.16 (d, *J* = 7.9 Hz, 1H), 7.91 (td, *J* = 7.8, 1.6 Hz, 1H), 7.66 (d, *J* = 7.9 Hz, 1H), 7.53 – 7.46 (m, 2H), 7.43 (t, *J* = 7.1 Hz, 1H), 7.34 (td, *J* = 7.7, 1.8 Hz, 1H), 7.24 – 7.13 (m, 2H), 7.13 – 7.05 (m, 2H), 3.46 (d, *J* = 17.0 Hz, 1H), 3.41 – 3.32 (m, 1H), 3.27 – 3.16 (m, 4H), 3.07 (d, *J* = 16.3 Hz, 1H), 3.02 (d, *J* = 15.8 Hz, 1H), 2.38 – 2.28 (m, 2H); **^{13}C NMR** (101 MHz, CD_3OD) δ 180.2, 178.4, 153.4, 150.2, 143.1, 142.6, 138.1, 134.2, 132.2, 130.8, 128.6, 127.5, 127.4, 126.7, 125.3, 125.3, 123.8, 121.5, 111.1, 59.0, 53.5, 42.6, 39.8, 36.2, 30.2; **HRMS** (ESI) 456.1070 m/z (M + H$^+$), calc. for $C_{26}H_{23}BrN_3^+$ 456.1070.

The ee was determined by HPLC analysis: CHIRALPAK IE (4.6 mm i.d. x 250 mm); hexane/2-propanol = 85/15; flow rate 1.0 mL/min; 25 °C; 254 nm; retention time: 25.8 min (minor) and 30.1 min (major).

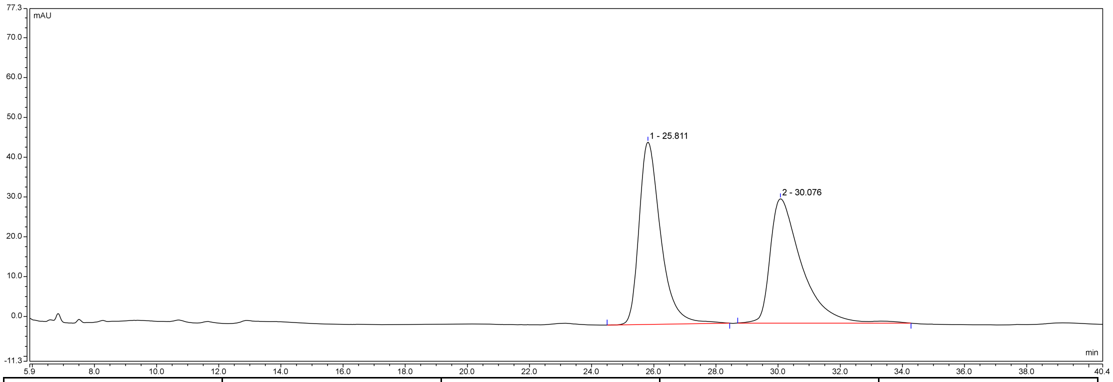

Entry	Retention Time	Area	Height	%Area
1	25.811	36.8749	45.77	50.13
2	30.076	36.6763	31.23	49.87

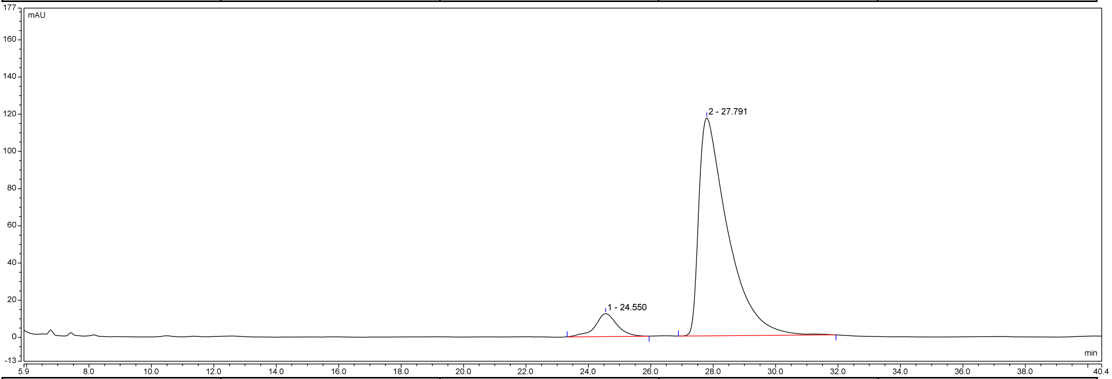

Entry	Retention Time	Area	Height	%Area
1	24.550	10.0530	12.34	7.40
2	27.791	125.8880	117.19	92.60

(*R*)-13-(2-bromophenyl)-2-(pyridin-2-yl)-1,14-diazadispiro[4.0.5^6.3^5]tetradeca-1,13-diene (45)

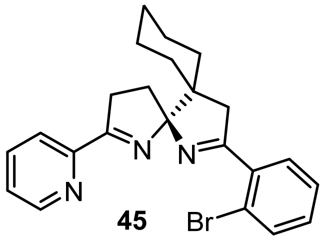

45

White solid; Mp 120.8−121.6 °C; 32.9 mg, 78% yield, 94% ee; $[\alpha]_D^{22}$ −36.0 (*c* 1.0, CH_3OH); **^{1}H NMR** (400 MHz, CD_3OD) δ 8.64 (m, 1H), 8.14 (dt, *J* = 7.9, 1.0 Hz, 1H), 7.90 (td, *J* = 7.8, 1.7 Hz, 1H), 7.69 – 7.62 (m, 1H), 7.48 (m, 1H), 7.44 – 7.40 (m, 2H), 7.33 (m, 1H), 3.35 (d, *J* = 17.1 Hz, 1H), 3.30 (m, 2H), 3.19 (dd, *J* = 17.1, 1.1 Hz, 1H), 2.31 (m, 1H), 2.21 (m, 1H), 1.75 – 1.70 (m, 3H), 1.58 – 1.19 (m, 7H); **^{13}C NMR** (151 MHz, CD_3OD) δ 180.2, 177.6, 153.6, 150.2, 138.4, 138.1, 134.2, 132.1, 130.8, 128.6 , 126.6, 123.6, 121.4, 112.5, 50.7, 35.8, 31.8, 31.5, 30.7, 28.7, 27.2, 25.0, 23.7; **HRMS** (ESI) 422.1222 m/z (M + H$^+$), calc. for $C_{23}H_{25}BrN_3^+$ 422.1226.

The ee was determined by HPLC analysis: CHIRALPAK IE (4.6 mm i.d. x 250 mm); hexane/2-propanol = 80/20; flow rate 1.0 mL/min; 25 °C; 254 nm; retention time: 12.0 min (minor) and 14.7 min (major).

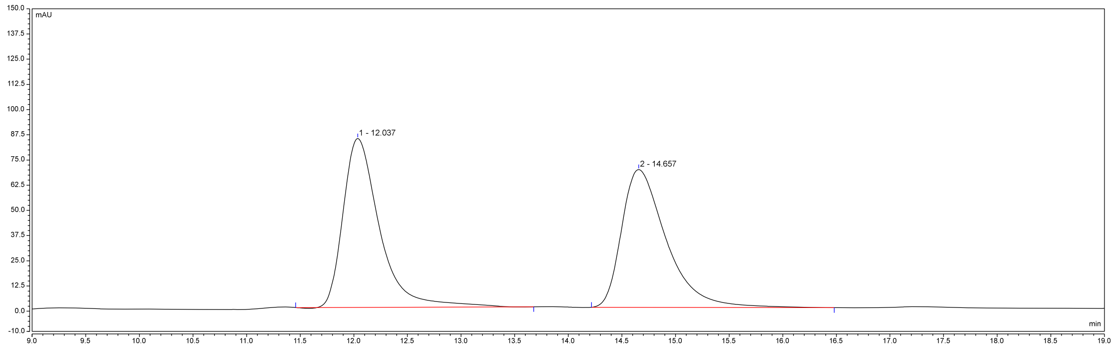

Entry	Retention Time	Area	Height	%Area
1	12.037	33.0808	83.71	50.04
2	14.657	33.0324	68.30	49.96

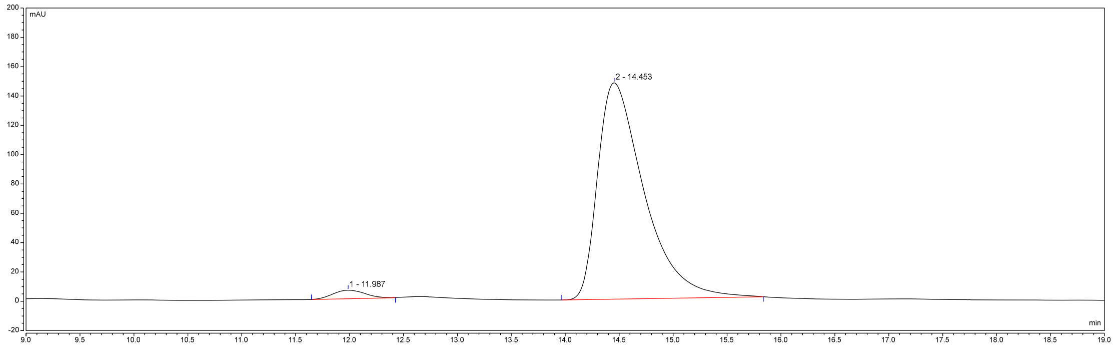

Entry	Retention Time	Area	Height	%Area
1	11.987	1.9936	5.78	2.64
2	14.453	73.6515	147.46	97.36

(*R*)-13-(2-bromophenyl)-9,9-difluoro-2-(pyridin-2-yl)-1,14-diazadispiro[4.0.5^6.3^5]tetradeca-1,13-diene (46)

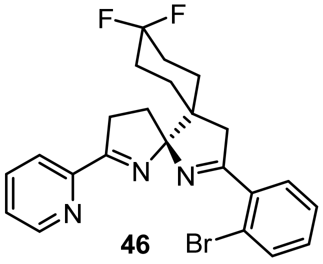

46

White solid; Mp 131.8−132.6 °C; 20.6 mg, 45% yield, 99% ee; $[\alpha]_D^{22}$ −34.1 (*c* 1.0, CH_3OH); **^{1}H NMR** (600 MHz, $CDCl_3$) δ 8.65 (m, 1H), 8.16 (d, *J* = 7.9 Hz, 1H), 7.72 (td, *J* = 7.7, 1.8 Hz, 1H), 7.58 (dd, *J* = 8.0, 1.0 Hz, 1H), 7.47 (dd, *J* = 7.6, 1.7 Hz, 1H), 7.35 – 7.28 (m, 2H), 7.25 – 7.18 (m, 1H), 3.37 – 3.29 (m, 3H), 3.21 (dd, *J* = 16.5, 1.0 Hz, 1H), 2.32 (m, 1H), 2.15 – 2.11 (m, 2H), 2.09 – 2.04 (m, 1H), 1.92 (m, 1H), 1.79 (m, 2H), 1.71 (m, 2H), 1.57 (td, *J* = 13.8, 3.7 Hz, 1H); **^{13}C NMR** (151 MHz, $CDCl_3$) δ 176.4, 175.9 153.3, 149.3, 137.5, 136.3, 133.3, 130.9, 130.6, 127.6, 125.0, 123.5 (dd, *J* = 242.5, 239.4 Hz), 122.7, 121.2), 110.8, 48.6, 46.5, 35.1, 32.3 (dd, *J* = 25.5, 22.4 Hz), 30.9 (dd, *J* = 25.1, 23.3 Hz), 28.5, 27.29 (d, *J* = 9.6 Hz); **^{19}F NMR** (565 MHz, CD_3OD) δ -92.21 (d, *J* =

236.3 Hz), -104.90 (d, J = 236.6 Hz); **HRMS** (ESI) 458.1038 m/z (M + H^+), calc. for $C_{23}H_{23}BrF_2N_3^+$ 458.1038.

The ee was determined by HPLC analysis: CHIRALPAK IC (4.6 mm i.d. x 250 mm); hexane/2-propanol = 90/10; flow rate 1.0 mL/min; 25 °C; 254 nm; retention time: 18.2 min (major) and 23.3 min (minor).

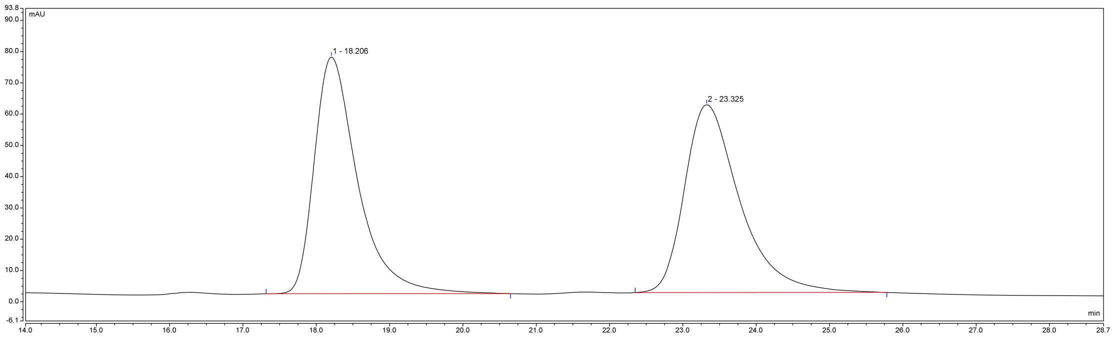

Entry	Retention Time	Area	Height	%Area
1	18.206	51.9119	75.66	49.79
2	23.325	52.3536	60.01	50.21

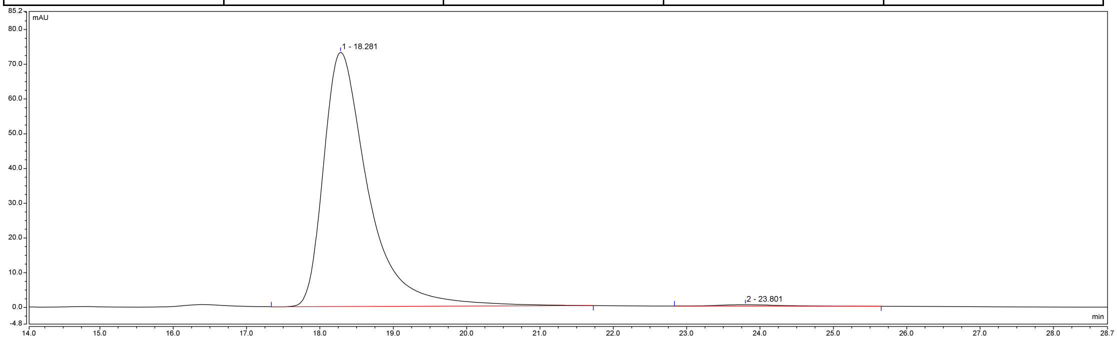

Entry	Retention Time	Area	Height	%Area
1	18.281	51.5021	73.17	99.36
2	23.801	0.3298	0.38	0.64

(*R*)-13-(2-bromophenyl)-9,9-dimethyl-2-(pyridin-2-yl)-1,14-diazadispiro[4.0.5^6.3^5]-tetradeca-1,13-diene (47)

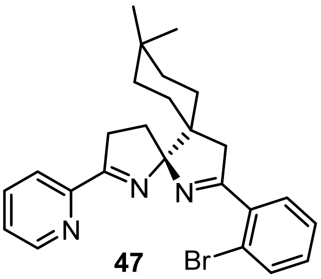
47

White solid; Mp 113.3−114.1 °C; 31.9 mg, 71% yield, 92% ee; $[\alpha]_D^{22}$ −32.9 (c 1.0, CH_3OH); **^{1}H NMR** (600 MHz, CD_3OD) δ 8.69 – 8.59 (m, 1H), 8.15 (d, J = 7.9 Hz, 1H), 7.91 (t, J = 7.7 Hz, 1H), 7.67 (d, J = 8.0 Hz, 1H), 7.51 – 7.47 (m, 1H), 7.44 – 7.39 (m, 2H), 7.34 (m, 1H), 3.37 – 3.33 (m, 1H), 3.33 – 3.29 (m, 2H), 3.17 (t, J = 16.9 Hz, 1H), 2.36 – 2.29 (m, 1H), 2.28 – 2.20 (m, 1H), 1.80 – 1.72 (m, 1H), 1.67 – 1.62 (m, 1H), 1.59 – 1.54 (m, 1H), 1.54 – 1.47 (m, 2H), 1.43 – 1.37 (m, 2H), 1.34 – 1.29 (m, 1H),

0.95 (s, 3H), 0.94 (s, 3H). **^{13}C NMR** (151 MHz, CD_3OD) δ 180.2, 177.6, 153.6, 150.2, 138.3, 138.1, 134.2, 132.1, 130.8, 128.6, 126.6, 123.6, 121.3, 112.5, 50.5, 37.8, 36.5, 35.9, 33.2, 30.6, 28.9, 27.6, 27.3, 24.0. **HRMS** (ESI) 450.1539 m/z (M + H^+), calc. for $C_{25}H_{29}BrN_3^+$ 450.1539. The ee was determined by HPLC analysis: CHIRALPAK IE (4.6 mm i.d. x 250 mm); hexane/2-propanol = 80/20; flow rate 1.0 mL/min; 25 °C; 254 nm; retention time: 9.1 min (minor) and 10.3 min (major).

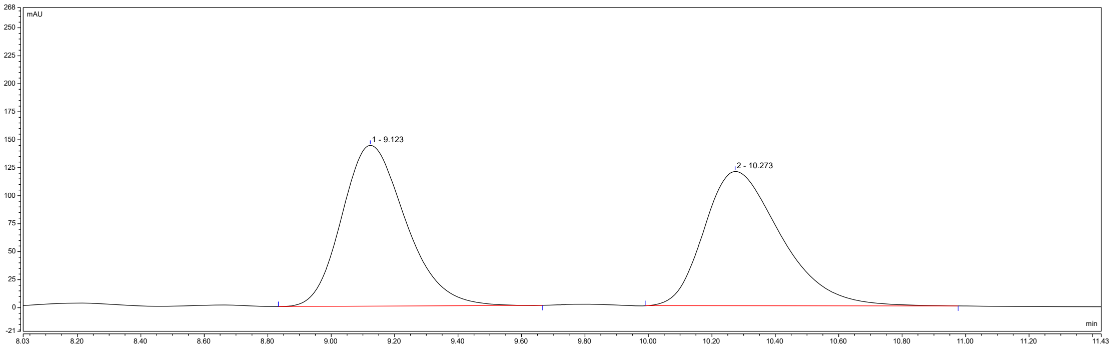

Entry	Retention Time	Area	Height	%Area
1	9.123	33.5631	143.61	49.89
2	10.273	33.7144	119.91	50.11

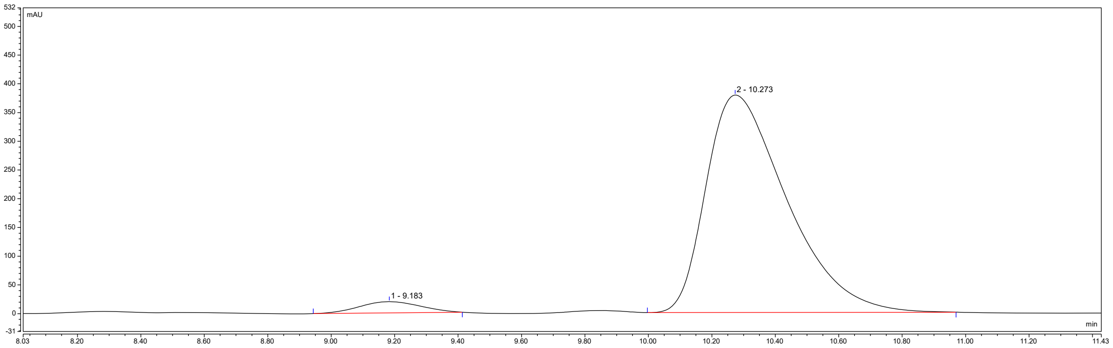

Entry	Retention Time	Area	Height	%Area
1	9.183	4.3674	19.66	3.77
2	10.273	111.5968	378.55	96.23

(*R*)-17-(2-bromophenyl)-2-(pyridin-2-yl)-10,13-dioxa-1,18-diazatrispiro[4.0.2.4^9.2^6.3^5]-octadeca-1,17-diene (48)

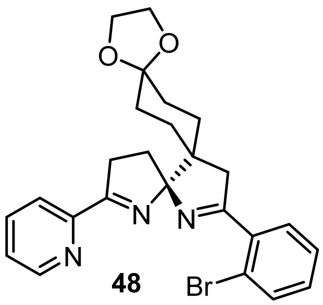

48

White solid; Mp 79.4−80.3 °C; 20.6 mg, 43% yield, 90% ee; $[\alpha]_D^{22}$ +36.7 (*c* 1.0, CH_3OH); **^{1}H NMR** (400 MHz, CD_3OD) δ 8.69 – 8.60 (m, 1H), 8.20 – 8.10 (m, 1H), 7.91 (td, *J* = 7.8, 1.6 Hz, 1H), 7.67 (d, *J* = 7.8 Hz, 1H), 7.53 – 7.46 (m, 1H), 7.46 – 7.38 (m, 2H), 7.35 (m, 1H), 3.93 (s, 4H), 3.39 (d, *J* = 17.1 Hz, 1H), 3.32 – 3.28 (m, 2H), 3.23 (d, *J* = 17.1 Hz, 1H), 2.36 – 2.19 (m, 2H), 1.95 (d, *J* = 9.9 Hz, 1H), 1.76 (m, 5H), 1.64 – 1.58 (m, 2H). **^{13}C NMR** (101 MHz, CD_3OD) δ 180.0, 177.7, 153.5, 150.2, 138.2, 138.0, 134.2, 132.2, 130.8, 128.6, 126.6, 123.7, 121.4, 112.1, 109.4, 65.1, 65.1, 35.9, 33.7, 32.4, 29.2, 28.8; **HRMS** (ESI) 480.1281 m/z (M + H^+), calc. for $C_{25}H_{27}BrN_3O_2^+$ 480.1281.

The ee was determined by HPLC analysis: CHIRALPAK IC (4.6 mm i.d. x 250 mm); hexane/2-propanol = 80/20; flow rate 1.0 mL/min; 25 °C; 254 nm; retention time: 18.3 min (major) and 24.3 min (minor).

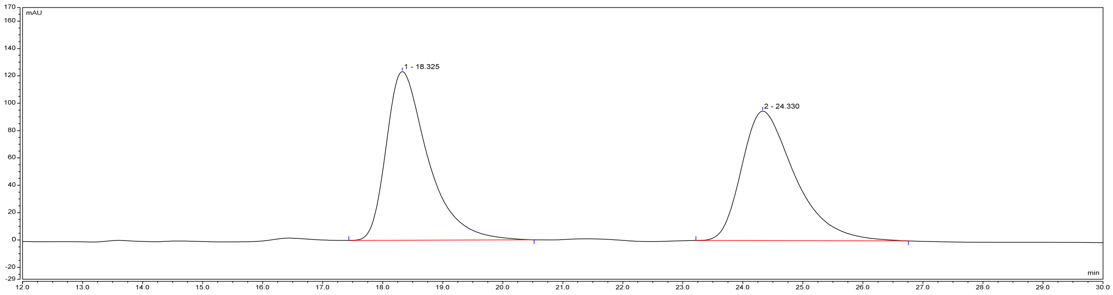

Entry	Retention Time	Area	Height	%Area
1	18.325	99.5481	123.27	50.58
2	24.330	97.2757	94.66	49.42

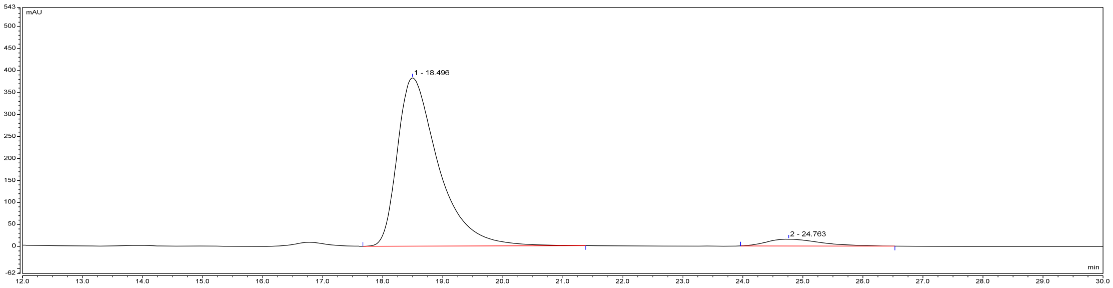

Entry	Retention Time	Area	Height	%Area
1	18.496	302.8833	382.19	95.04
2	24.763	15.7916	15.72	4.96

(*R*)-13-(2-bromophenyl)-2-(pyridin-2-yl)-9-oxa-1,14-diazadispiro[4.0.5^6.3^5]tetradeca-1,13-diene (49)

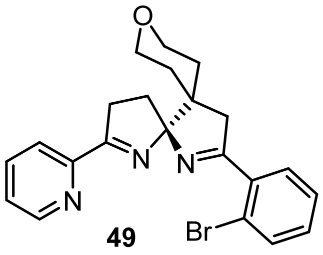

49

White solid; Mp 91.6–92.5 °C; 22.0 mg, 52% yield, 90% ee; $[\alpha]_D^{22}$ −25.0 (*c* 1.0, CH_3OH); **^{1}H NMR** (400 MHz, CD_3OD) δ 8.65 (m, 1H), 8.15 (dt, *J* = 7.9, 1.0 Hz, 1H), 7.90 (td, *J* = 7.8, 1.7 Hz, 1H), 7.67 (dd, *J* = 8.0, 0.7 Hz, 1H), 7.49 (m, 1H), 7.47 – 7.39 (m, 2H), 7.35 (m, 1H), 3.92 (m, 2H), 3.73 – 3.63 (m, 1H), 3.58 – 3.45 (m, 2H), 3.36 – 3.30 (m, 3H), 2.34 (m, 1H), 2.29 – 2.18 (m, 1H), 1.82 – 1.75 (m, 2H), 1.72 – 1.56 (m, 2H); **^{13}C NMR** (151 MHz, CD_3OD) δ 179.7, 178.2, 153.4, 150.2, 138.1, 134.2, 132.2, 130.8, 128.6, 126.7, 125.9, 123.7, 121.4, 112.2, 66.8, 65.5, 47.8, 35.9, 32.2, 31.9, 30.7, 28.9; **HRMS** (ESI) 424.1019 m/z (M +H$^+$), calc. for $C_{22}H_{23}BrN_3O^+$ 424.1019.

The ee was determined by HPLC analysis: CHIRALPAK IC (4.6 mm i.d. x 250 mm); hexane/2-propanol = 80/20; flow rate 1.0 mL/min; 25 °C; 254 nm; retention time: 26.1 min (major) and 31.6 min (minor).

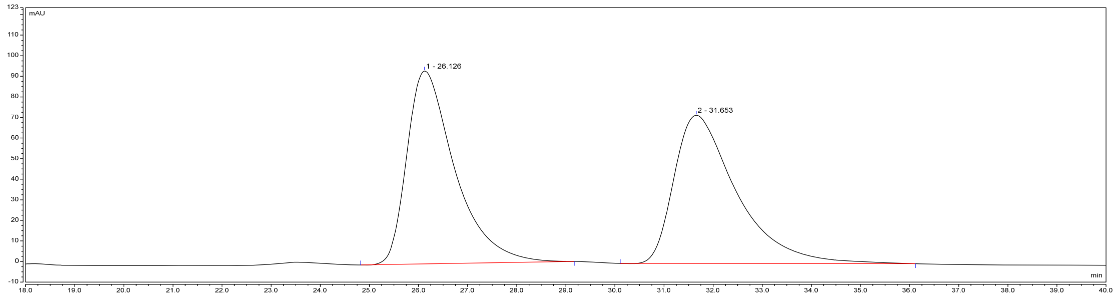

Entry	Retention Time	Area	Height	%Area
1	26.126	106.0119	93.66	49.88
2	31.653	106.5155	72.02	50.12

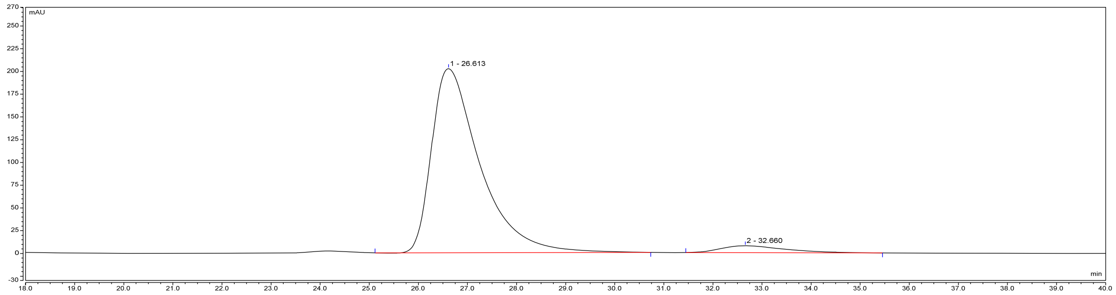

Entry	Retention Time	Area	Height	%Area
1	26.613	231.0167	202.37	95.23
2	32.660	11.5803	7.64	4.77

(*R*)-14-(2-bromophenyl)-2-(pyridin-2-yl)-1,15-diazadispiro[4.0.6^6.3^5]pentadeca-1,14-diene (50)

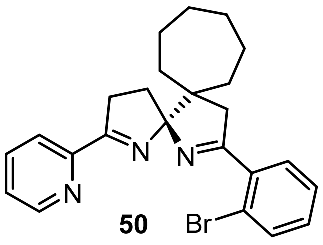

50

White solid; Mp 91.2−92.1 °C; 29.6 mg, 68% yield, 90% ee; $[\alpha]_D^{22}$ −34.8 (*c* 1.0, CH_3OH); **^{1}H NMR** (400 MHz, CD_3OD) δ 8.64 (d, *J* = 4.8 Hz, 1H), 8.16 (d, *J* = 7.9 Hz, 1H), 7.90 (t, *J* = 7.8 Hz, 1H), 7.65 (d, *J* = 7.9 Hz, 1H), 7.52 – 7.45 (m, 1H), 7.43 – 7.38 (m, 2H), 7.36 – 7.29 (m, 1H), 3.28 (dd, *J* = 8.2, 4.2 Hz, 2H), 3.23 (d, *J* = 8.8 Hz, 2H), 2.36 – 2.28 (m, 2H), 1.96 (dd, *J* = 14.0, 7.5 Hz, 1H), 1.83 (dd, *J* = 14.3, 6.5 Hz, 1H), 1.65 (m, 10H); **^{13}C NMR** (101 MHz, CD_3OD) δ 180.1, 177.3, 153.6, 150.2, 138.4, 138.1, 134.2, 132.1, 130.8, 128.5, 126.6, 123.7, 121.3, 113.1, 53.7, 51.6, 36.1, 35.9, 34.1, 30.2, 29.9, 29.4, 25.7, 24.7; **HRMS** (ESI) 436.1383 m/z (M + H^+), calc. for $C_{24}H_{27}BrN_3^+$ 436.1383.

The ee was determined by HPLC analysis: CHIRALPAK IE (4.6 mm i.d. x 250 mm); hexane/2-propanol = 80/20; flow rate 1.0 mL/min; 25 °C; 254 nm; retention time: 11.7 min (minor) and 13.6 min (major).

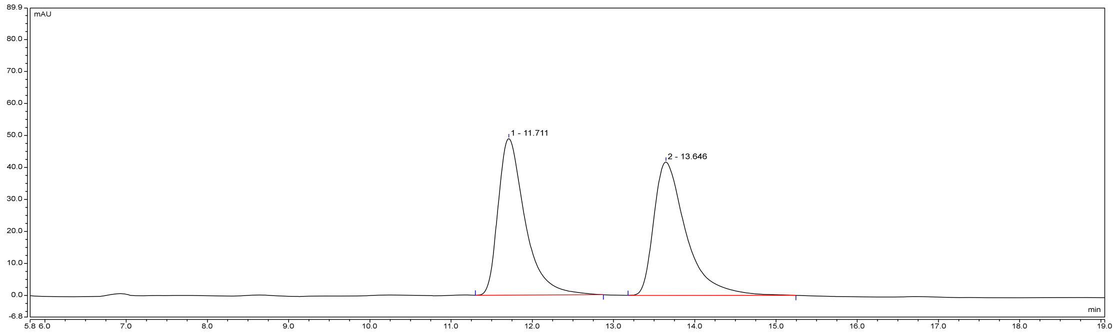

Entry	Retention Time	Area	Height	%Area
1	11.711	18.7634	48.88	49.46
2	13.646	19.1736	41.67	50.54

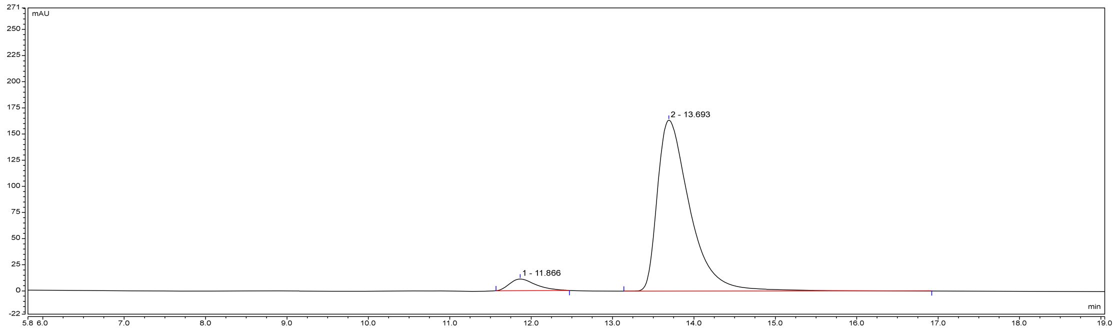

Entry	Retention Time	Area	Height	%Area
1	11.866	4.1697	11.15	5.25
2	13.693	75.2429	163.39	94.75

(*R*)-19-(2-bromophenyl)-2-(pyridin-2-yl)-1,20-diazadispiro[4.0.11^6.3^5]icosa-1,19-diene (51)

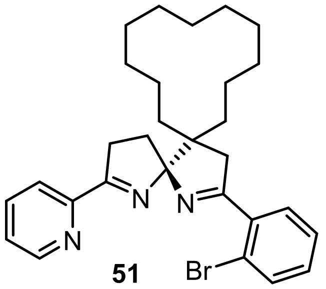

51

White solid; Mp 70.5−71.4 °C; 21.7 mg, 43% yield, 91% ee; $[\alpha]_D^{22}$ −21.6 (*c* 1.0, CH_3OH) ; ^{1}**H NMR** (600 MHz, CD_3OD) δ 8.64 (d, *J* = 4.7 Hz, 1H), 8.15 (d, *J* = 7.9 Hz, 1H), 7.96 – 7.78 (m, 1H), 7.64 (d, *J* = 8.0 Hz, 1H), 7.53 – 7.43 (m, 1H), 7.43 – 7.34 (m, 2H), 7.34 – 7.23 (m, 1H), 3.36 – 3.28 (m, 2H), 3.21 (d, *J* = 17.3 Hz, 1H), 3.10 (d, *J* = 17.3 Hz, 1H), 2.32 (dd, *J* = 8.0, 4.3 Hz, 2H), 1.83 (dd, *J* = 11.3, 6.3 Hz, 2H), 1.54 – 1.21 (m, 20H); ^{13}**C NMR** (151 MHz, CD_3OD) δ 179.5, 177.6, 153.6, 150.2, 138.2, 138.1, 134.1, 132.0, 130.7, 128.5, 126.6, 123.6, 121.3, 112.6, 52.4, 51.3, 35.4, 30.6, 30.3, 28.8, 27.9, 27.8, 27.0, 23.7, 23.6, 23.3, 22.8, 20.7, 20.4; **HRMS** (ESI) 506.2165 m/z (M + H^+), calc. for $C_{29}H_{37}BrN_3^+$ 506.2165.

The ee was determined by HPLC analysis: CHIRALPAK IC (4.6 mm i.d. x 250 mm); hexane/2-propanol = 80/20; flow rate 1.0 mL/min; 25 °C; 254 nm; retention time: 9.3 min (major) and 11.9 min (minor).

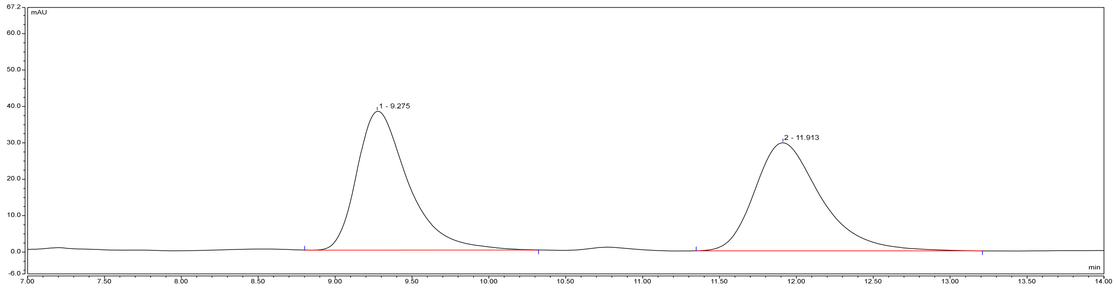

Entry	Retention Time	Area	Height	%Area
1	9.275	14.7538	38.13	50.01
2	11.913	14.7491	29.68	49.99

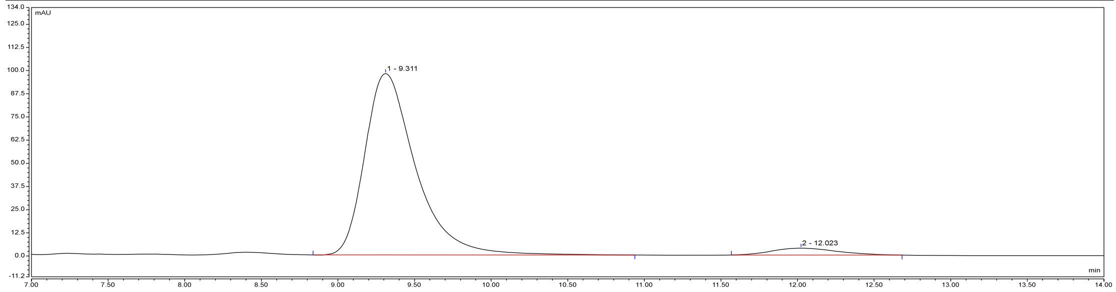

Entry	Retention Time	Area	Height	%Area
1	9.311	37.6599	97.87	95.38
2	12.023	1.8225	3.77	4.62

(*R*)-isobutyl-7-methoxy-2-(phenylethynyl)quinoline-1(2H)-carboxylate (54)

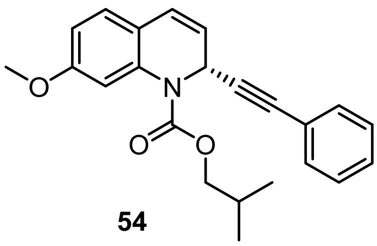

54

Colorless oil; 20.3 mg, 56% yield, 90% ee; $[\alpha]_D^{22}$ −38.8 (*c* 1.0, CH_3OH); **^{1}H NMR** (600 MHz, $CDCl_3$) δ 7.35 (s, 1H), 7.33 – 7.29 (m, 2H), 7.23 (m, 3H), 7.05 (d, *J* = 8.4 Hz, 1H), 6.67 (dd, *J* = 8.4, 2.5 Hz, 1H), 6.51 (d, *J* = 9.3 Hz, 1H), 6.08 (d, *J* = 5.5 Hz, 1H), 5.95 (dd, *J* = 9.1, 6.3 Hz, 1H), 4.12 – 3.98 (m, 2H), 3.82 (s, 3H), 2.08 – 1.99 (m, 1H), 1.00 (s, 3H), 0.99 (s, 3H); **^{13}C NMR** (151 MHz, $CDCl_3$) δ 159.4, 135.7, 132.0, 128.41, 128.2, 127.5, 125.7, 122.7), 119.9, 110.6, 86.2, 83.2, 77.3, 77.1, 76.9, 72.8, 55.5, 44.8, 28.0, 19.3; **HRMS** (ESI) 362.1750 m/z (M + H$^+$), calc. for $C_{23}H_{24}NO_3^+$ 362.1751.

The ee was determined by HPLC analysis: CHIRALPAK ODH (4.6 mm i.d. x 250 mm); hexane/2-propanol = 90/10; flow rate 1.0 mL/min; 25 °C; 254 nm; retention time: 6.3 min (major) and 8.0 min (minor).

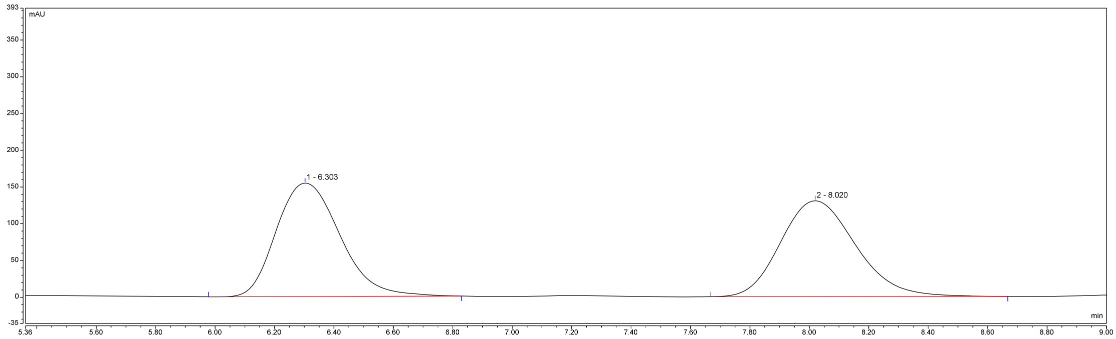

Entry	Retention Time	Area	Height	%Area
1	6.303	38.3051	154.30	50.12
2	8.020	38.1287	130.31	49.88

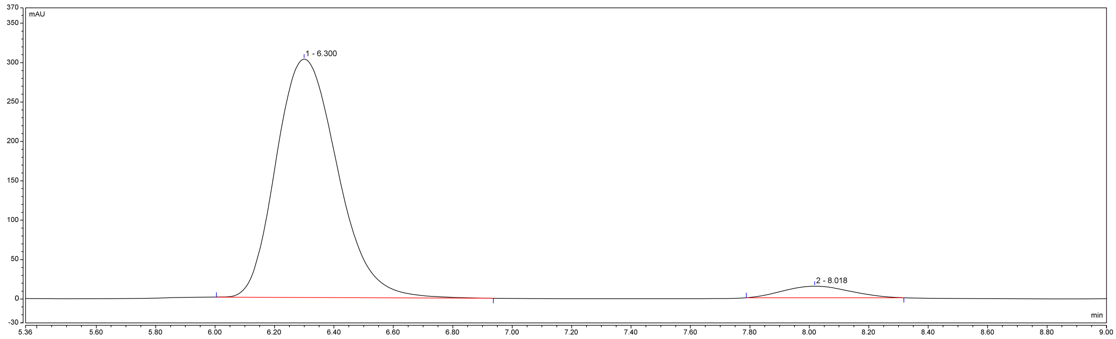

Entry	Retention Time	Area	Height	%Area
1	6.300	72.5252	302.57	95.02
2	8.018	3.8014	14.68	4.98

8. Copies of NMR spectra

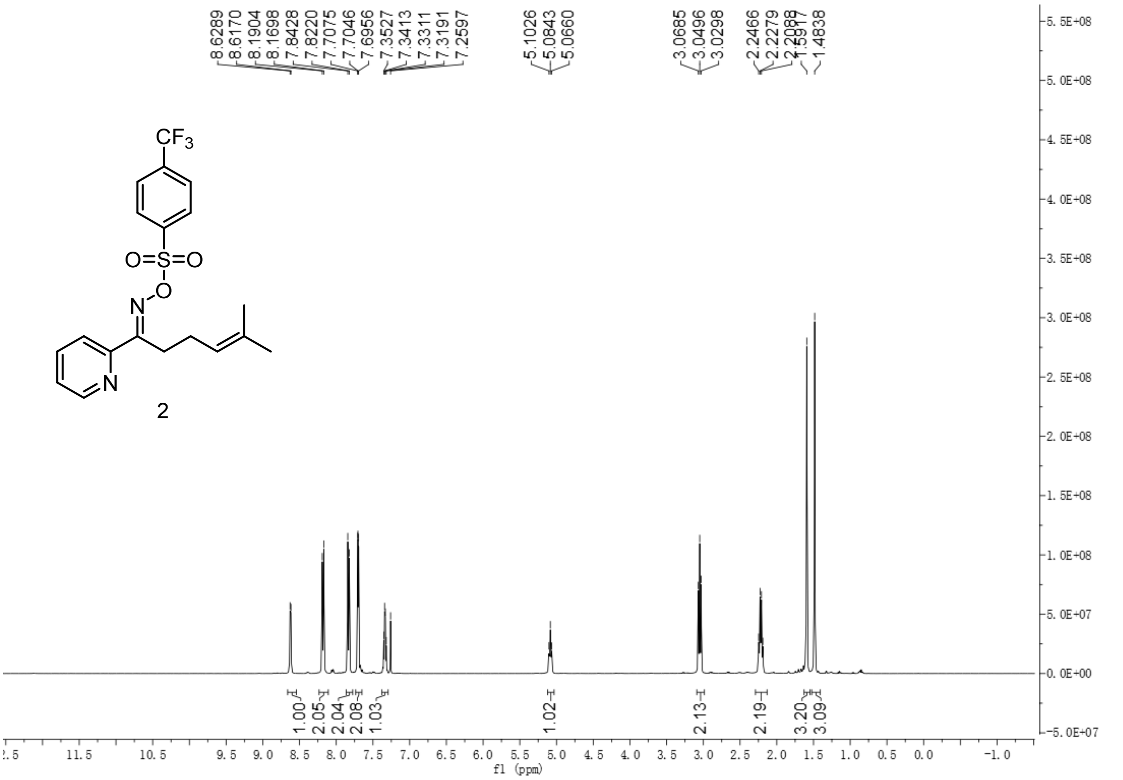

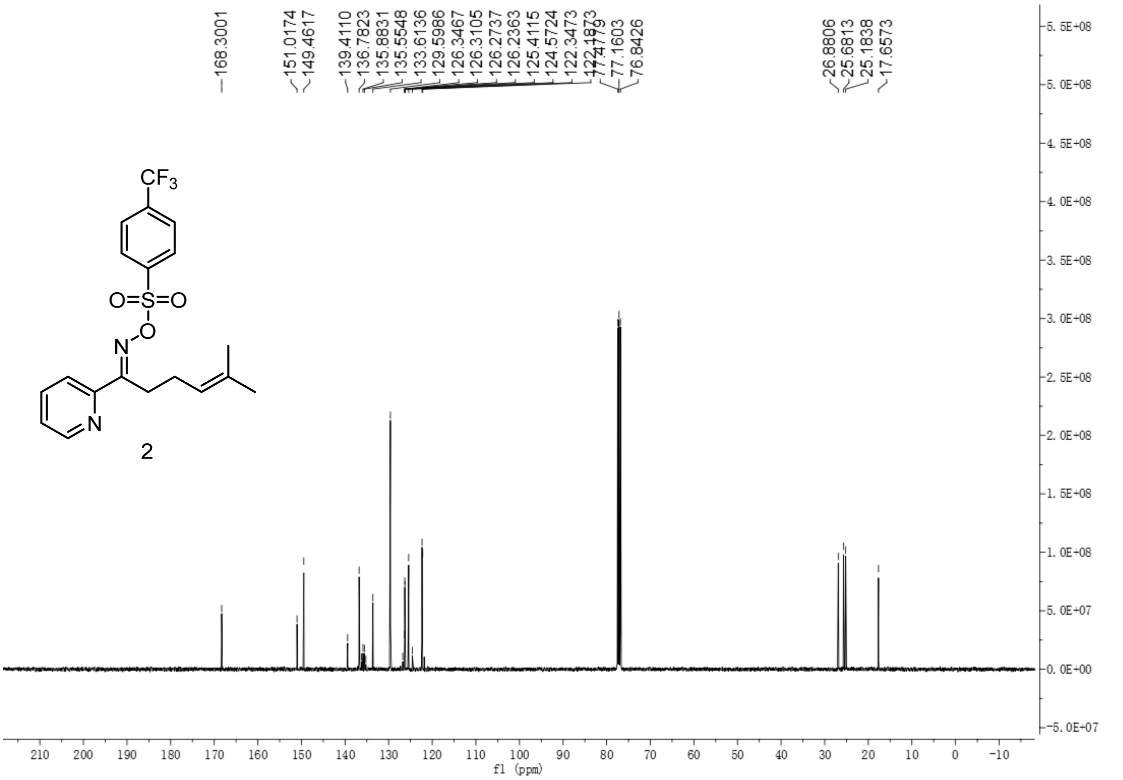

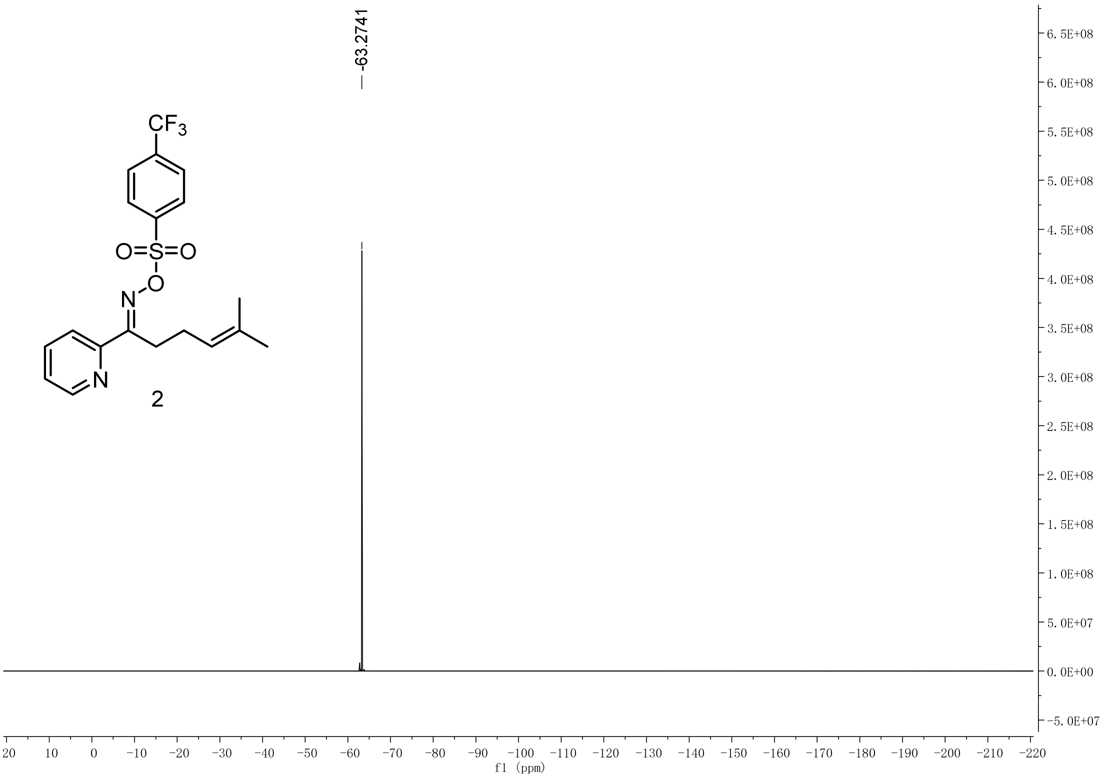
-63.2741
CF3
O=S=O
O
N
N
2
f1 (ppm)

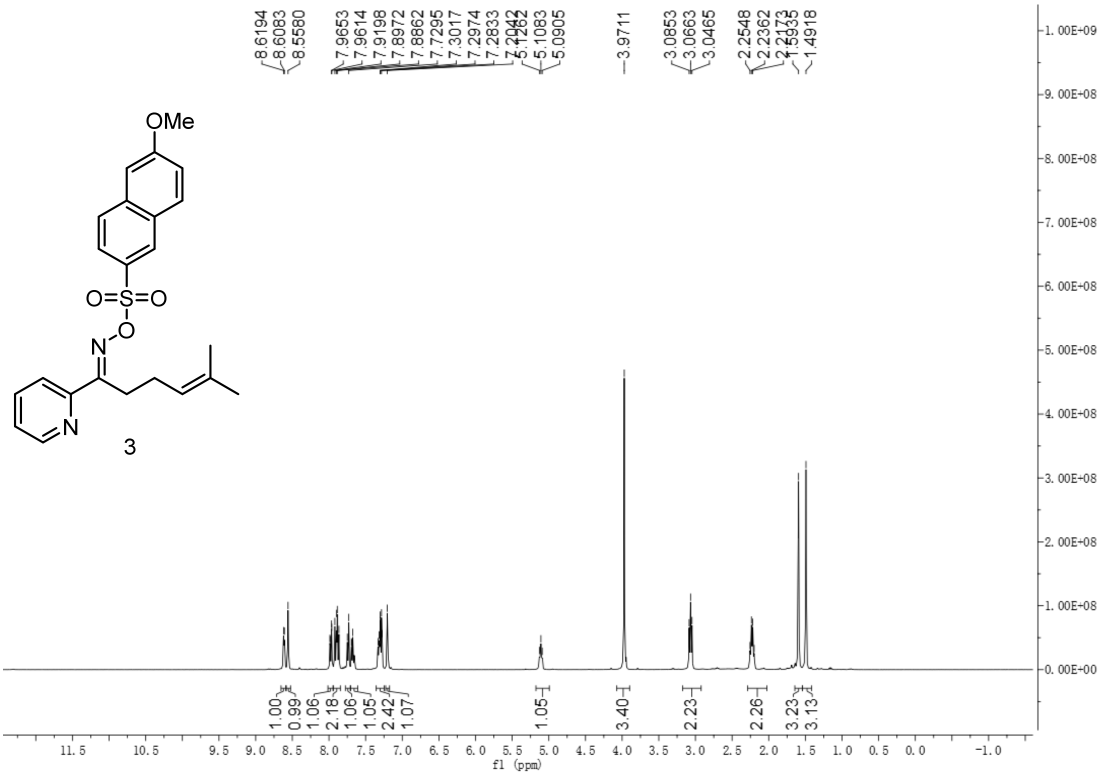
8.6194
8.6083
8.5580
7.9653
7.9614
7.9198
7.8972
7.8862
7.7295
7.3017
7.2974
7.2833
5.1083
5.0905
3.9711
3.0853
3.0663
3.0465
2.2548
2.2362
1.4918
OMe
O=S=O
O
N
N
3
1.00
0.99
1.06
2.18
1.06
1.05
2.42
1.07
1.05
3.40
2.23
2.26
3.23
3.13
f1 (ppm)

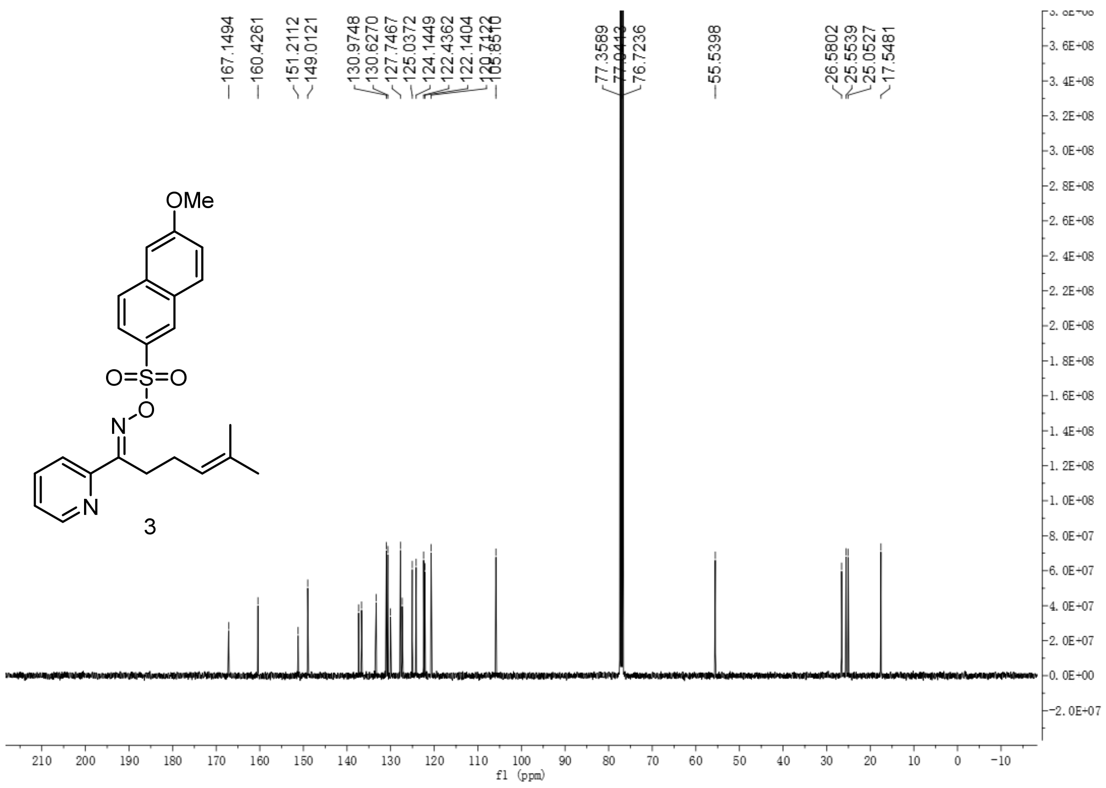

167.1494
160.4261
151.2112
149.0121
130.9748
130.6270
127.7467
125.0372
124.1449
122.4362
122.1404
120.7122
105.8510
77.3589
76.7236
55.5398
26.5802
25.5539
25.0527
17.5481
OMe
O=S=O
O
N
N
3
f1 (ppm)

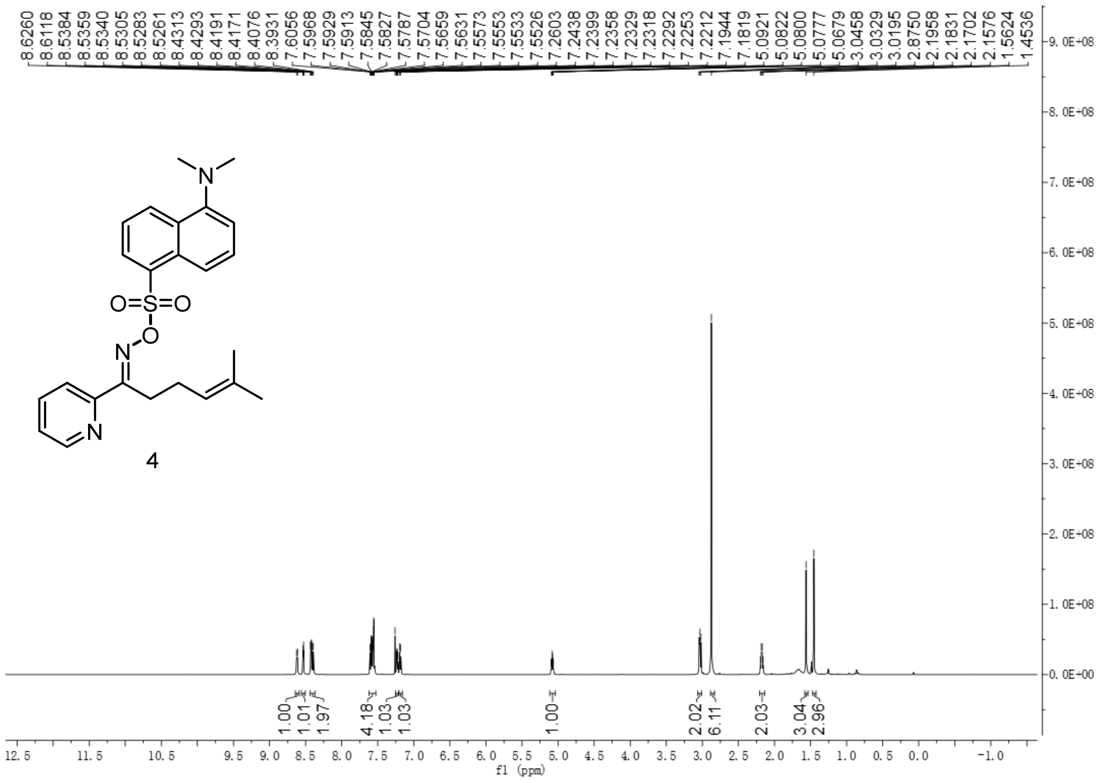

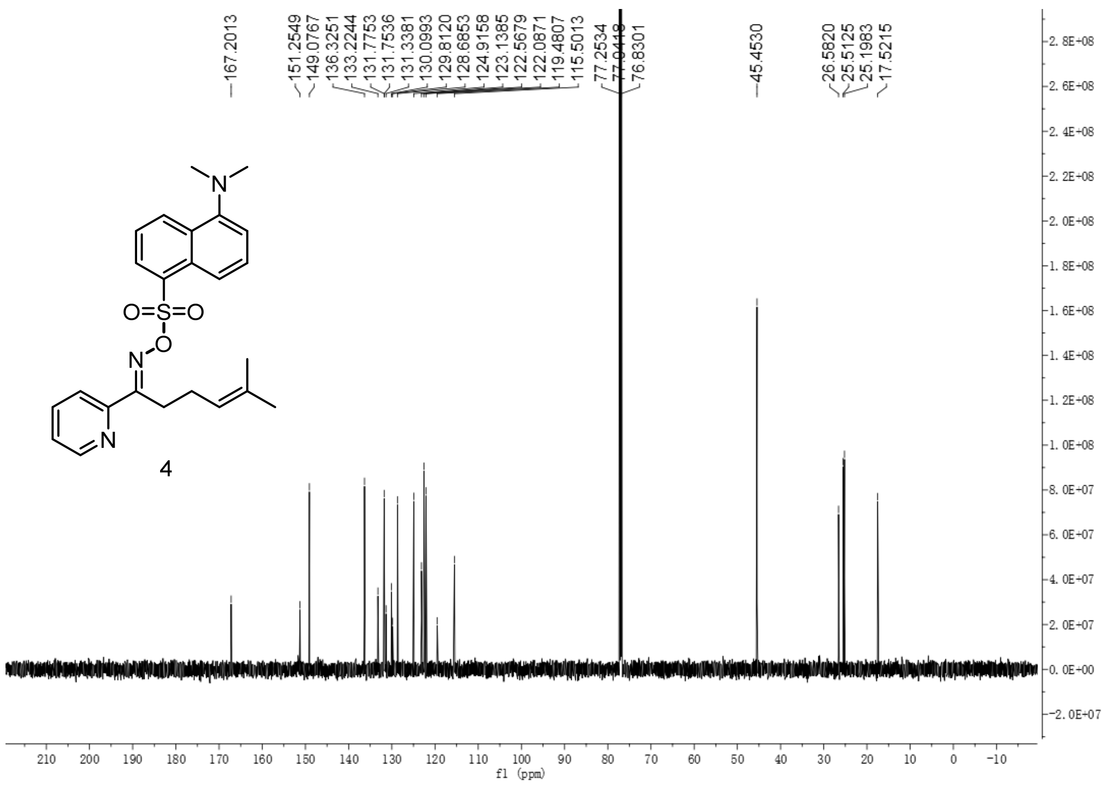

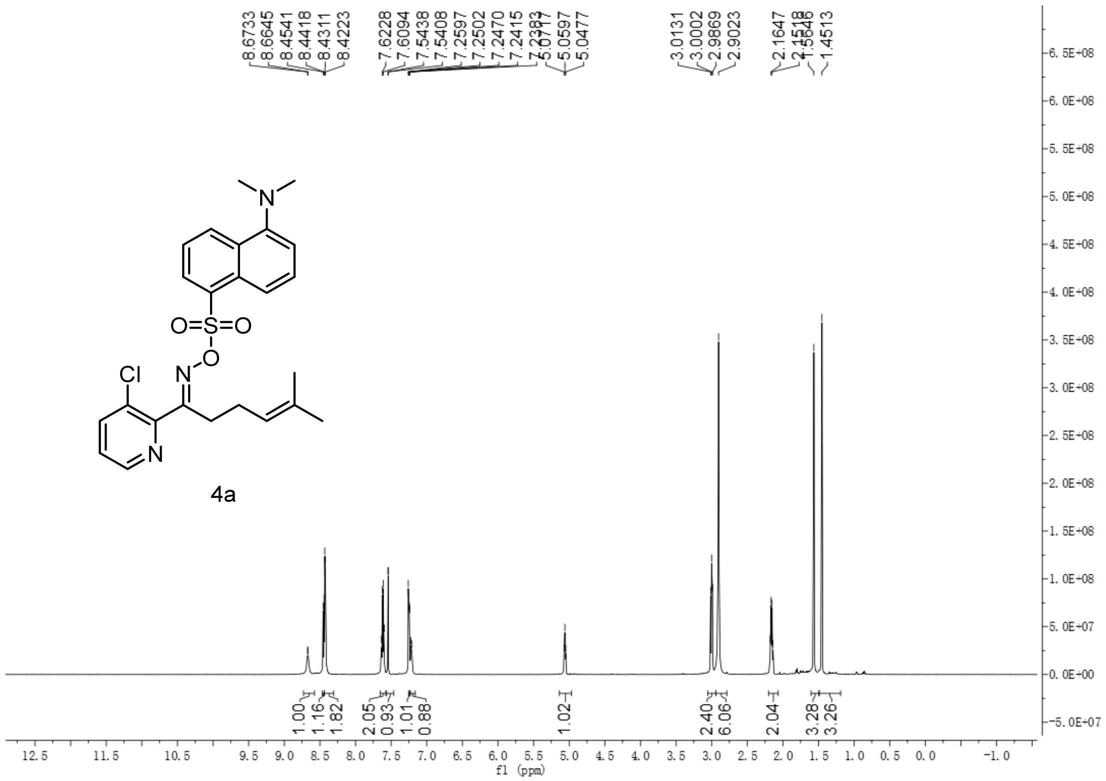
8.6733
8.6645
8.4541
8.4418
8.4311
8.4223
7.6228
7.6094
7.5438
7.5408
7.2597
7.2502
7.2470
7.2415
5.0597
5.0477
3.0131
3.0002
2.9869
2.9023
2.1647
1.4513
Cl
N
O=S=O
4a
1.00
1.16
1.82
2.05
0.93
1.01
0.88
1.02
2.40
6.06
2.04
3.28
3.26
f1 (ppm)

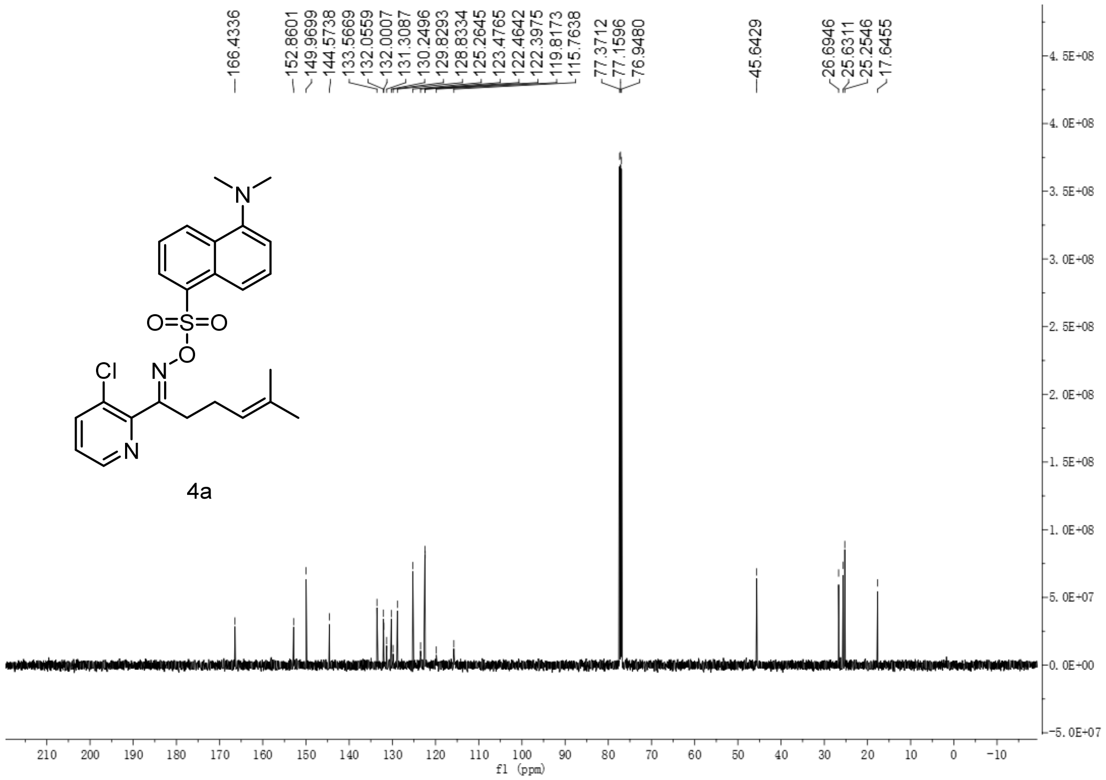
166.4336
152.8601
149.9699
144.5738
133.5669
132.0559
132.0007
131.3087
130.2496
129.8293
128.8334
125.2645
123.4765
122.4642
122.3975
119.8173
115.7638
77.3712
77.1596
76.9480
45.6429
26.6946
25.6311
25.2546
17.6455
Cl
N
O=S=O
4a
f1 (ppm)

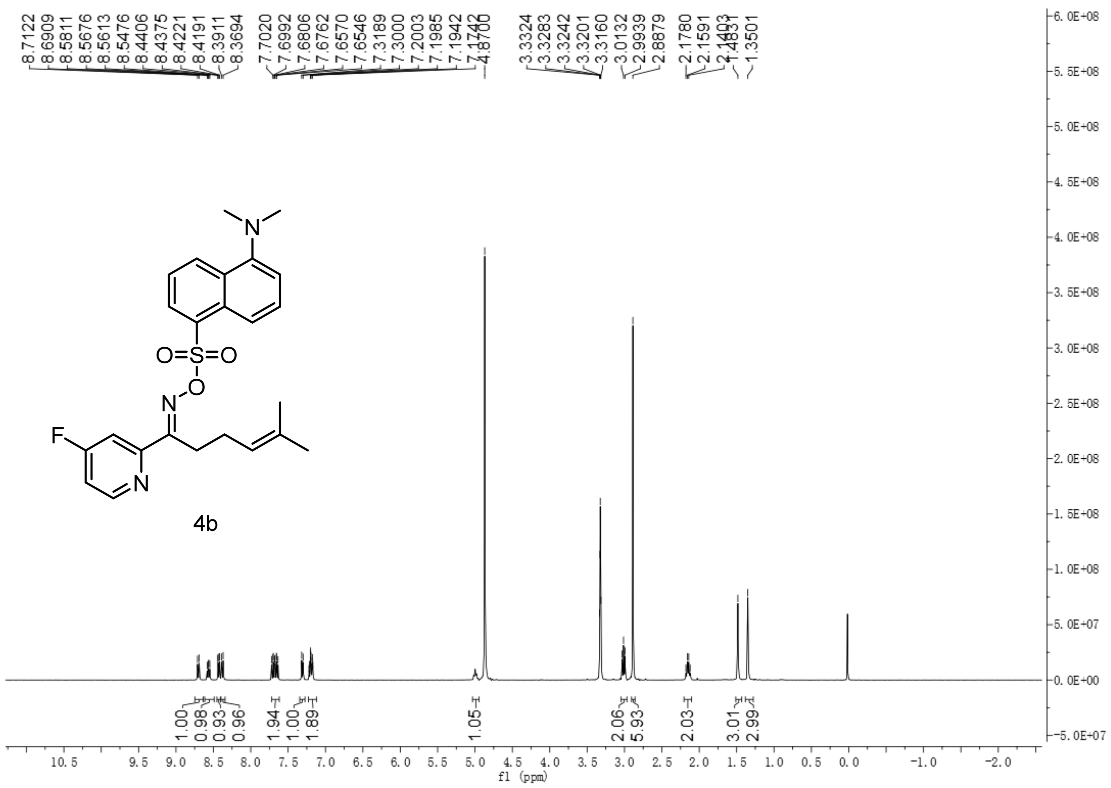

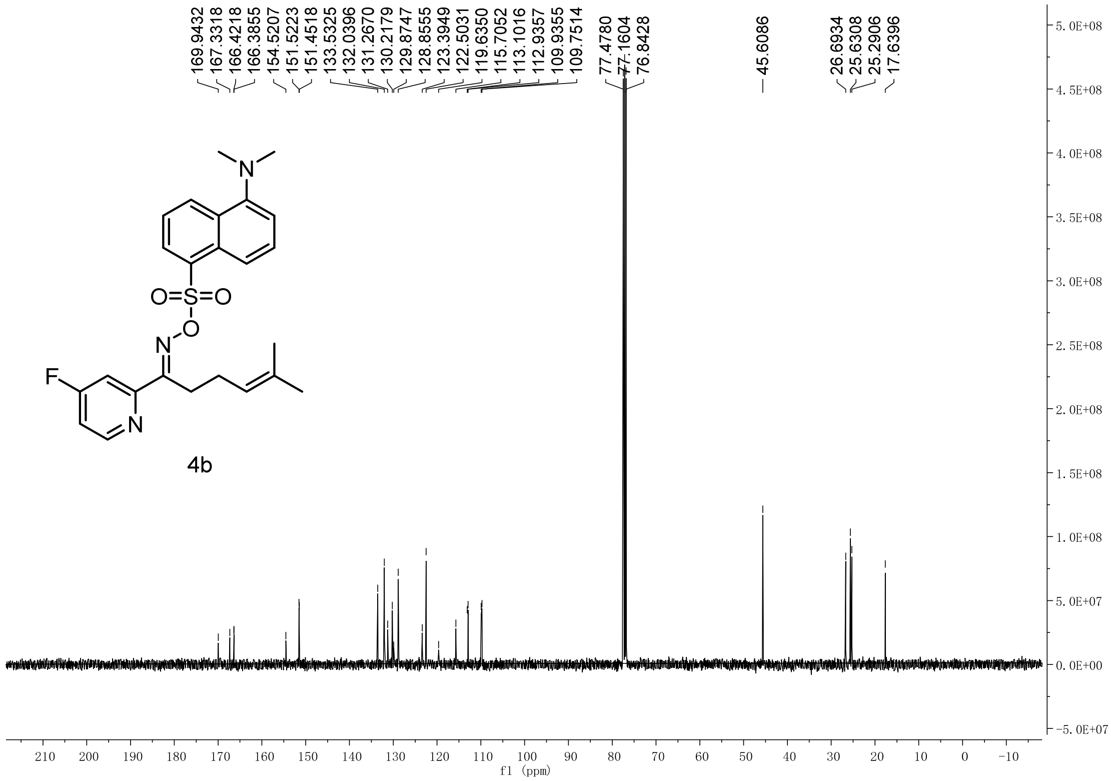

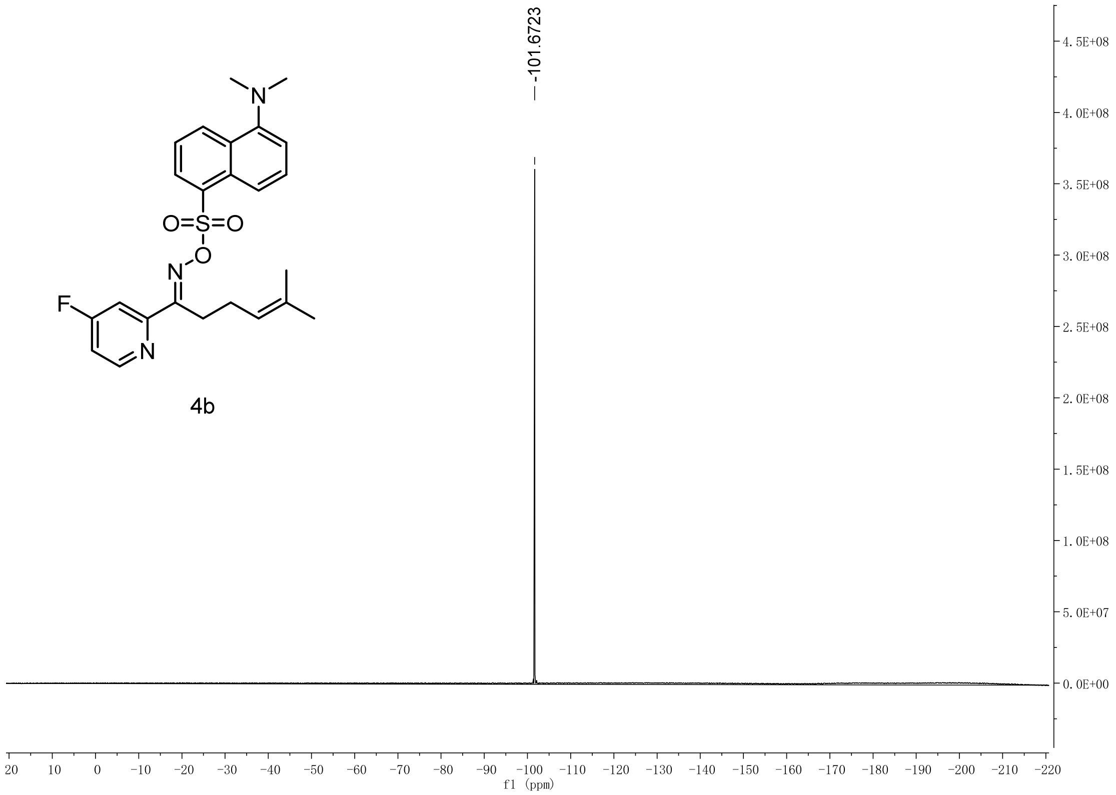
-101.6723
4b
f1 (ppm)

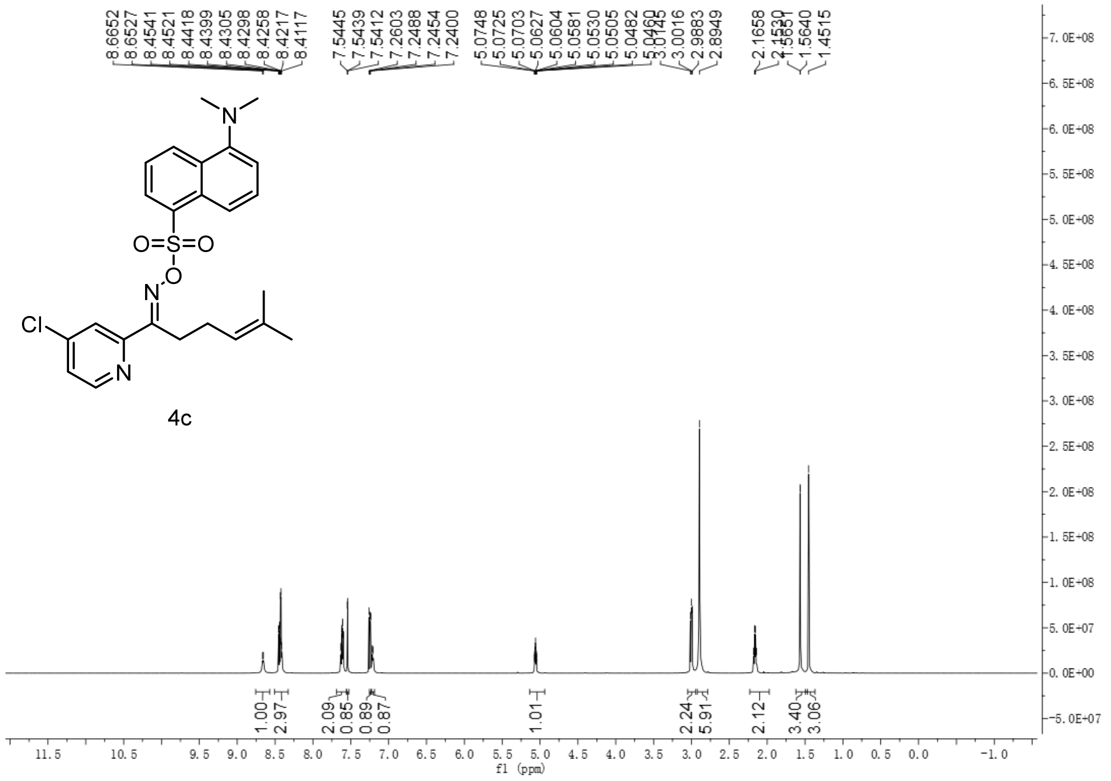
8.6652
8.6527
8.4541
8.4521
8.4418
8.4399
8.4305
8.4298
8.4258
8.4217
8.4117
7.5445
7.5439
7.5412
7.2603
7.2488
7.2454
7.2400
5.0748
5.0725
5.0703
5.0627
5.0604
5.0581
5.0530
5.0505
5.0482
3.0016
2.9883
2.8949
2.1658
1.5640
1.4515
4c
1.00
2.97
2.09
0.85
0.89
0.87
1.01
2.24
5.91
2.12
3.40
3.06
f1 (ppm)

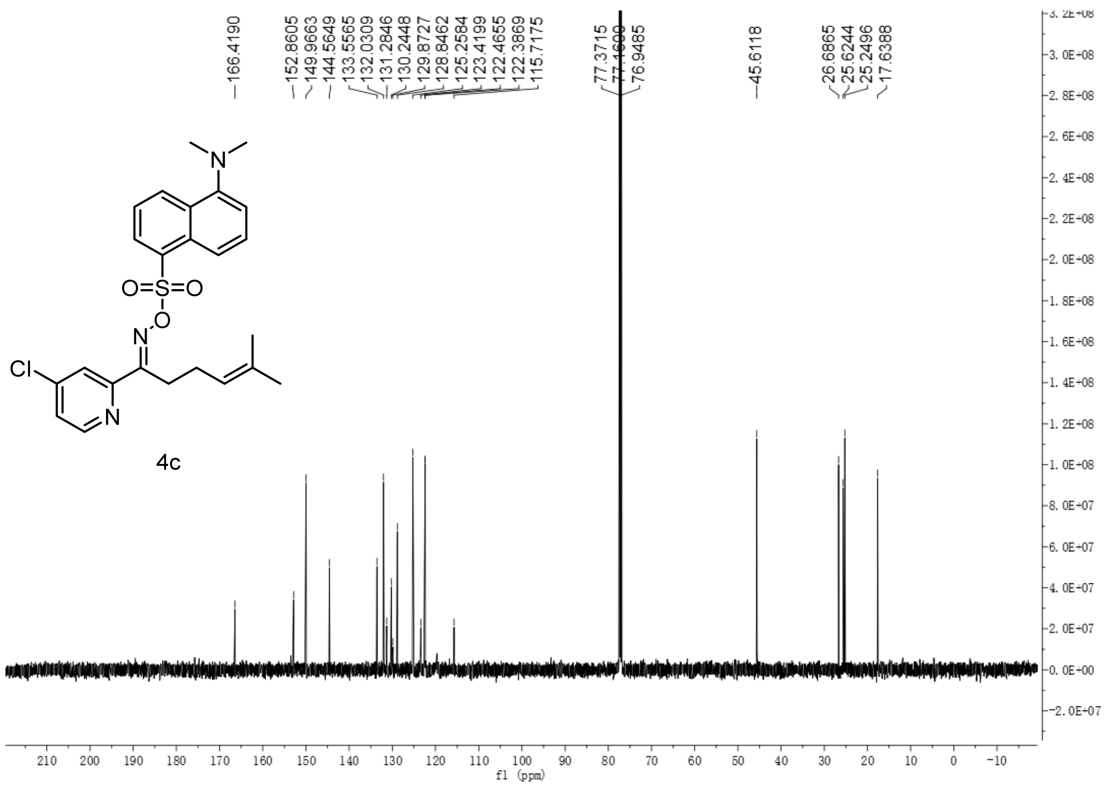

166.4190
152.8605
149.9663
144.5649
133.5565
132.0309
131.2846
130.2448
129.8727
128.8462
125.2584
123.4199
122.4655
122.3869
115.7175
77.3715
76.9485
45.6118
26.6865
25.6244
25.2496
17.6388
Cl
N
N
O
O=S=O
4c
fl (ppm)

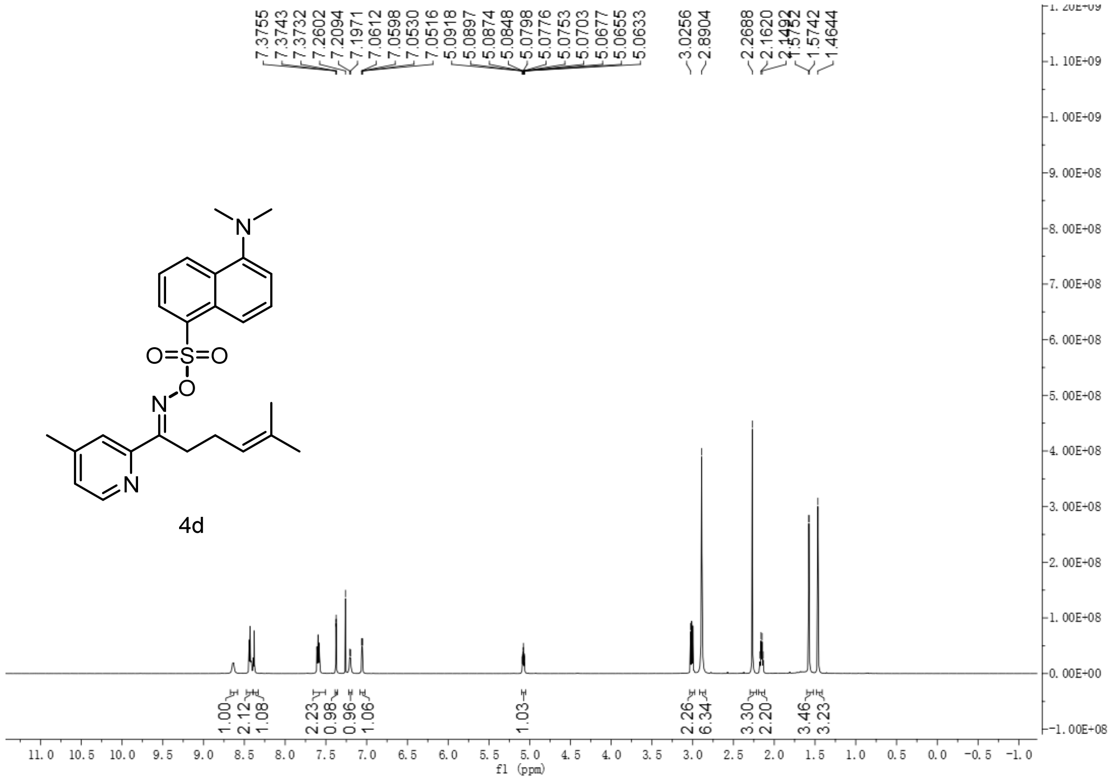

7.3755
7.3743
7.3732
7.2602
7.2094
7.1971
7.0612
7.0598
7.0530
7.0516
5.0918
5.0897
5.0874
5.0848
5.0798
5.0776
5.0753
5.0703
5.0677
5.0655
5.0633
3.0256
2.8904
2.2688
2.1620
1.5742
1.4644
N
N
O
O=S=O
4d
1.00
2.12
1.08
2.23
0.98
0.96
1.06
1.03
2.26
6.34
3.30
2.20
3.46
3.23
fl (ppm)

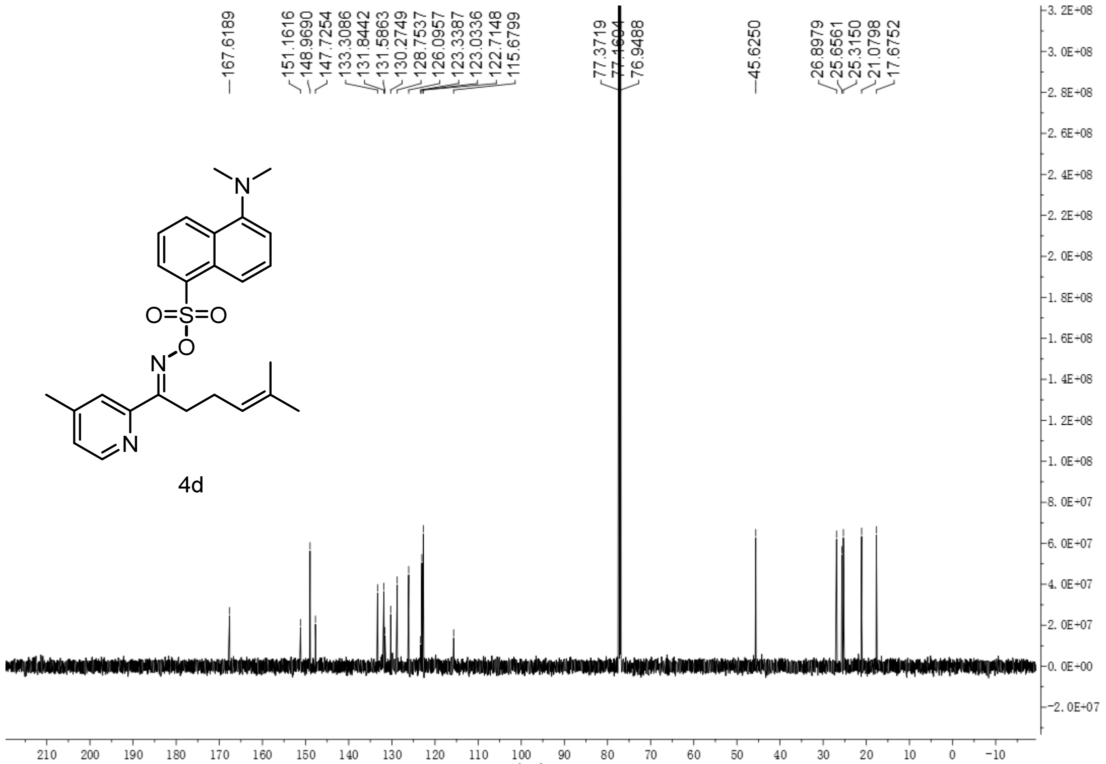

167.6189
151.1616
148.9690
147.7254
133.3086
131.8442
131.5863
130.2749
128.7537
126.0957
123.3387
123.0336
122.7148
115.6799
77.3719
76.9488
45.6250
26.8979
25.6561
25.3150
21.0798
17.6752
4d
f1 (ppm)

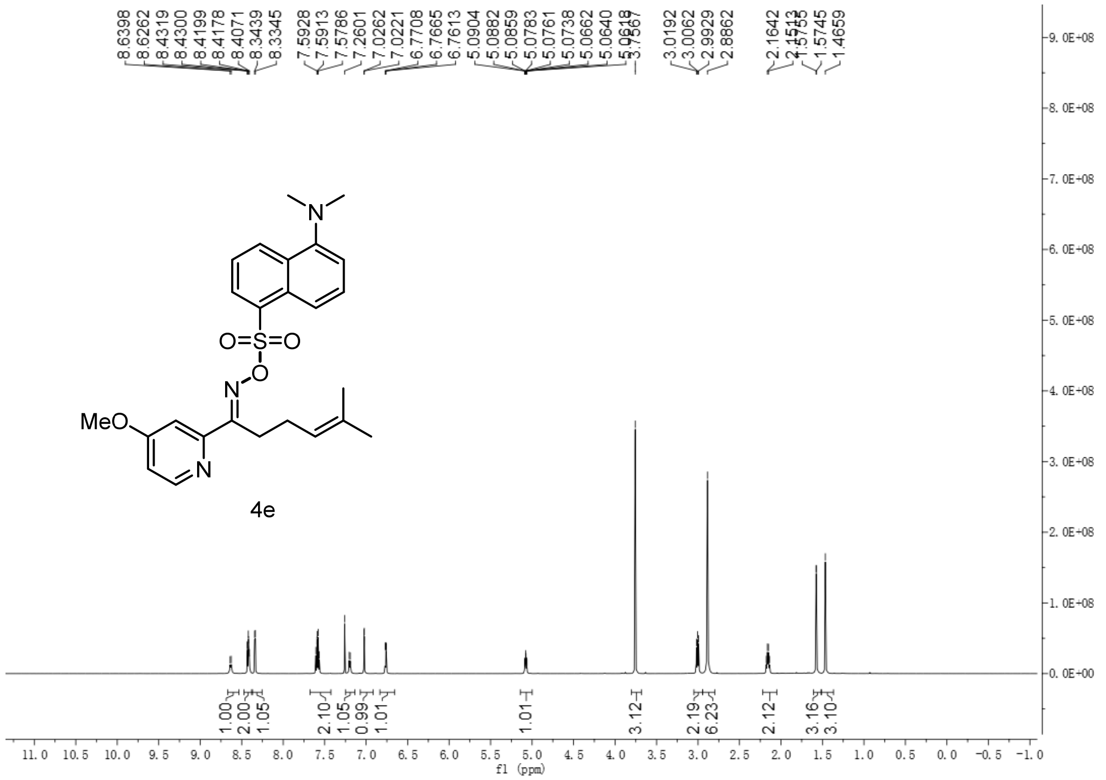

8.6398
8.6262
8.4319
8.4300
8.4199
8.4178
8.4071
8.3439
8.3345
7.5928
7.5913
7.5786
7.2601
7.0262
7.0221
6.7708
6.7665
6.7613
5.0904
5.0882
5.0859
5.0783
5.0761
5.0738
5.0662
5.0640
3.0192
3.0062
2.9929
2.8862
2.1642
1.5745
1.4659
MeO
4e
f1 (ppm)

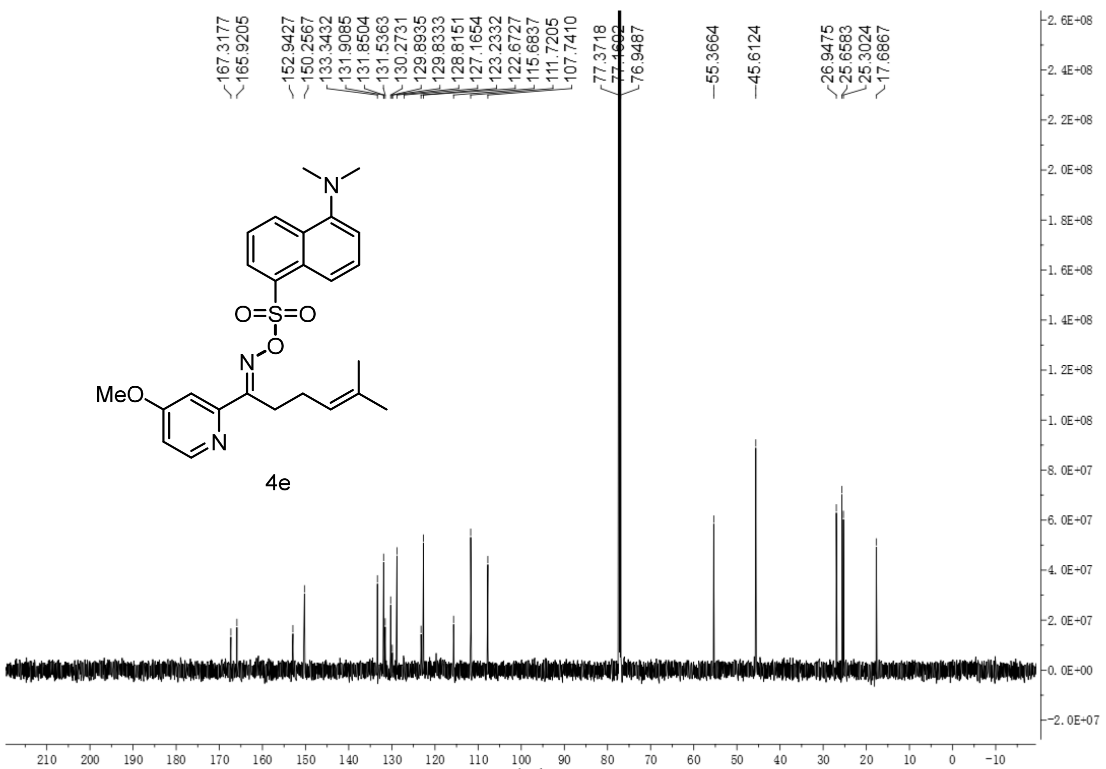
167.3177
165.9205
152.9427
150.2567
133.3432
131.9085
131.8504
131.5363
130.2731
129.8935
129.8333
128.8151
127.1654
123.2332
122.6727
115.6837
111.7205
107.7410
77.3718
76.9487
55.3664
45.6124
26.9475
25.6583
25.3024
17.6867
MeO
N
O
S
4e
f1 (ppm)

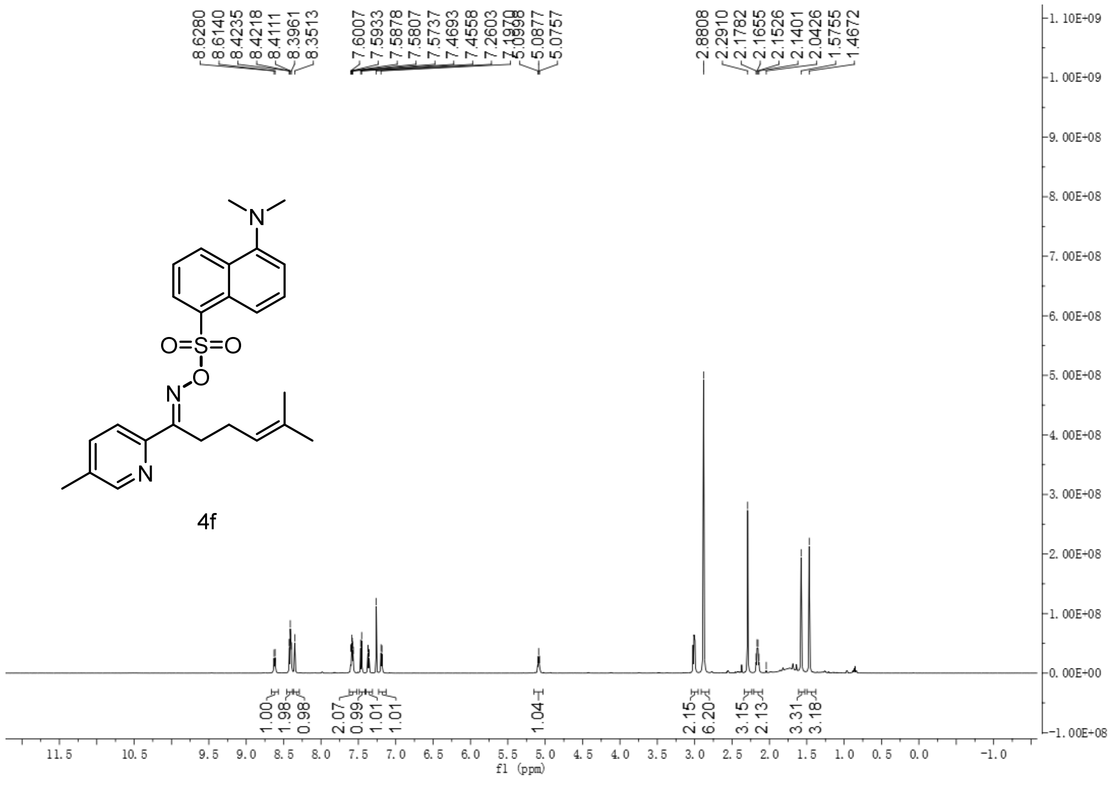
8.6280
8.6140
8.4235
8.4218
8.4111
8.3961
8.3513
7.6007
7.5933
7.5878
7.5807
7.5737
7.4693
7.4558
7.2603
5.0877
5.0757
2.8808
2.2910
2.1782
2.1655
2.1526
2.1401
2.0426
1.5755
1.4672
1.00
1.98
0.98
2.07
0.99
1.01
1.01
1.04
2.15
6.20
3.15
2.13
3.31
3.18
N
O
S
4f
f1 (ppm)

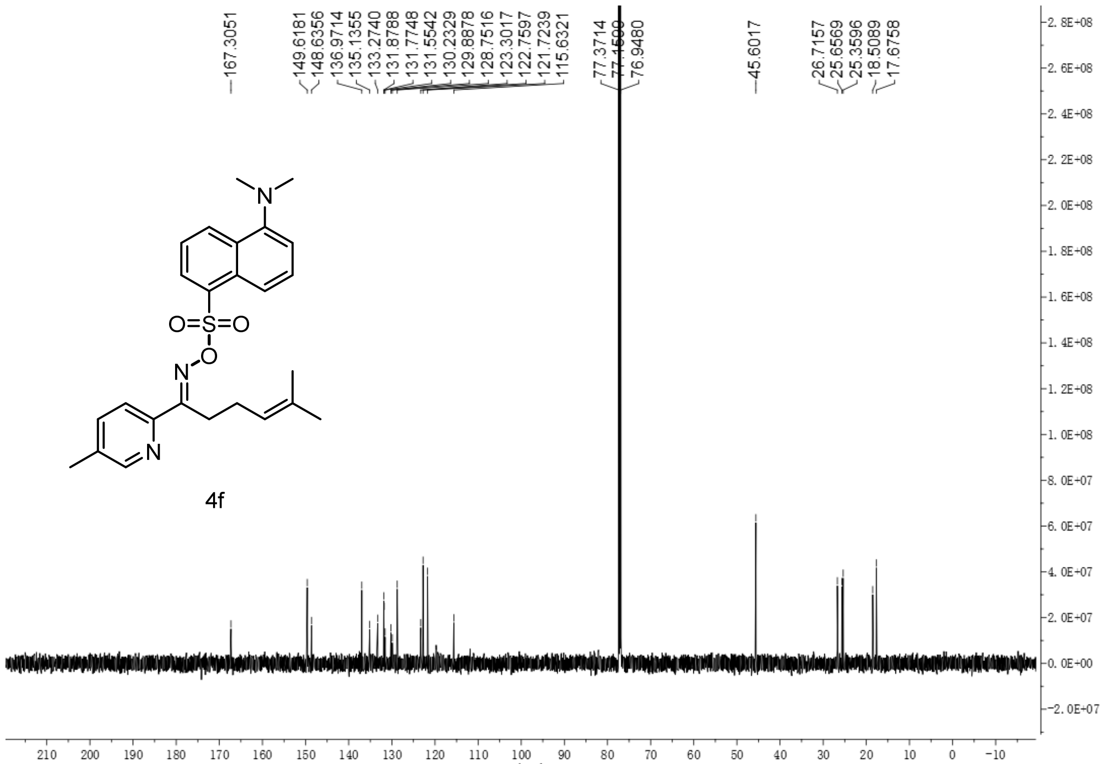

167.3051
149.6181
148.6356
136.9714
135.1355
133.2740
131.8788
131.7748
131.5542
130.2329
129.8878
128.7516
123.3017
122.7597
121.7239
115.6321
77.3714
76.9480
45.6017
26.7157
25.6569
25.3596
18.5089
17.6758
4f
f1 (ppm)

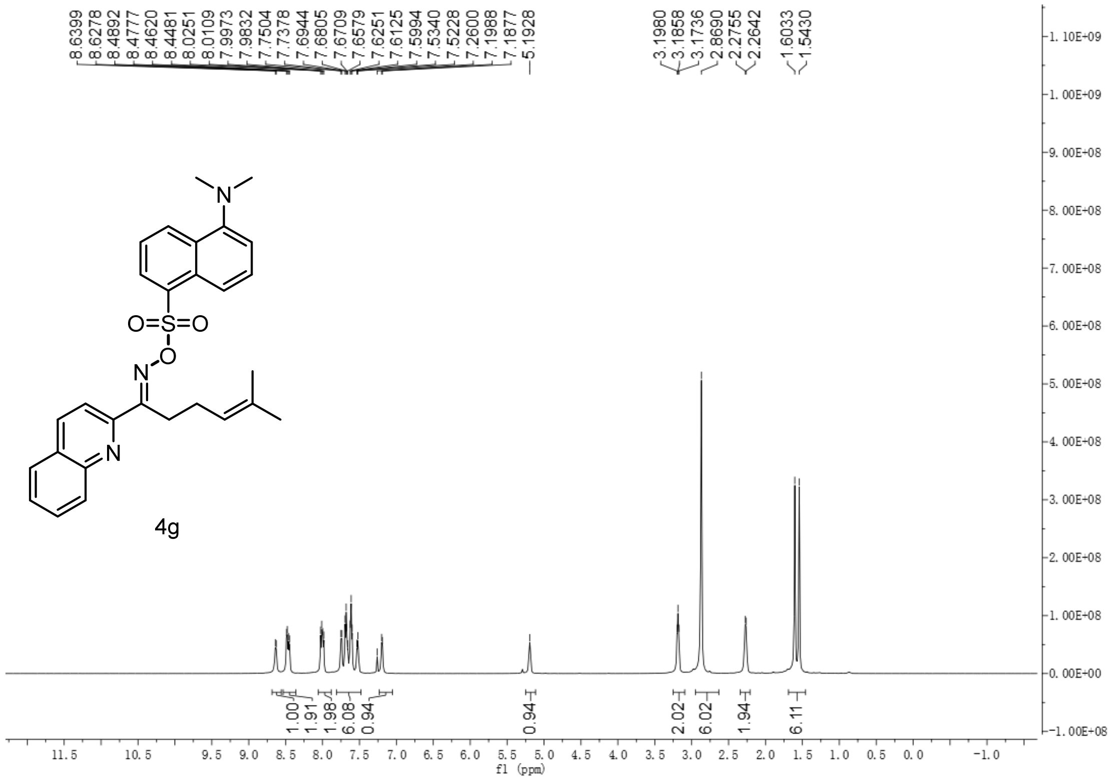

8.6399
8.6278
8.4892
8.4777
8.4620
8.4481
8.0251
8.0109
7.9973
7.9832
7.7504
7.7378
7.6944
7.6805
7.6709
7.6579
7.6251
7.6125
7.5994
7.5340
7.5228
7.2600
7.1988
7.1877
5.1928
3.1980
3.1858
3.1736
2.8690
2.2755
2.2642
1.6033
1.5430
4g
1.00
1.91
1.98
6.08
0.94
0.94
2.02
6.02
1.94
6.11
f1 (ppm)

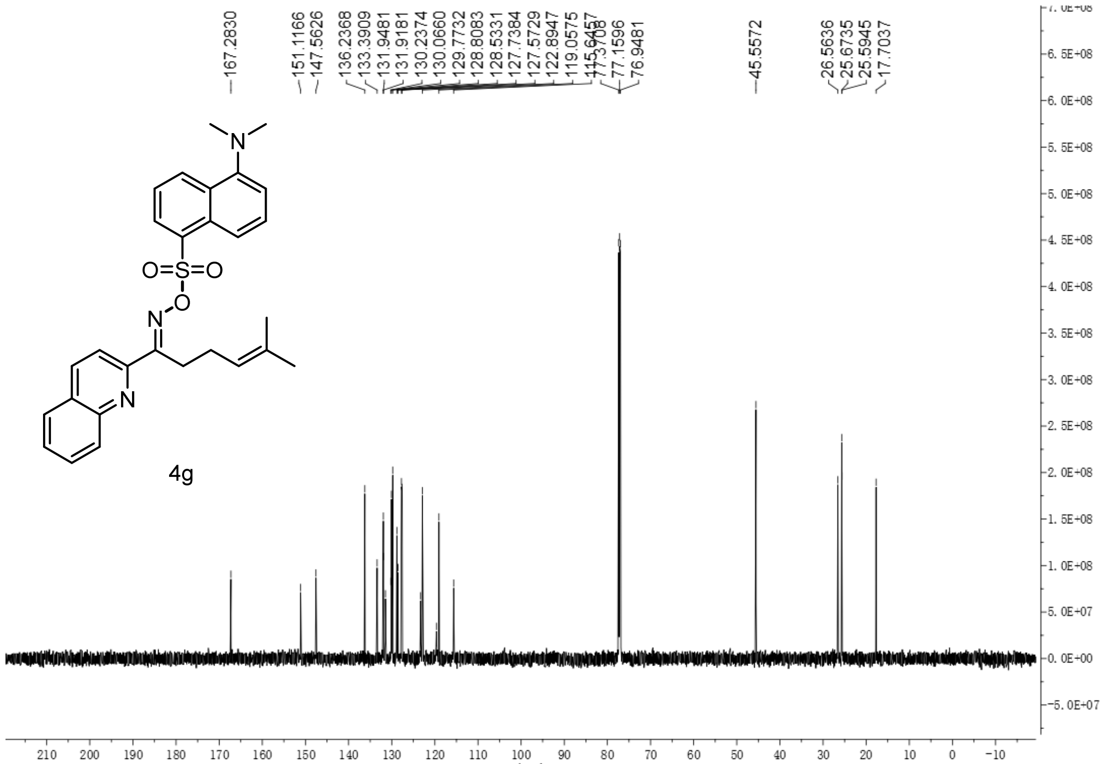
167.2830
151.1166
147.5626
136.2368
133.3909
131.9481
131.9181
130.2374
130.0660
129.7732
128.8083
128.5331
127.7384
127.5729
122.8947
119.0575
77.1596
76.9481
45.5572
26.5636
25.6735
25.5945
17.7037
O=S=O
N
O
4g
f1 (ppm)

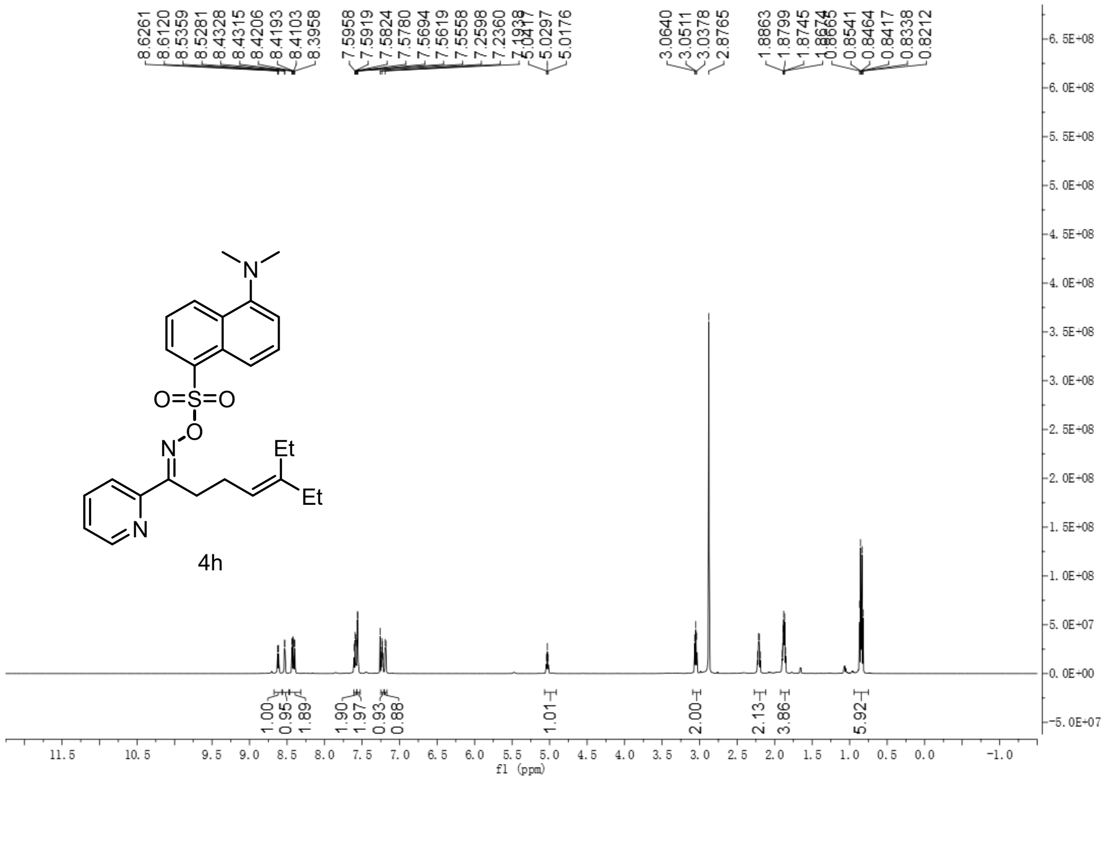
8.6261
8.6120
8.5359
8.5281
8.4328
8.4315
8.4206
8.4193
8.4103
8.3958
7.5958
7.5919
7.5824
7.5780
7.5694
7.5619
7.5558
7.2598
7.2360
5.0297
5.0176
3.0640
3.0511
3.0378
2.8765
1.8863
1.8799
1.8745
0.8541
0.8464
0.8417
0.8338
0.8212
O=S=O
N
O
Et
Et
4h
1.00
0.95
1.89
1.90
1.97
0.93
0.88
1.01
2.00
2.13
3.86
5.92
f1 (ppm)

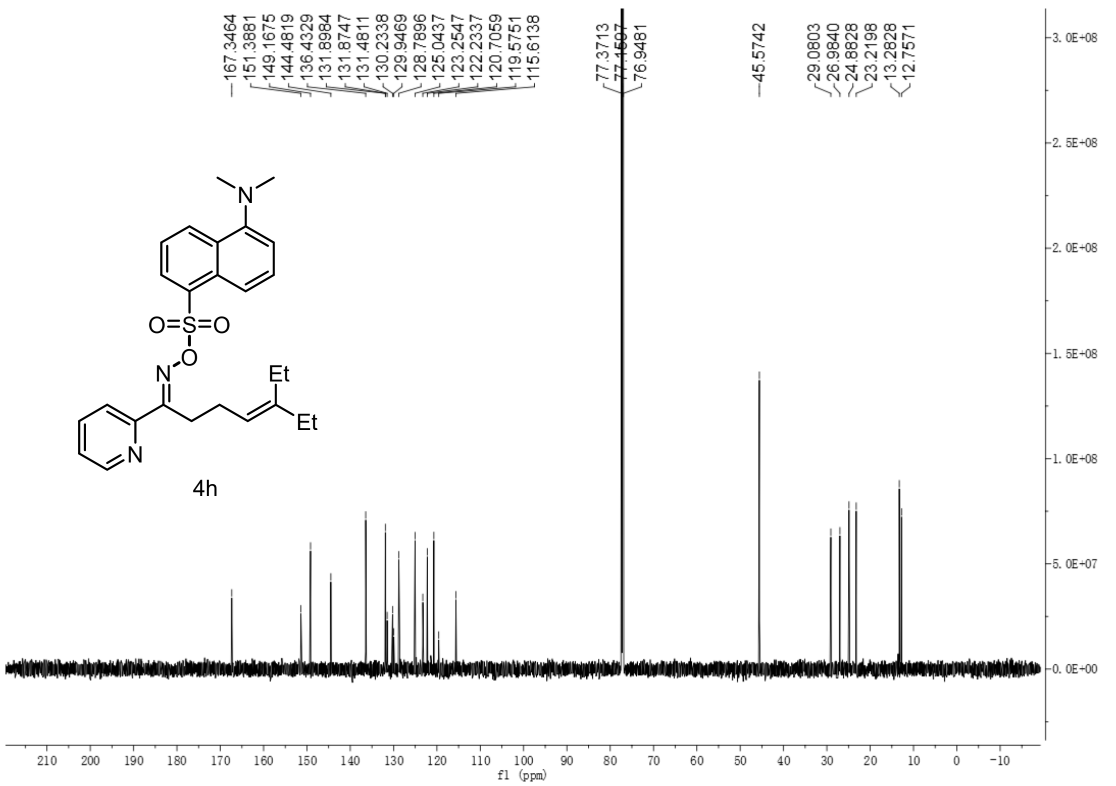

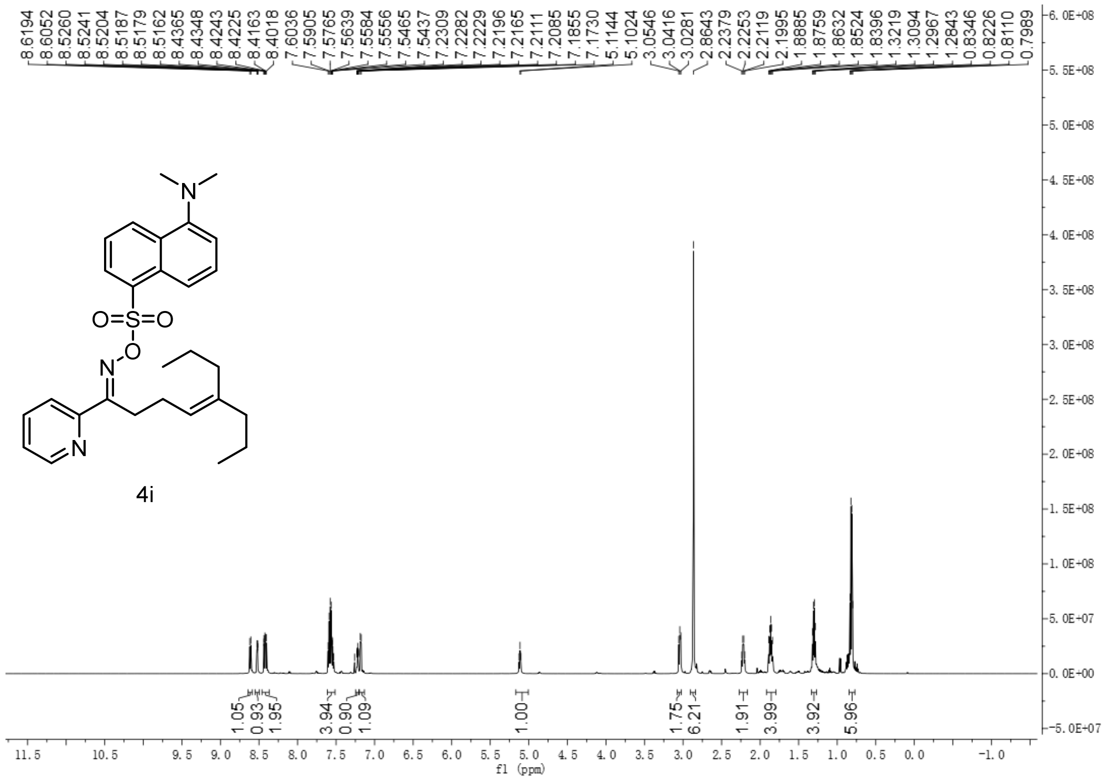

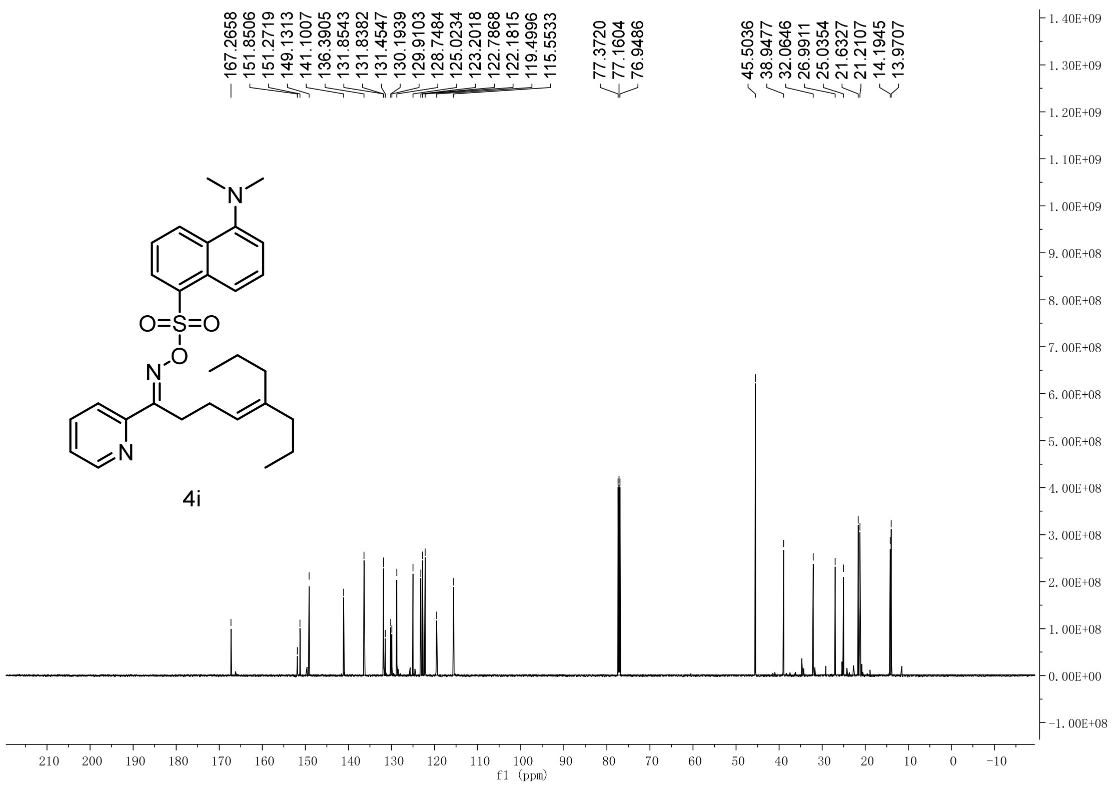

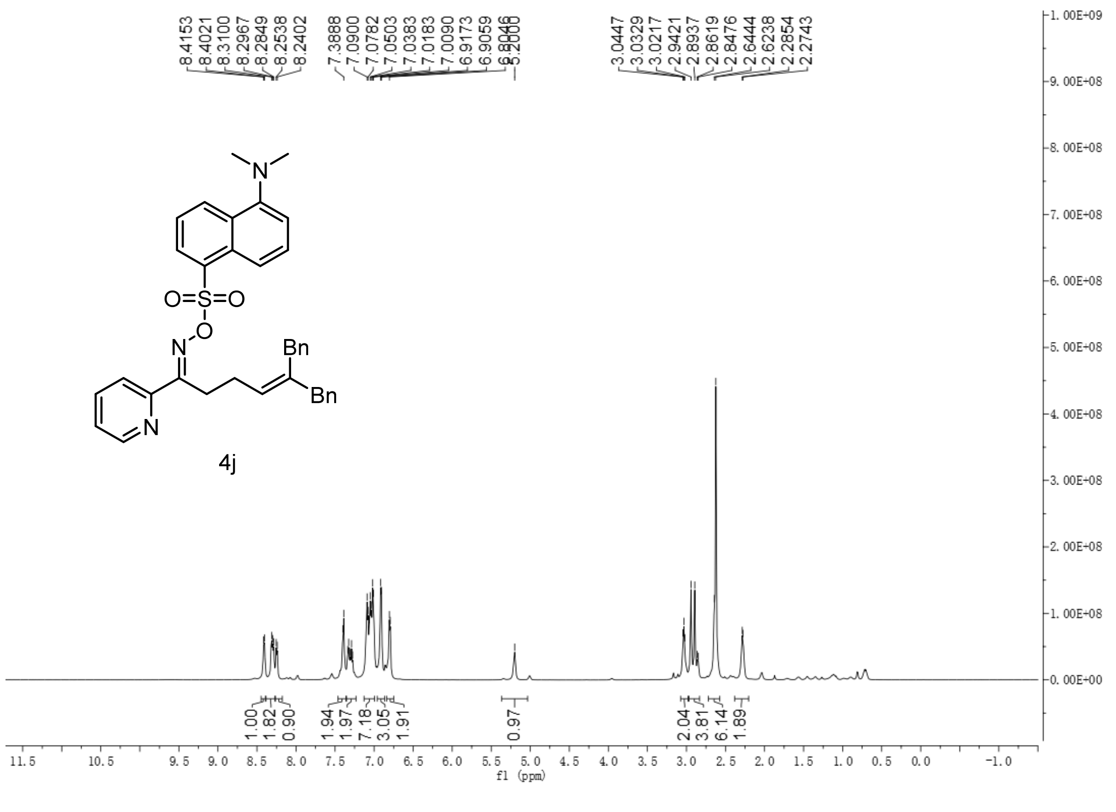

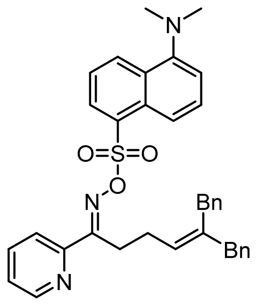

4j

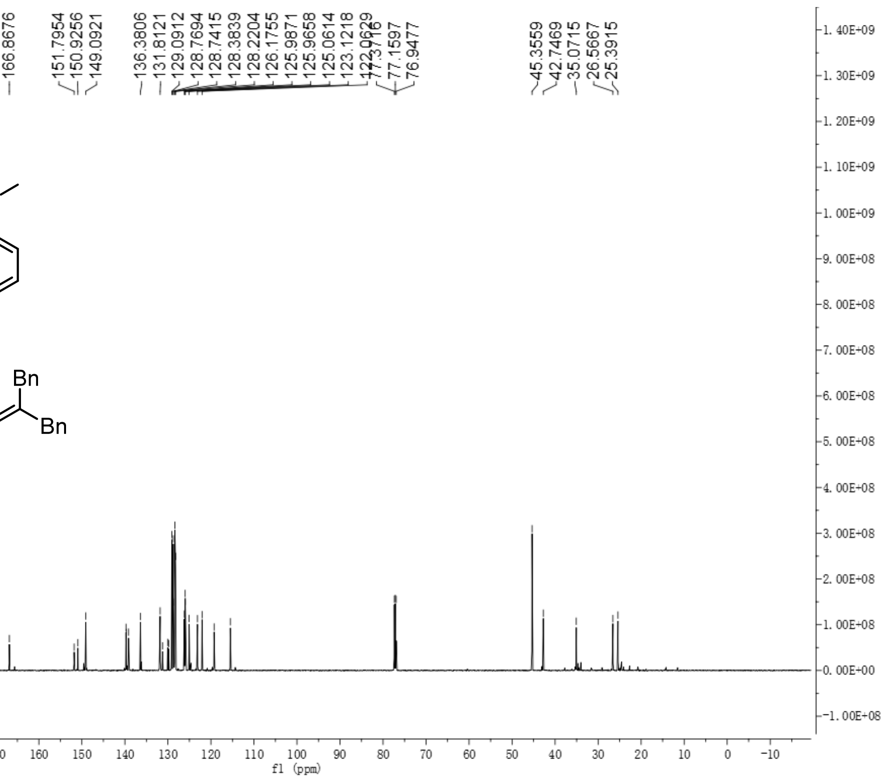

166.8676
151.7954
150.9256
149.0921
136.3806
131.8121
129.0912
128.7694
128.7415
128.3839
128.2204
126.1755
125.9871
125.9658
125.0614
123.1218
122.0629
77.1597
76.9477
45.3559
42.7469
35.0715
26.5667
25.3915
fl (ppm)

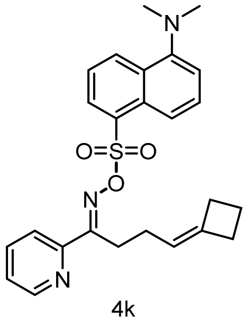

4k

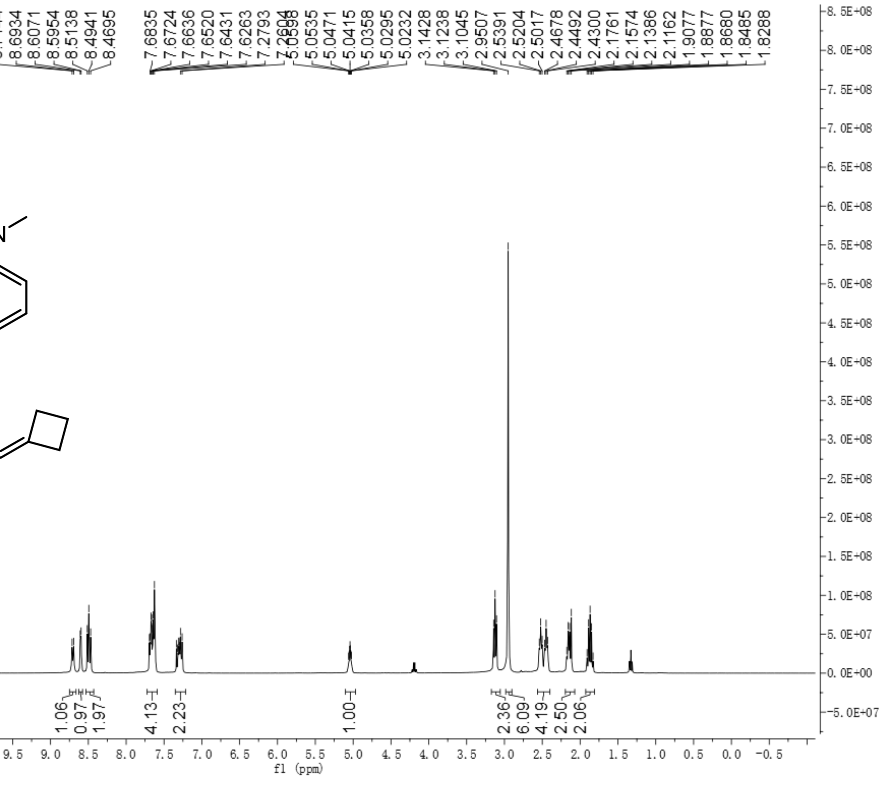

8.7144
8.6934
8.6071
8.5954
8.5138
8.4941
8.4695
7.6835
7.6724
7.6636
7.6520
7.6431
7.6263
7.2793
7.2604
5.0535
5.0471
5.0415
5.0358
5.0295
5.0232
3.1428
3.1238
3.1045
2.9507
2.5391
2.5204
2.5017
2.4678
2.4492
2.4300
2.1761
2.1574
2.1386
2.1162
1.9077
1.8877
1.8680
1.8485
1.8288
1.06
0.97
1.97
4.13
2.23
1.00
2.36
6.09
4.19
2.50
2.06
fl (ppm)

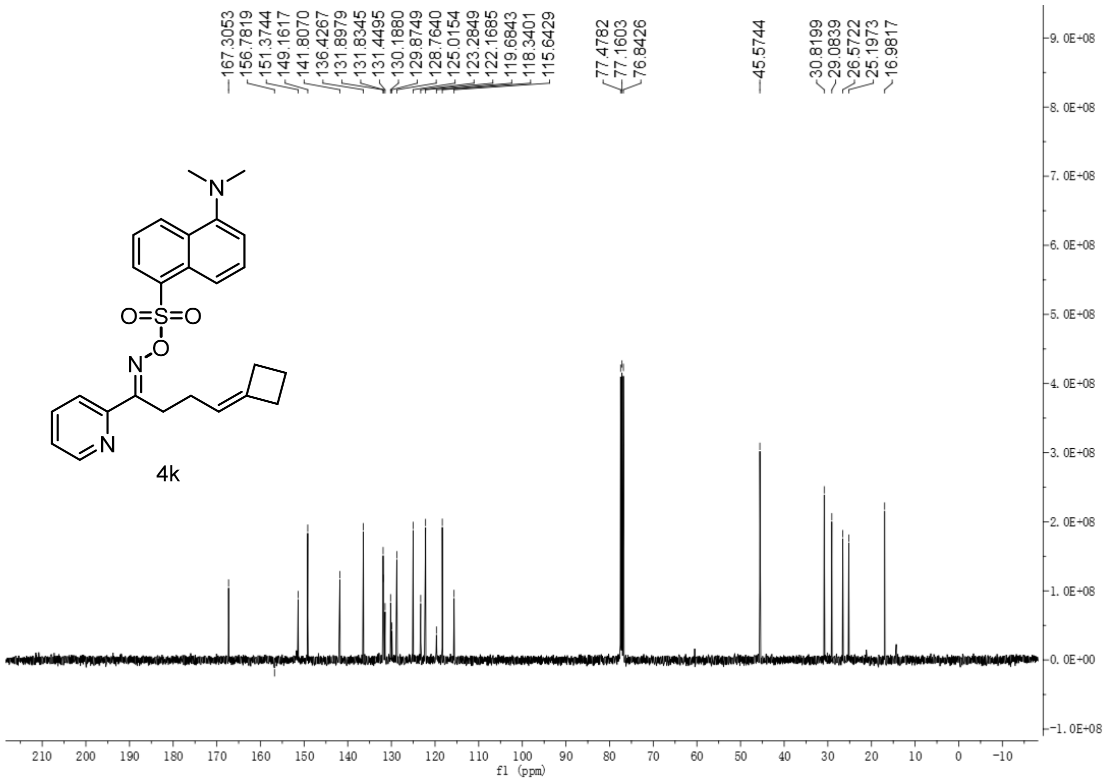
167.3053
156.7819
151.3744
149.1617
141.8070
136.4267
131.8979
131.8345
131.4495
130.1880
129.8749
128.7640
125.0154
123.2849
122.1685
119.6843
118.3401
115.6429
77.4782
77.1603
76.8426
45.5744
30.8199
29.0839
26.5722
25.1973
16.9817
4k
fl (ppm)

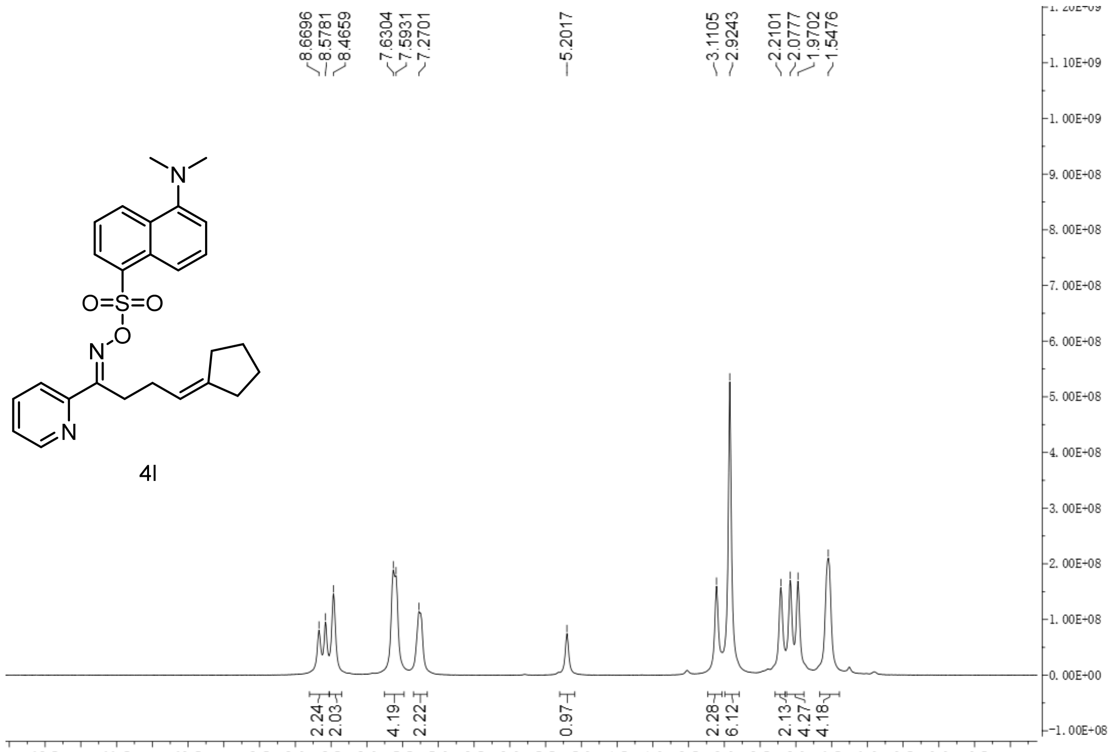
8.6696
8.5781
8.4659
7.6304
7.5931
7.2701
5.2017
3.1105
2.9243
2.2101
2.0777
1.9702
1.5476
4l
2.24
2.03
4.19
2.22
0.97
2.28
6.12
2.13
4.27
4.18
fl (ppm)

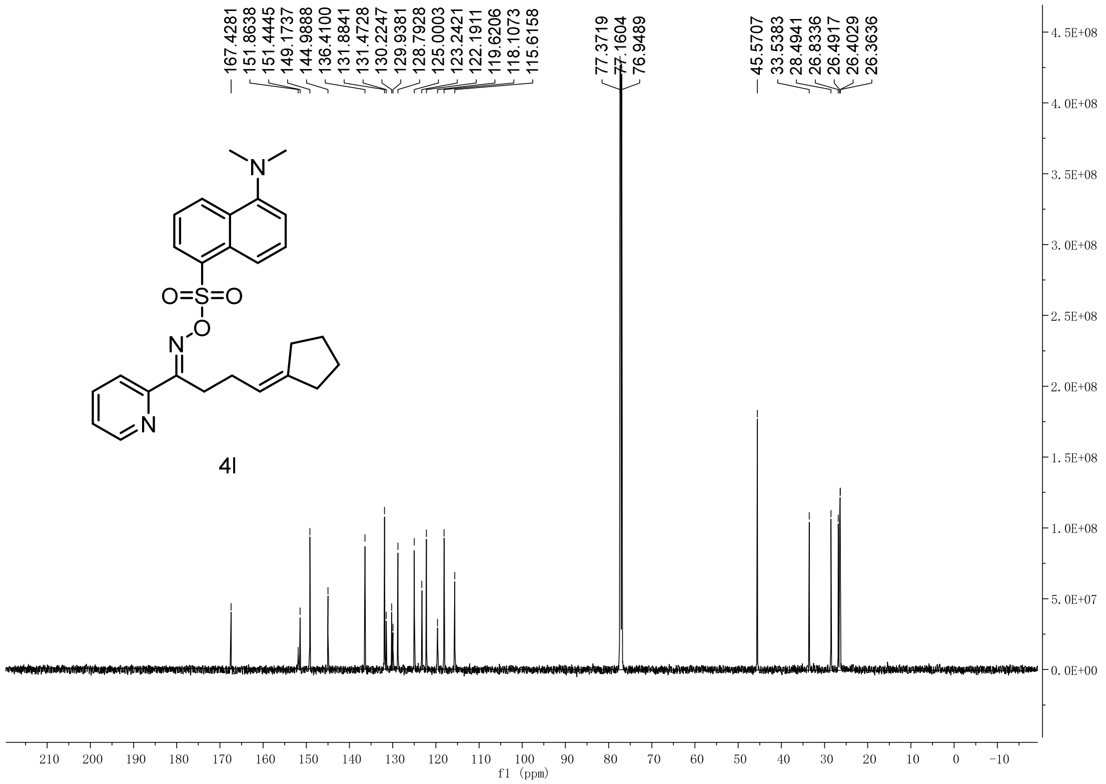

4l

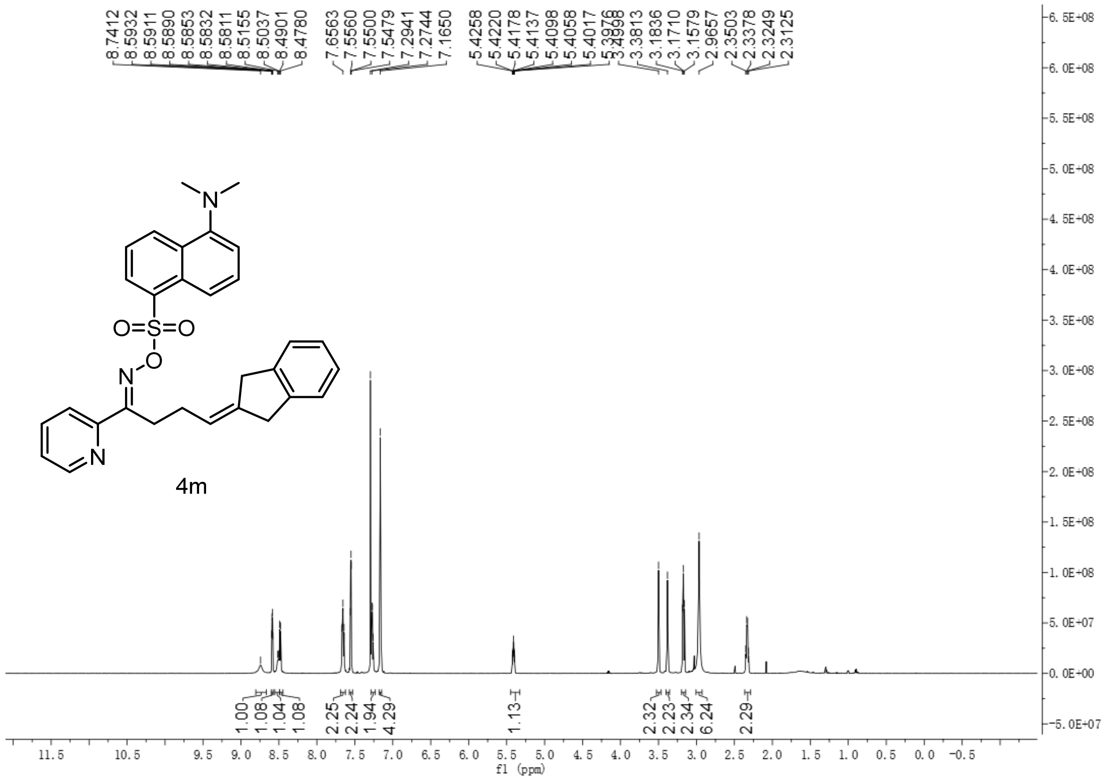

4m

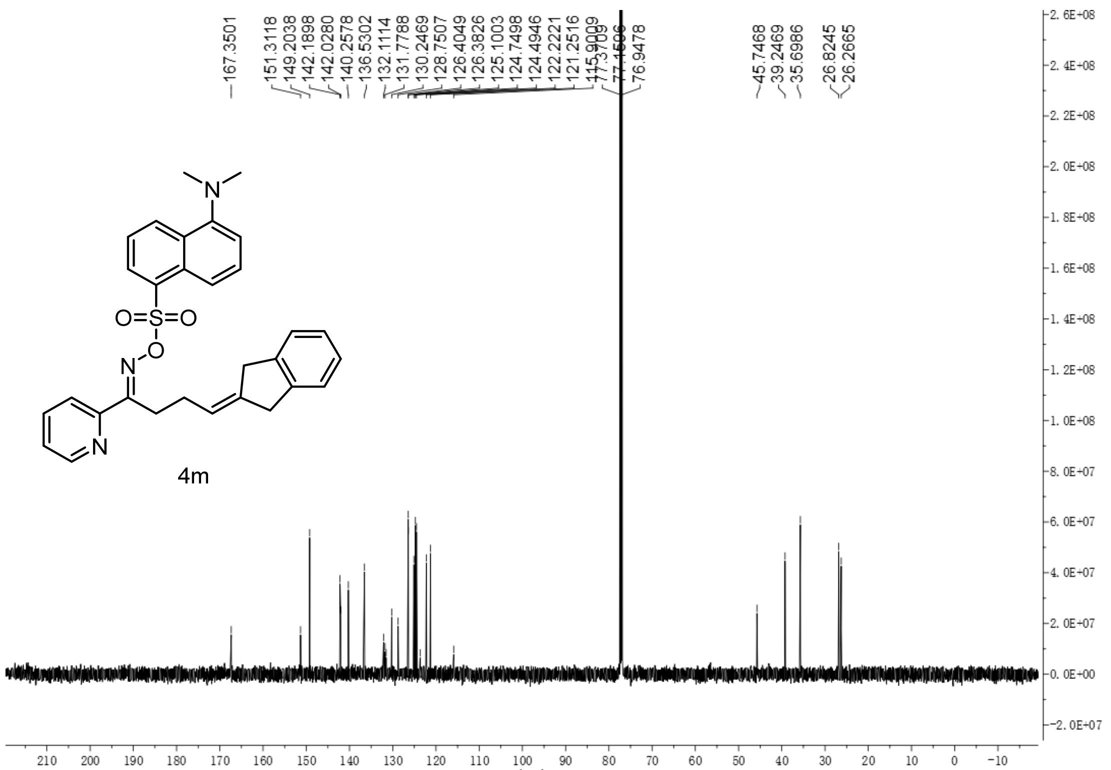
167.3501
151.3118
149.2038
142.1898
142.0280
140.2578
136.5302
132.1114
131.7788
130.2469
128.7507
126.4049
126.3826
125.1003
124.7498
124.4946
122.2221
121.2516
115.9009
77.3709
77.4506
76.9478
45.7468
39.2469
35.6986
26.8245
26.2665
4m
f1 (ppm)

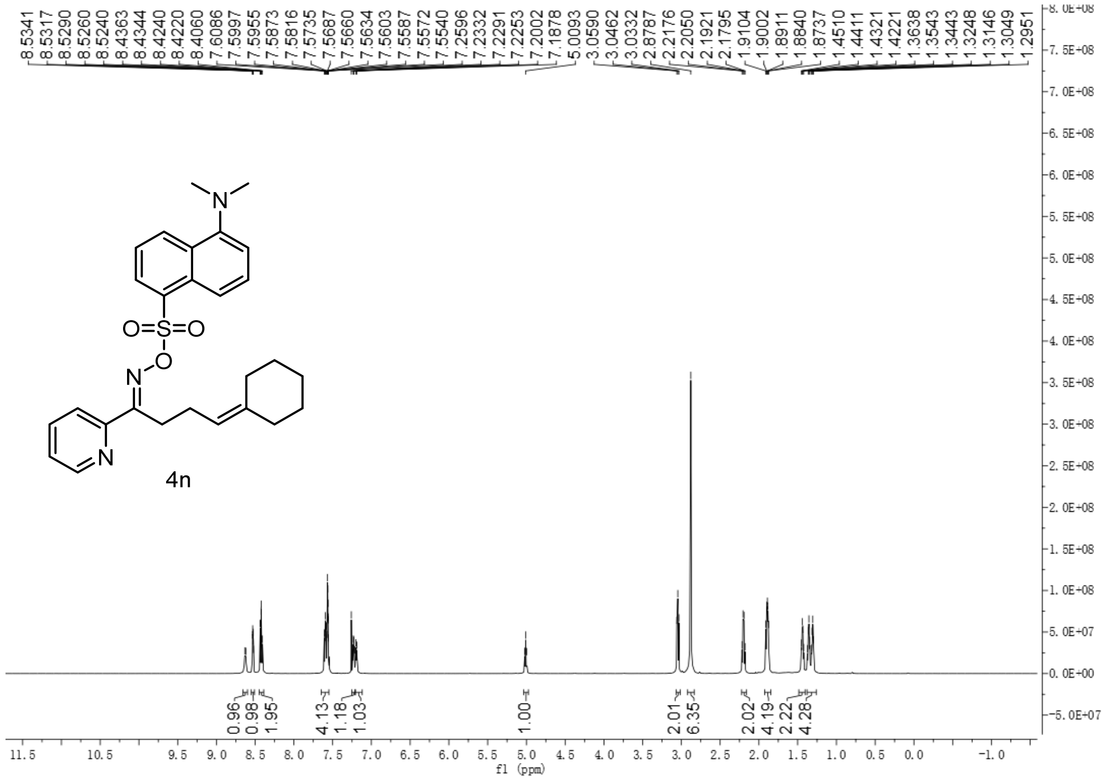
8.5341
8.5317
8.5290
8.5260
8.5240
8.4363
8.4344
8.4240
8.4220
8.4060
7.6086
7.5997
7.5955
7.5873
7.5816
7.5735
7.5687
7.5660
7.5634
7.5603
7.5587
7.5572
7.5540
7.2596
7.2332
7.2291
7.2253
7.2002
7.1878
5.0093
3.0590
3.0462
3.0332
2.8787
2.2176
2.2050
2.1921
2.1795
1.9104
1.9002
1.8911
1.8840
1.8737
1.4510
1.4411
1.4321
1.4221
1.3638
1.3543
1.3443
1.3248
1.3146
1.3049
1.2951
4n
0.96
0.98
1.95
4.13
1.18
1.03
1.00
2.01
6.35
2.02
4.19
2.22
4.28
f1 (ppm)

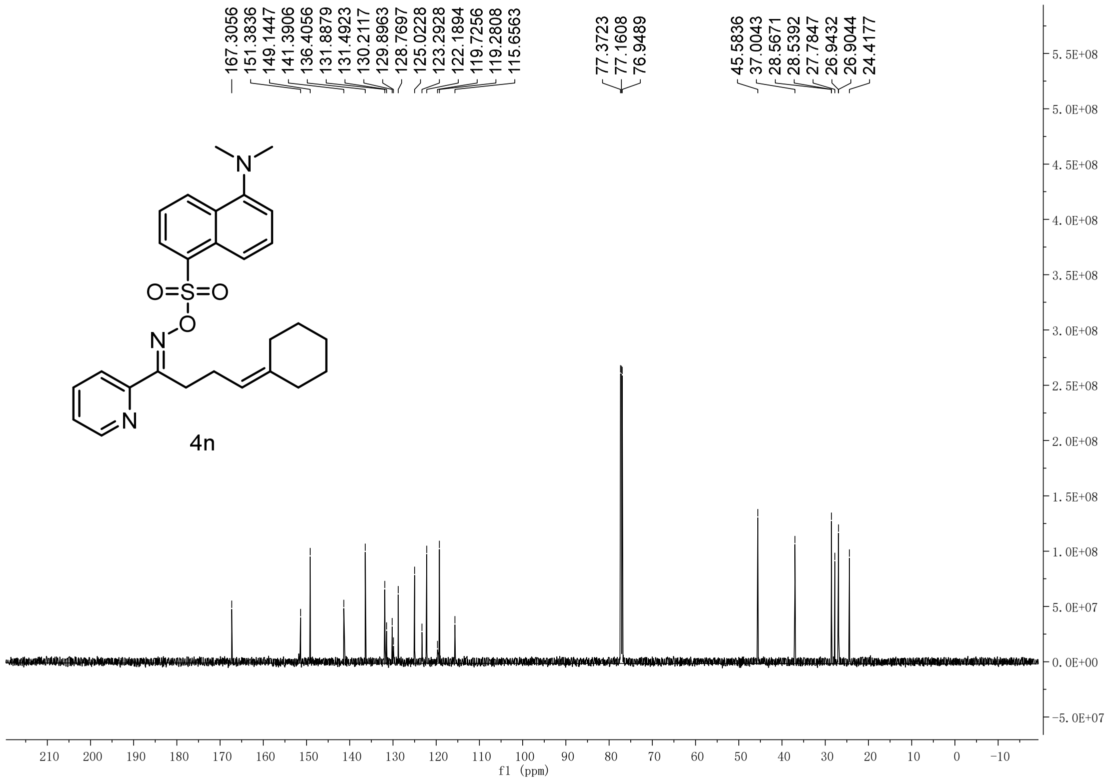
167.3056
151.3836
149.1447
141.3906
136.4056
131.8879
131.4923
130.2117
129.8963
128.7697
125.0228
123.2928
122.1894
119.7256
119.2808
115.6563
77.3723
77.1608
76.9489
45.5836
37.0043
28.5671
28.5392
27.7847
26.9432
26.9044
24.4177
4n
f1 (ppm)

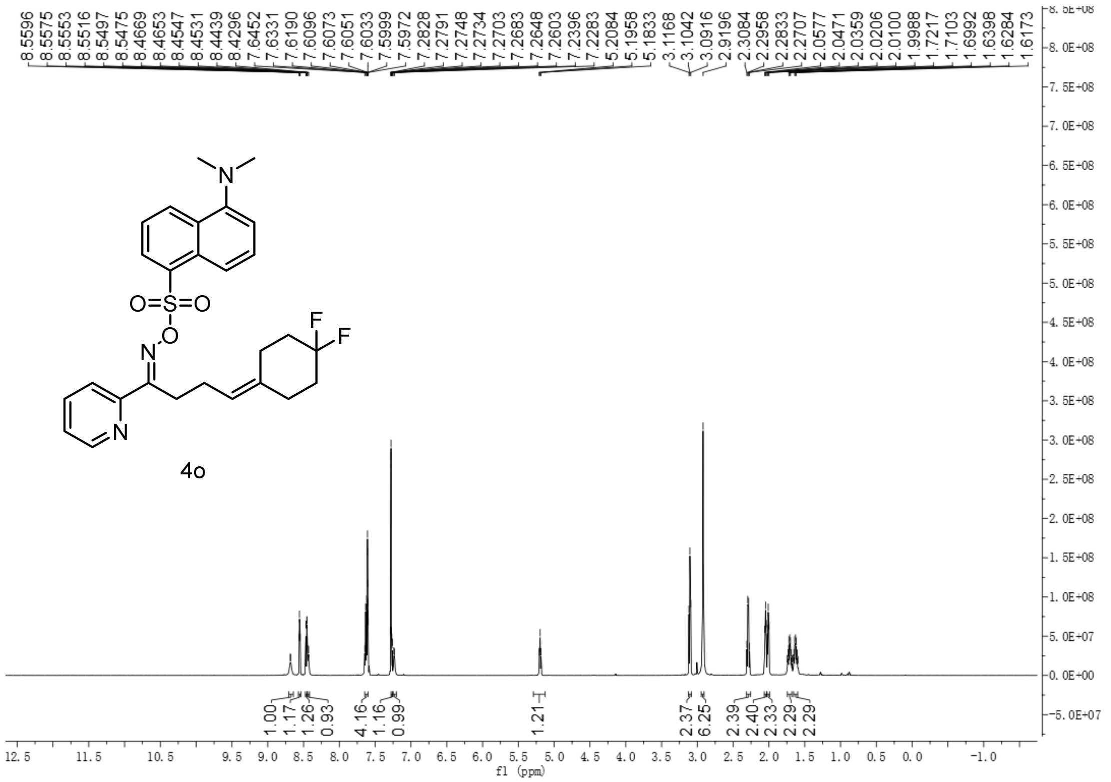
8.5596
8.5575
8.5553
8.5516
8.5497
8.5475
8.4669
8.4653
8.4547
8.4531
8.4439
8.4296
7.6452
7.6331
7.6190
7.6096
7.6073
7.6051
7.6033
7.5999
7.5972
7.2828
7.2791
7.2748
7.2734
7.2703
7.2683
7.2648
7.2603
7.2396
7.2283
5.2084
5.1958
5.1833
3.1168
3.1042
3.0916
2.9196
2.3084
2.2958
2.2833
2.2707
2.0577
2.0471
2.0359
2.0206
2.0100
1.9988
1.7217
1.7103
1.6992
1.6398
1.6284
1.6173
4o
1.00
1.17
1.26
0.93
4.16
1.16
0.99
1.21
2.37
6.25
2.39
2.40
2.33
2.29
2.29
f1 (ppm)

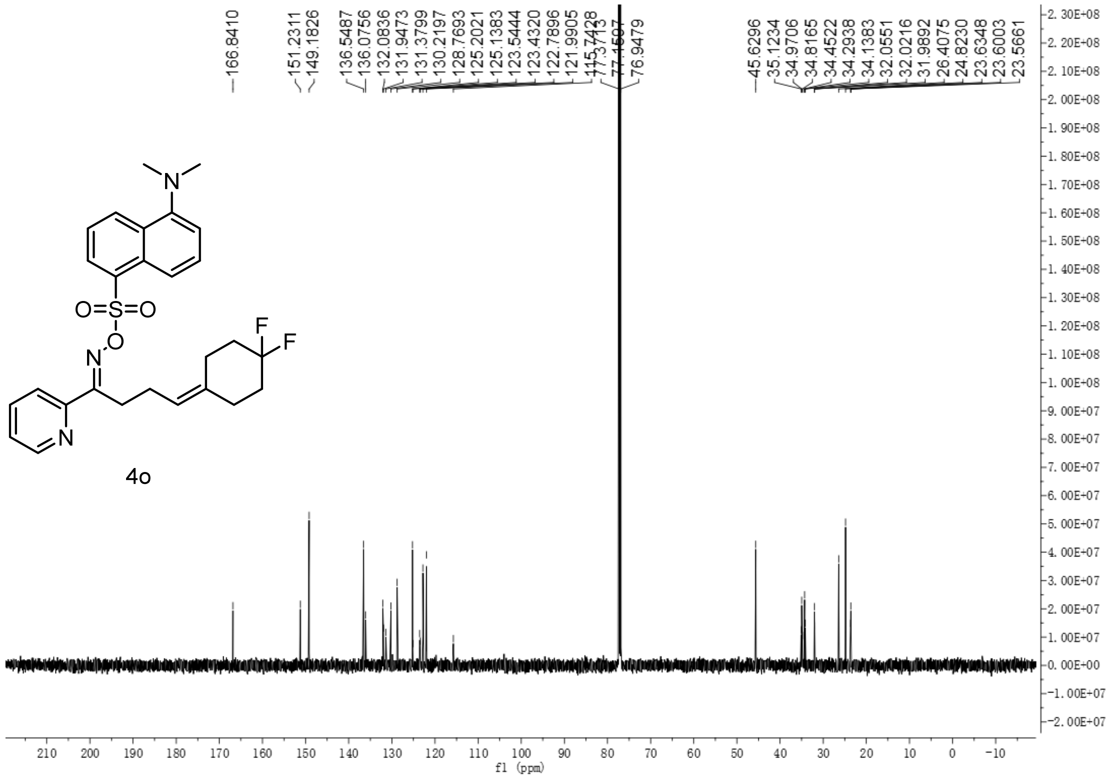
166.8410
151.2311
149.1826
136.5487
136.0756
132.0836
131.9473
131.3799
130.2197
128.7693
125.2021
125.1383
123.5444
123.4320
122.7896
121.9905
115.7428
77.3713
77.1597
76.9479
45.6296
35.1234
34.9706
34.8165
34.4522
34.2938
34.1383
32.0551
32.0216
31.9892
26.4075
24.8230
23.6348
23.6003
23.5661
4o
f1 (ppm)

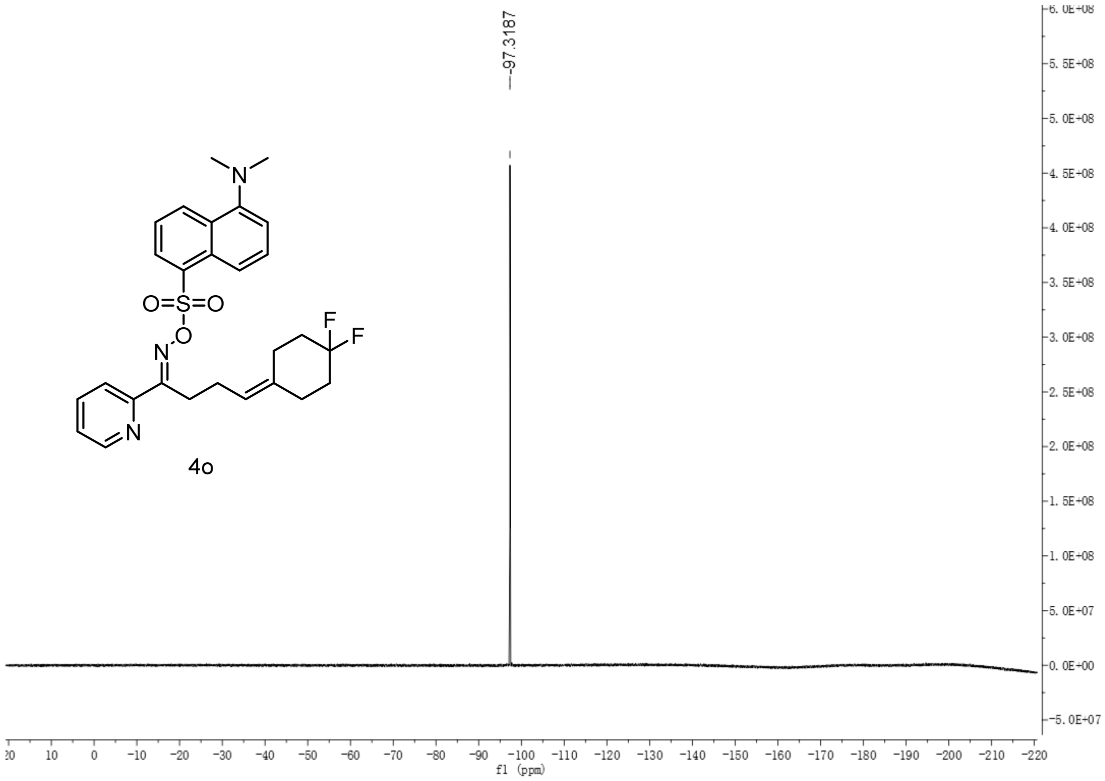
-97.3187
4o
f1 (ppm)

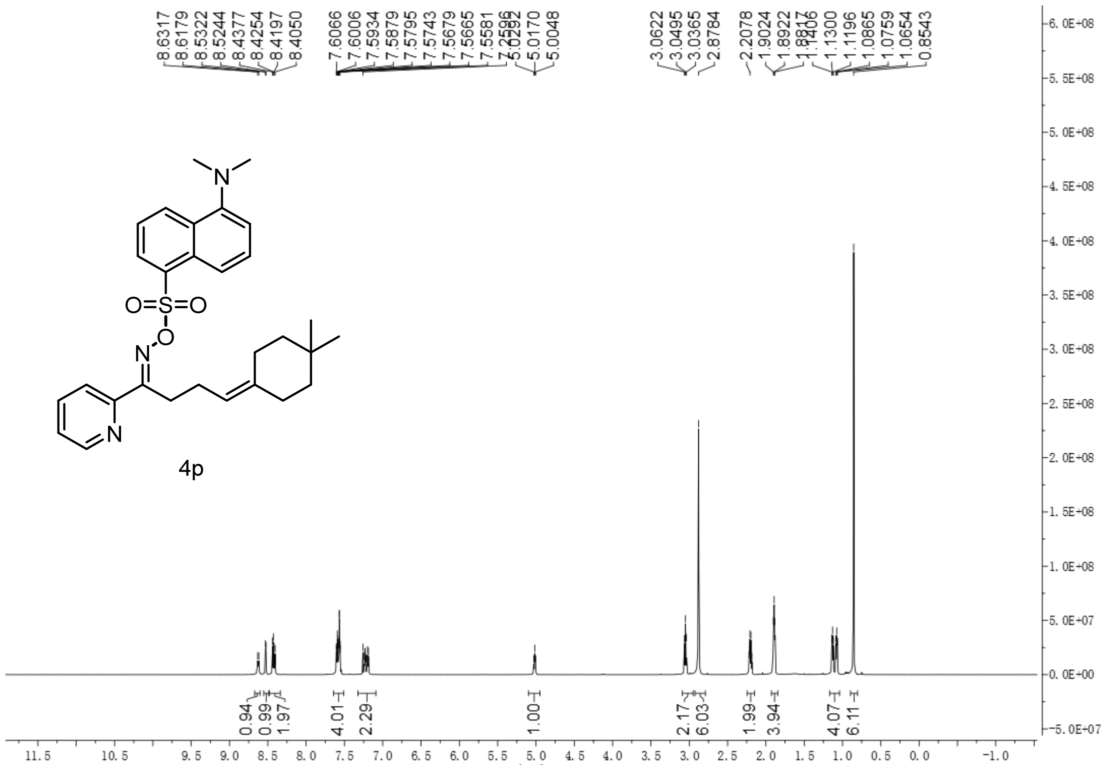
8.6317
8.6179
8.5322
8.5244
8.4377
8.4254
8.4197
8.4050
7.6066
7.6006
7.5934
7.5879
7.5795
7.5743
7.5679
7.5665
7.5581
5.0170
5.0048
3.0622
3.0495
3.0365
2.8784
2.2078
1.9024
1.8922
1.1300
1.1196
1.0865
1.0759
1.0654
0.8543
N
O=S=O
O
4p
0.94
0.99
1.97
4.01
2.29
1.00
2.17
6.03
1.99
3.94
4.07
6.11
f1 (ppm)

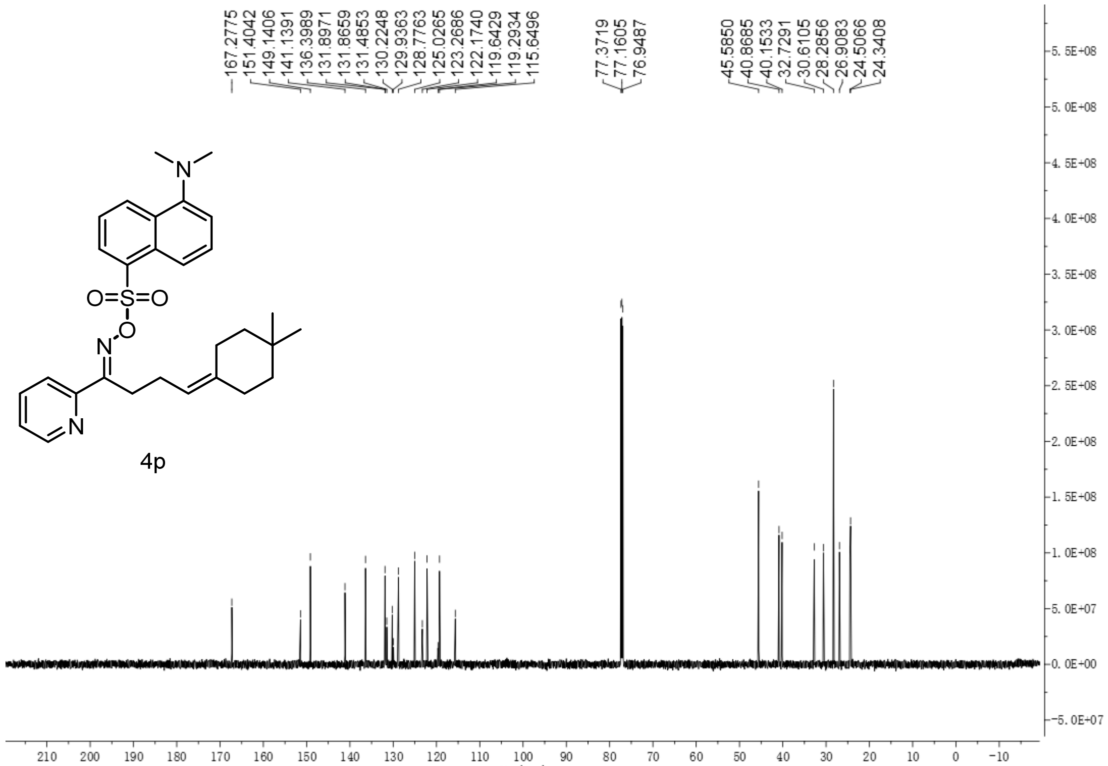
167.2775
151.4042
149.1406
141.1391
136.3989
131.8971
131.8659
131.4853
130.2248
129.9363
128.7763
125.0265
123.2686
122.1740
119.6429
119.2934
115.6496
77.3719
77.1605
76.9487
45.5850
40.8685
40.1533
32.7291
30.6105
28.2856
26.9083
24.5066
24.3408
N
O=S=O
O
4p
f1 (ppm)

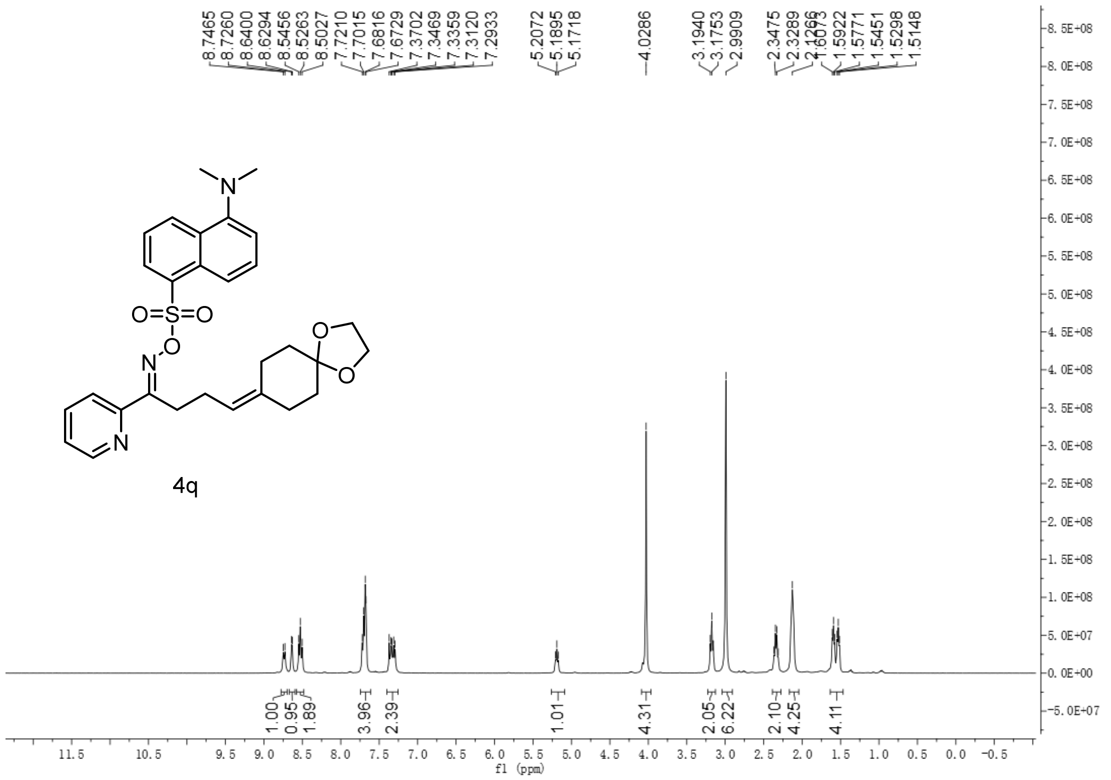

4q

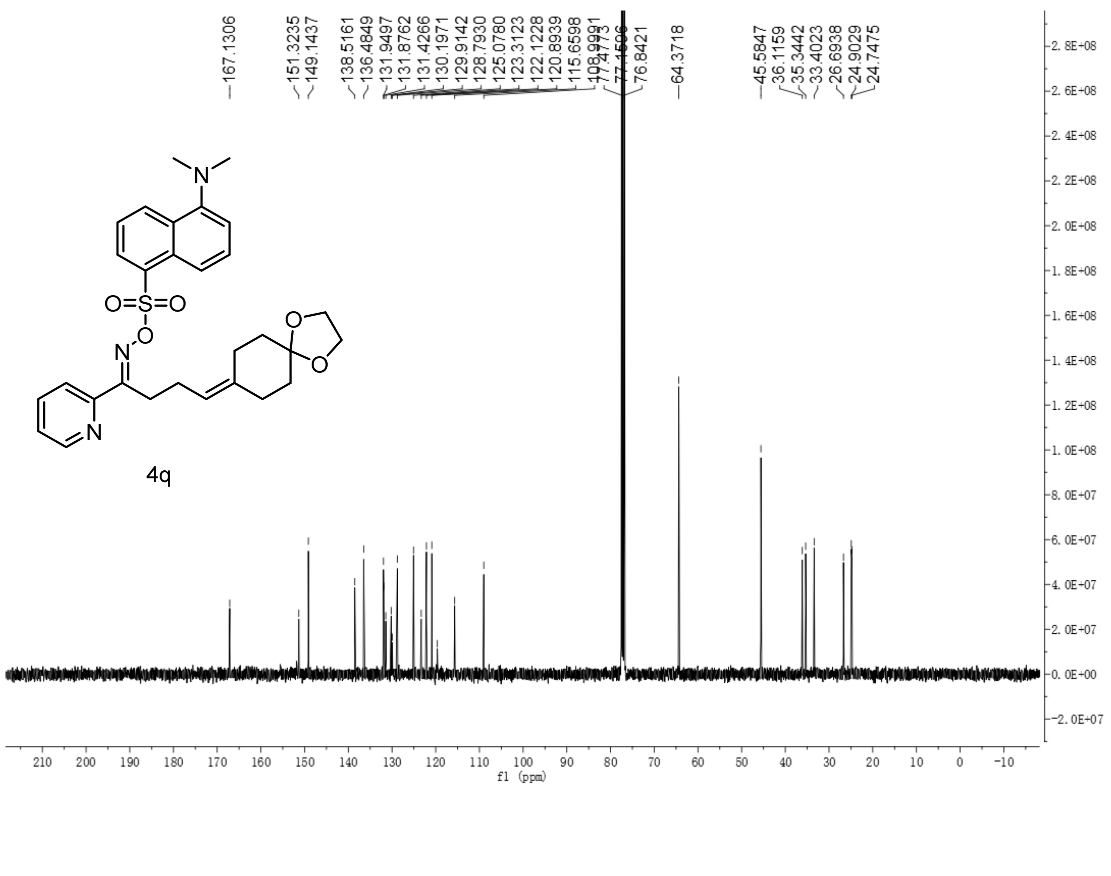

4q

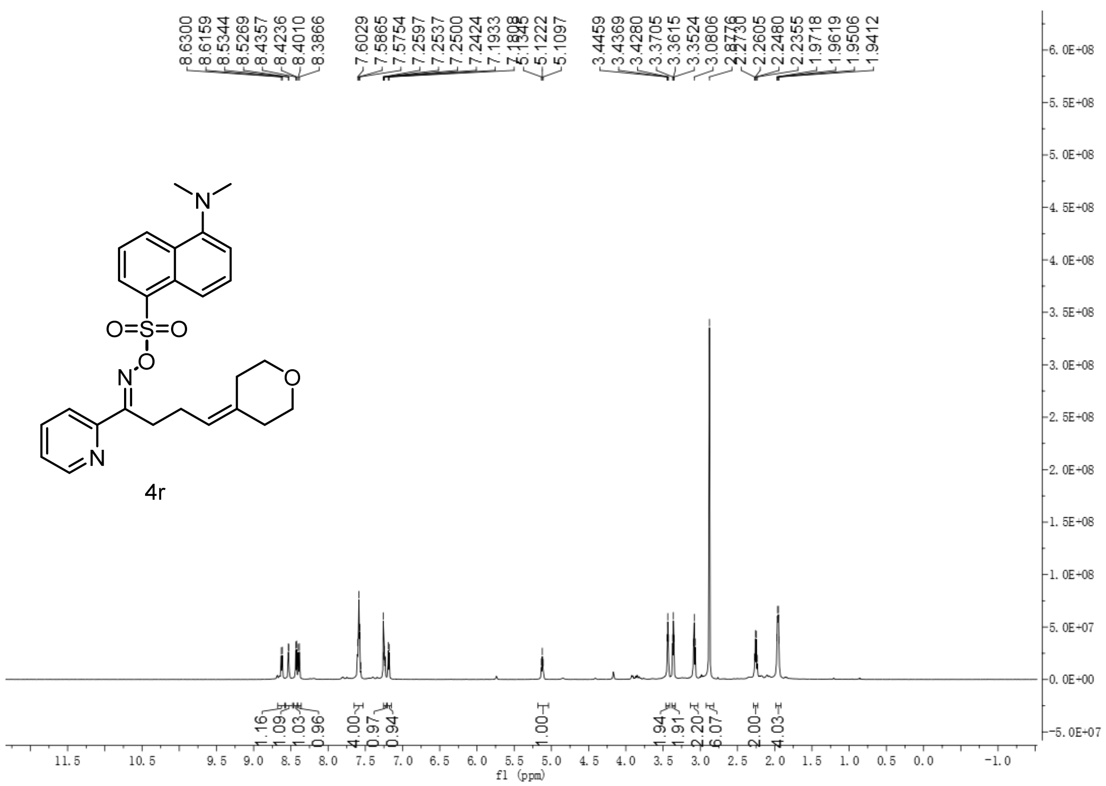
8.6300
8.6159
8.5344
8.5269
8.4357
8.4236
8.4010
8.3866
7.6029
7.5865
7.5754
7.2597
7.2537
7.2500
7.2424
7.1933
5.1222
5.1097
3.4459
3.4369
3.4280
3.3705
3.3615
3.3524
3.0806
2.2605
2.2480
2.2355
1.9718
1.9619
1.9506
1.9412
4r
1.16
1.09
1.03
0.96
4.00
0.97
0.94
1.00
1.94
1.91
2.20
6.07
2.00
4.03
f1 (ppm)

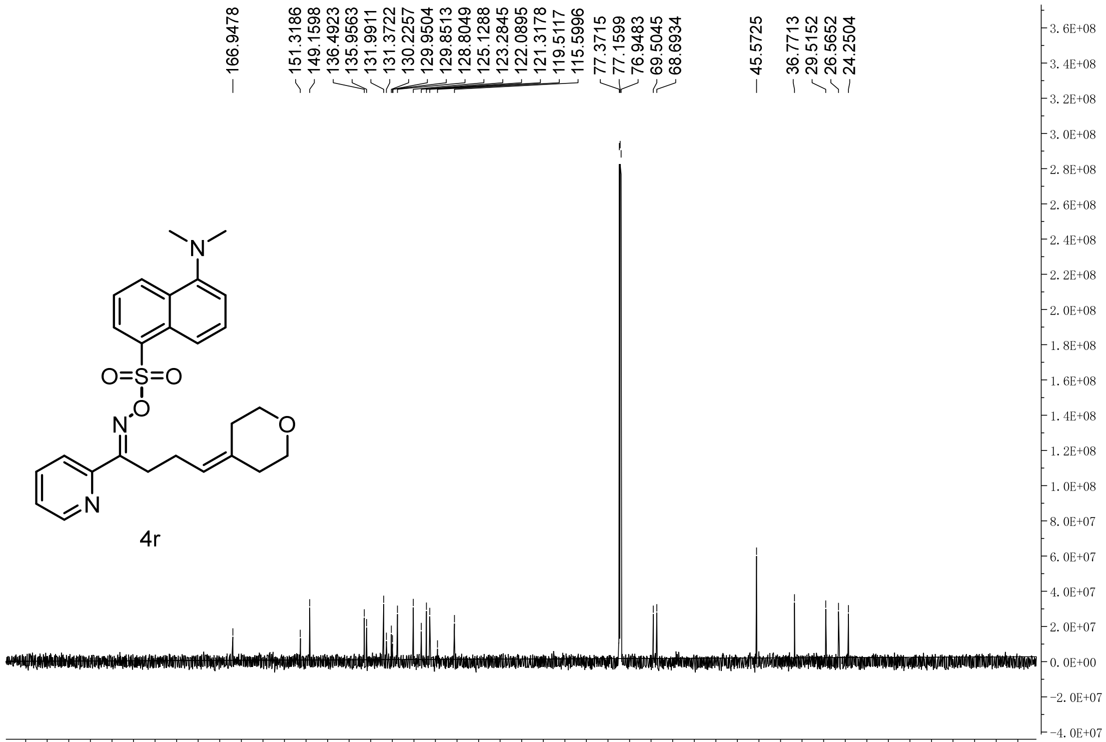
166.9478
151.3186
149.1598
136.4923
135.9563
131.9911
131.3722
130.2257
129.9504
129.8513
128.8049
125.1288
123.2845
122.0895
121.3178
119.5117
115.5996
77.3715
77.1599
76.9483
69.5045
68.6934
45.5725
36.7713
29.5152
26.5652
24.2504
4r
f1 (ppm)

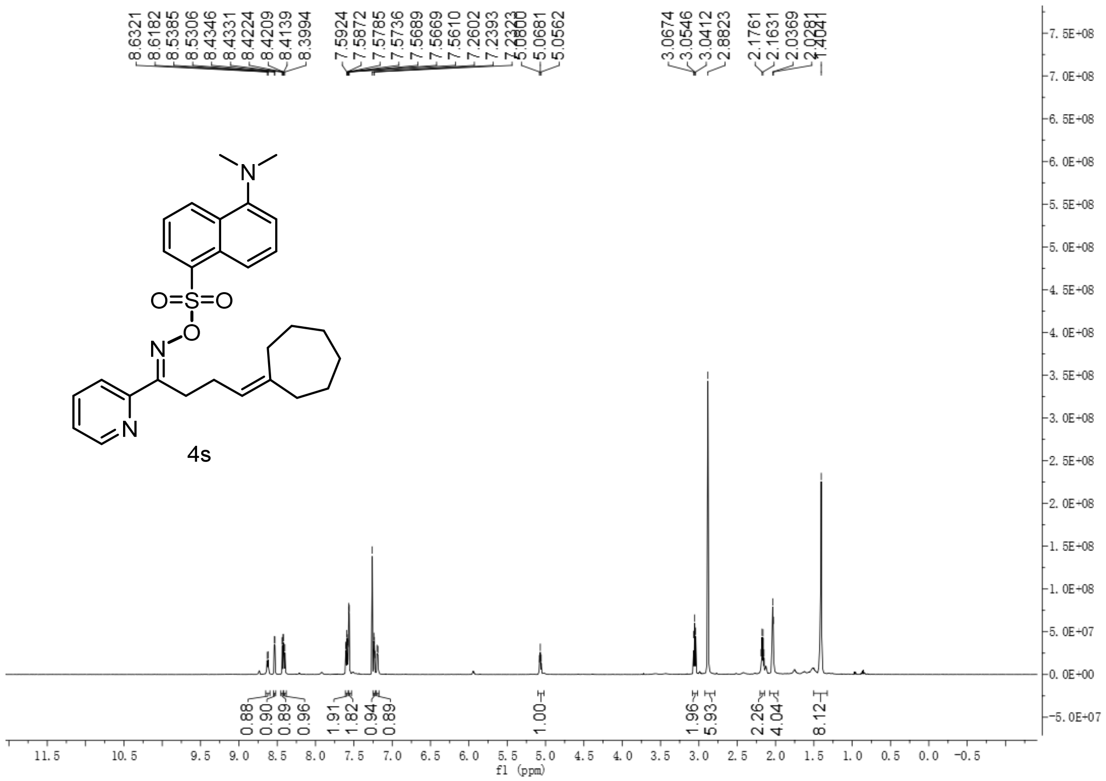

4s

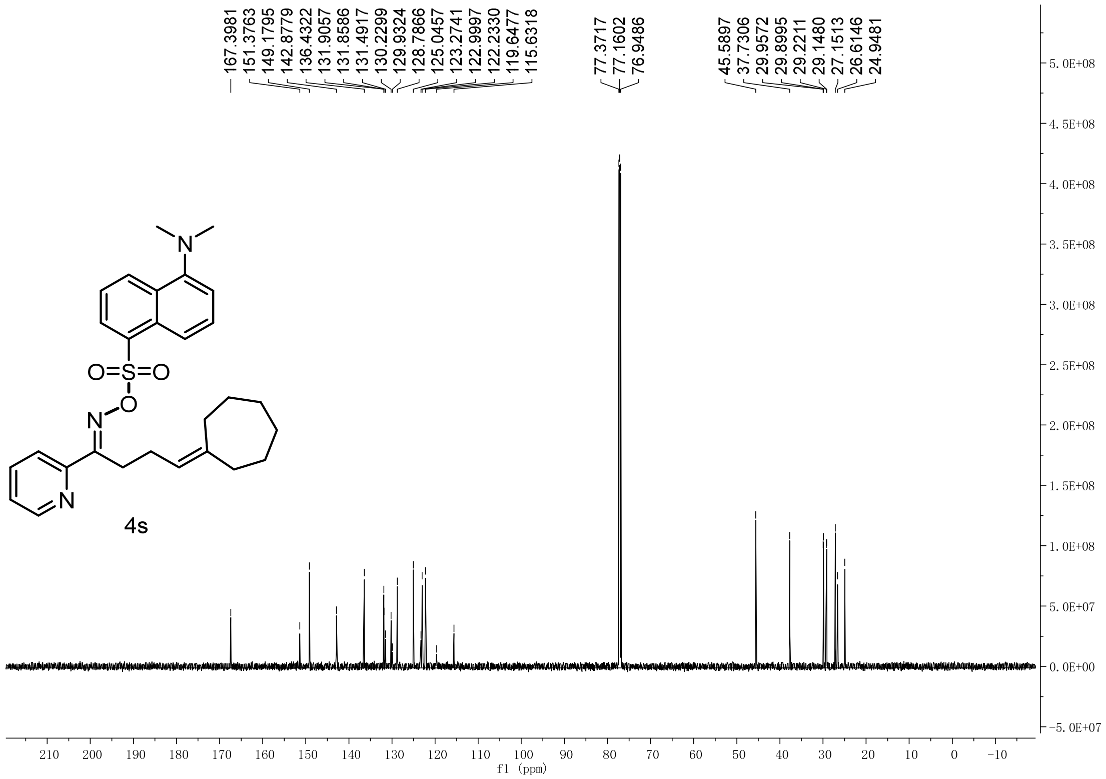

4s

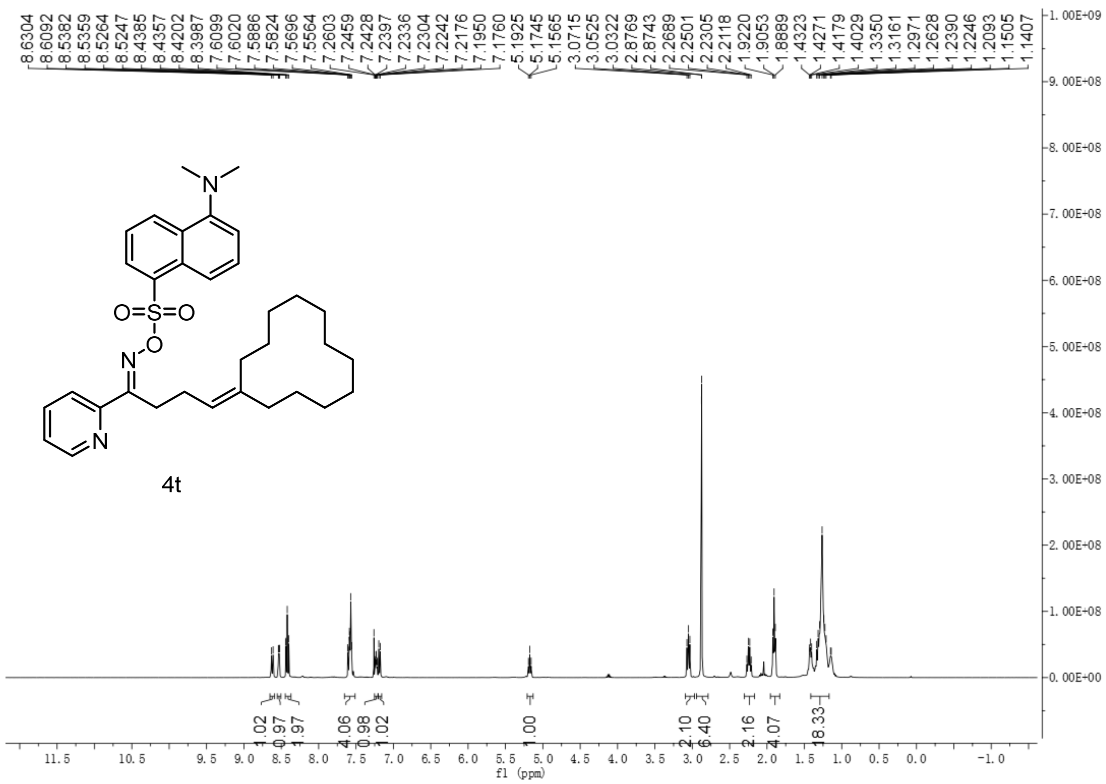
8.6304 8.6092 8.5382 8.5359 8.5264 8.5247 8.4385 8.4357 8.4202 8.3987 7.6099 7.6020 7.5886 7.5824 7.5696 7.5564 7.2603 7.2459 7.2428 7.2397 7.2336 7.2304 7.2242 7.2176 7.1950 7.1760 5.1925 5.1745 5.1565 3.0715 3.0525 3.0322 2.8769 2.8743 2.2689 2.2501 2.2305 2.2118 1.9220 1.9053 1.8889 1.4323 1.4271 1.4179 1.4029 1.3350 1.3161 1.2971 1.2628 1.2390 1.2246 1.2093 1.1505 1.1407
4t
1.02 0.97 1.97 4.06 0.98 1.02 1.00 2.10 6.40 2.16 4.07 18.33
f1 (ppm)

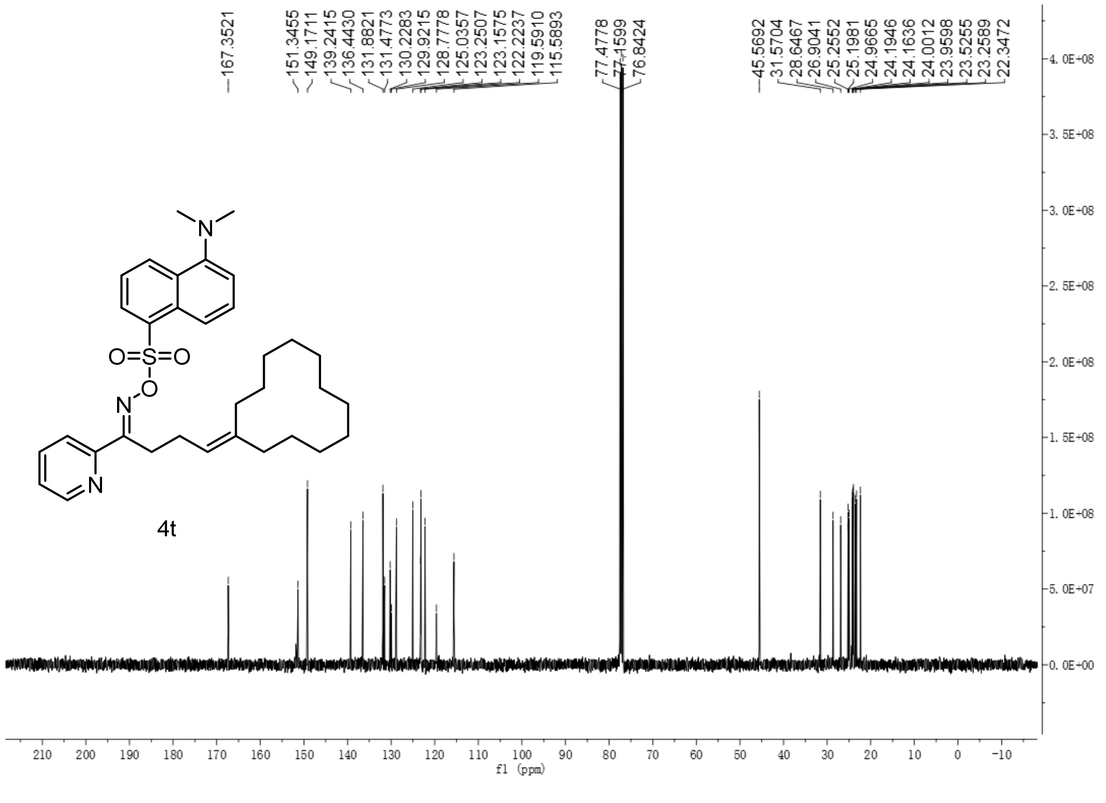
167.3521 151.3455 149.1711 139.2415 136.4430 131.8821 131.4773 130.2283 129.9215 128.7778 125.0357 123.2507 123.1575 122.2237 119.5910 115.5893 77.4778 77.1599 76.8424 45.5692 31.5704 28.6467 26.9041 25.2552 25.1981 24.9665 24.1946 24.1636 24.0012 23.9598 23.5255 23.2589 22.3472
4t
f1 (ppm)

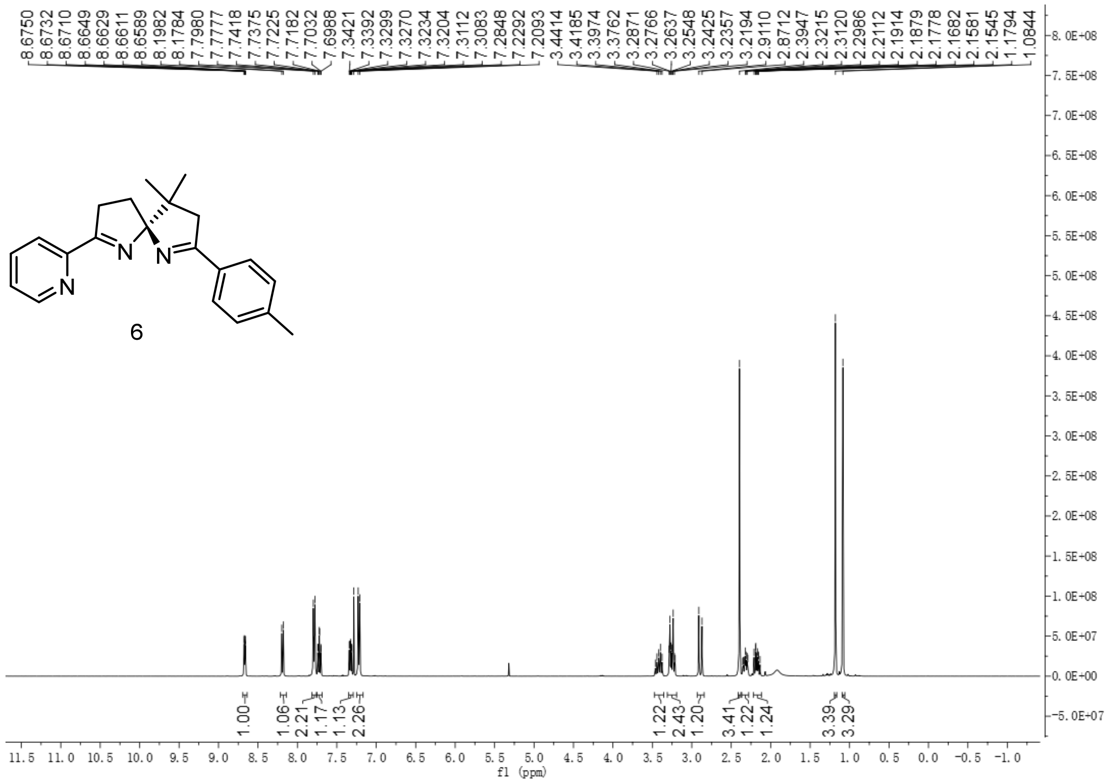
8.6750
8.6732
8.6710
8.6649
8.6629
8.6611
8.6589
8.1982
8.1784
7.7980
7.7777
7.7418
7.7375
7.7225
7.7182
7.7032
7.6988
7.3421
7.3392
7.3299
7.3270
7.3234
7.3204
7.3112
7.3083
7.2848
7.2292
7.2093
3.4414
3.4185
3.3974
3.3762
3.2871
3.2766
3.2637
3.2548
3.2425
3.2357
3.2194
2.9110
2.8712
2.3947
2.3215
2.3120
2.2986
2.2112
2.1914
2.1879
2.1778
2.1682
2.1581
2.1545
1.1794
1.0844
N
N
N
6
1.00
1.06
2.21
1.17
1.13
2.26
1.22
2.43
1.20
3.41
1.22
1.24
3.39
3.29
fl (ppm)

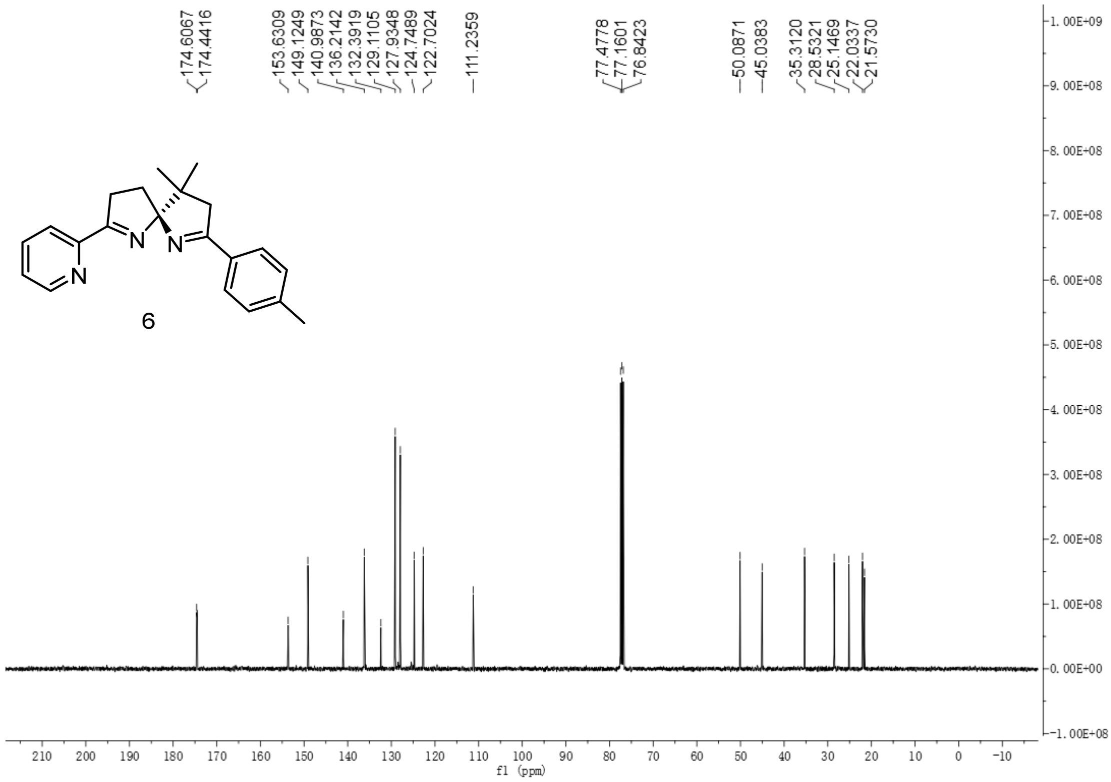
174.6067
174.4416
153.6309
149.1249
140.9873
136.2142
132.3919
129.1105
127.9348
124.7489
122.7024
111.2359
77.4778
77.1601
76.8423
50.0871
45.0383
35.3120
28.5321
25.1469
22.0337
21.5730
N
N
N
6
fl (ppm)

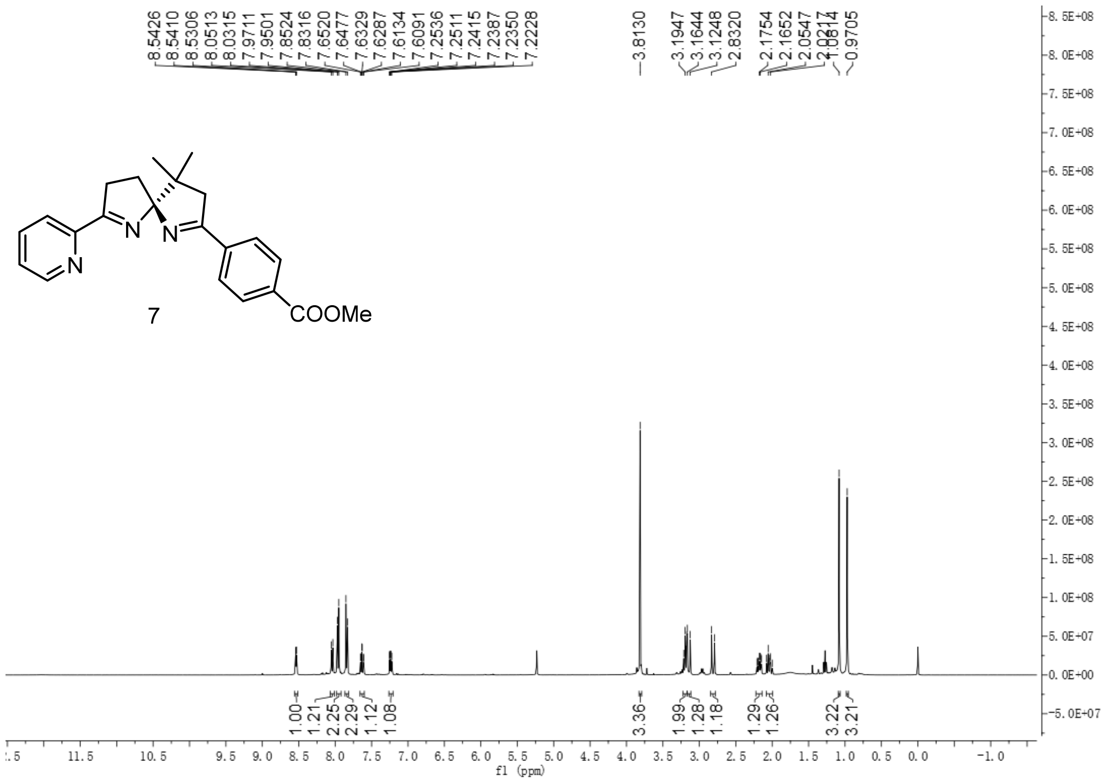

8.5426
8.5410
8.5306
8.0513
8.0315
7.9711
7.9501
7.8524
7.8316
7.6520
7.6477
7.6329
7.6287
7.6134
7.6091
7.2536
7.2511
7.2415
7.2387
7.2350
7.2228
3.8130
3.1947
3.1644
3.1248
2.8320
2.1754
2.1652
2.0547
0.9705
COOMe
7
1.00
1.21
2.25
2.29
1.12
1.08
3.36
1.99
1.28
1.18
1.29
1.26
3.22
3.21
fl (ppm)

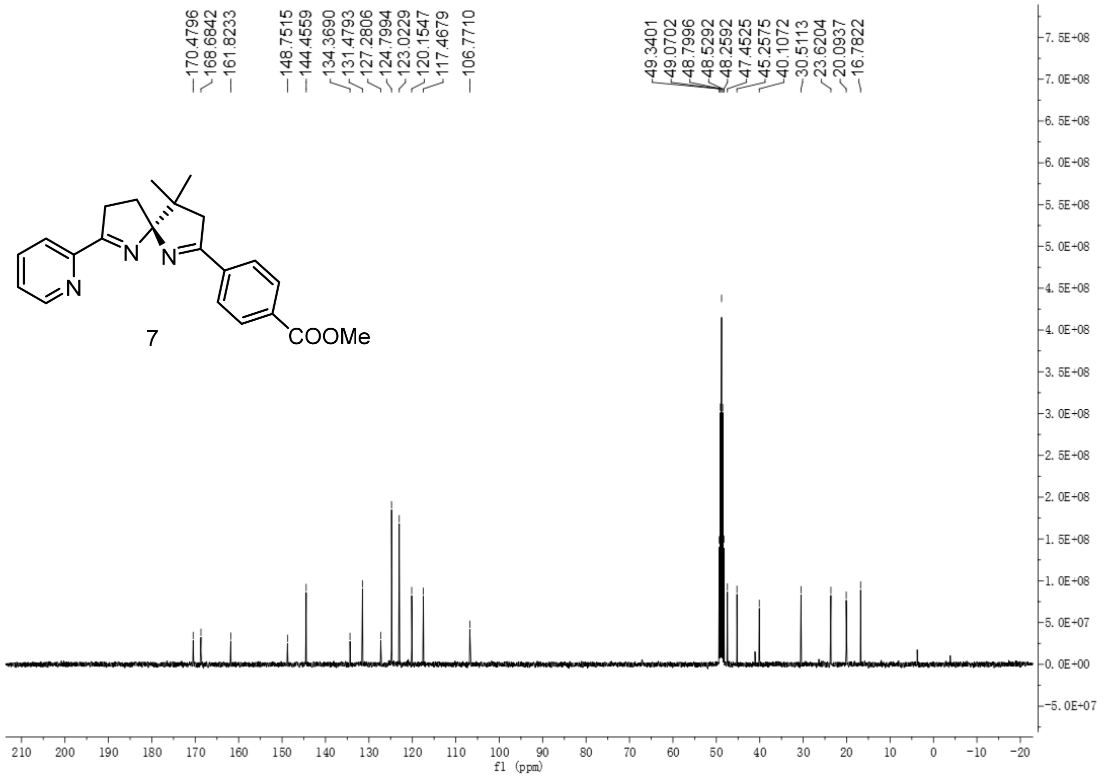

170.4796
168.6842
161.8233
148.7515
144.4559
134.3690
131.4793
127.2806
124.7994
123.0229
120.1547
117.4679
106.7710
49.3401
49.0702
48.7996
48.5292
48.2592
47.4525
45.2575
40.1072
30.5113
23.6204
20.0937
16.7822
COOMe
7
fl (ppm)

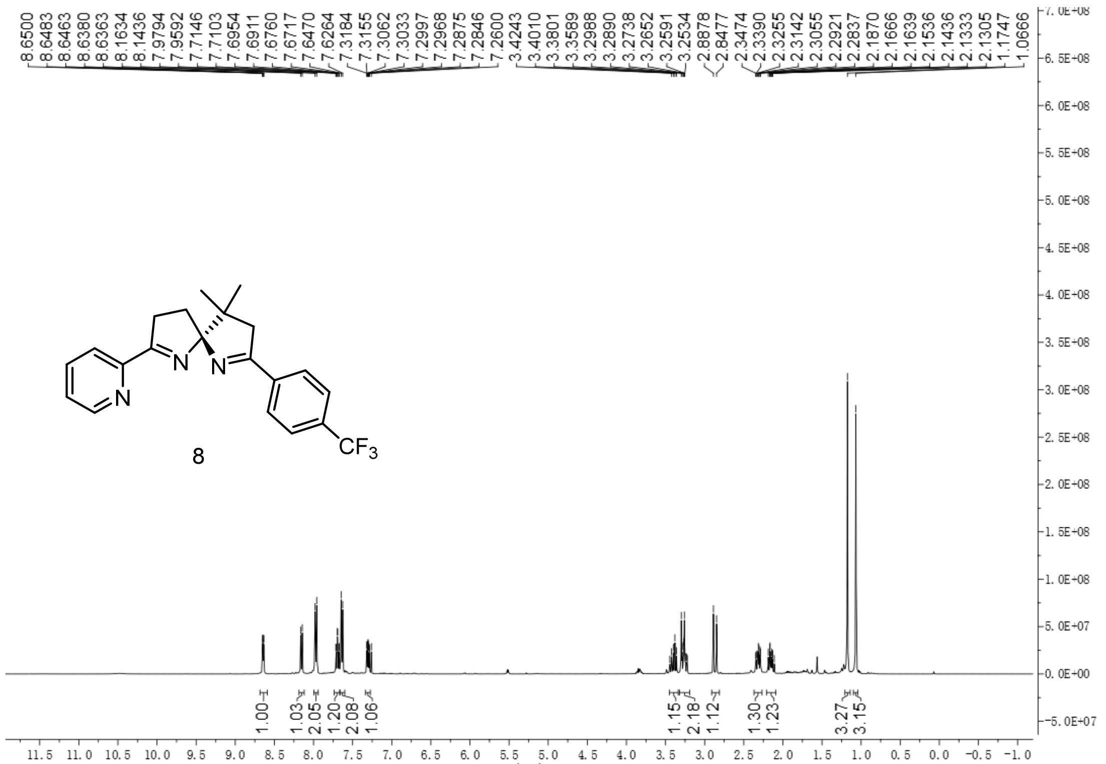
8.6500
8.6483
8.6463
8.6380
8.6363
8.1634
8.1436
7.9794
7.9592
7.7146
7.7103
7.6954
7.6911
7.6760
7.6717
7.6470
7.6264
7.3184
7.3155
7.3062
7.3033
7.2997
7.2968
7.2875
7.2846
7.2600
3.4243
3.4010
3.3801
3.3589
3.2988
3.2890
3.2738
3.2652
3.2591
3.2534
2.8878
2.8477
2.3474
2.3390
2.3255
2.3142
2.3055
2.2921
2.2837
2.1870
2.1666
2.1639
2.1536
2.1436
2.1333
2.1305
1.1747
1.0666
N
N
N
CF3
8
1.00
1.03
2.05
1.20
2.08
1.06
1.15
2.18
1.12
1.30
1.23
3.27
3.15
f1 (ppm)

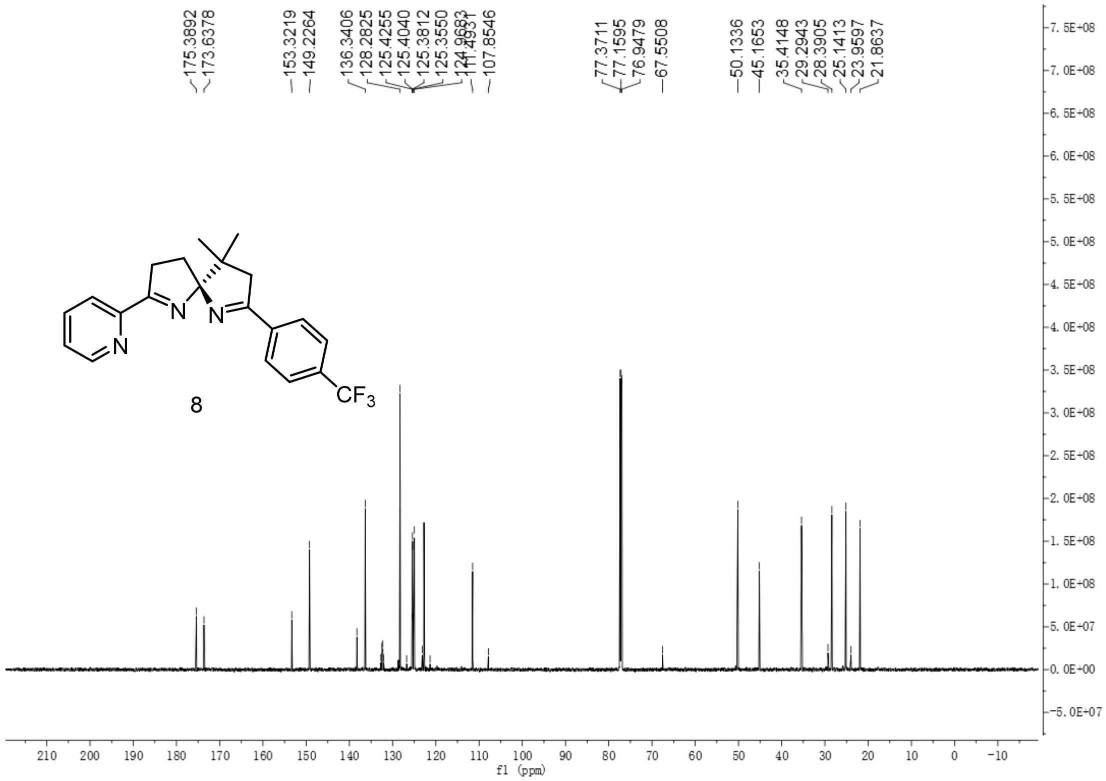
175.3892
173.6378
153.3219
149.2264
136.3406
128.2825
125.4255
125.4040
125.3812
125.3550
124.9683
111.4931
107.8546
77.3711
77.1595
76.9479
67.5508
50.1336
45.1653
35.4148
29.2943
28.3905
25.1413
23.9597
21.8637
N
N
N
CF3
8
f1 (ppm)

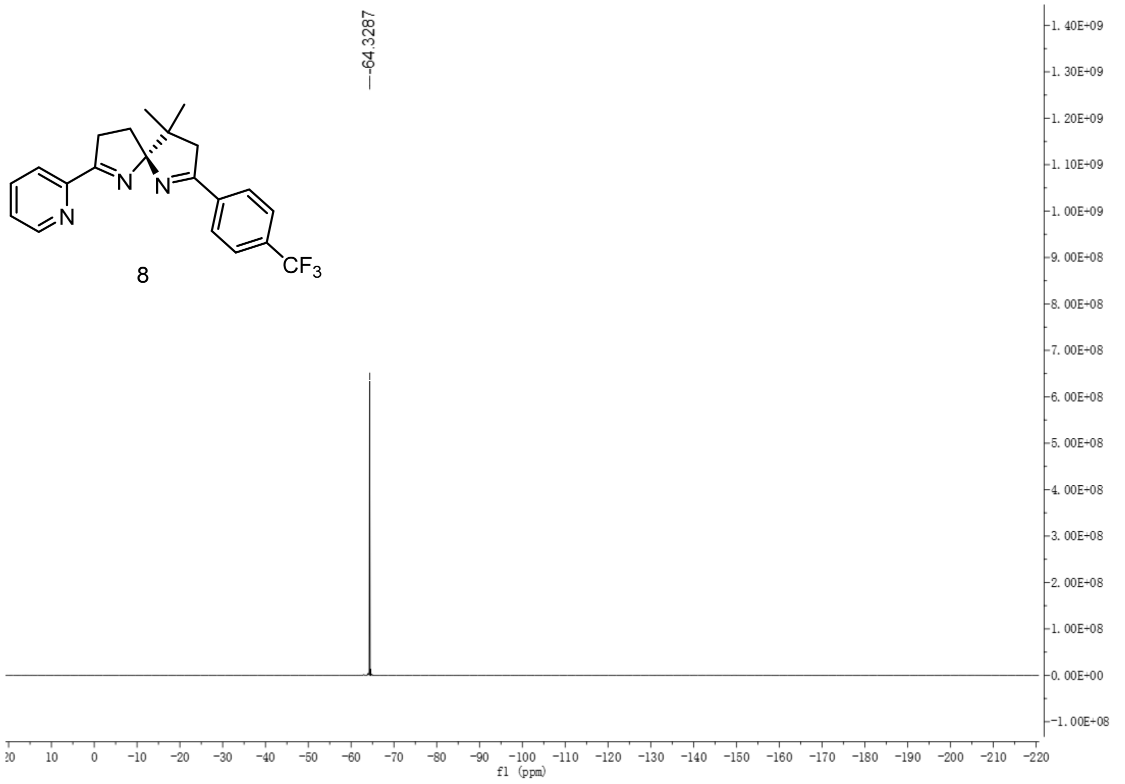

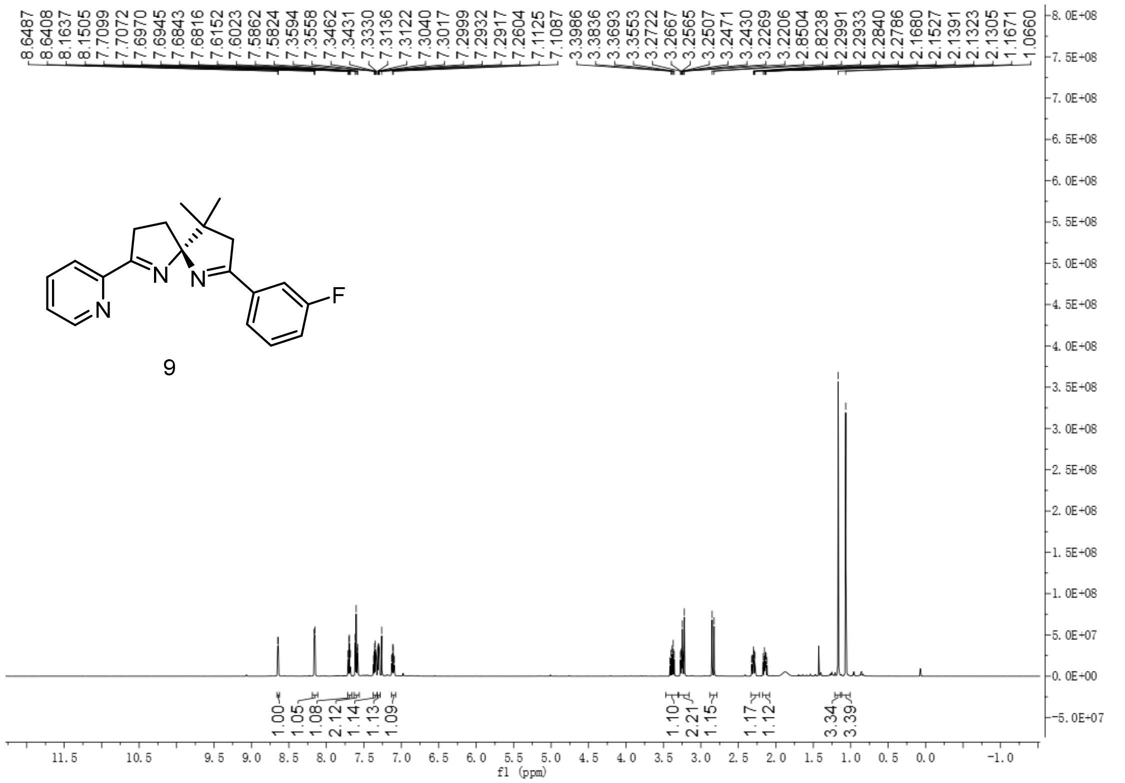

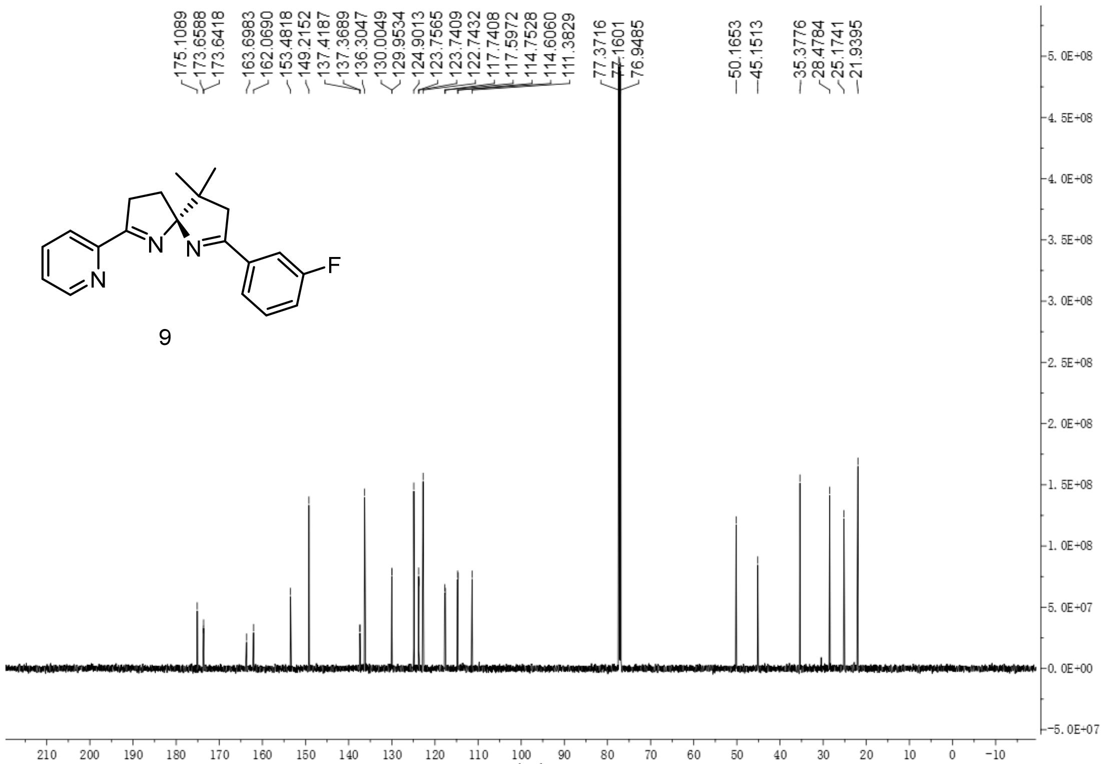
175.1089
173.6588
173.6418
163.6983
162.0690
153.4818
149.2152
137.4187
137.3689
136.3047
130.0049
129.9534
124.9013
123.7565
123.7409
122.7432
117.7408
117.5972
114.7528
114.6060
111.3829
77.3716
77.1601
76.9485
50.1653
45.1513
35.3776
28.4784
25.1741
21.9395
F
N
N
N
fl (ppm)

9

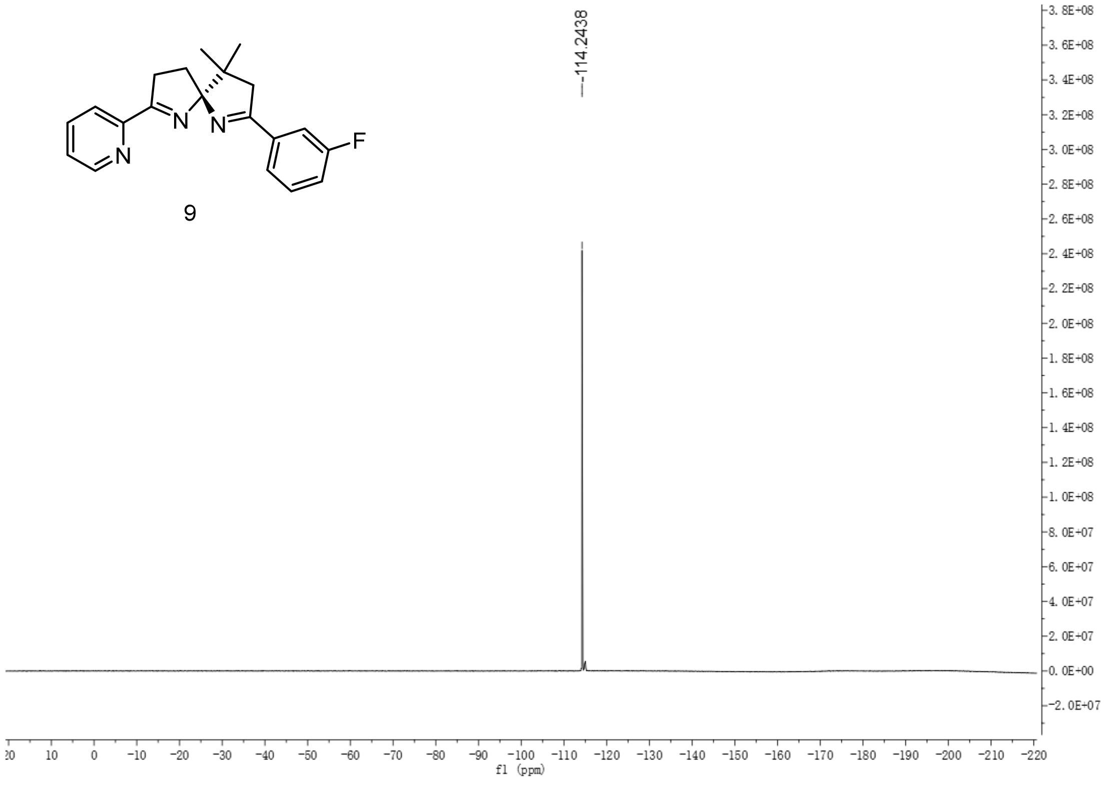
-114.2438
F
N
N
N
fl (ppm)

9

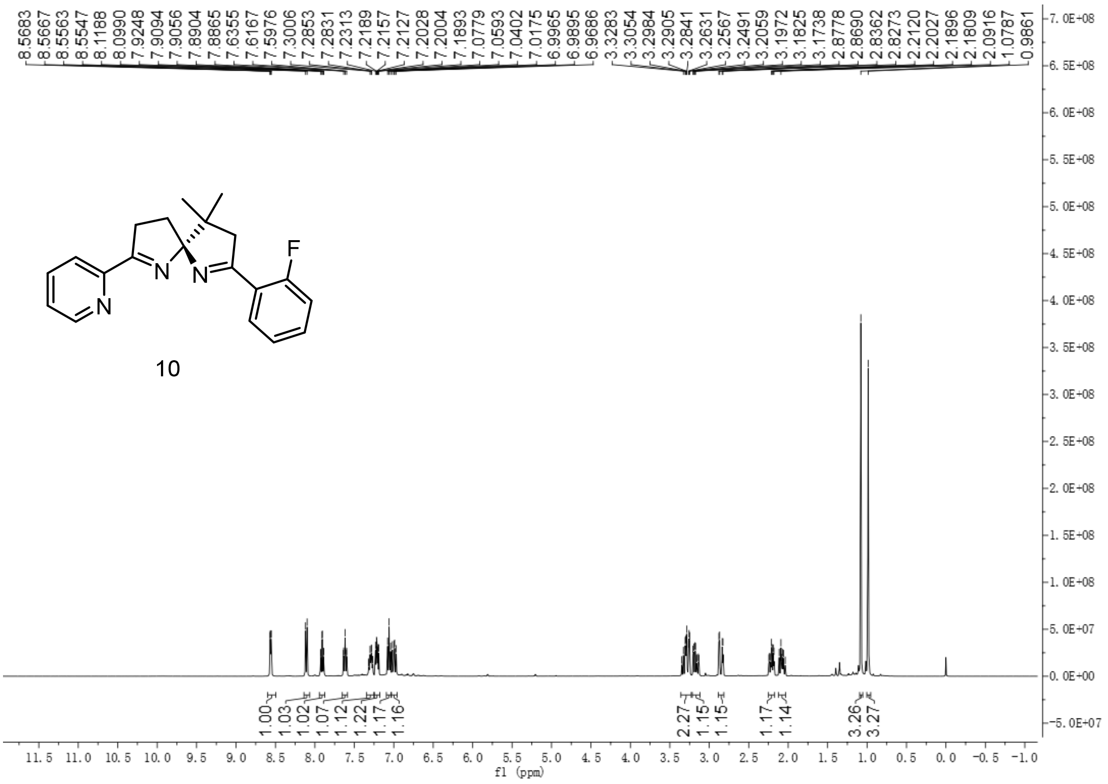

8.5683 8.5667 8.5563 8.5547 8.1188 8.0990 7.9248 7.9094 7.9056 7.8904 7.8865 7.6355 7.6167 7.5976 7.3006 7.2853 7.2831 7.2313 7.2189 7.2157 7.2127 7.2028 7.2004 7.1893 7.0779 7.0593 7.0402 7.0175 6.9965 6.9895 6.9686
3.3283 3.3054 3.2984 3.2905 3.2841 3.2631 3.2567 3.2491 3.2059 3.1972 3.1825 3.1738 2.8778 2.8690 2.8362 2.8273 2.2120 2.2027 2.1896 2.1809 2.0916 1.0787 0.9861
F
N
N
N
10
1.00 1.03 1.02 1.07 1.12 1.22 1.17 1.16
2.27 1.15 1.15
1.17 1.14
3.26 3.27
fl (ppm)

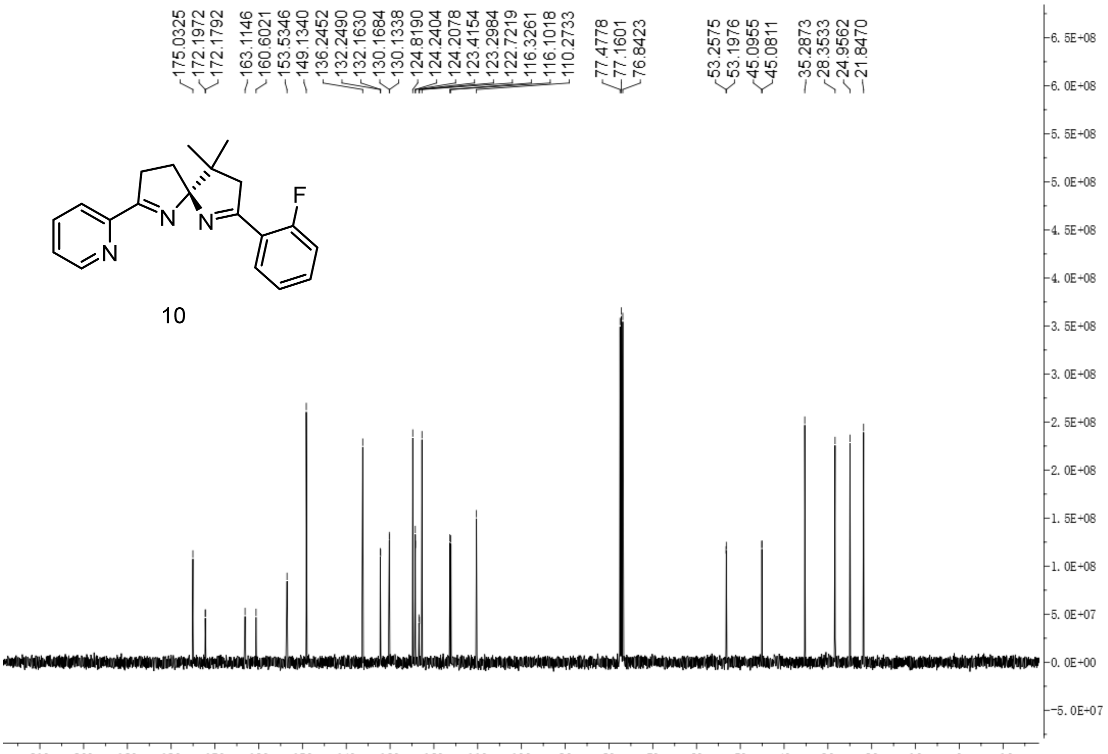

175.0325 172.1972 172.1792 163.1146 160.6021 153.5346 149.1340 136.2452 132.2490 132.1630 130.1684 130.1338 124.8190 124.2404 124.2078 123.4154 123.2984 122.7219 116.3261 116.1018 110.2733
77.4778 77.1601 76.8423
53.2575 53.1976 45.0955 45.0811
35.2873 28.3533 24.9562 21.8470
F
N
N
N
10
fl (ppm)

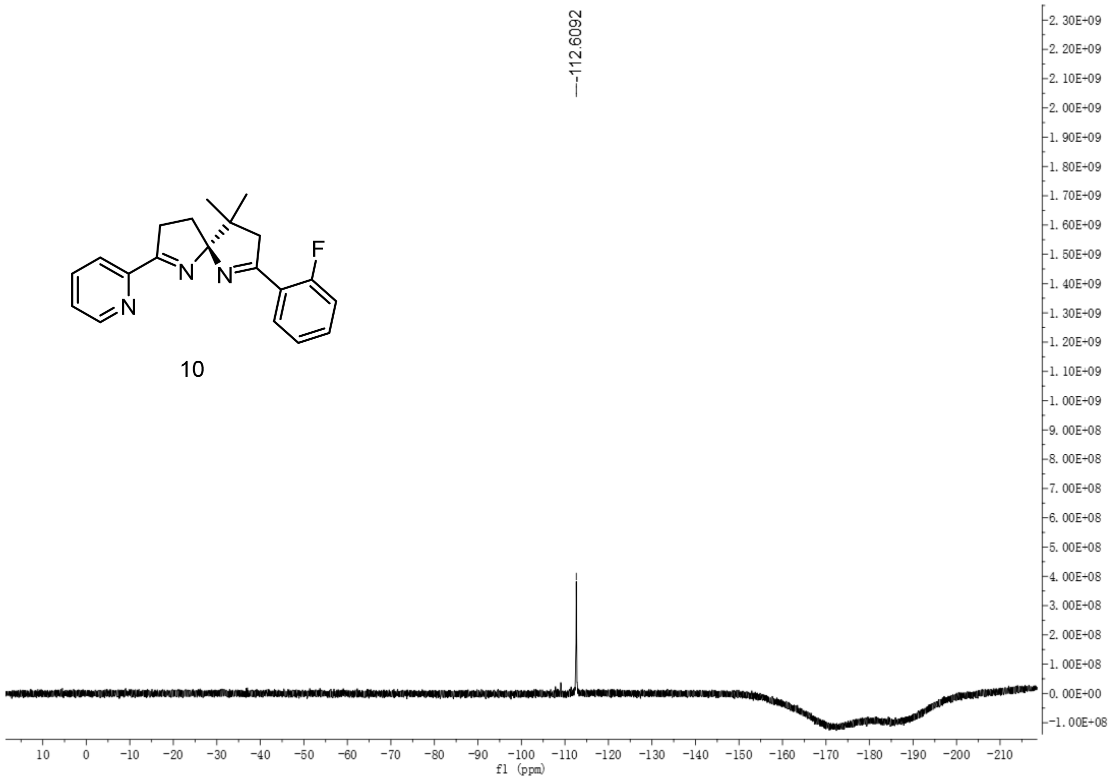

-112.6092
F
N
N
N
10
f1 (ppm)

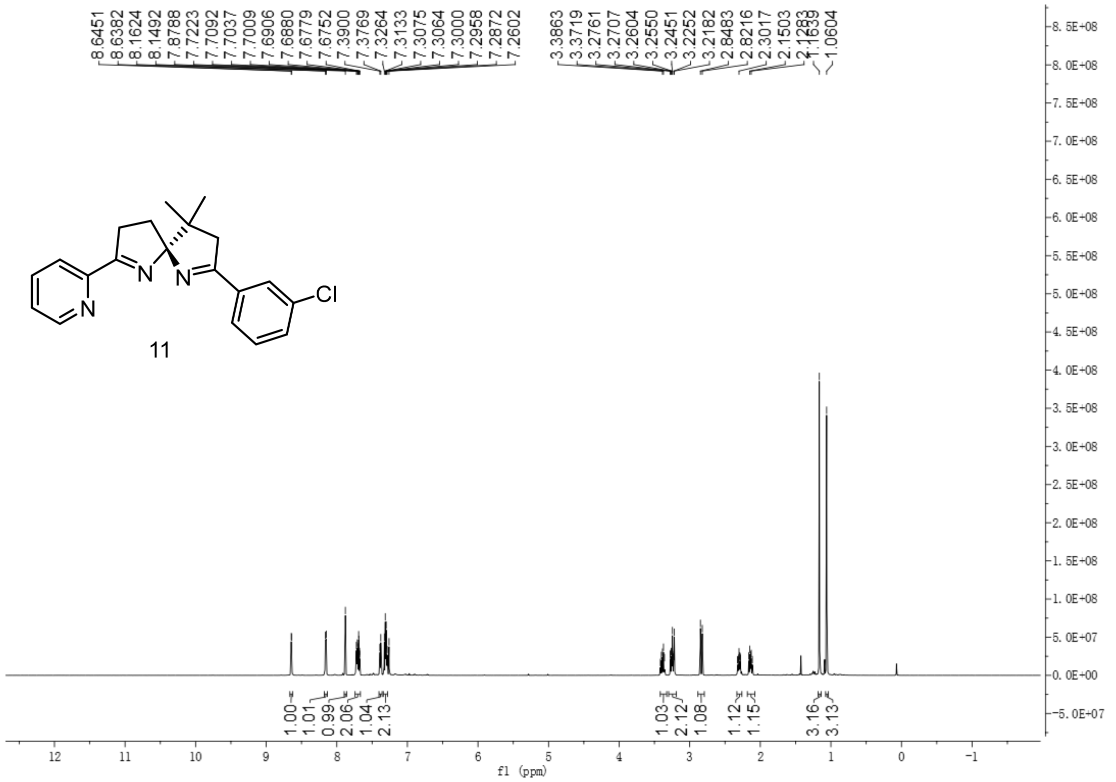

8.6451
8.6382
8.1624
8.1492
7.8788
7.7223
7.7092
7.7037
7.7009
7.6906
7.6880
7.6779
7.6752
7.3900
7.3769
7.3264
7.3133
7.3075
7.3064
7.3000
7.2958
7.2872
7.2602
3.3863
3.3719
3.2761
3.2707
3.2604
3.2550
3.2451
3.2252
3.2182
2.8483
2.8216
2.3017
2.1503
2.1283
1.1639
1.0604
Cl
N
N
N
11
1.00
1.01
0.99
2.06
1.04
2.13
1.03
2.12
1.08
1.12
1.15
3.16
3.13
f1 (ppm)

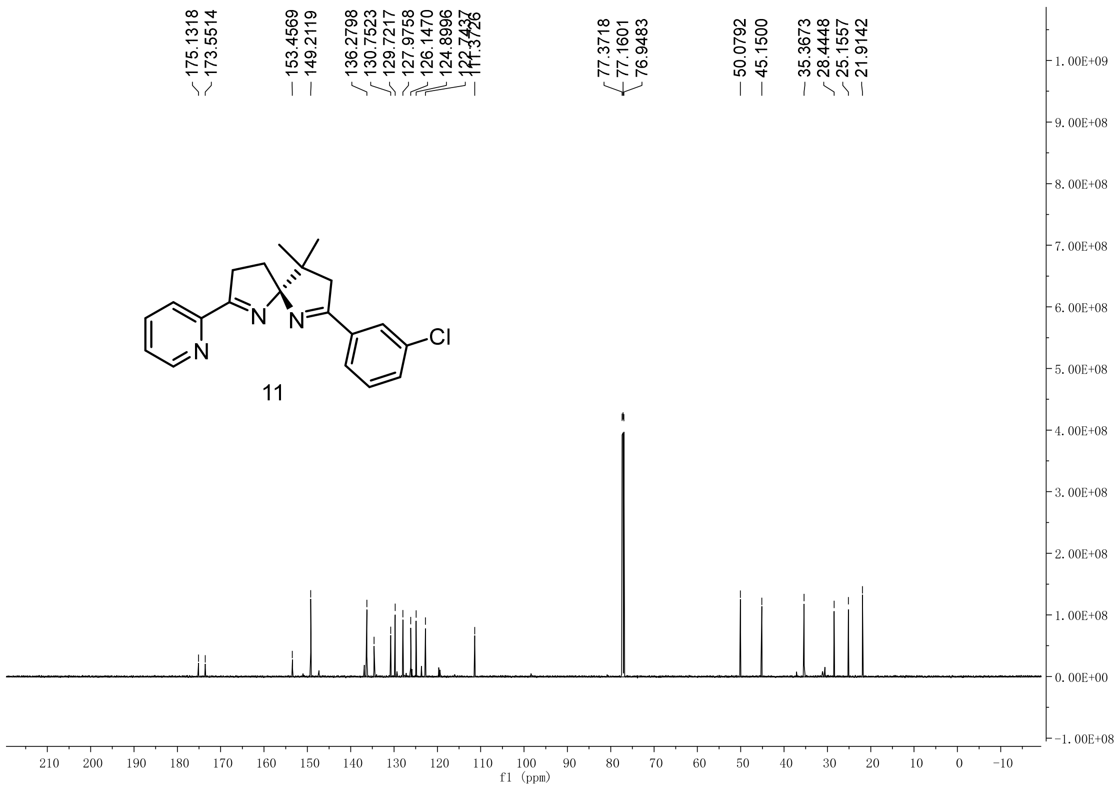

11

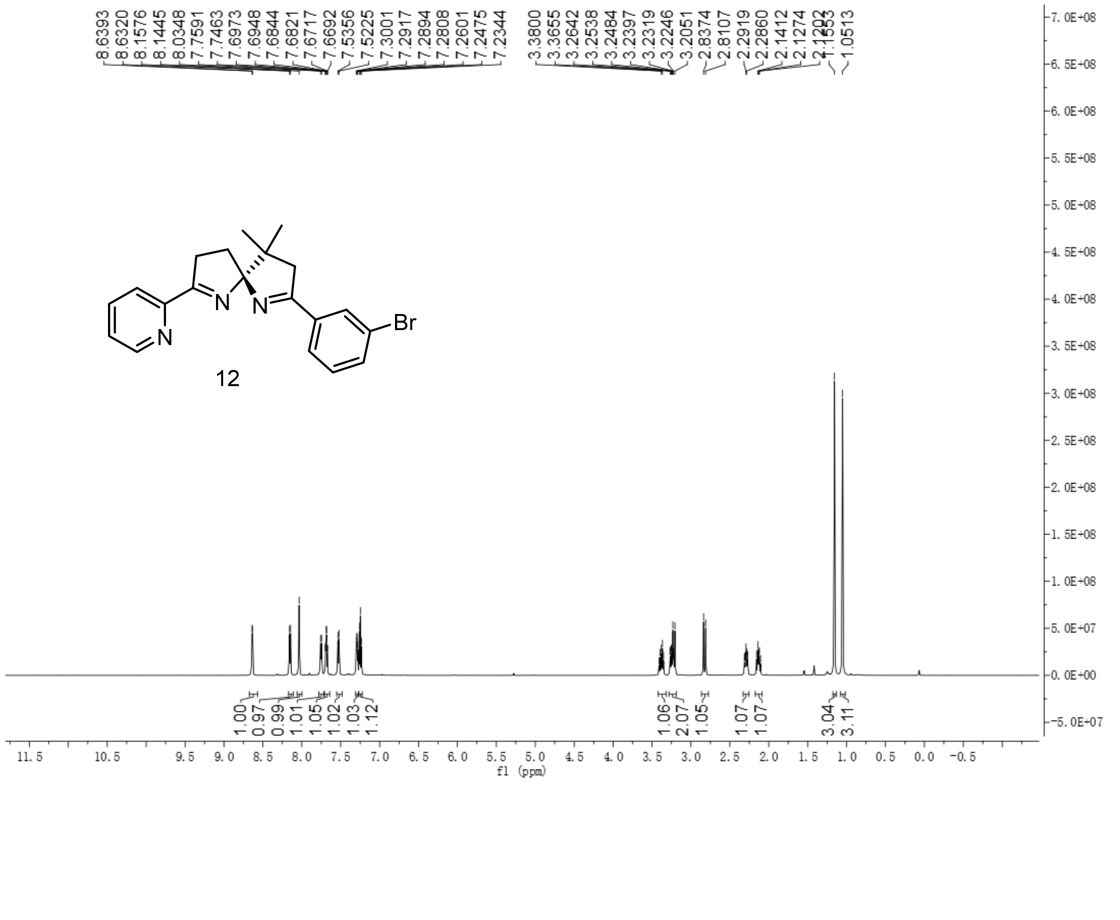

12

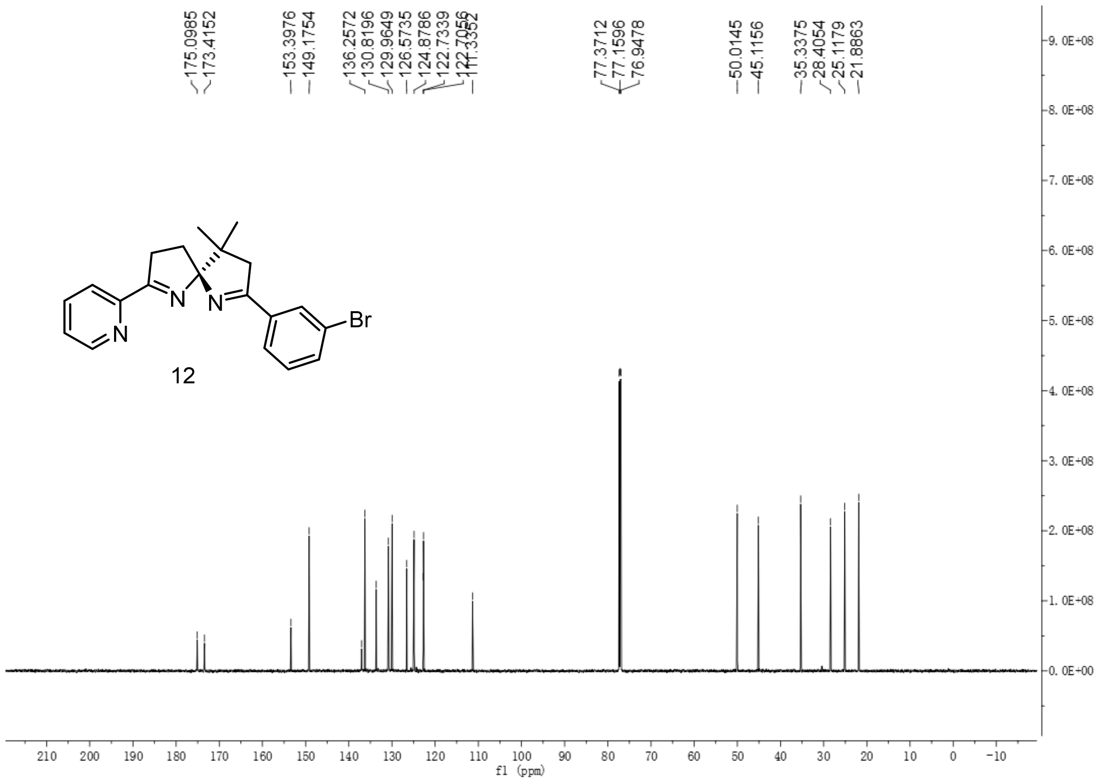

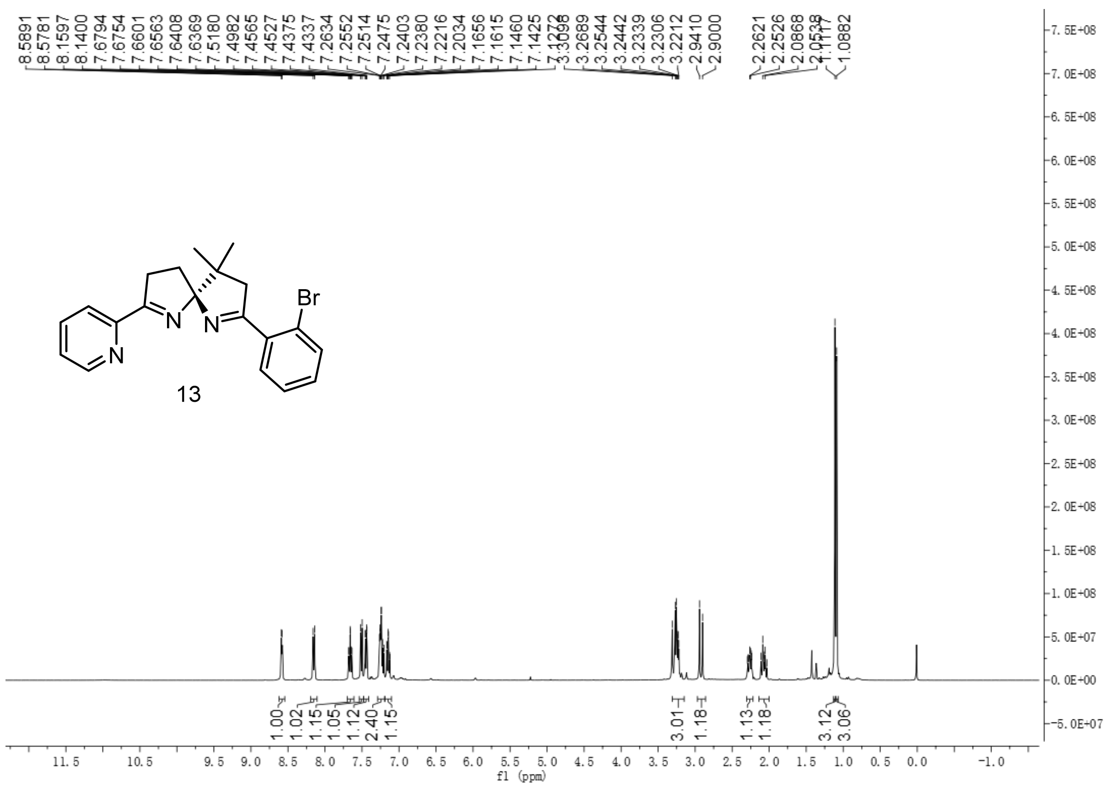

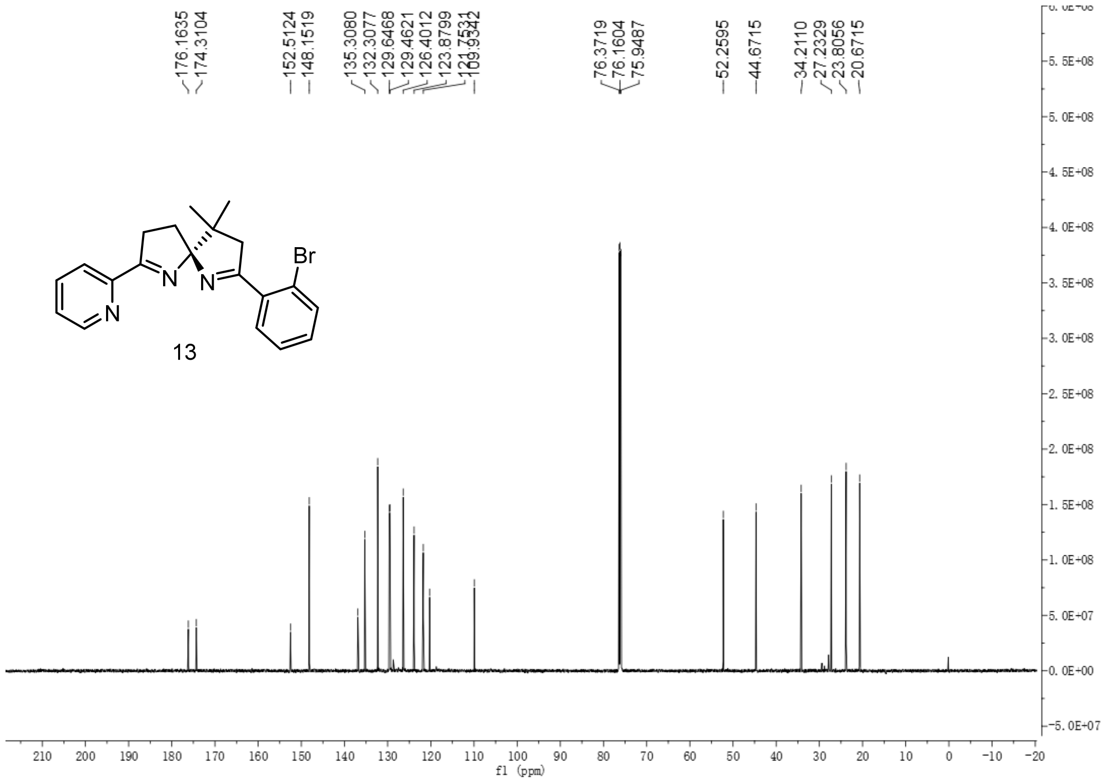

176.1635
174.3104
152.5124
148.1519
135.3080
132.3077
129.6468
129.4621
126.4012
123.8799
76.3719
76.1604
75.9487
52.2595
44.6715
34.2110
27.2329
23.8056
20.6715
Br
N
13
fl (ppm)

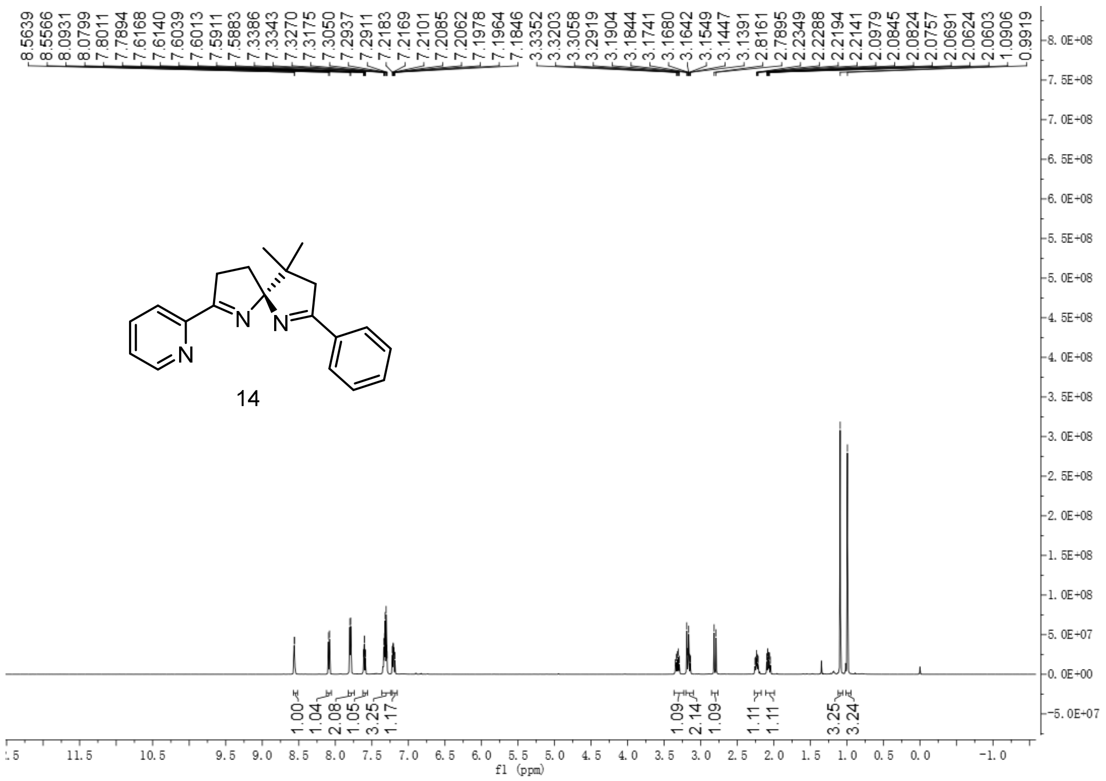

8.5639
8.5566
8.0931
8.0799
7.8011
7.7894
7.6168
7.6140
7.6039
7.6013
7.5911
7.5883
7.3386
7.3343
7.3270
7.3175
7.3050
7.2937
7.2911
7.2183
7.2169
7.2101
7.2085
7.2062
7.1978
7.1964
7.1846
3.3352
3.3203
3.3058
3.2919
3.1904
3.1844
3.1741
3.1680
3.1642
3.1549
3.1447
3.1391
2.8161
2.7895
2.2349
2.2288
2.2194
2.2141
2.0979
2.0845
2.0824
2.0757
2.0691
2.0624
2.0603
1.0906
0.9919
N
14
1.00
1.04
2.08
1.05
3.25
1.17
1.09
2.14
1.09
1.11
1.11
3.25
3.24
fl (ppm)

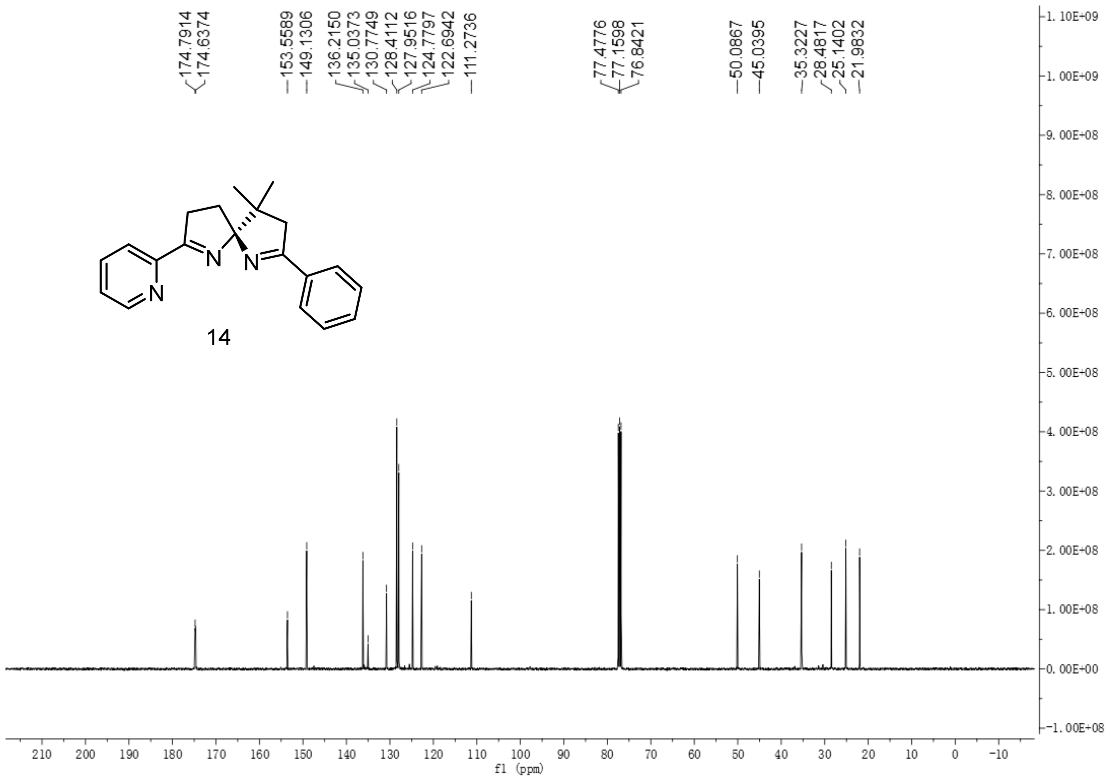
174.7914
174.6374
153.5589
149.1306
136.2150
135.0373
130.7749
128.4112
127.9516
124.7797
122.6942
111.2736
77.4776
77.1598
76.8421
50.0867
45.0395
35.3227
28.4817
25.1402
21.9832
14
f1 (ppm)

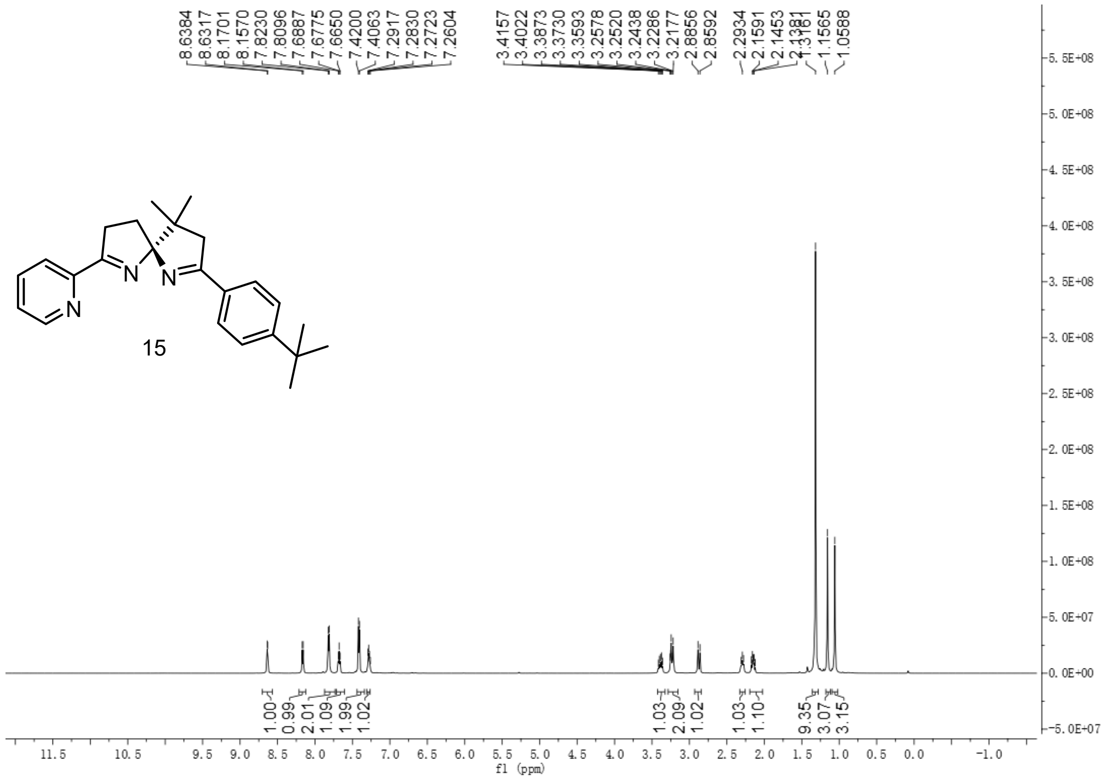
8.6384
8.6317
8.1701
8.1570
7.8230
7.8096
7.6887
7.6775
7.6650
7.4200
7.4063
7.2917
7.2830
7.2723
7.2604
3.4157
3.4022
3.3873
3.3730
3.3593
3.2578
3.2520
3.2438
3.2286
3.2177
2.8856
2.8592
2.2934
2.1591
2.1453
1.1565
1.0588
15
1.00
0.99
2.01
1.09
1.99
1.02
1.03
2.09
1.02
1.03
1.10
9.35
3.07
3.15
f1 (ppm)

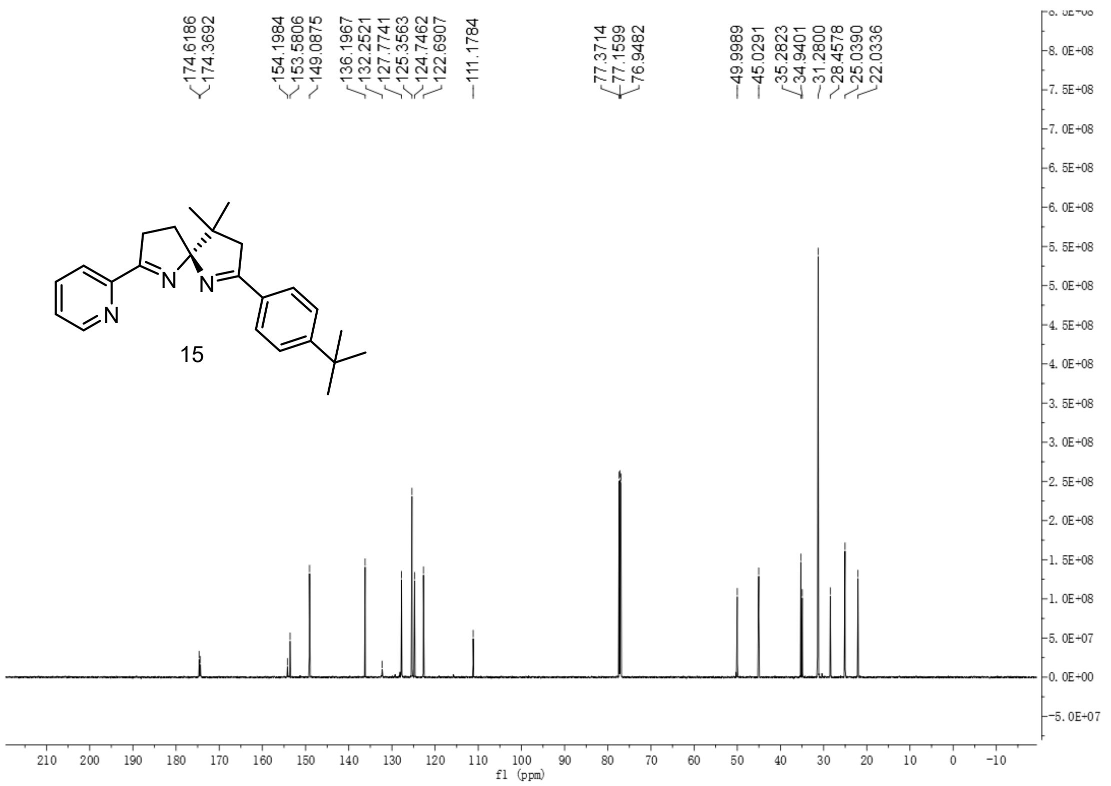
174.6186
174.3692
154.1984
153.5806
149.0875
136.1967
132.2521
127.7741
125.3563
124.7462
122.6907
111.1784
77.3714
77.1599
76.9482
49.9989
45.0291
35.2823
34.9401
31.2800
28.4578
25.0390
22.0336
15
N
N
N
fl (ppm)

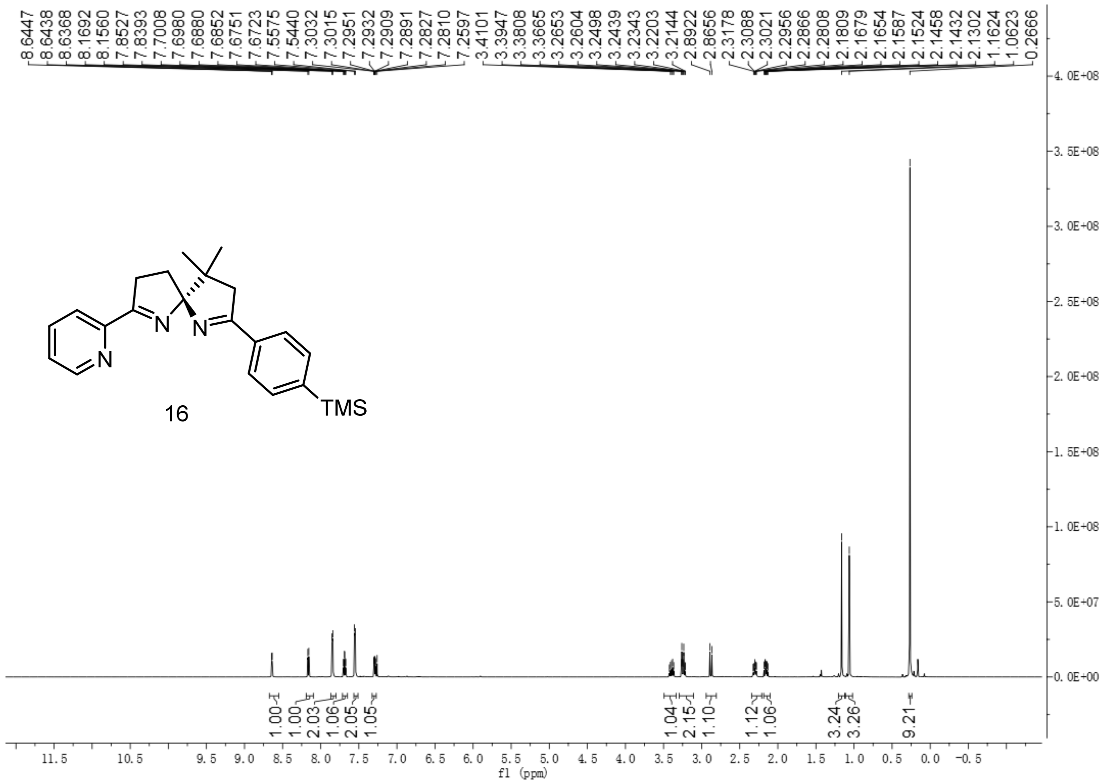
8.6447
8.6438
8.6368
8.1692
8.1560
7.8527
7.8393
7.7008
7.6980
7.6880
7.6852
7.6723
7.6751
7.5575
7.5440
7.3032
7.3015
7.2951
7.2932
7.2909
7.2891
7.2827
7.2810
7.2597
3.4101
3.3947
3.3808
3.3665
3.2653
3.2604
3.2498
3.2439
3.2343
3.2203
3.2144
2.8922
2.8656
2.3178
2.3088
2.3021
2.2956
2.2866
2.2808
2.1809
2.1679
2.1654
2.1587
2.1524
2.1458
2.1432
2.1302
1.1624
1.0623
0.2666
16
TMS
N
N
N
1.00
1.00
2.03
1.06
2.05
1.05
1.04
2.15
1.10
1.12
1.06
3.24
3.26
9.21
fl (ppm)

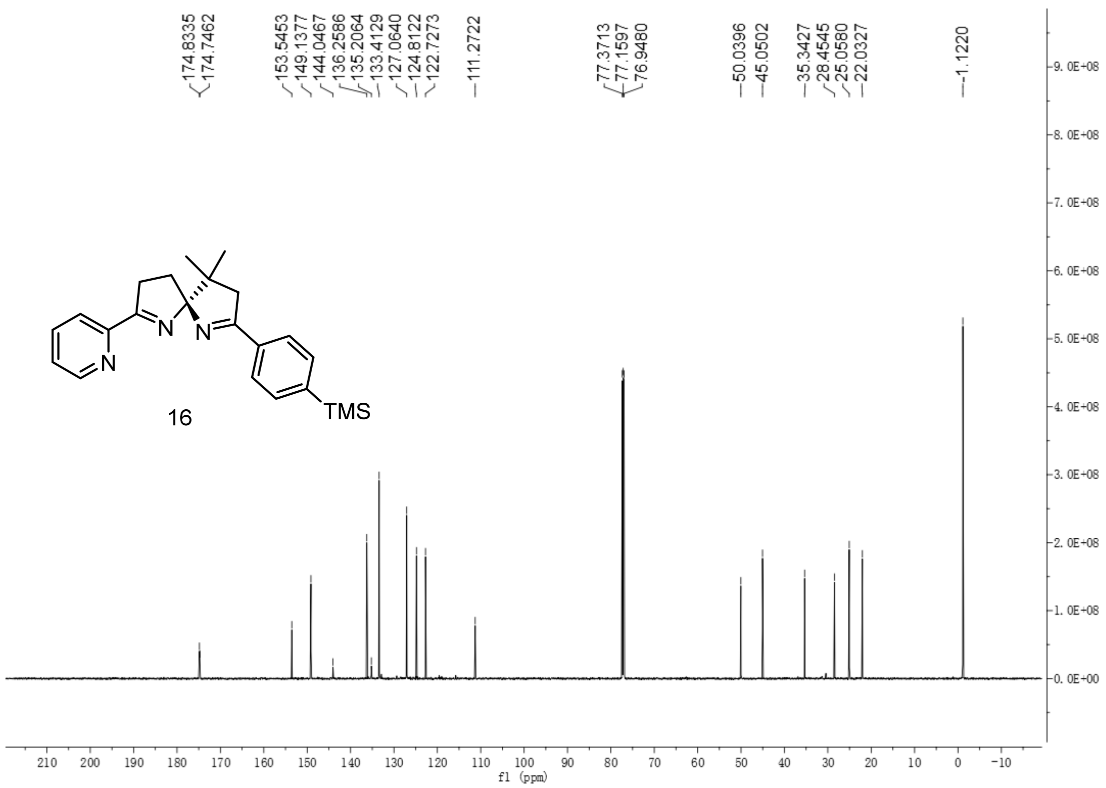
174.8335
174.7462
153.5453
149.1377
144.0467
136.2586
135.2064
133.4129
127.0640
124.8122
122.7273
111.2722
77.3713
77.1597
76.9480
50.0396
45.0502
35.3427
28.4545
25.0580
22.0327
-1.1220
TMS
16
9.0E+08
8.0E+08
7.0E+08
6.0E+08
5.0E+08
4.0E+08
3.0E+08
2.0E+08
1.0E+08
0.0E+00
210 200 190 180 170 160 150 140 130 120 110 100 90 80 70 60 50 40 30 20 10 0 -10
f1 (ppm)

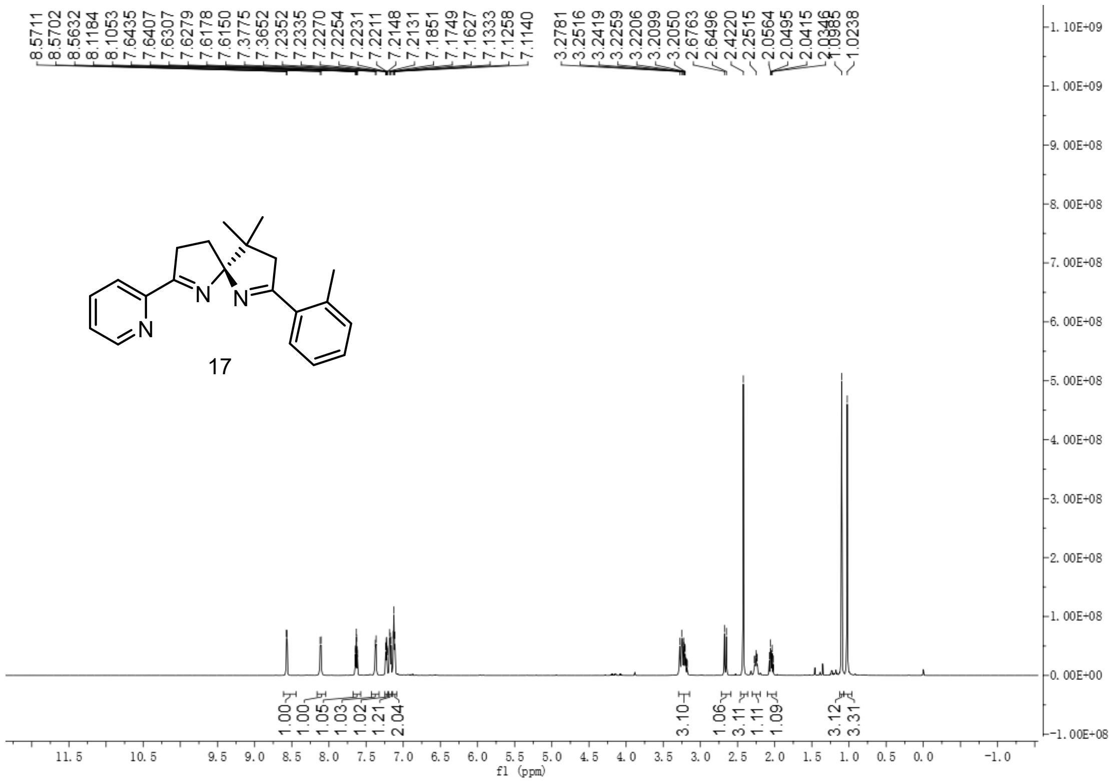
8.5711
8.5702
8.5632
8.1184
8.1053
7.6435
7.6407
7.6307
7.6279
7.6178
7.6150
7.3775
7.3652
7.2352
7.2335
7.2270
7.2254
7.2231
7.2211
7.2148
7.2131
7.1851
7.1749
7.1627
7.1333
7.1258
7.1140
3.2781
3.2516
3.2419
3.2259
3.2206
3.2099
3.2050
2.6763
2.6496
2.4220
2.2515
2.0564
2.0495
2.0415
2.0346
1.0985
1.0238
17
1.10E+09
1.00E+09
9.00E+08
8.00E+08
7.00E+08
6.00E+08
5.00E+08
4.00E+08
3.00E+08
2.00E+08
1.00E+08
0.00E+00
-1.00E+08
1.00
1.00
1.05
1.03
1.02
1.21
2.04
3.10
1.06
3.11
1.11
1.09
3.12
3.31
11.5 10.5 9.5 9.0 8.5 8.0 7.5 7.0 6.5 6.0 5.5 5.0 4.5 4.0 3.5 3.0 2.5 2.0 1.5 1.0 0.5 0.0 -1.0
f1 (ppm)

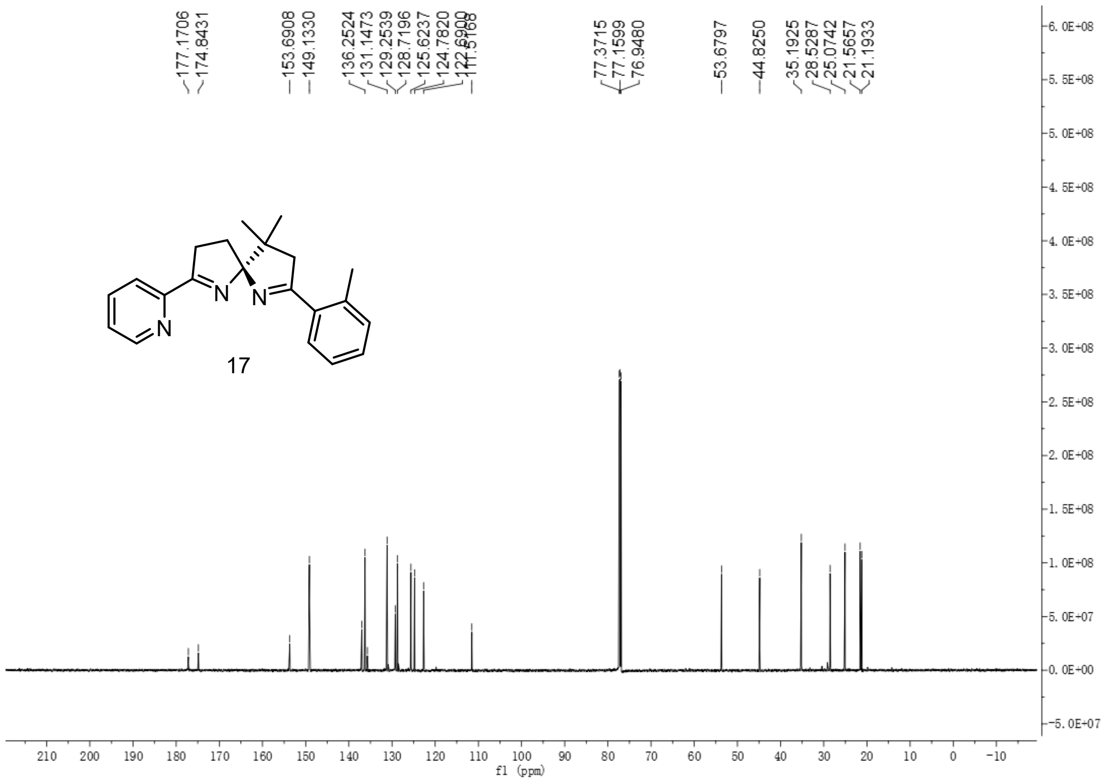
177.1706
174.8431
153.6908
149.1330
136.2524
131.1473
129.2539
128.7196
125.6237
124.7820
122.6900
111.5168
77.3715
77.1599
76.9480
53.6797
44.8250
35.1925
28.5287
25.0742
21.5657
21.1933
17
fl (ppm)

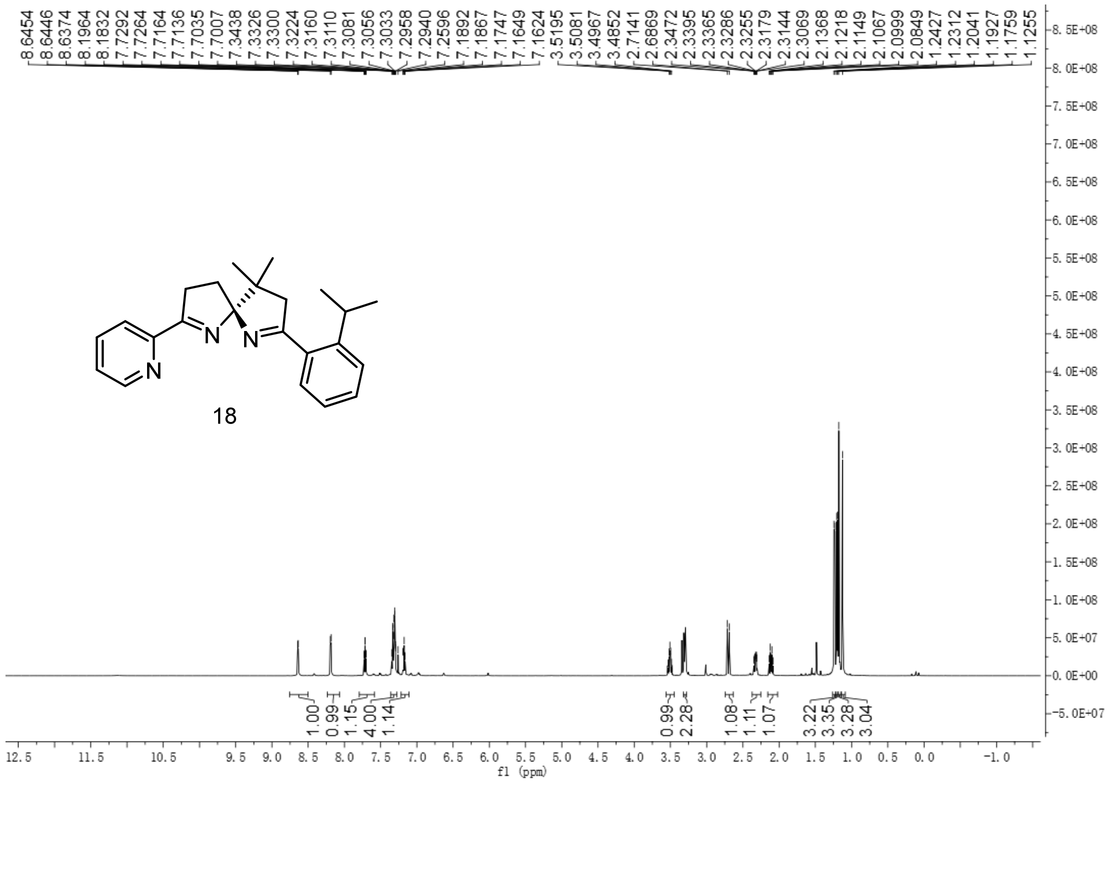
8.6454
8.6446
8.6374
8.1964
8.1832
7.7292
7.7264
7.7164
7.7136
7.7035
7.7007
7.3438
7.3326
7.3300
7.3224
7.3160
7.3110
7.3081
7.3056
7.3033
7.2958
7.2940
7.2596
7.1892
7.1867
7.1747
7.1649
7.1624
3.5195
3.5081
3.4967
3.4852
2.7141
2.6869
2.3472
2.3395
2.3365
2.3286
2.3255
2.3179
2.3144
2.3069
2.1368
2.1218
2.1149
2.1067
2.0999
2.0849
1.2427
1.2312
1.2041
1.1927
1.1759
1.1255
18
1.00
0.99
1.15
4.00
1.14
0.99
2.28
1.08
1.11
1.07
3.22
3.35
3.28
3.04
fl (ppm)

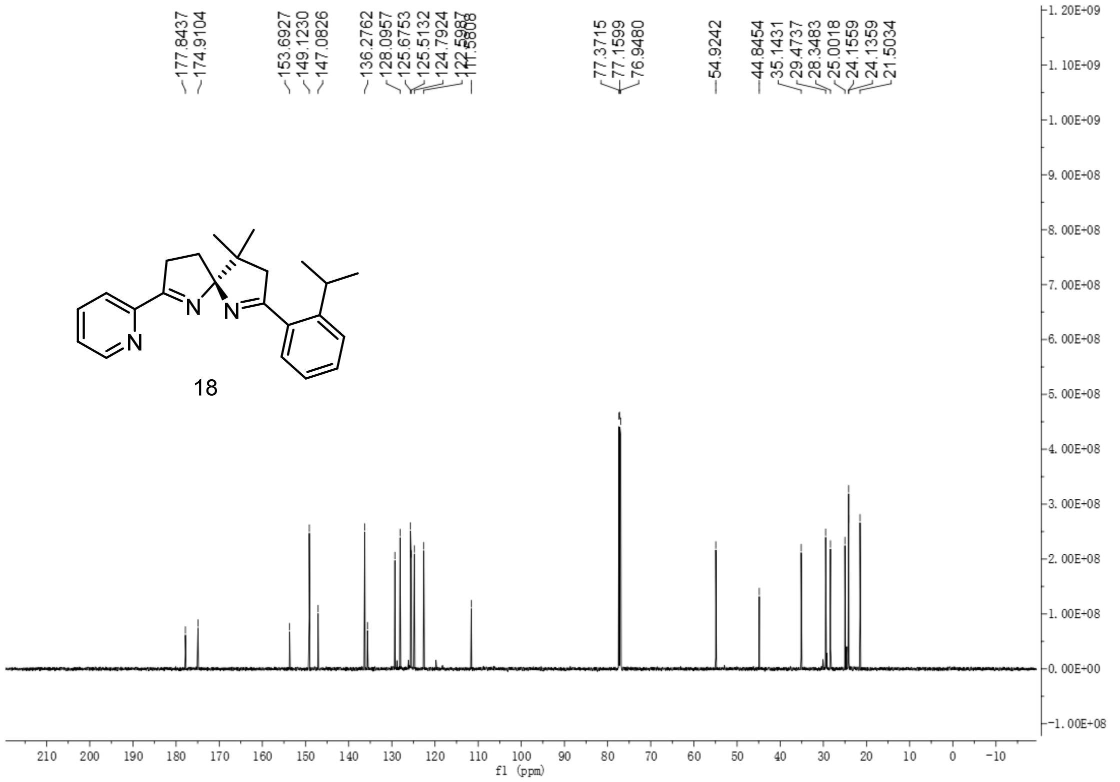
177.8437
174.9104
153.6927
149.1230
147.0826
136.2762
128.0957
125.6753
125.5132
124.7924
122.5987
[illegible]
77.3715
77.1599
76.9480
54.9242
44.8454
35.1431
29.4737
28.3483
25.0018
24.1559
24.1359
21.5034
N
N
N
18
fl (ppm)

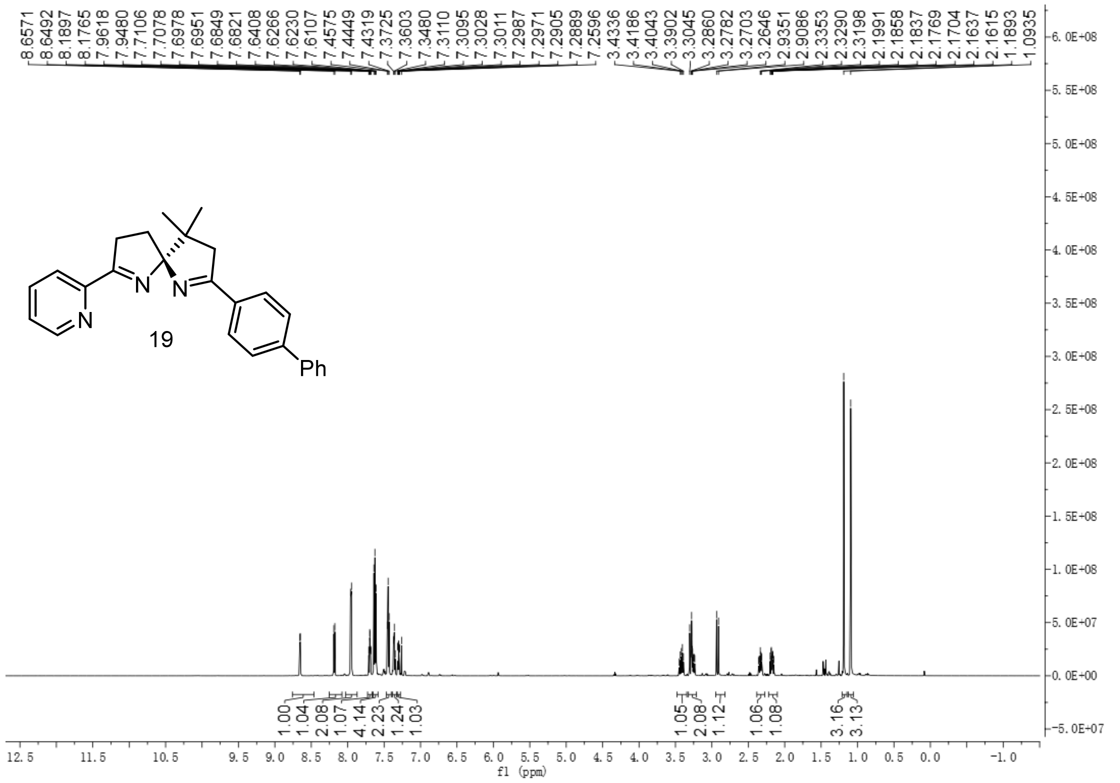
8.6571
8.6492
8.1897
8.1765
7.9618
7.9480
7.7106
7.7078
7.6978
7.6951
7.6849
7.6821
7.6408
7.6266
7.6230
7.6107
7.4575
7.4449
7.4319
7.3725
7.3603
7.3480
7.3110
7.3095
7.3028
7.3011
7.2987
7.2971
7.2905
7.2889
7.2596
3.4336
3.4186
3.4043
3.3902
3.3045
3.2860
3.2782
3.2703
3.2646
2.9351
2.9086
2.3353
2.3290
2.3198
2.1991
2.1858
2.1837
2.1769
2.1704
2.1637
2.1615
1.1893
1.0935
N
N
N
19
Ph
1.00
1.04
2.08
1.07
4.14
2.23
1.24
1.03
1.05
2.08
1.12
1.06
1.08
3.16
3.13
fl (ppm)

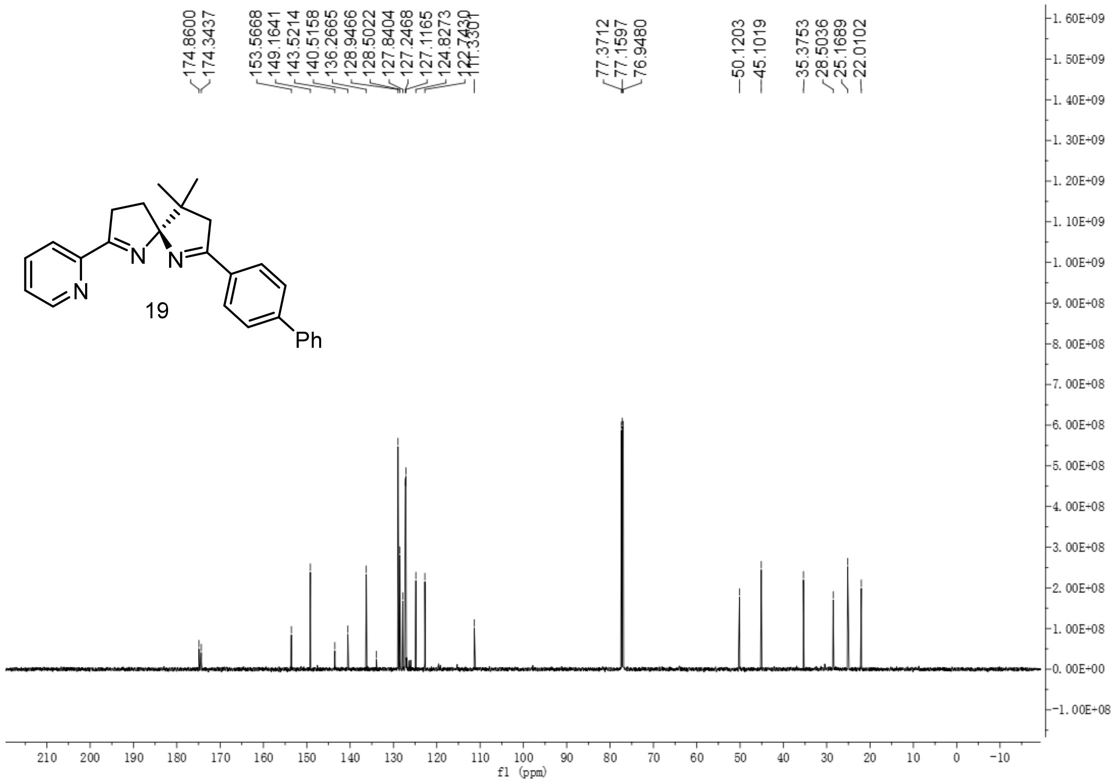

19

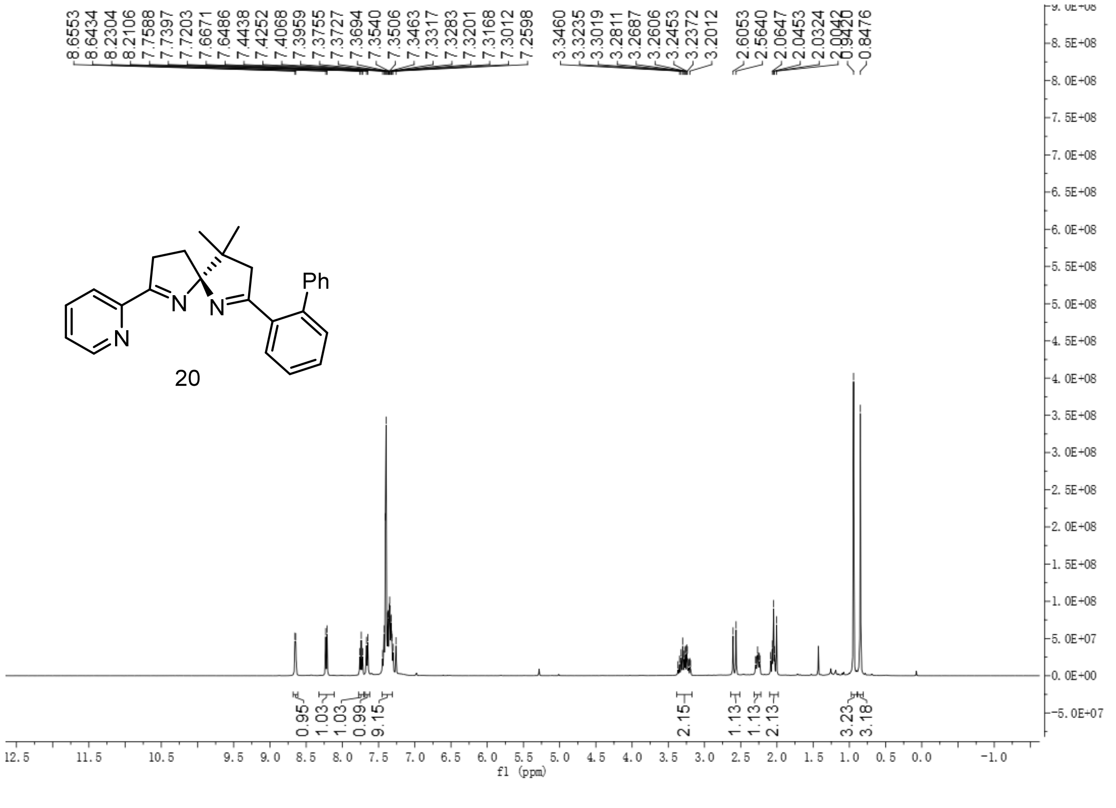

20

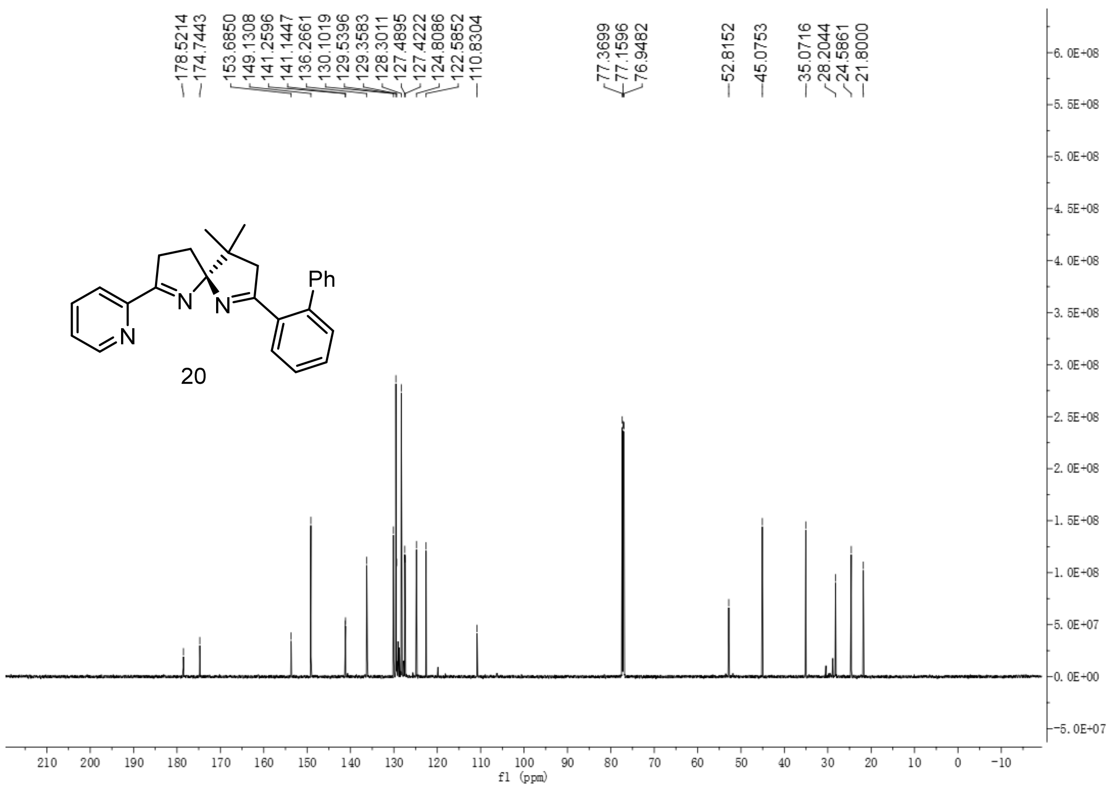

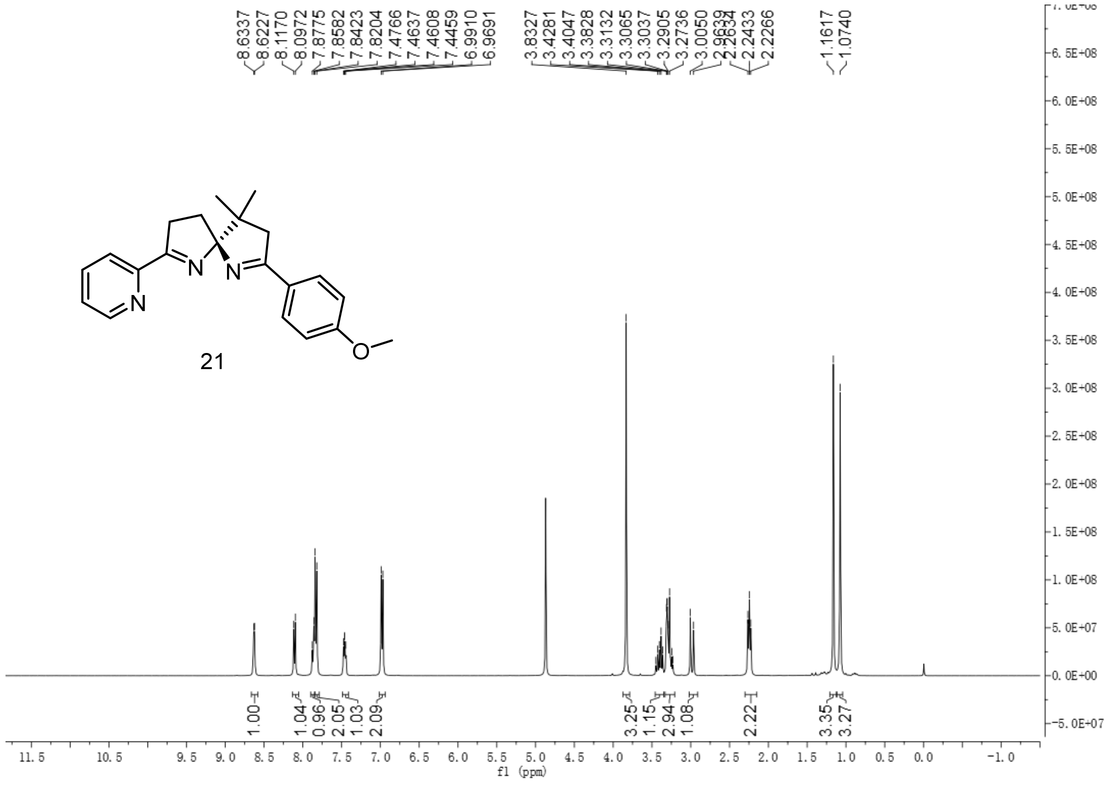

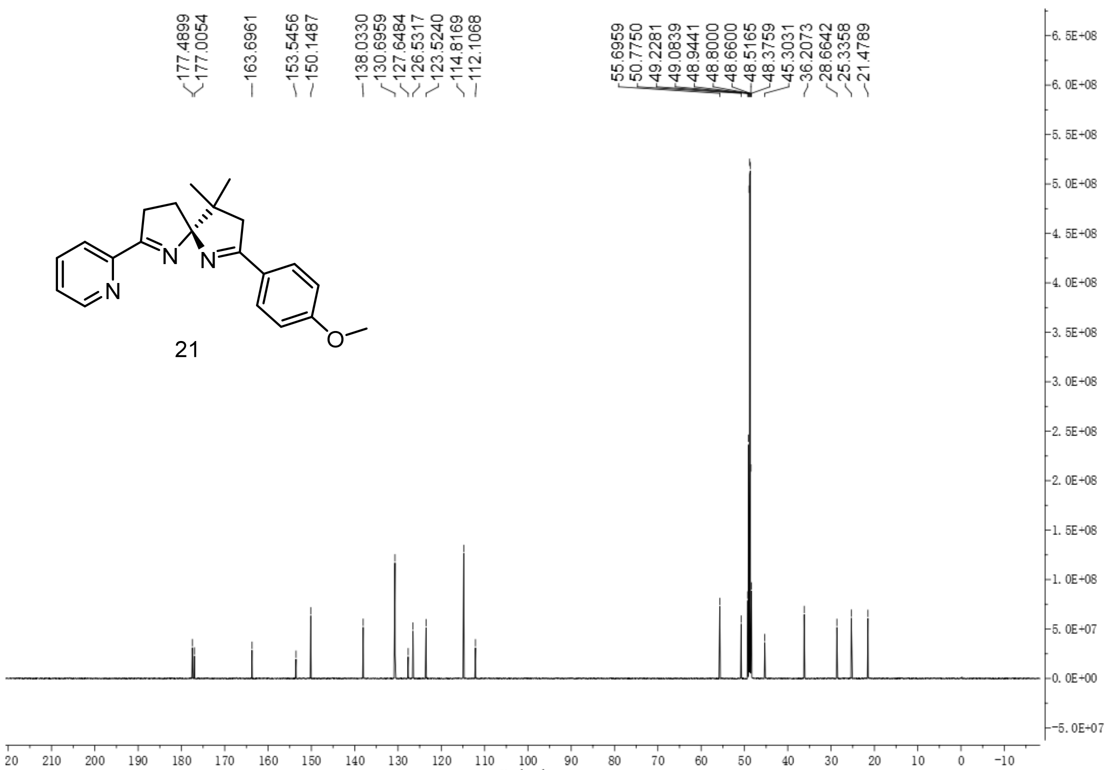
177.4899
177.0054
163.6961
153.5456
150.1487
138.0330
130.6959
127.6484
126.5317
123.5240
114.8169
112.1068
55.6959
50.7750
49.2281
49.0839
48.9441
48.8000
48.6600
48.5165
48.3759
45.3031
36.2073
28.6642
25.3358
21.4789
N
N
N
O
21
fl (ppm)

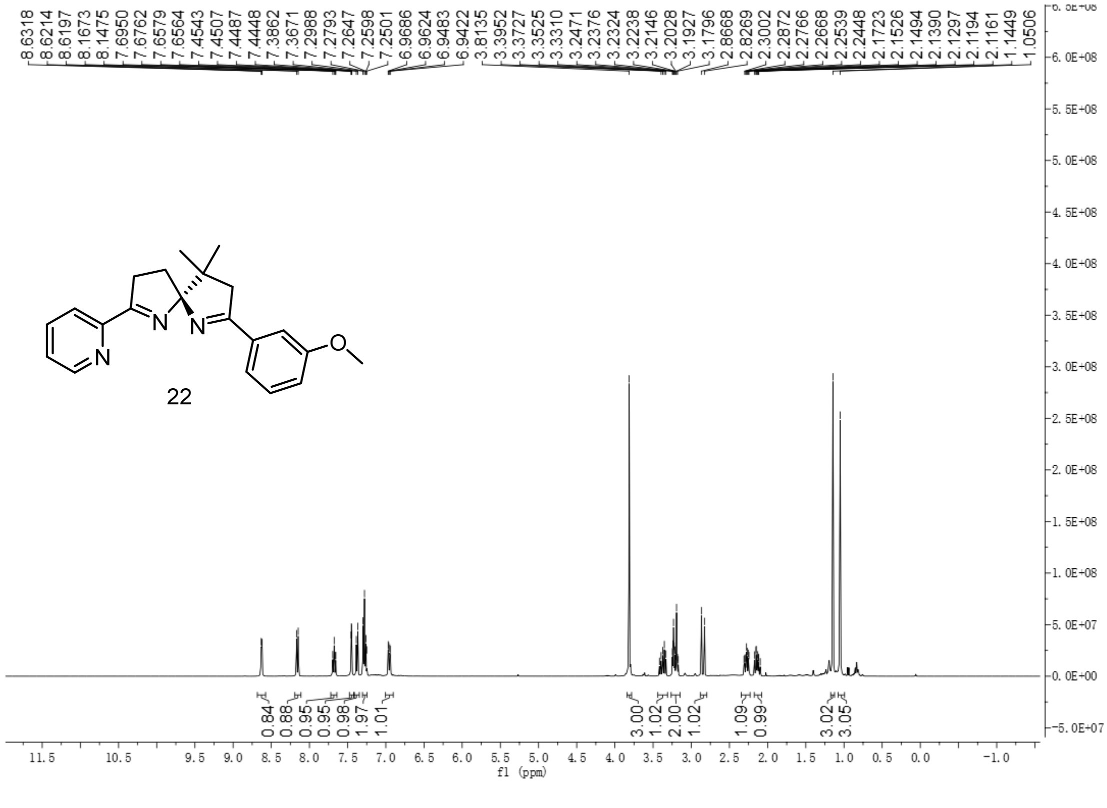
8.6318
8.6214
8.6197
8.1673
8.1475
7.6950
7.6762
7.6579
7.6564
7.4543
7.4507
7.4487
7.4448
7.3862
7.3671
7.2988
7.2793
7.2647
7.2598
7.2501
6.9686
6.9624
6.9483
6.9422
3.8135
3.3952
3.3727
3.3525
3.3310
3.2471
3.2376
3.2324
3.2238
3.2146
3.2028
3.1927
3.1796
2.8668
2.8269
2.3002
2.2872
2.2766
2.2668
2.2539
2.2448
2.1723
2.1526
2.1494
2.1390
2.1297
2.1194
2.1161
1.1449
1.0506
N
N
N
O
22
0.84
0.88
0.95
0.95
0.98
1.97
1.01
3.00
1.02
2.00
1.02
1.09
0.99
3.02
3.05
fl (ppm)

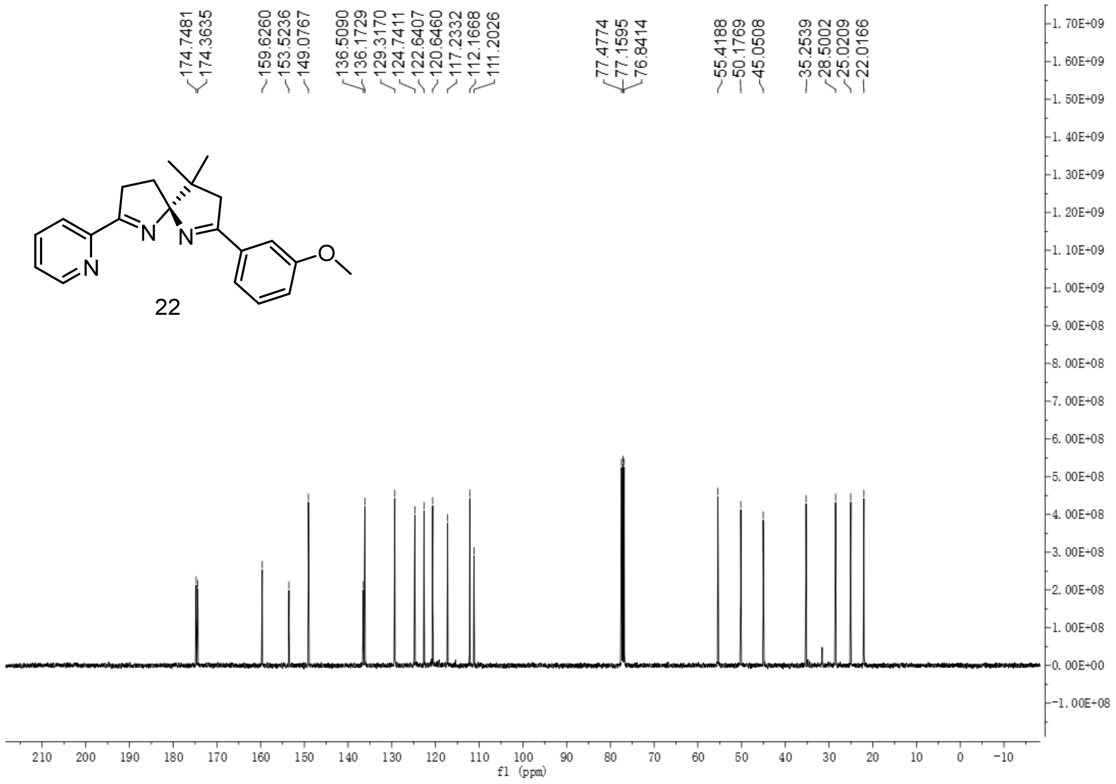

174.7481
174.3635
159.6260
153.5236
149.0767
136.5090
136.1729
129.3170
124.7411
122.6407
120.6460
117.2332
112.1668
111.2026
77.4774
77.1595
76.8414
55.4188
50.1769
45.0508
35.2539
28.5002
25.0209
22.0166
22
f1 (ppm)

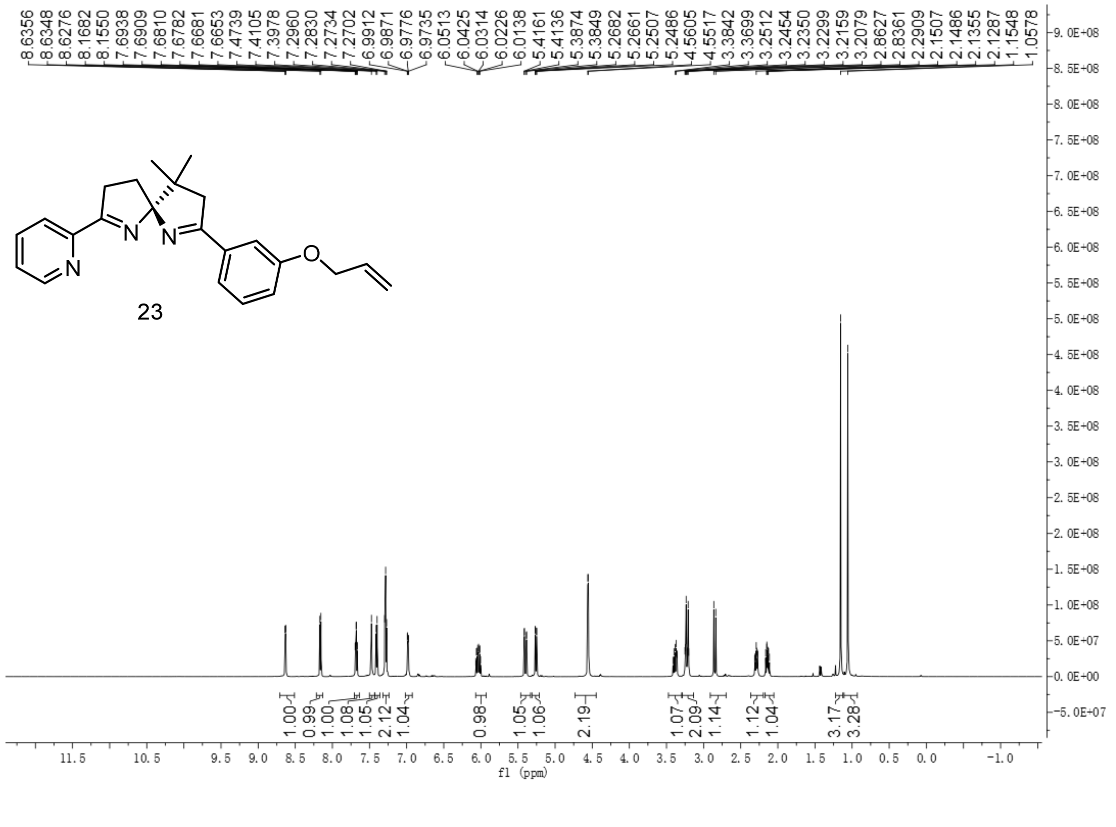

8.6356
8.6348
8.6276
8.1682
8.1550
7.6938
7.6909
7.6810
7.6782
7.6681
7.6653
7.4739
7.4105
7.3978
7.2960
7.2830
7.2734
7.2702
6.9912
6.9871
6.9776
6.9735
6.0513
6.0425
6.0314
6.0226
6.0138
5.4161
5.4136
5.3874
5.3849
5.2682
5.2661
5.2507
5.2486
4.5605
4.5517
3.3842
3.3699
3.2512
3.2454
3.2350
3.2299
3.2159
3.2079
2.8627
2.8361
2.2909
2.1507
2.1486
2.1355
2.1287
1.1548
1.0578
23
f1 (ppm)

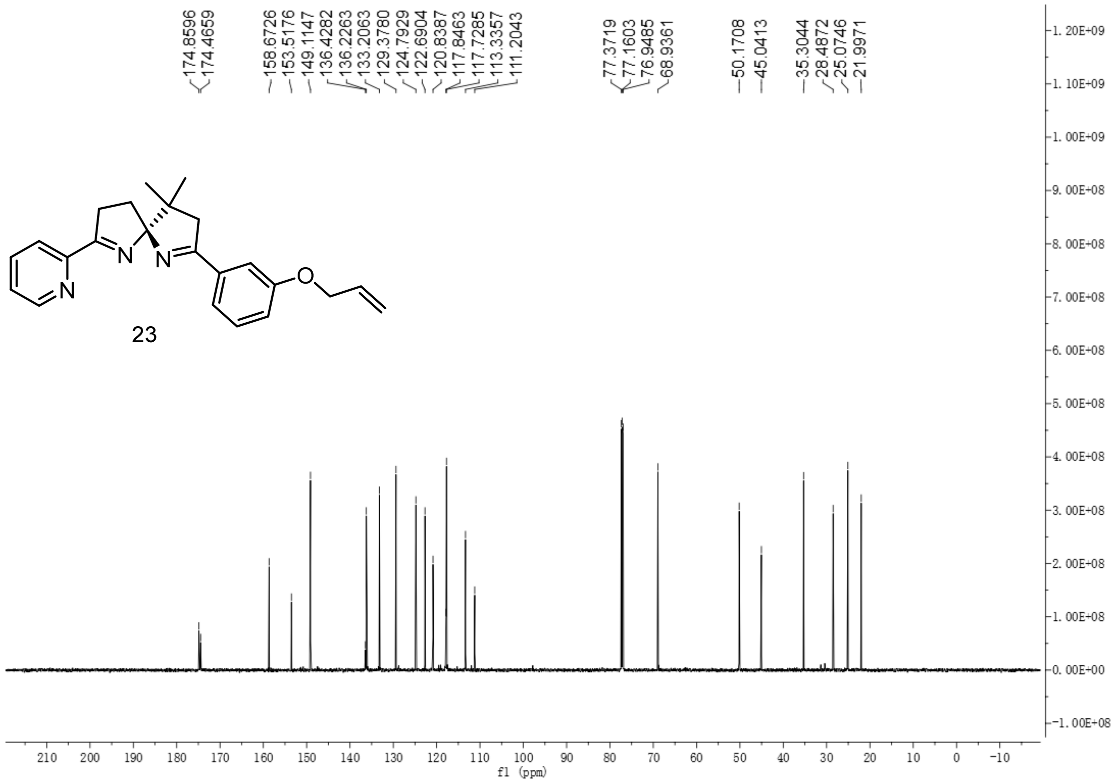
174.8596
174.4659
158.6726
153.5176
149.1147
136.4282
136.2263
133.2063
129.3780
124.7929
122.6904
120.8387
117.8463
117.7285
113.3357
111.2043
77.3719
77.1603
76.9485
68.9361
50.1708
45.0413
35.3044
28.4872
25.0746
21.9971
N
N
N
O
23
fl (ppm)

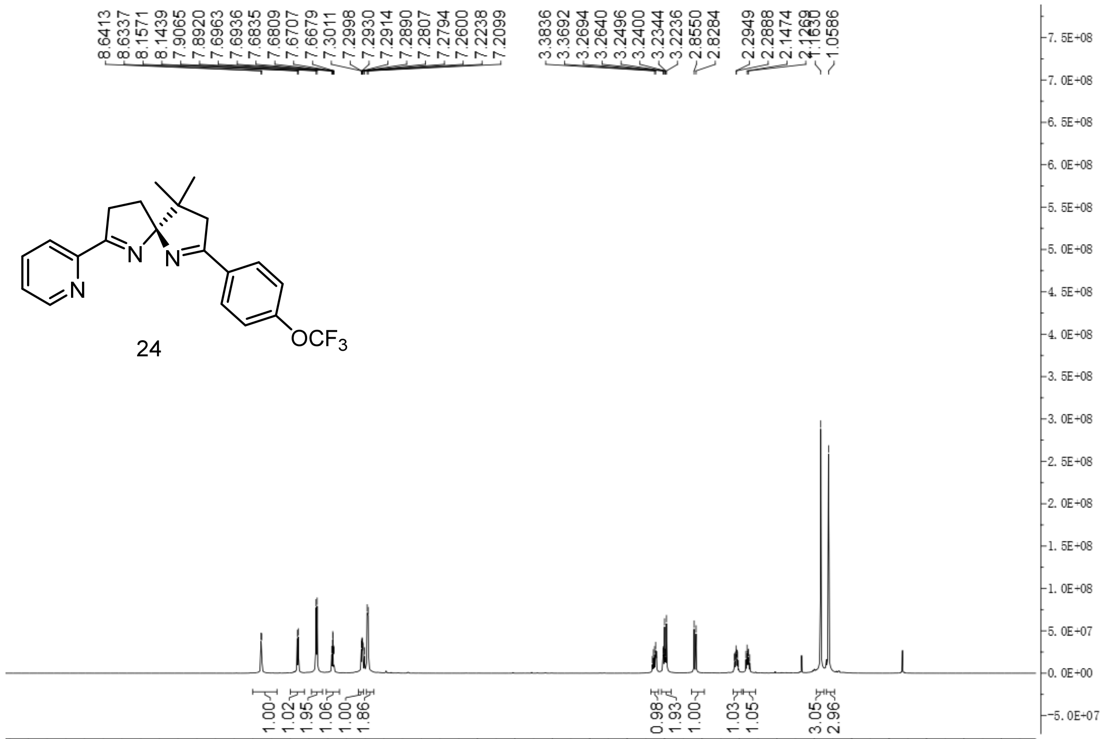
8.6413
8.6337
8.1571
8.1439
7.9065
7.8920
7.6963
7.6936
7.6835
7.6809
7.6707
7.6679
7.3011
7.2998
7.2930
7.2914
7.2890
7.2807
7.2794
7.2600
7.2238
7.2099
3.3836
3.3692
3.2694
3.2640
3.2496
3.2400
3.2344
3.2236
2.8550
2.8284
2.2949
2.2888
2.1474
N
N
N
OCF_3
24
1.00
1.02
1.95
1.06
1.00
1.86
0.98
1.93
1.00
1.03
1.05
3.05
2.96
fl (ppm)

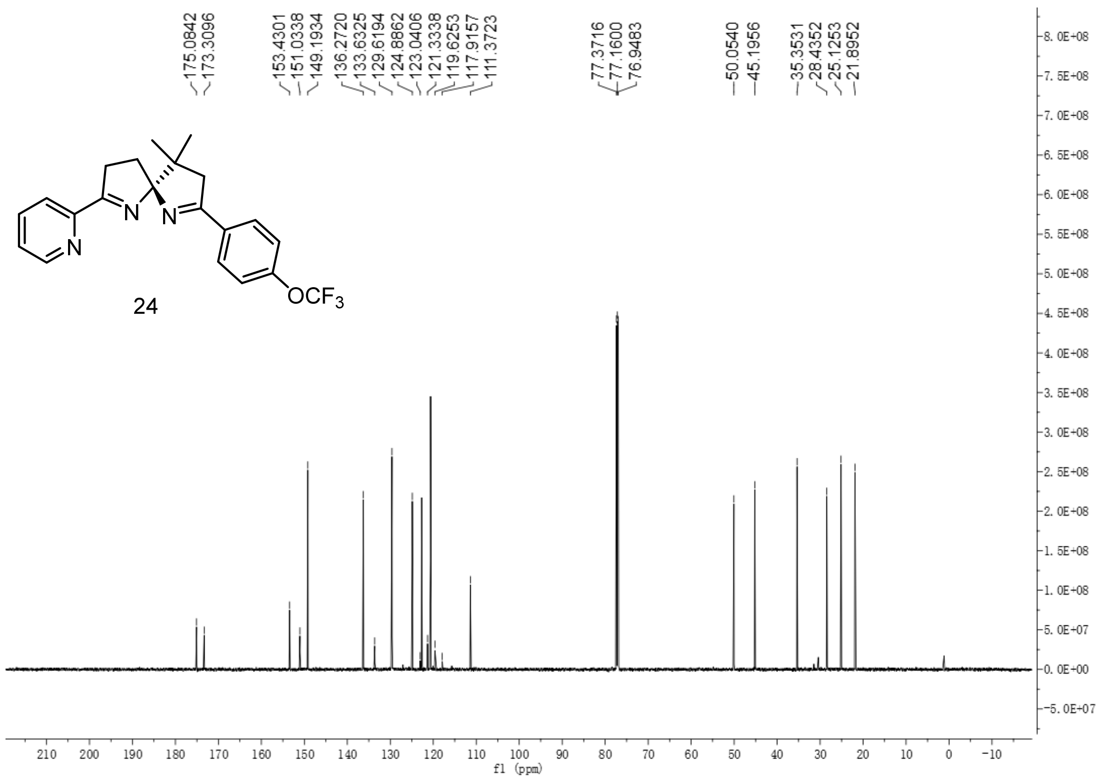
175.0842
173.3096
153.4301
151.0338
149.1934
136.2720
133.6325
129.6194
124.8862
123.0406
121.3338
119.6253
117.9157
111.3723
77.3716
77.1600
76.9483
50.0540
45.1956
35.3531
28.4352
25.1253
21.8952
N
N
N
OCF3
24
fl (ppm)

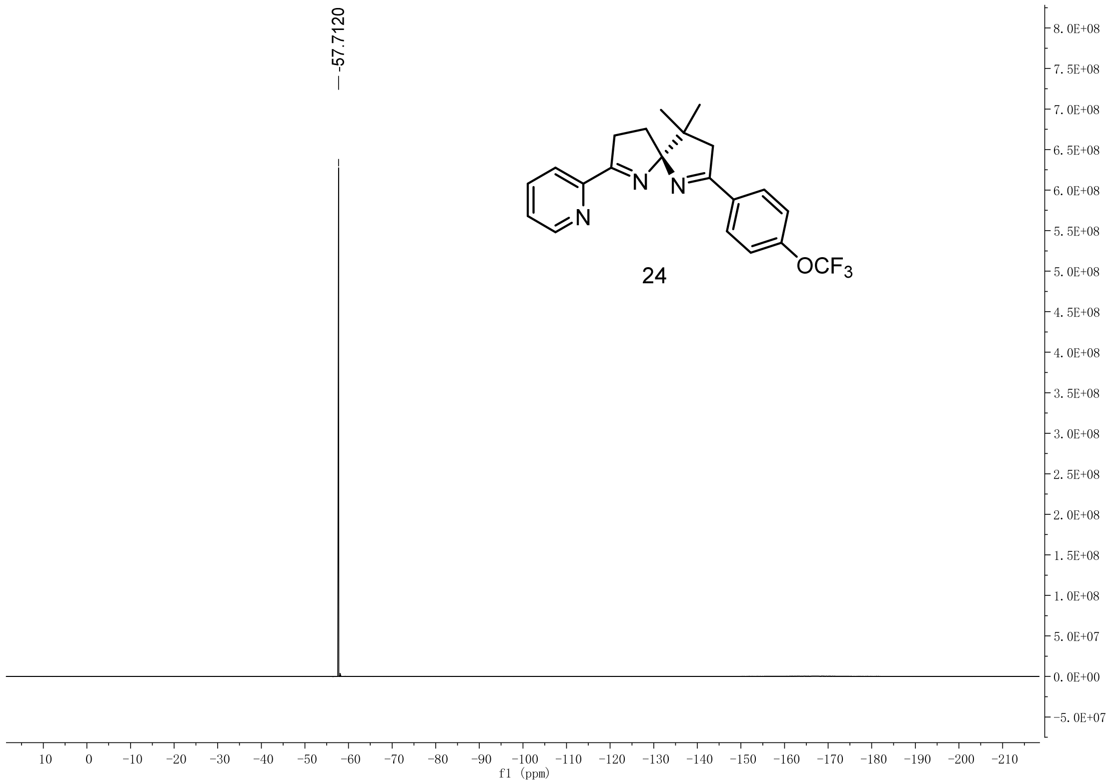

-57.7120
N
N
N
OCF3
24
f1 (ppm)

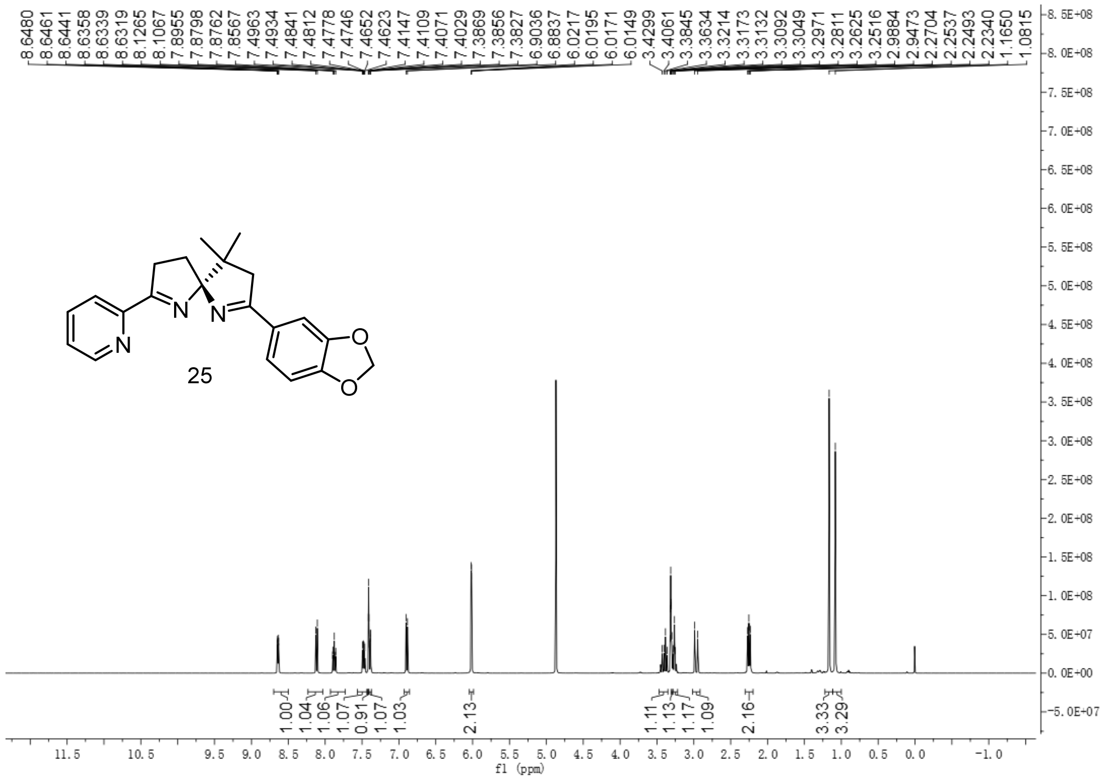

8.6480
8.6461
8.6441
8.6358
8.6339
8.6319
8.1265
8.1067
7.8955
7.8798
7.8762
7.8567
7.4963
7.4934
7.4841
7.4812
7.4778
7.4746
7.4652
7.4623
7.4147
7.4109
7.4071
7.4029
7.3869
7.3856
7.3827
6.9036
6.8837
6.0217
6.0195
6.0171
6.0149
3.4299
3.4061
3.3845
3.3634
3.3214
3.3173
3.3132
3.3092
3.3049
3.2971
3.2811
3.2625
3.2516
2.9884
2.9473
2.2704
2.2537
2.2493
2.2340
1.1650
1.0815
N
N
N
O
O
25
1.00
1.04
1.06
1.07
0.91
1.07
1.03
2.13
1.11
1.13
1.17
1.09
2.16
3.33
3.29
f1 (ppm)

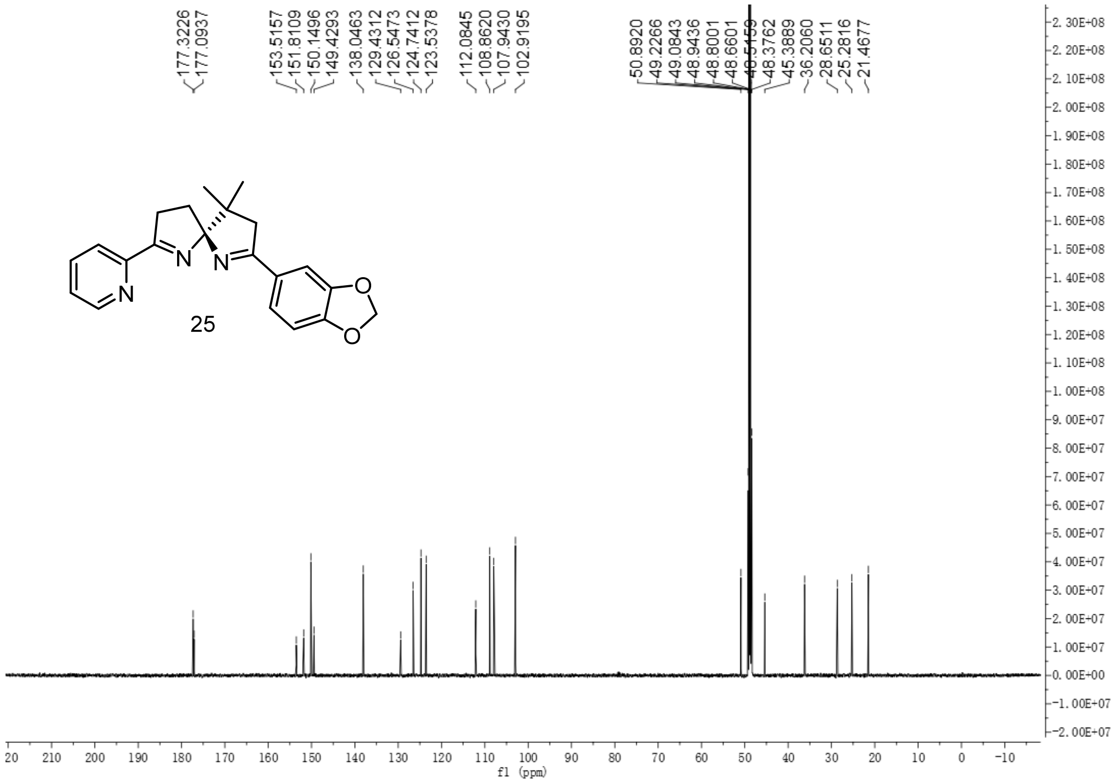

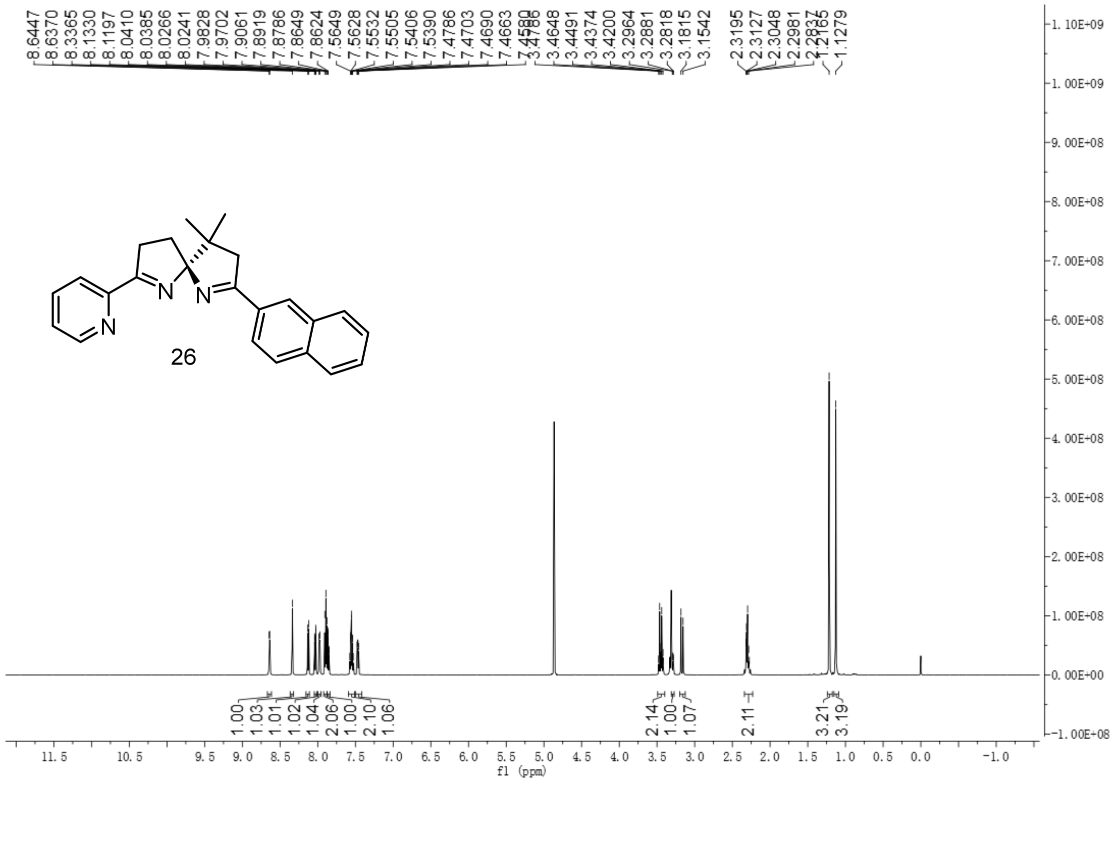

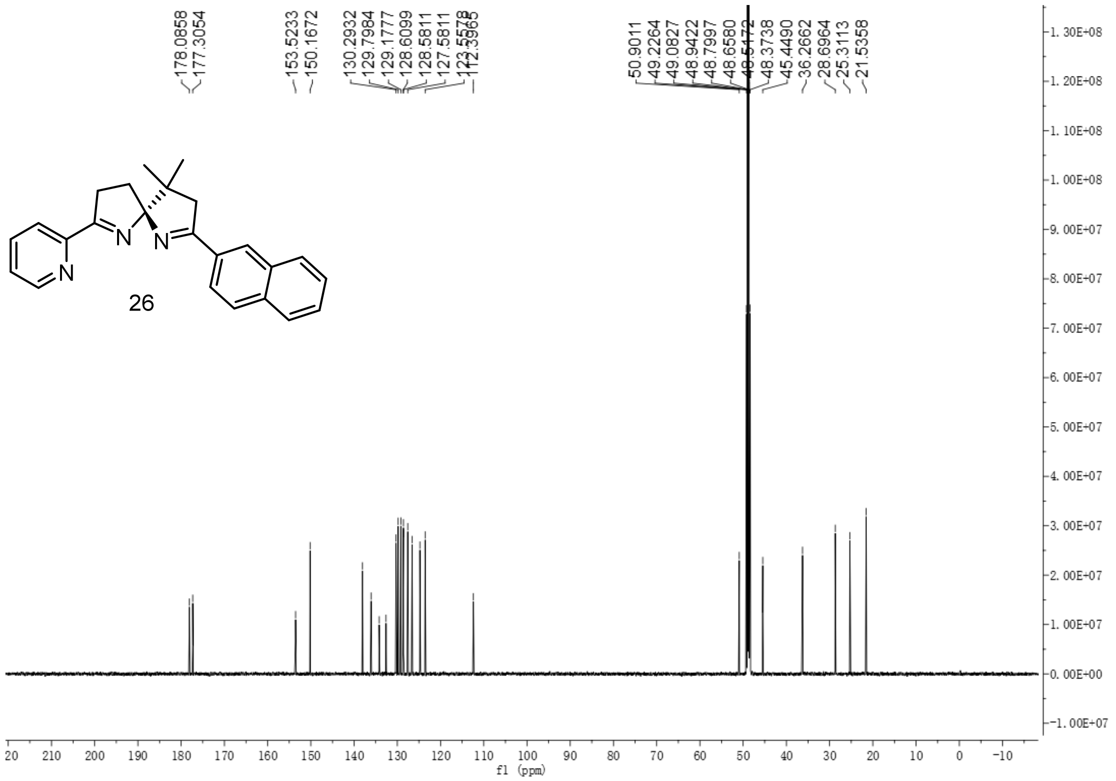

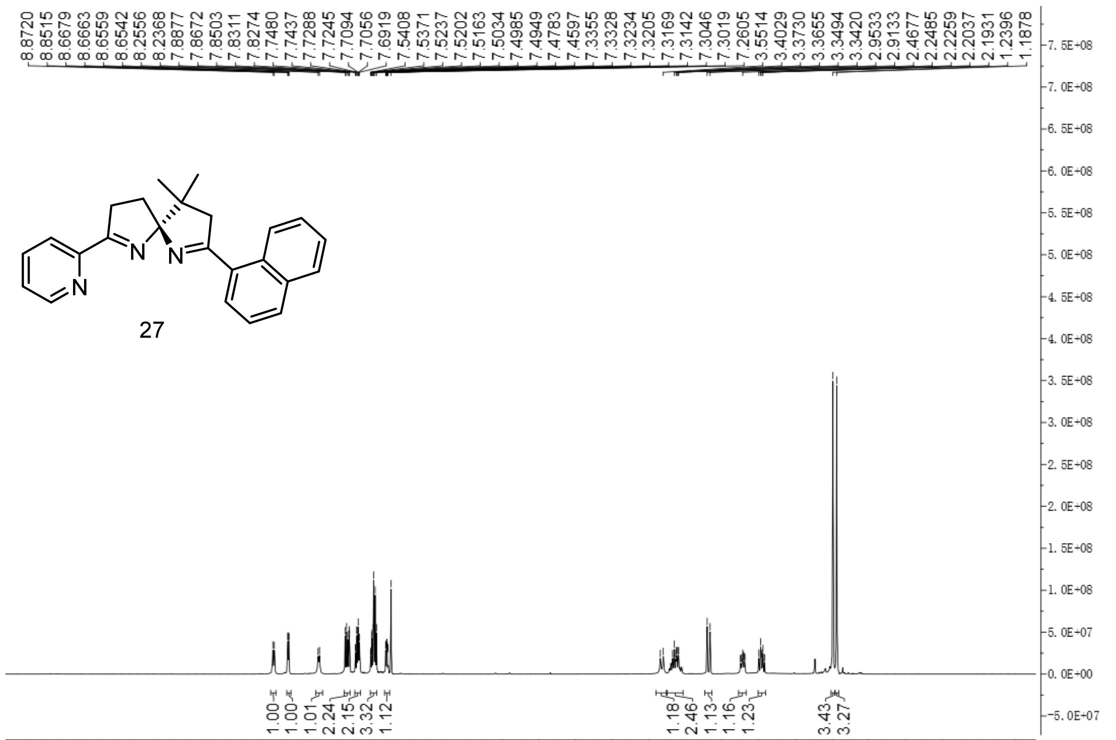

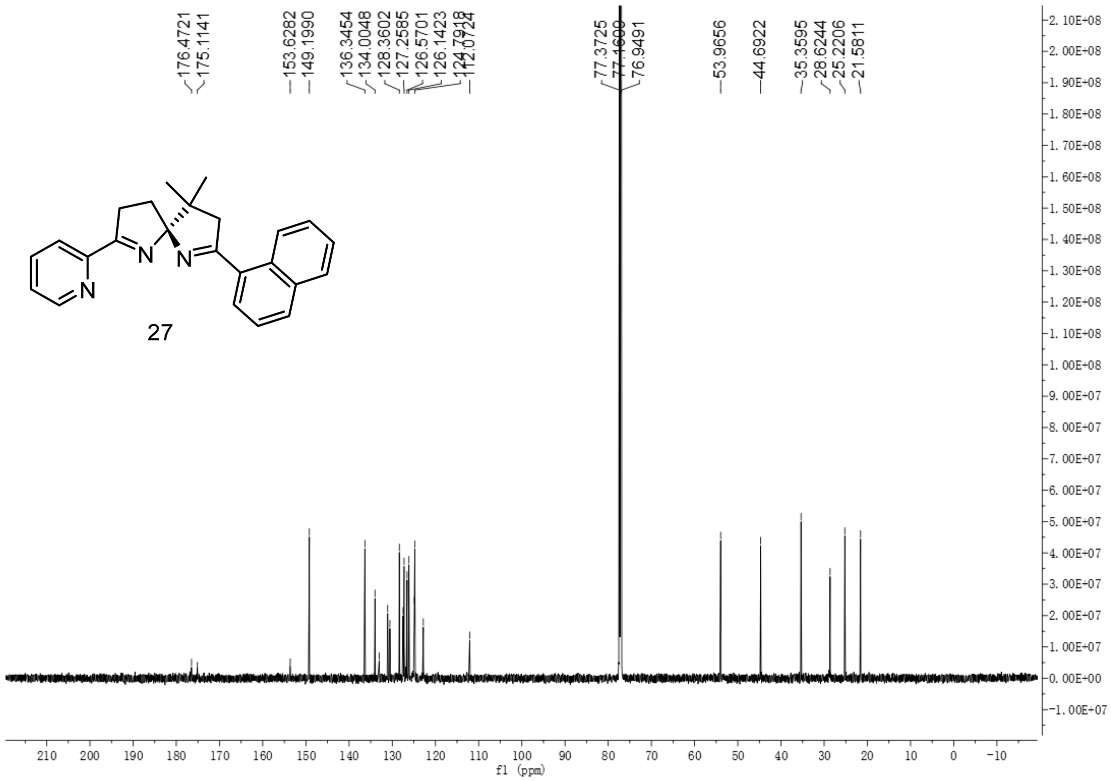
176.4721
175.1141
153.6282
149.1990
136.3454
134.0048
128.3602
127.2585
126.5701
126.1423
112.0724
77.3725
76.9491
53.9656
44.6922
35.3595
28.6244
25.2206
21.5811
N
N
N
27
fl (ppm)

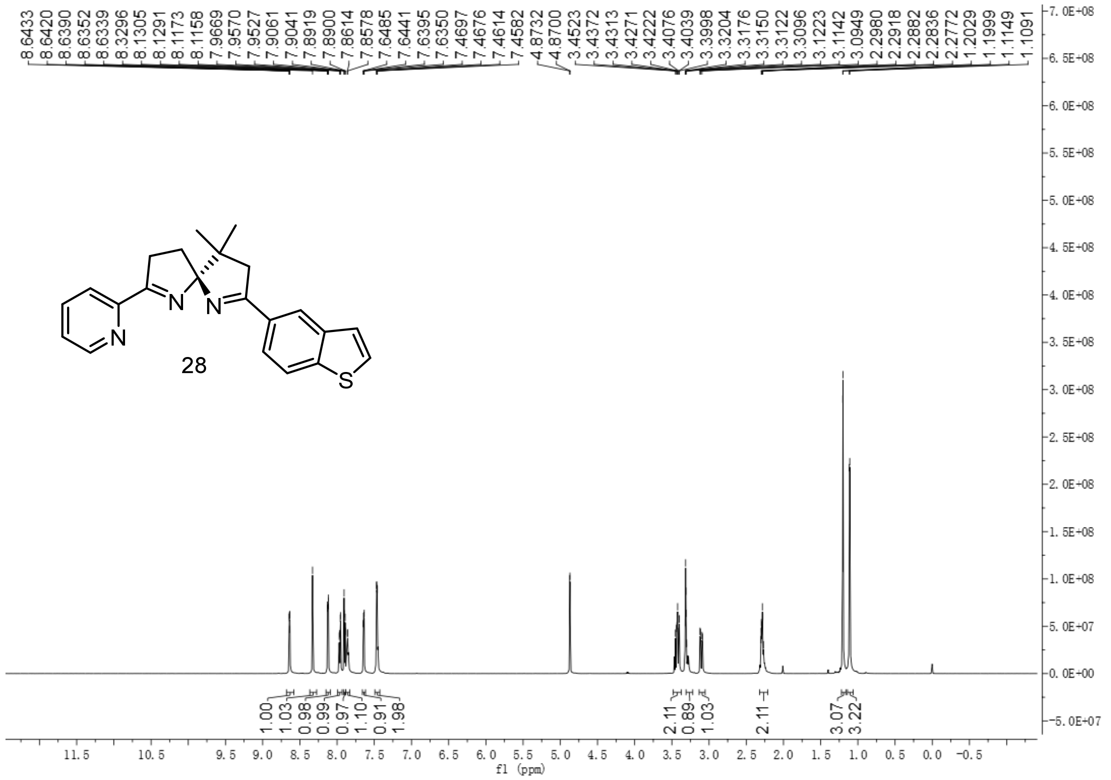
8.6433
8.6420
8.6390
8.6352
8.6339
8.3296
8.1305
8.1291
8.1173
8.1158
7.9669
7.9570
7.9527
7.9061
7.9041
7.8919
7.8900
7.8614
7.8578
7.6485
7.6441
7.6395
7.6350
7.4697
7.4676
7.4614
7.4582
4.8732
4.8700
3.4523
3.4372
3.4313
3.4271
3.4222
3.4076
3.4039
3.3998
3.3204
3.3176
3.3150
3.3122
3.3096
3.1223
3.1142
3.0949
2.2980
2.2918
2.2882
2.2836
2.2772
1.2029
1.1999
1.1149
1.1091
N
N
N
S
28
1.00
1.03
0.98
0.99
0.97
1.10
0.91
1.98
2.11
0.89
1.03
2.11
3.07
3.22
fl (ppm)

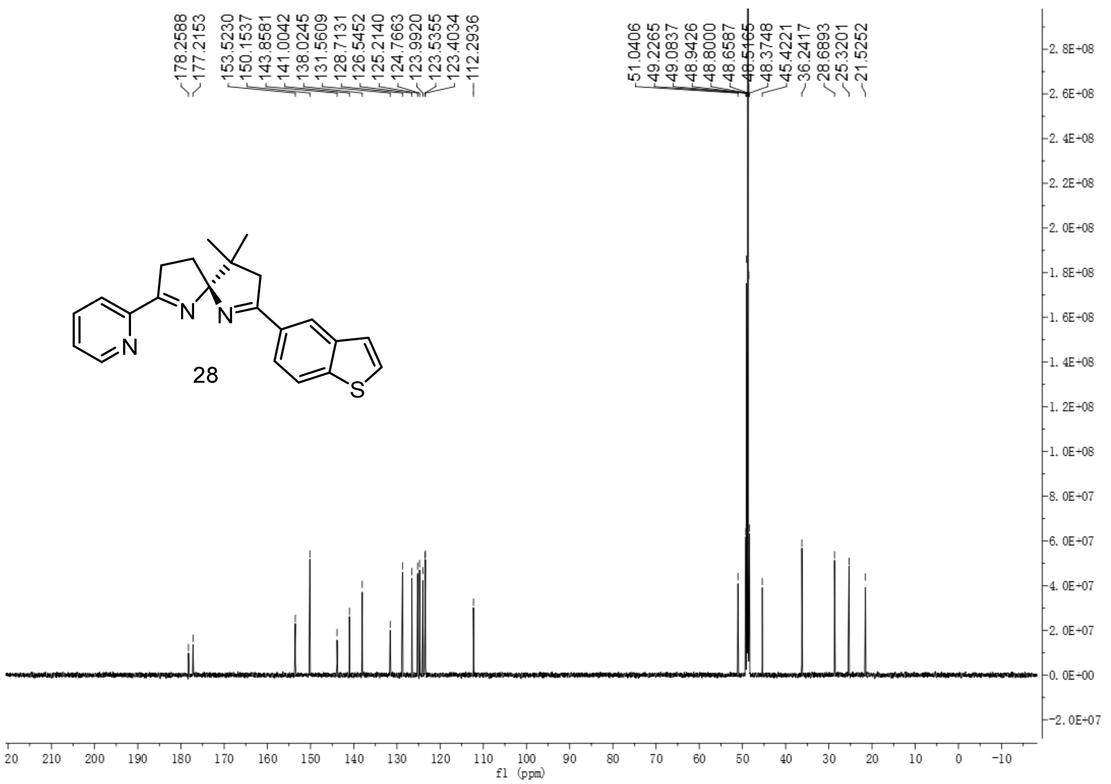
178.2588
177.2153
153.5230
150.1537
143.8581
141.0042
138.0245
131.5609
128.7131
126.5452
125.2140
124.7663
123.9920
123.5355
123.4034
112.2936
51.0406
49.2265
49.0837
48.9426
48.8000
48.6587
48.5165
48.3748
45.4221
36.2417
28.6893
25.3201
21.5252
28
fl (ppm)

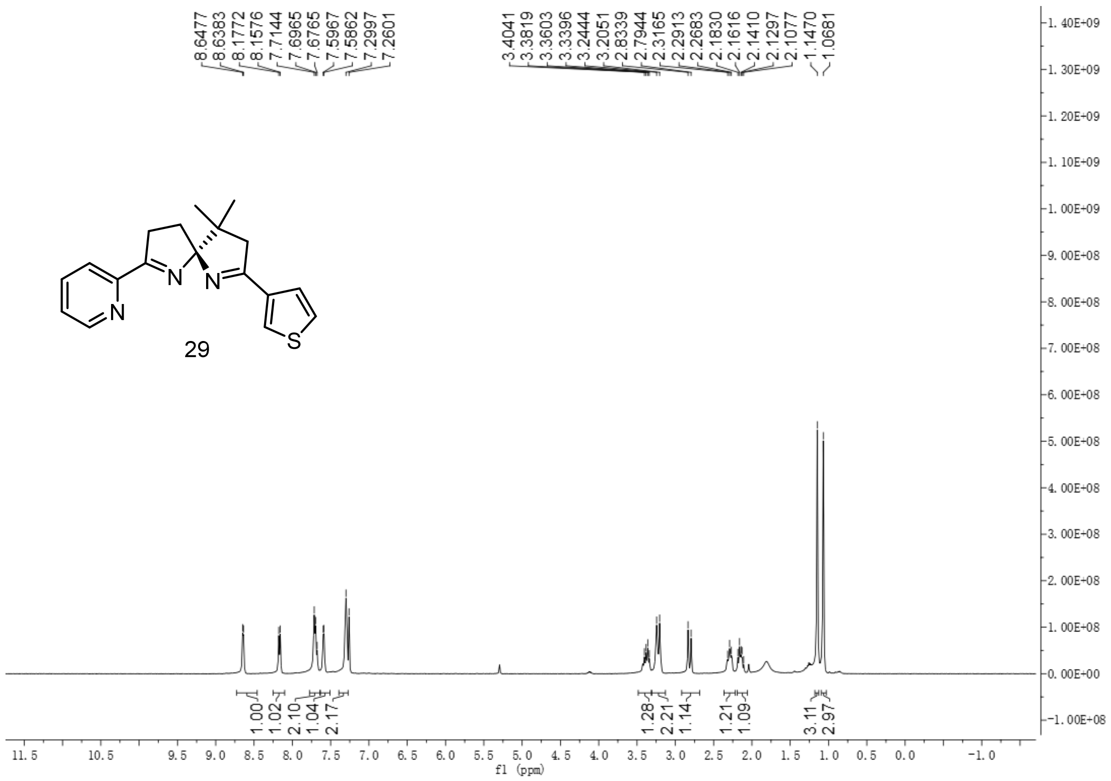
8.6477
8.6383
8.1772
8.1576
7.7144
7.6965
7.6765
7.5967
7.5862
7.2997
7.2601
3.4041
3.3819
3.3603
3.3396
3.2444
3.2051
2.8339
2.7944
2.3165
2.2913
2.2683
2.1830
2.1616
2.1410
2.1297
2.1077
1.1470
1.0681
29
1.00
1.02
2.10
1.04
2.17
1.28
2.21
1.14
1.21
1.09
3.11
2.97
fl (ppm)

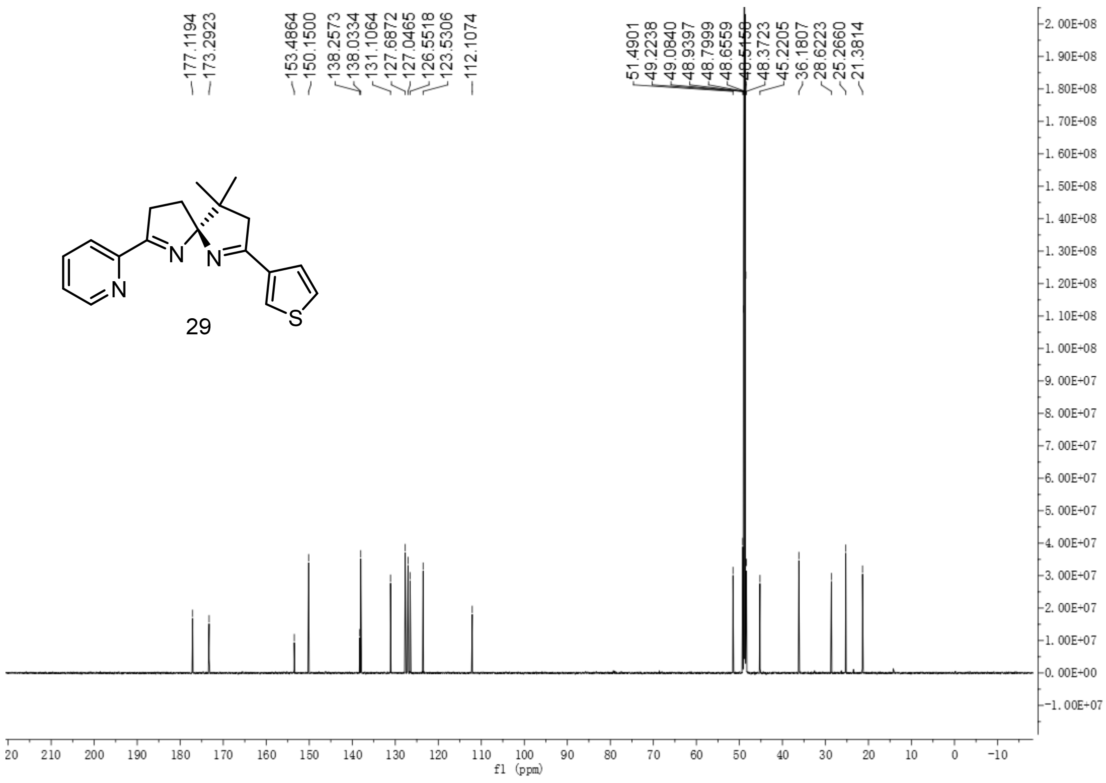
177.1194
173.2923
153.4864
150.1500
138.2573
138.0334
131.1064
127.6872
127.0465
126.5518
123.5306
112.1074
51.4901
49.2238
49.0840
48.9397
48.7999
48.6559
48.5158
48.3723
45.2205
36.1807
28.6223
25.2660
21.3814
N
N
N
S
29
f1 (ppm)

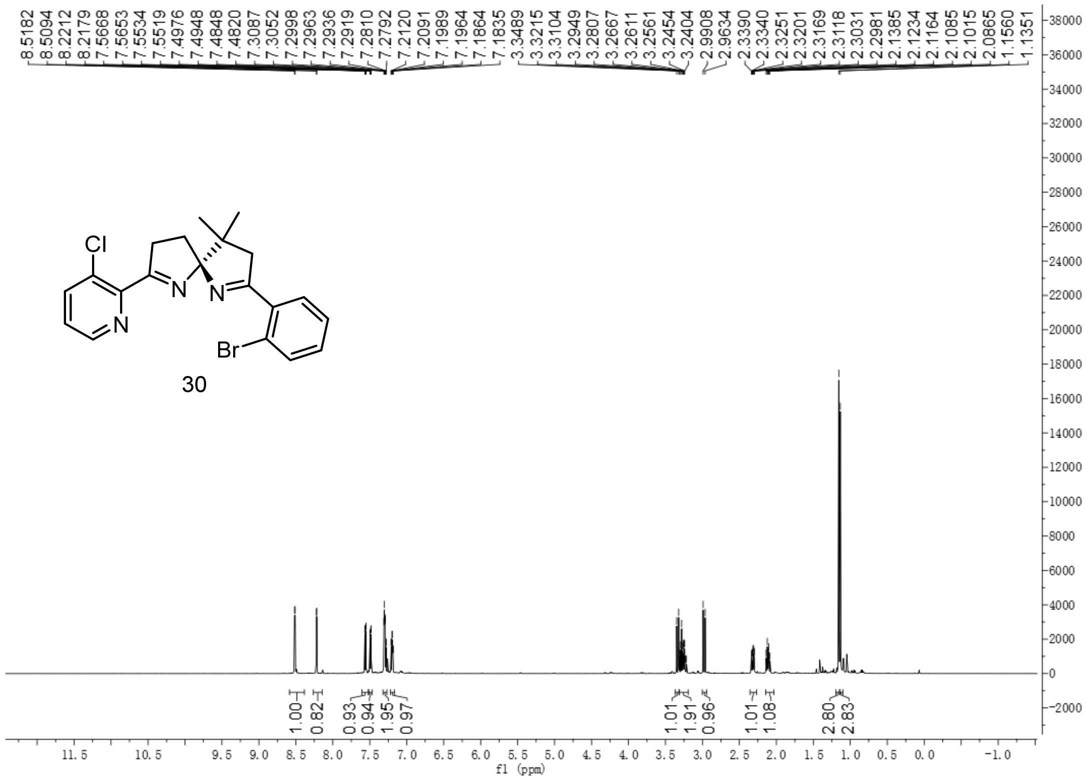
8.5182
8.5094
8.2179
8.2212
7.5668
7.5653
7.5534
7.5519
7.4976
7.4948
7.4848
7.4820
7.3087
7.3052
7.2998
7.2963
7.2936
7.2919
7.2810
7.2792
7.2120
7.2091
7.1989
7.1964
7.1864
7.1835
3.3489
3.3215
3.3104
3.2949
3.2807
3.2667
3.2611
3.2561
3.2454
3.2404
2.9908
2.9634
2.3390
2.3340
2.3251
2.3201
2.3169
2.3118
2.3031
2.2981
2.1385
2.1234
2.1164
2.1085
2.1015
2.0865
1.1560
1.1351
Cl
N
N
N
Br
30
1.00
0.82
0.93
0.94
1.95
0.97
1.01
1.91
0.96
1.01
1.08
2.80
2.83
f1 (ppm)

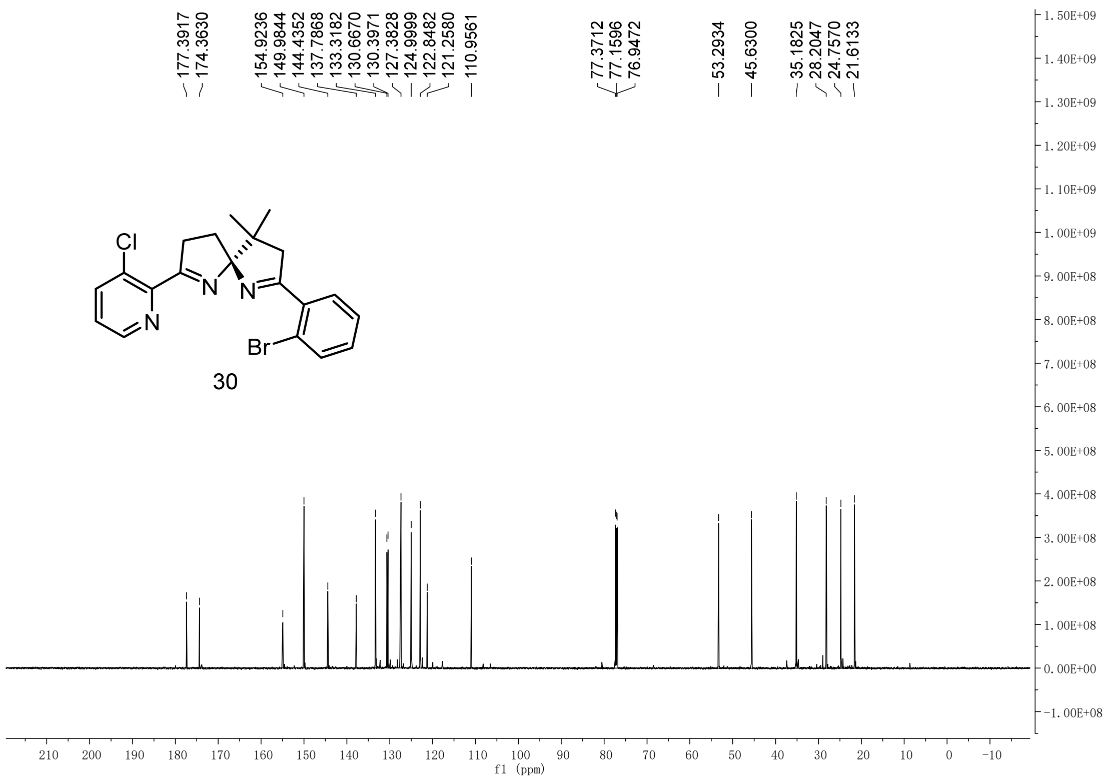
177.3917
174.3630
154.9236
149.9844
144.4352
137.7868
133.3182
130.6670
130.3971
127.3828
124.9999
122.8482
121.2580
110.9561
77.3712
77.1596
76.9472
53.2934
45.6300
35.1825
28.2047
24.7570
21.6133
Cl
N
N
N
Br
30
fl (ppm)

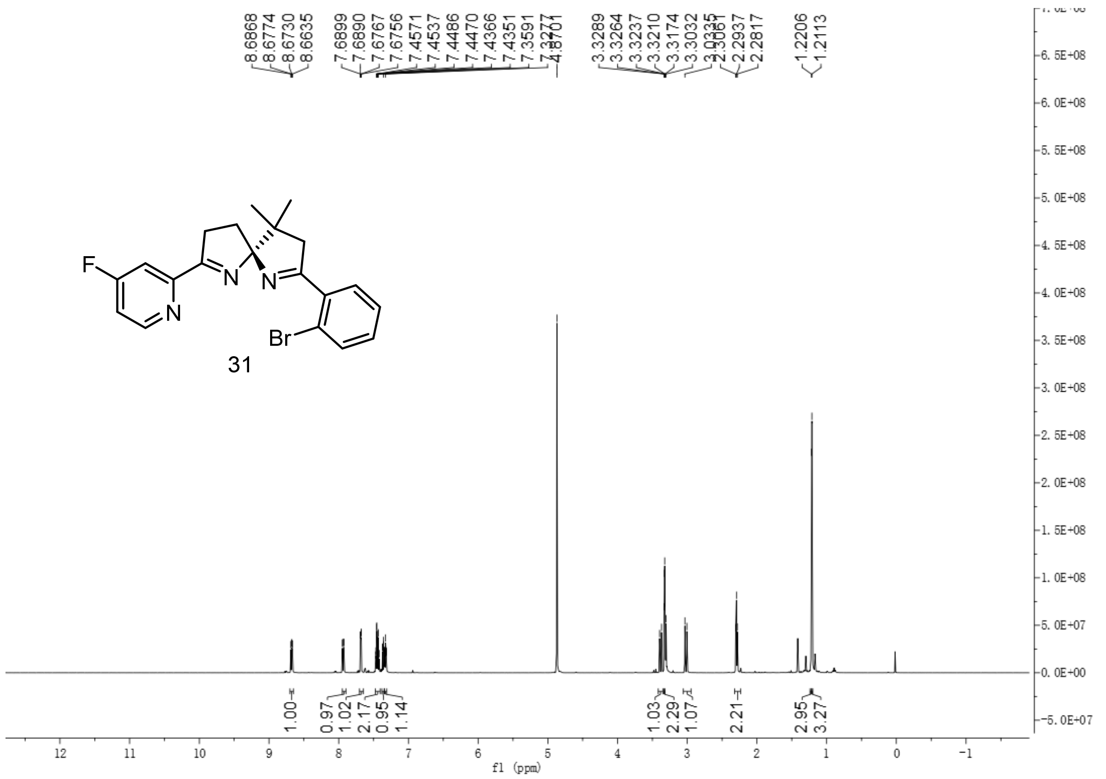
8.6868
8.6774
8.6730
8.6635
7.6899
7.6890
7.6767
7.6756
7.4571
7.4537
7.4486
7.4470
7.4366
7.4351
7.3591
3.3289
3.3264
3.3237
3.3210
3.3174
3.3032
2.2937
2.2817
1.2206
1.2113
F
N
N
N
Br
31
1.00
0.97
1.02
2.17
0.95
1.14
1.03
2.29
1.07
2.21
2.95
3.27
fl (ppm)

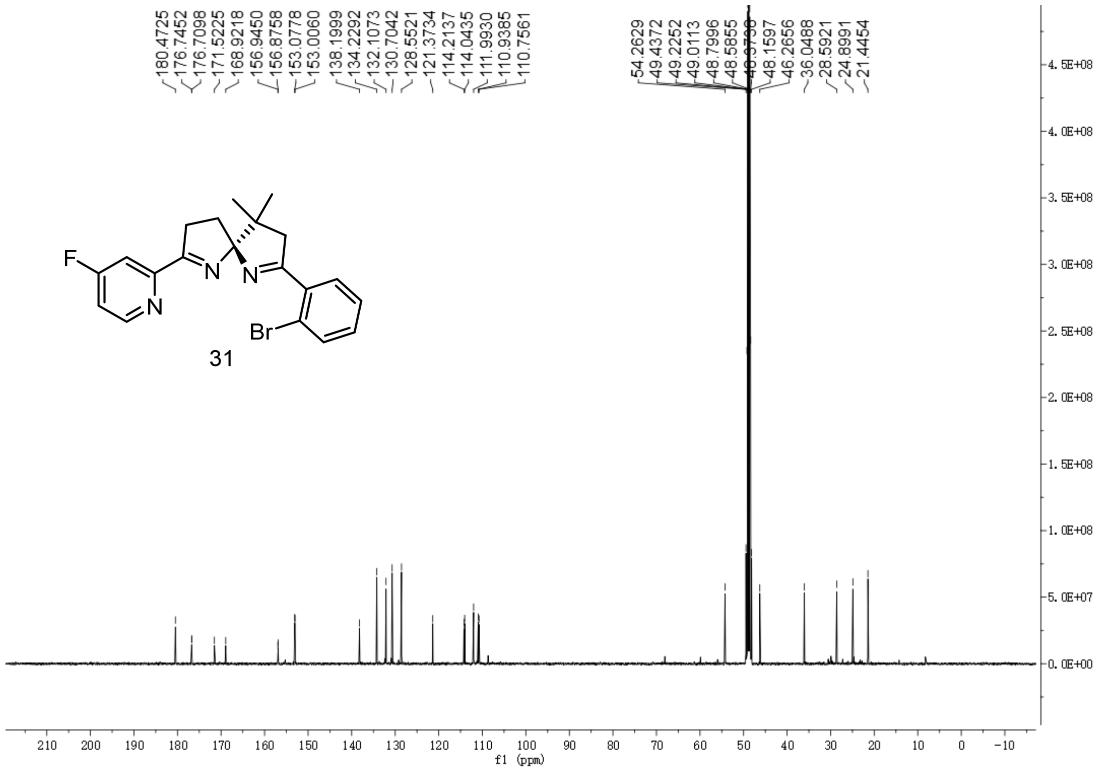

180.4725
176.7452
176.7098
171.5225
168.9218
156.9450
156.8758
153.0778
153.0060
138.1999
134.2292
132.1073
130.7042
128.5521
121.3734
114.2137
114.0435
111.9930
110.9385
110.7561
54.2629
49.4372
49.2252
49.0113
48.7996
48.5855
48.1597
46.2656
36.0488
28.5921
24.8991
21.4454
F
N
N
N
Br
31
f1 (ppm)

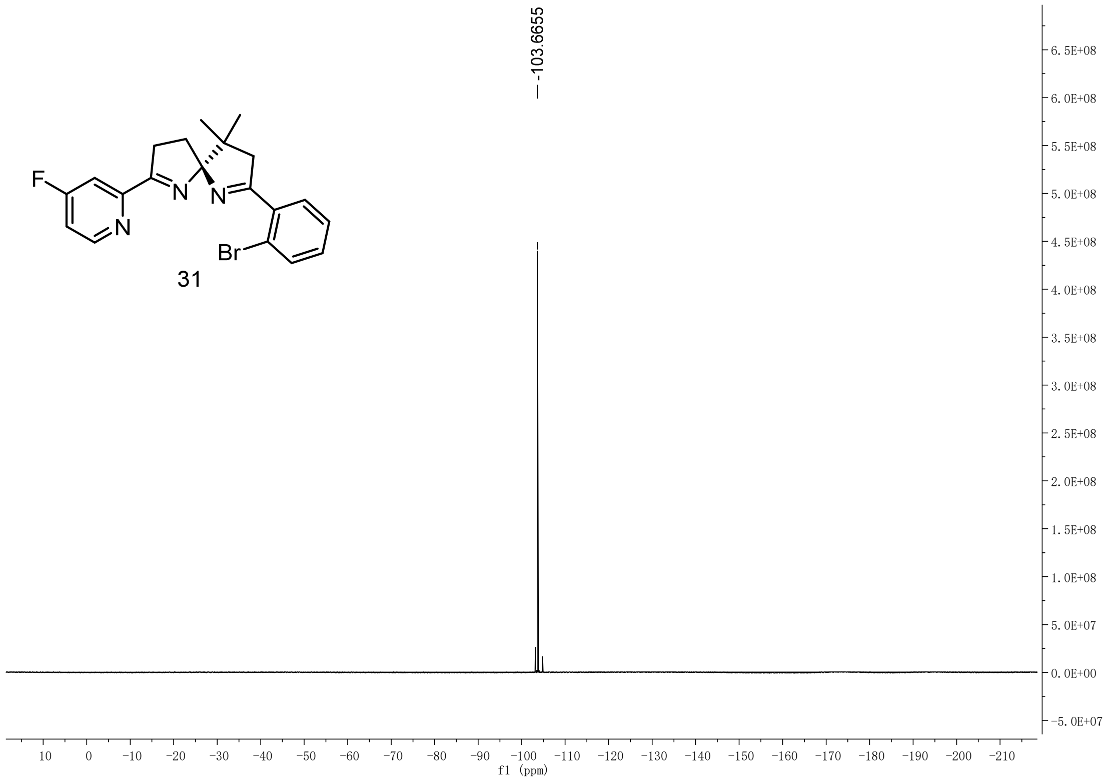

-103.6655
F
N
N
N
Br
31
f1 (ppm)

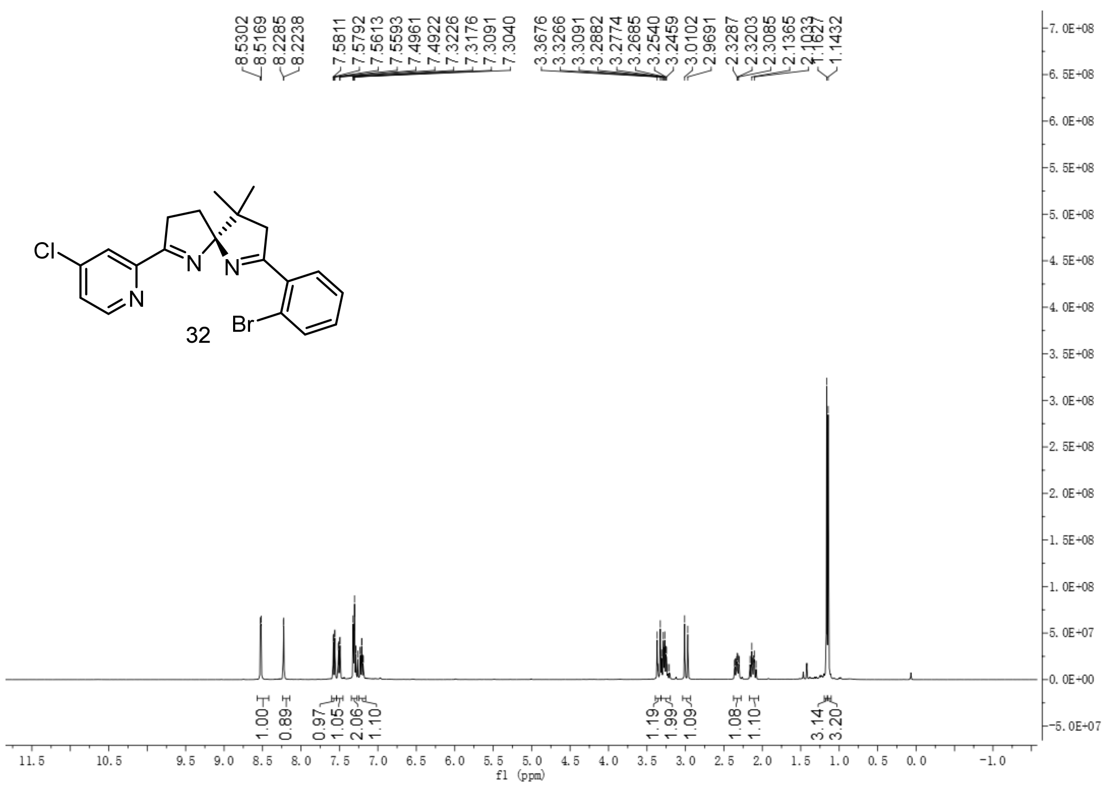
8.5302
8.5169
8.2285
8.2238
7.5811
7.5792
7.5613
7.5593
7.4961
7.4922
7.3226
7.3176
7.3091
7.3040
3.3676
3.3266
3.3091
3.2882
3.2774
3.2685
3.2540
3.2459
3.0102
2.9691
2.3287
2.3203
2.3085
2.1365
2.1033
1.1627
1.1432
Cl
N
N
N
Br
32
1.00
0.89
0.97
1.05
2.06
1.10
1.19
1.99
1.09
1.08
1.10
3.14
3.20
fl (ppm)

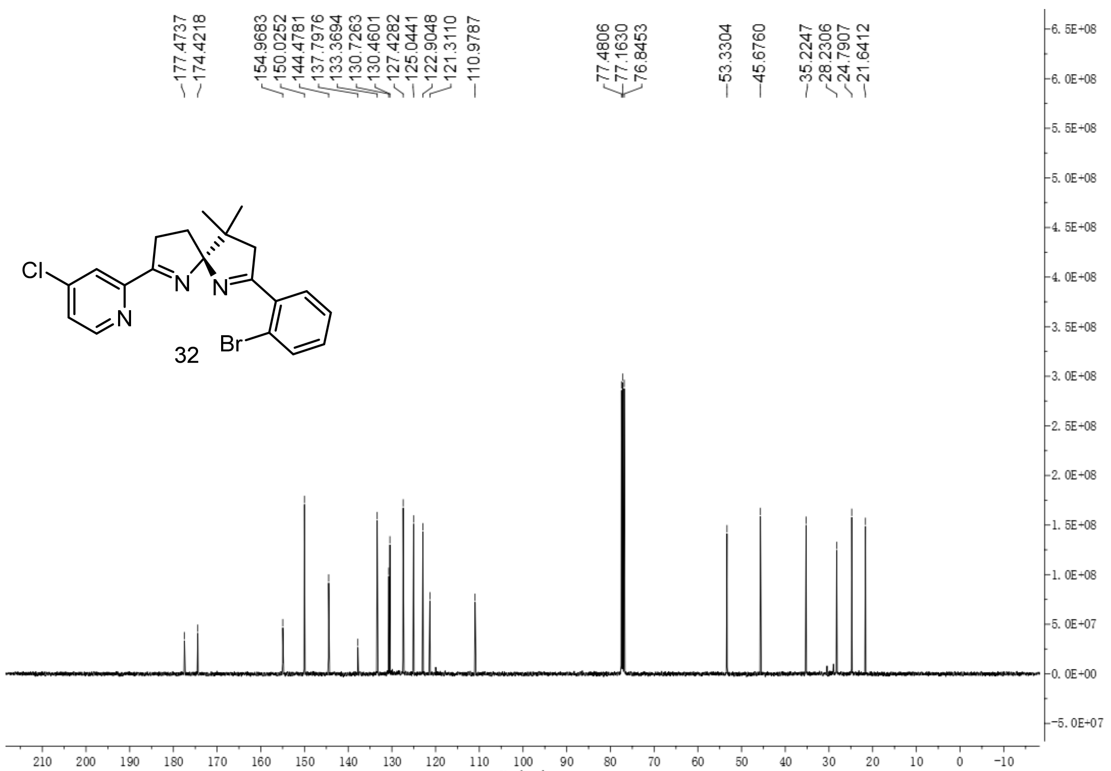
177.4737
174.4218
154.9683
150.0252
144.4781
137.7976
133.3694
130.7263
130.4601
127.4282
125.0441
122.9048
121.3110
110.9787
77.4806
77.1630
76.8453
53.3304
45.6760
35.2247
28.2306
24.7907
21.6412
Cl
N
N
N
Br
32
fl (ppm)

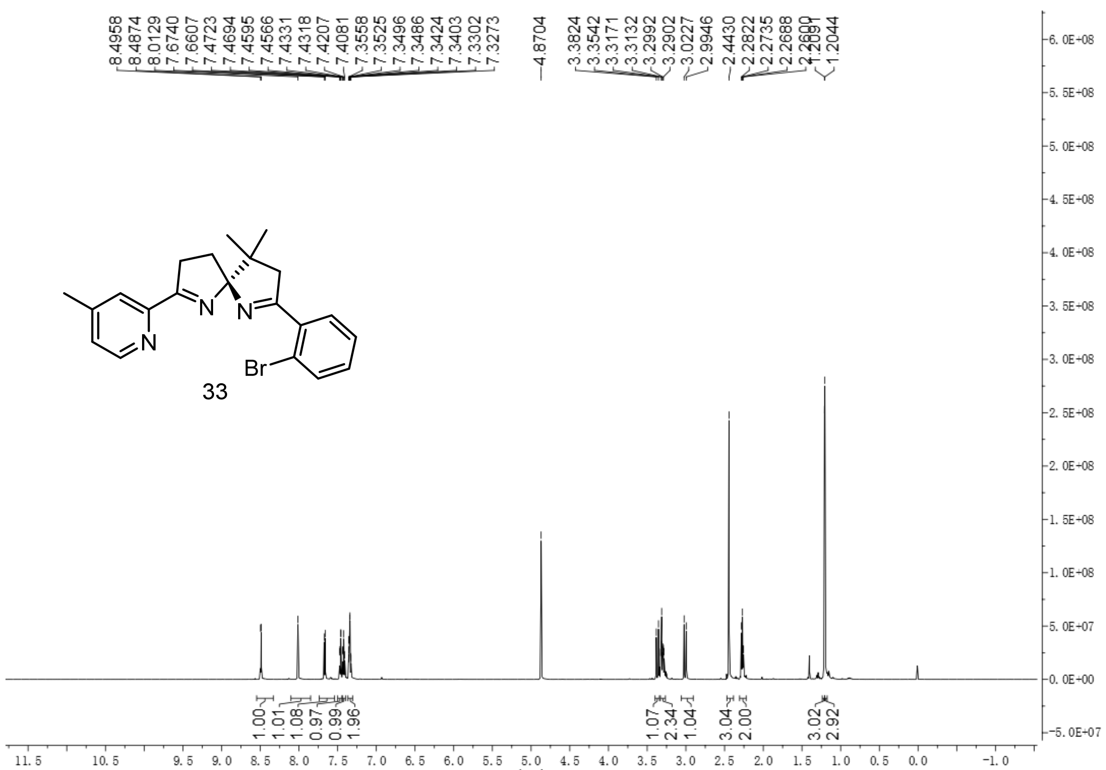

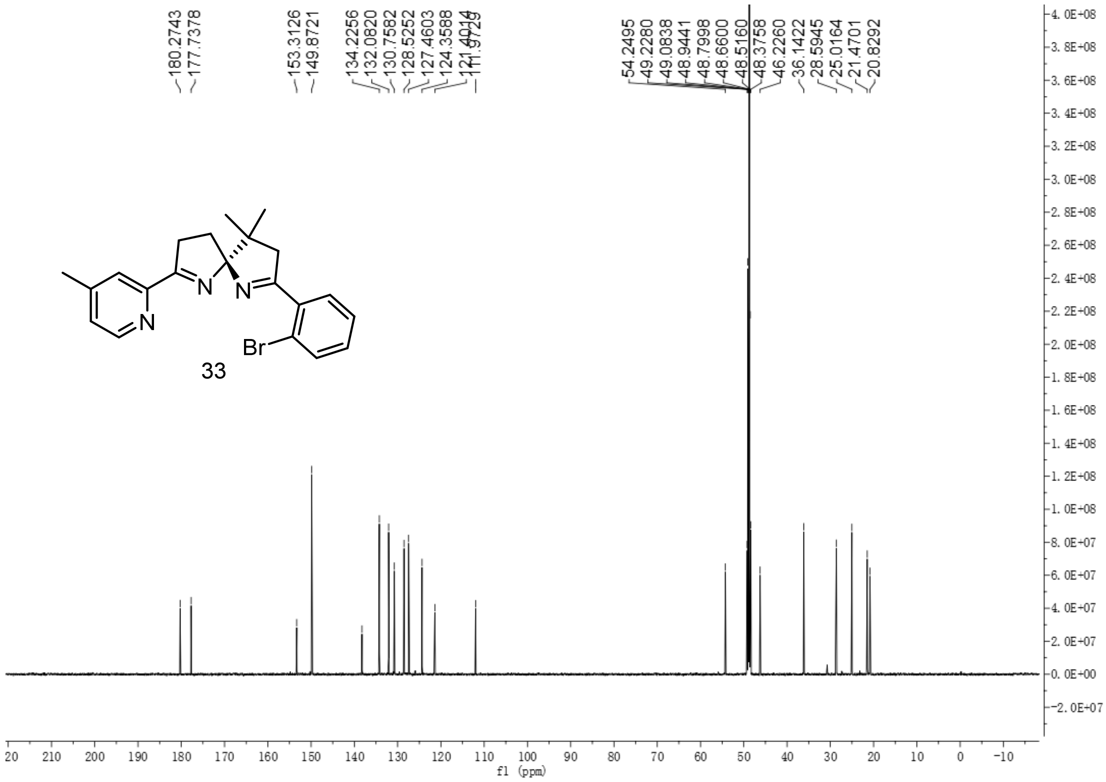

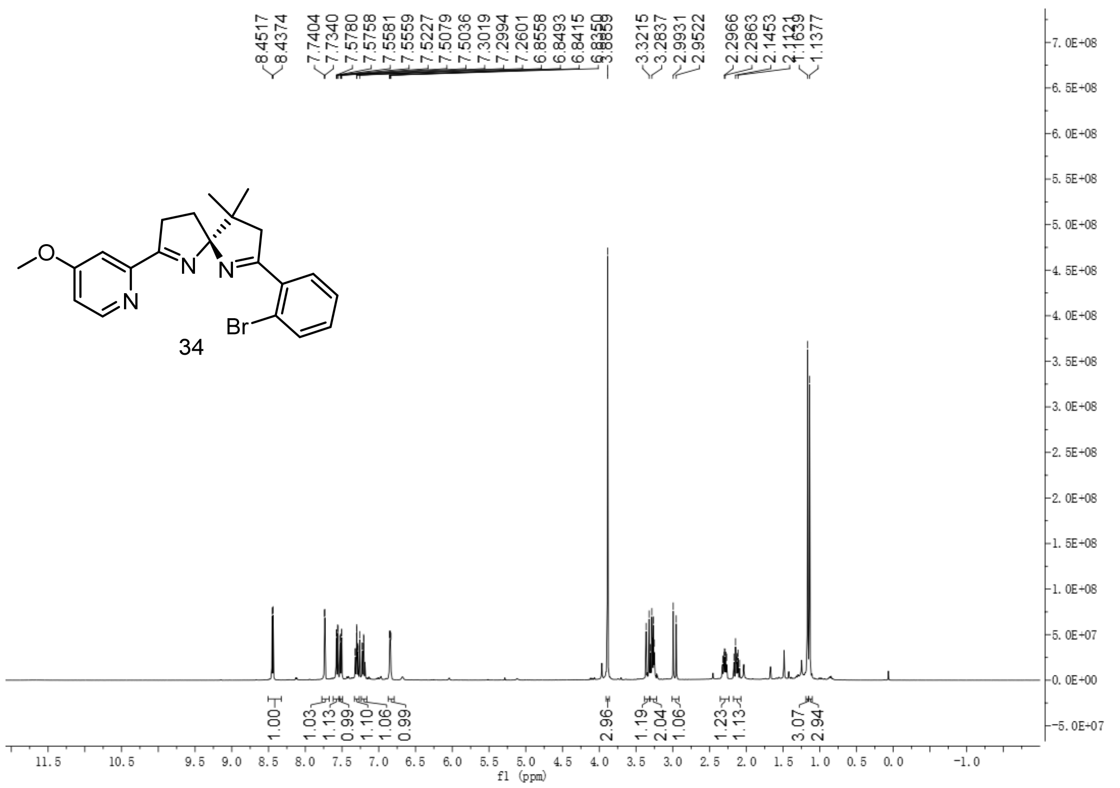
8.4517
8.4374
7.7404
7.7340
7.5780
7.5758
7.5581
7.5559
7.5227
7.5079
7.5036
7.3019
7.2994
7.2601
6.8558
6.8493
6.8415
3.3215
3.2837
2.9931
2.9522
2.2966
2.2863
2.1453
1.1377
O
N
N
N
Br
34
1.00
1.03
1.13
0.99
1.10
1.06
0.99
2.96
1.19
2.04
1.06
1.23
1.13
3.07
2.94
fl (ppm)

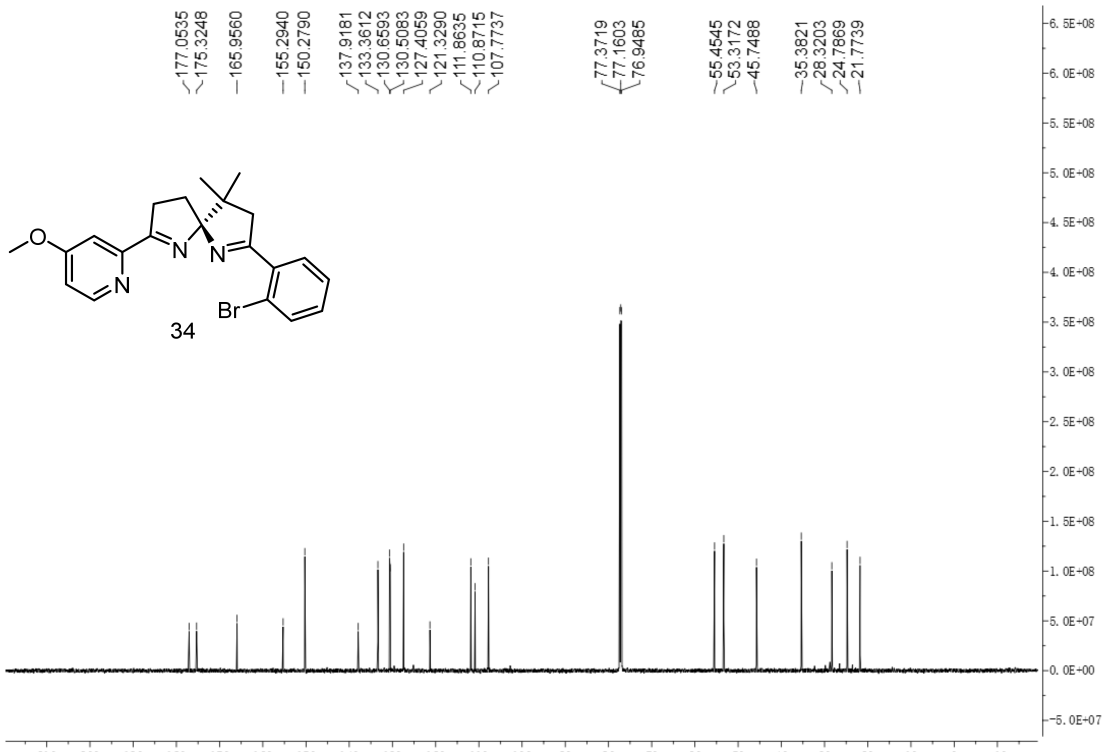
177.0535
175.3248
165.9560
155.2940
150.2790
137.9181
133.3612
130.6593
130.5083
127.4059
121.3290
111.8635
110.8715
107.7737
77.3719
77.1603
76.9485
55.4545
53.3172
45.7488
35.3821
28.3203
24.7869
21.7739
O
N
N
N
Br
34
fl (ppm)

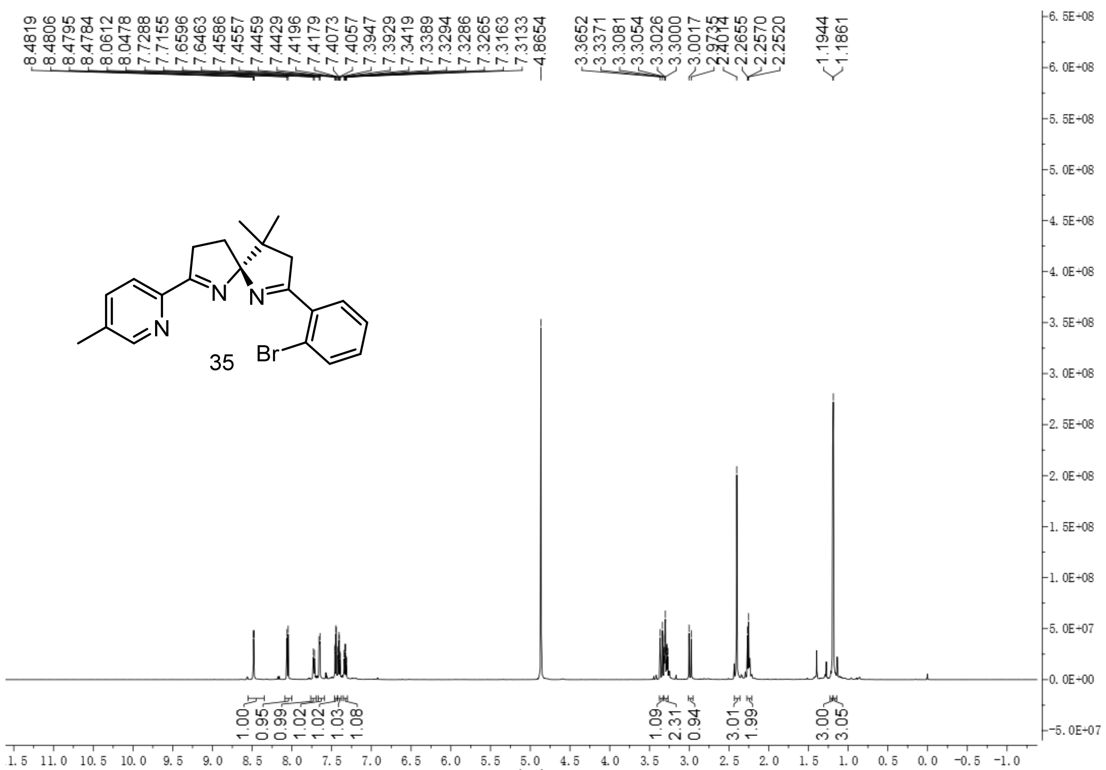
8.4819
8.4806
8.4795
8.4784
8.0612
8.0478
7.7288
7.7155
7.6596
7.6463
7.4586
7.4557
7.4459
7.4429
7.4196
7.4179
7.4073
7.4057
7.3947
7.3929
7.3419
7.3389
7.3294
7.3286
7.3265
7.3163
7.3133
4.8654
3.3652
3.3371
3.3081
3.3054
3.3026
3.3000
3.0017
2.2655
2.2570
2.2520
1.1944
1.1861
N
N
N
35
Br
1.00
0.95
0.99
1.02
1.02
1.03
1.08
1.09
2.31
0.94
3.01
1.99
3.00
3.05
f1 (ppm)

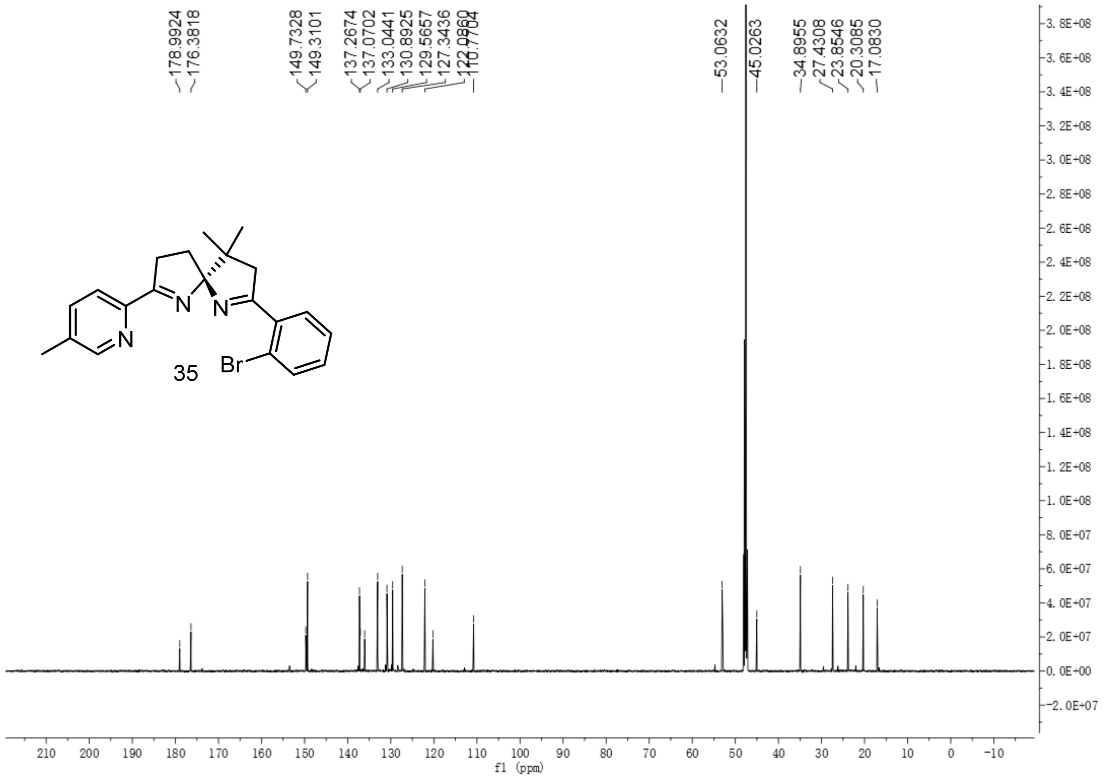
178.9924
176.3818
149.7328
149.3101
137.2674
137.0702
133.0441
130.8925
129.5657
127.3436
53.0632
45.0263
34.8955
27.4308
23.8546
20.3085
17.0830
N
N
N
35
Br
f1 (ppm)

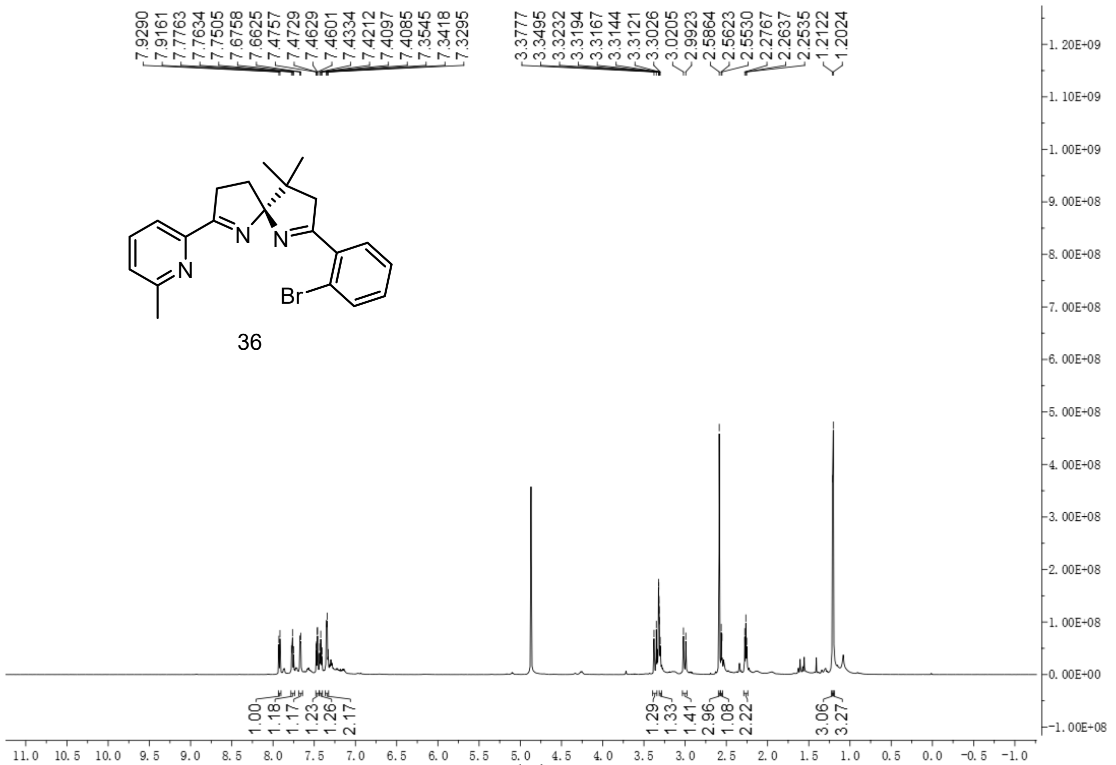
7.9290
7.9161
7.7763
7.7634
7.7505
7.6758
7.6625
7.4757
7.4729
7.4629
7.4601
7.4334
7.4212
7.4097
7.4085
7.3545
7.3418
7.3295
3.3777
3.3495
3.3232
3.3194
3.3167
3.3144
3.3121
3.3026
3.0205
2.9923
2.5864
2.5623
2.5530
2.2767
2.2637
2.2535
1.2122
1.2024
Br
N
N
N
36
1.00
1.18
1.17
1.23
1.26
2.17
1.29
1.33
1.41
2.96
1.08
2.22
3.06
3.27
f1 (ppm)

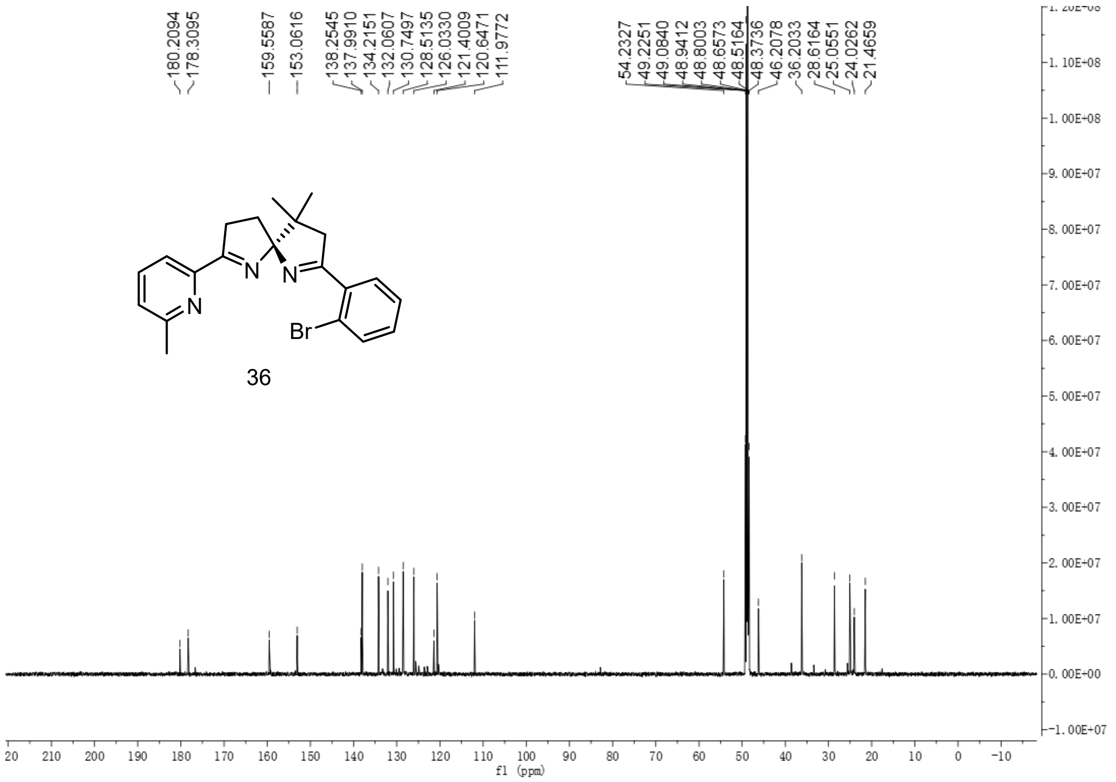
180.2094
178.3095
159.5587
153.0616
138.2545
137.9910
134.2151
132.0607
130.7497
128.5135
126.0330
121.4009
120.6471
111.9772
54.2327
49.2251
49.0840
48.9412
48.8003
48.6573
48.5164
48.3736
46.2078
36.2033
28.6164
25.0551
24.0262
21.4659
Br
N
N
N
36
f1 (ppm)

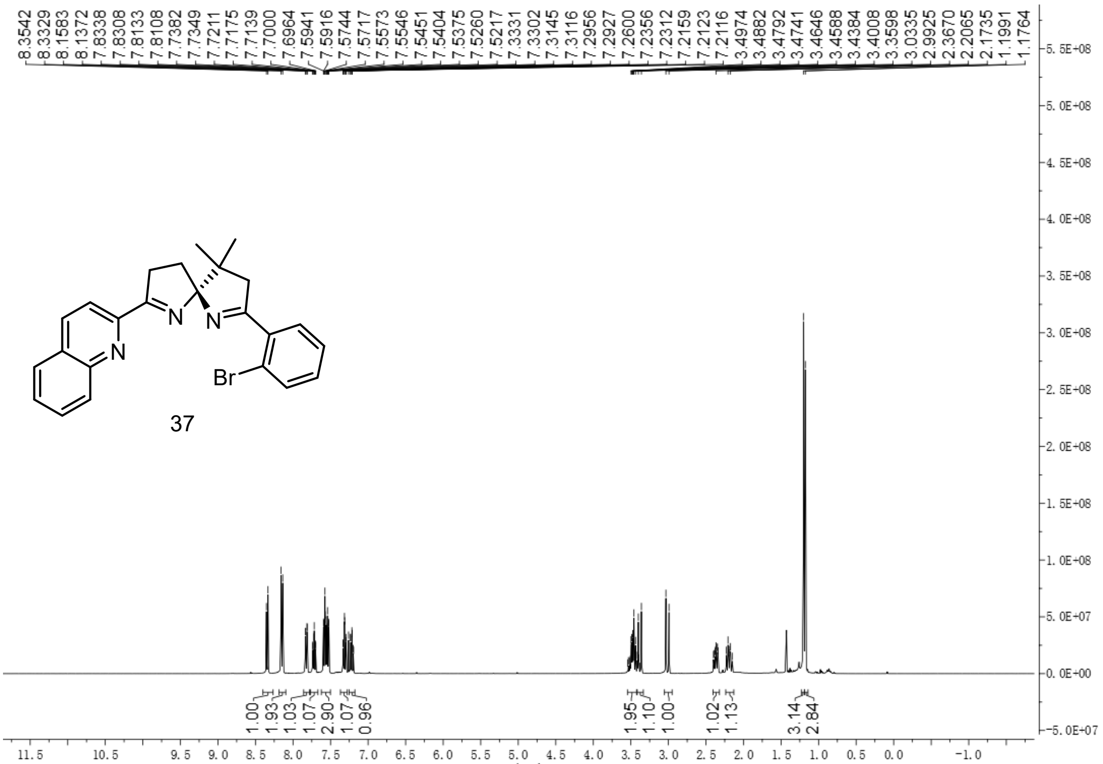
8.3542
8.3329
8.1583
8.1372
7.8338
7.8308
7.8133
7.8108
7.7382
7.7349
7.7211
7.7175
7.7139
7.7000
7.6964
7.5941
7.5916
7.5744
7.5717
7.5573
7.5546
7.5451
7.5404
7.5375
7.5260
7.5217
7.3331
7.3302
7.3145
7.3116
7.2956
7.2927
7.2600
7.2356
7.2312
7.2159
7.2123
7.2116
3.4974
3.4882
3.4792
3.4741
3.4646
3.4588
3.4384
3.4008
3.3598
3.0335
2.9925
2.3670
2.2065
2.1735
1.1991
1.1764
N
N
N
Br
37
1.00
1.93
1.03
1.07
2.90
1.07
0.96
1.95
1.10
1.00
1.02
1.13
3.14
2.84
f1 (ppm)

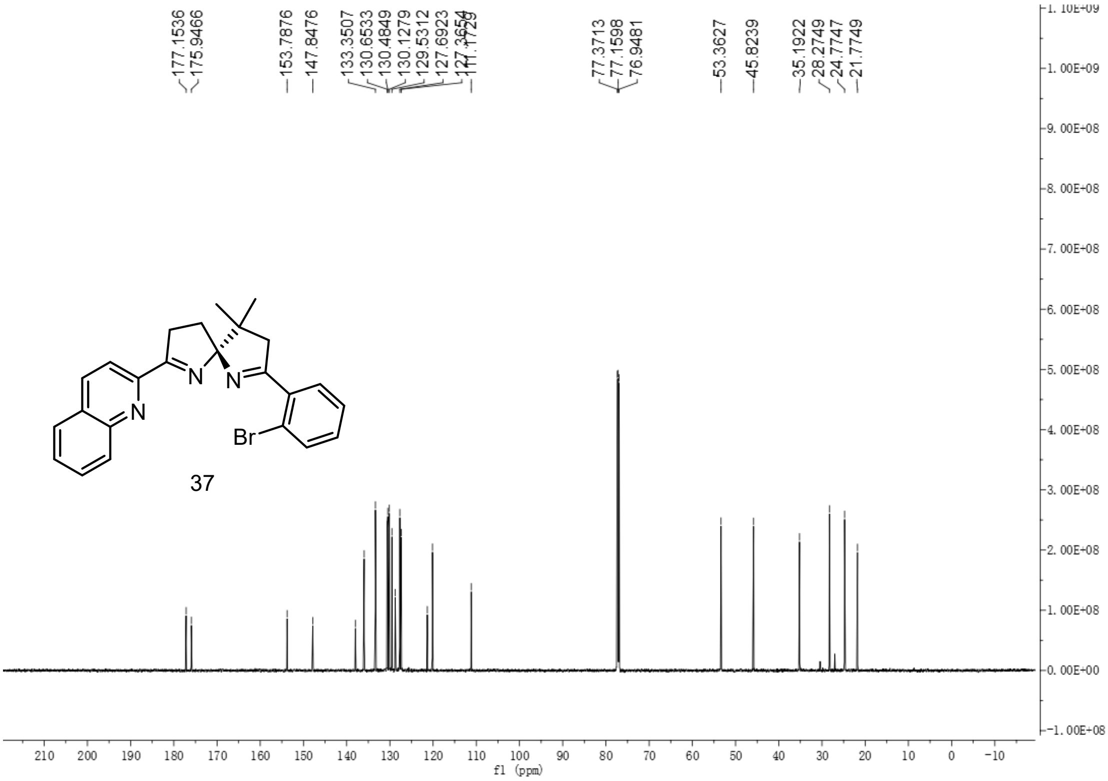
177.1536
175.9466
153.7876
147.8476
133.3507
130.6533
130.4849
130.1279
129.5312
127.6923
127.3654
111.1729
77.3713
77.1598
76.9481
53.3627
45.8239
35.1922
28.2749
24.7747
21.7749
N
N
N
Br
37
f1 (ppm)

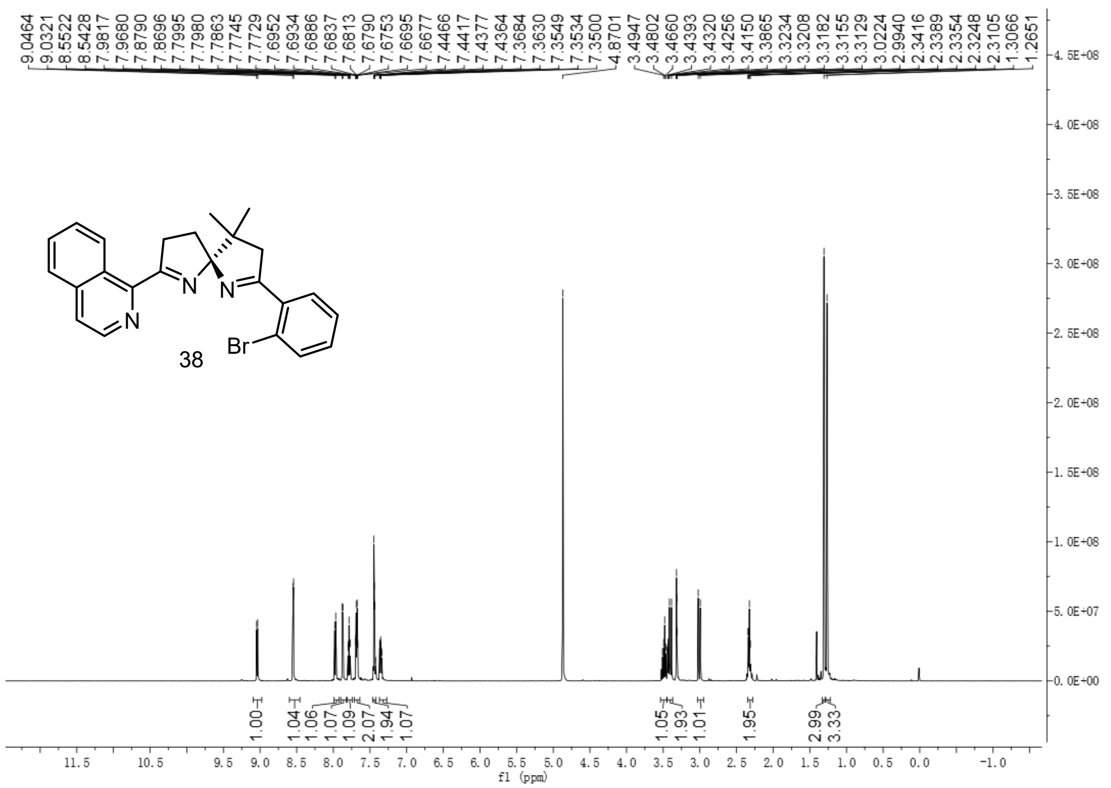

38

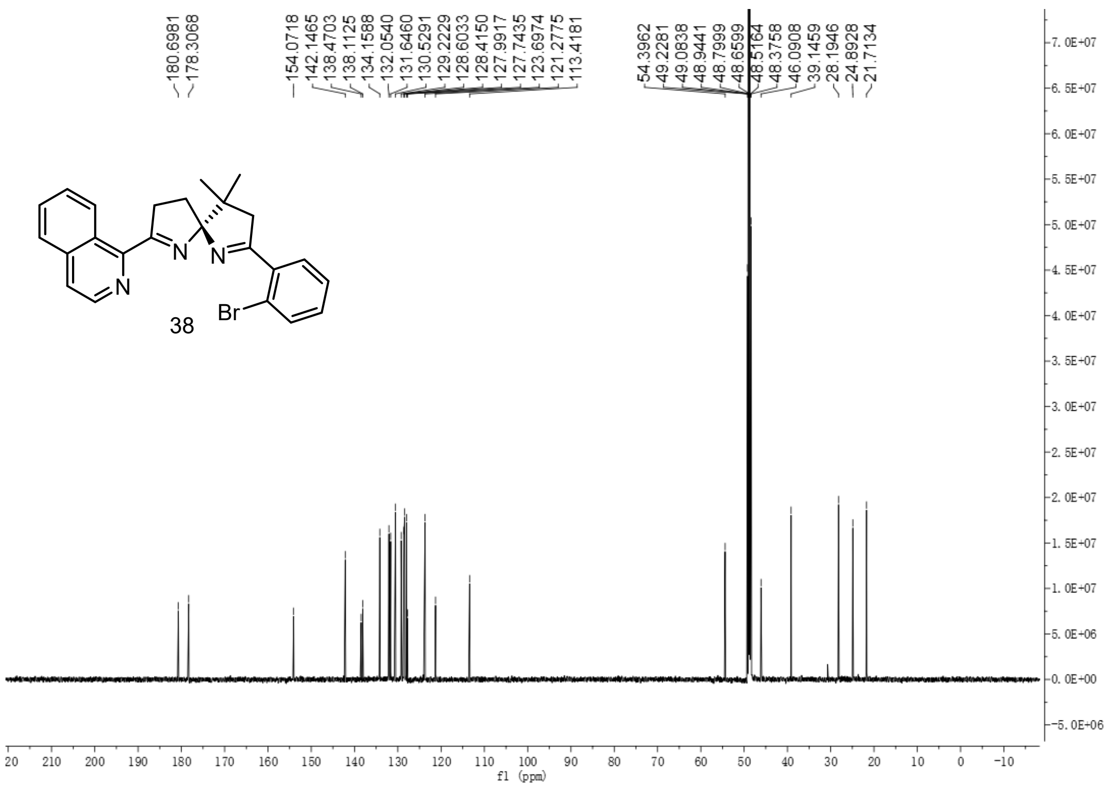

38

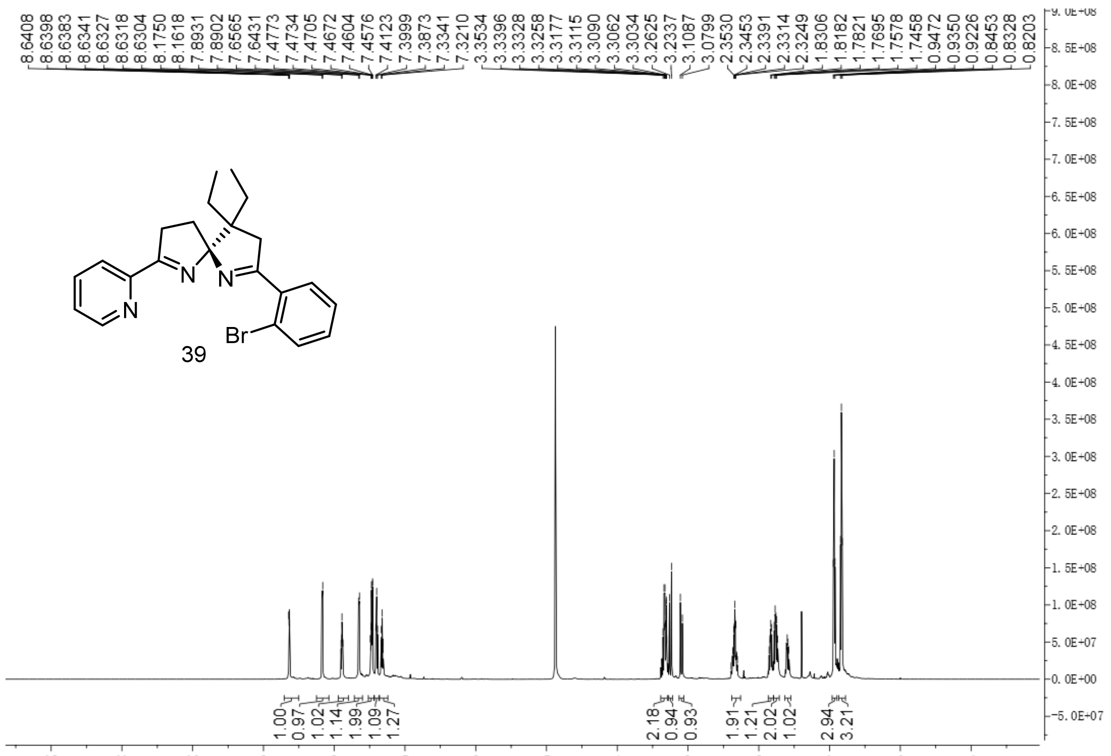

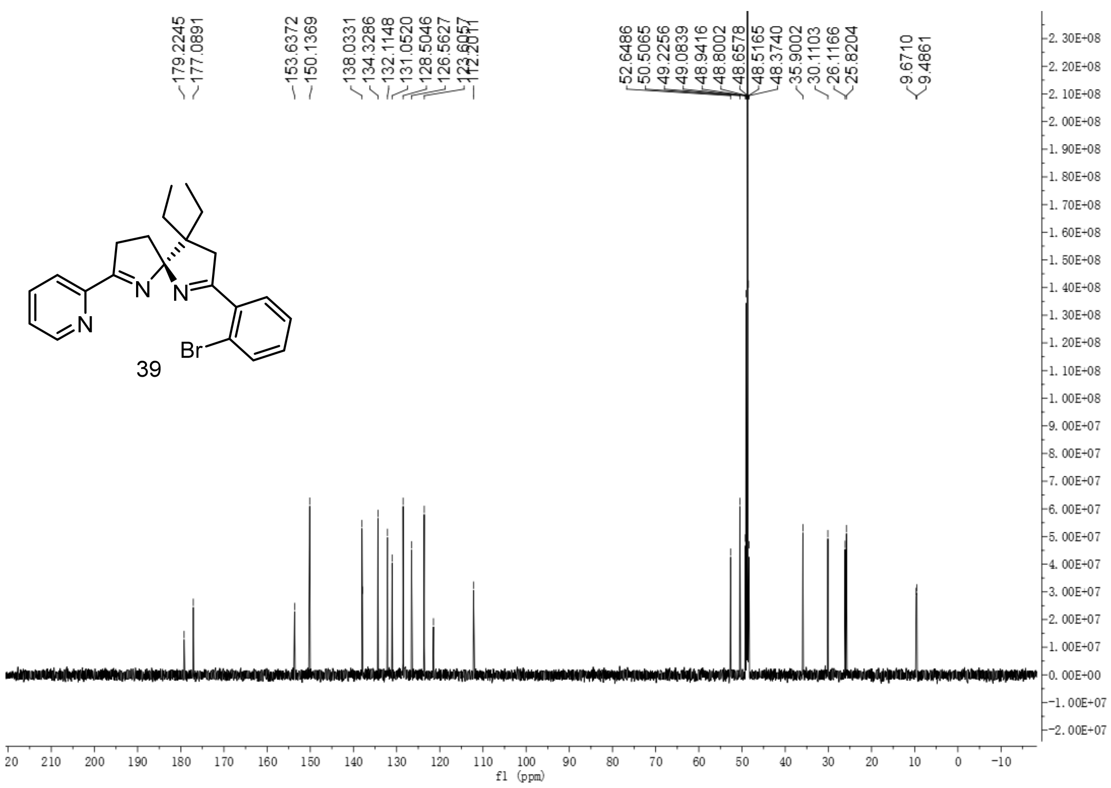

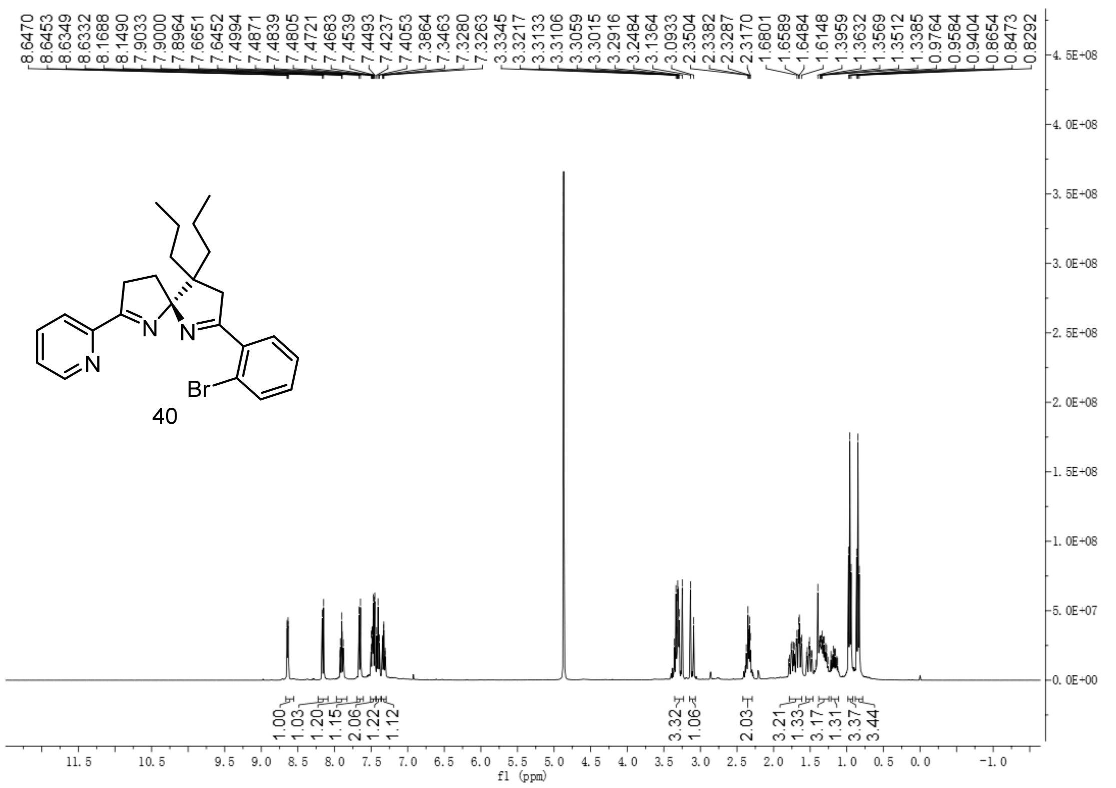
8.6470
8.6453
8.6349
8.6332
8.1688
8.1490
7.9033
7.9000
7.8964
7.6651
7.6452
7.4994
7.4871
7.4839
7.4805
7.4721
7.4683
7.4539
7.4493
7.4237
7.4053
7.3864
7.3463
7.3280
7.3263
3.3345
3.3217
3.3133
3.3106
3.3059
3.3015
3.2916
3.2484
3.1364
3.0933
2.3504
2.3382
2.3287
2.3170
1.6801
1.6589
1.6484
1.6148
1.3959
1.3632
1.3569
1.3512
1.3385
0.9764
0.9584
0.9404
0.8654
0.8473
0.8292
N
N
N
Br
40
1.00
1.03
1.20
1.15
2.06
1.22
1.12
3.32
1.06
2.03
3.21
1.33
3.17
1.31
3.37
3.44
f1 (ppm)

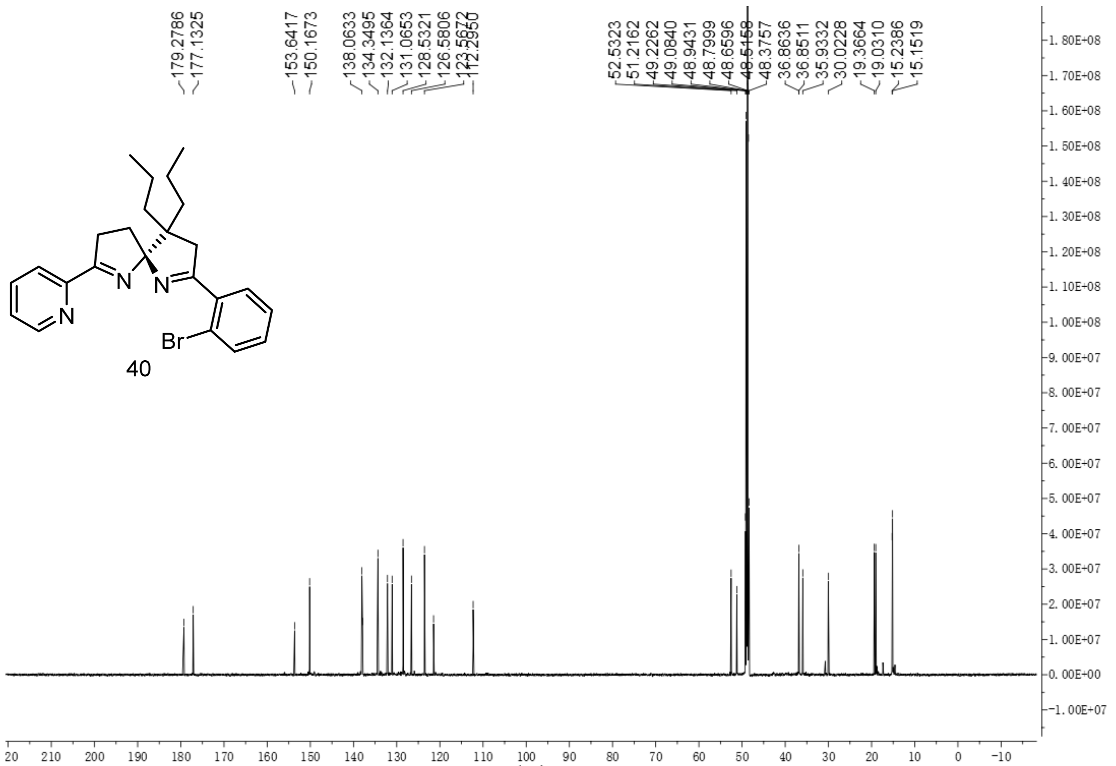
179.2786
177.1325
153.6417
150.1673
138.0633
134.3495
132.1364
131.0653
128.5321
126.5806
123.5672
112.2950
52.5323
51.2162
49.2262
49.0840
48.9431
48.7999
48.6596
48.5158
48.3757
36.8636
36.8511
35.9332
30.0228
19.3664
19.0310
15.2386
15.1519
N
N
N
Br
40
f1 (ppm)

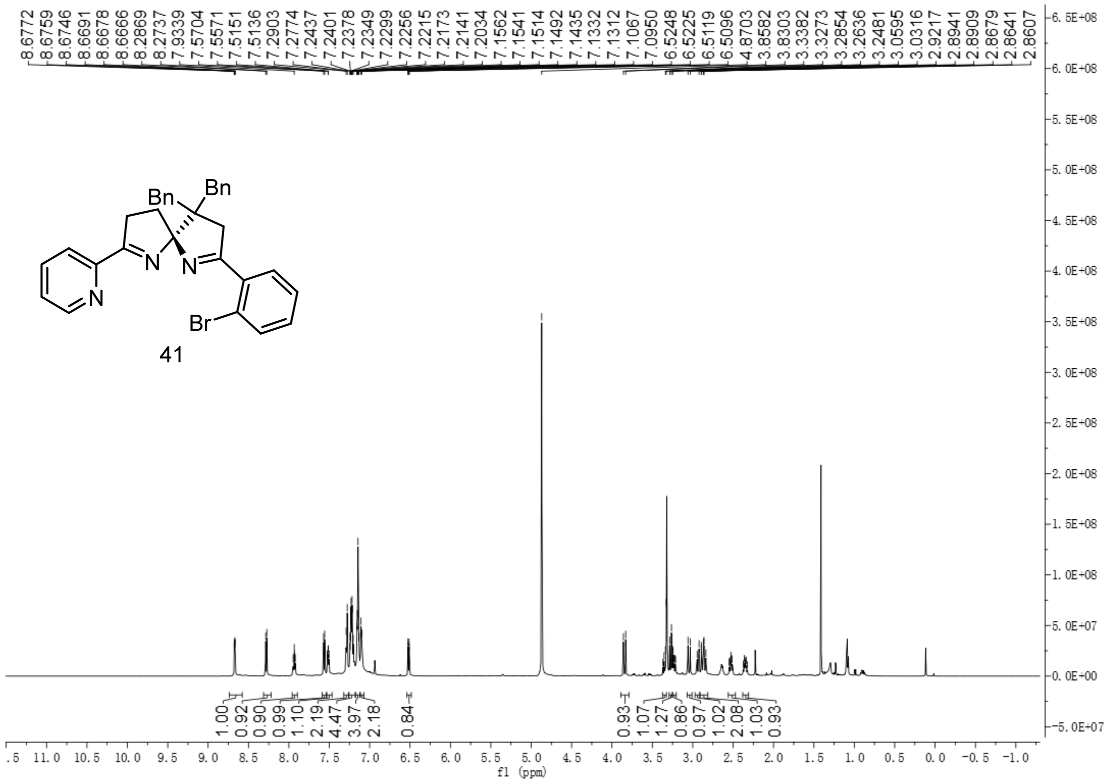
8.6772
8.6759
8.6746
8.6691
8.6678
8.6666
8.2869
8.2737
7.9339
7.5704
7.5571
7.5151
7.5136
7.2903
7.2774
7.2437
7.2401
7.2378
7.2349
7.2299
7.2256
7.2215
7.2173
7.2141
7.2034
7.1562
7.1541
7.1514
7.1492
7.1435
7.1332
7.1312
7.1067
7.0950
6.5248
6.5225
6.5119
6.5096
4.8703
3.8582
3.8303
3.3382
3.3273
3.2854
3.2636
3.2481
3.0595
3.0316
2.9217
2.8941
2.8909
2.8679
2.8641
2.8607
Bn
Bn
N
N
N
Br
41
1.00
0.92
0.90
0.99
1.10
2.19
4.47
3.97
2.18
0.84
0.93
1.07
1.27
0.86
0.97
1.02
2.08
1.03
0.93
f1 (ppm)
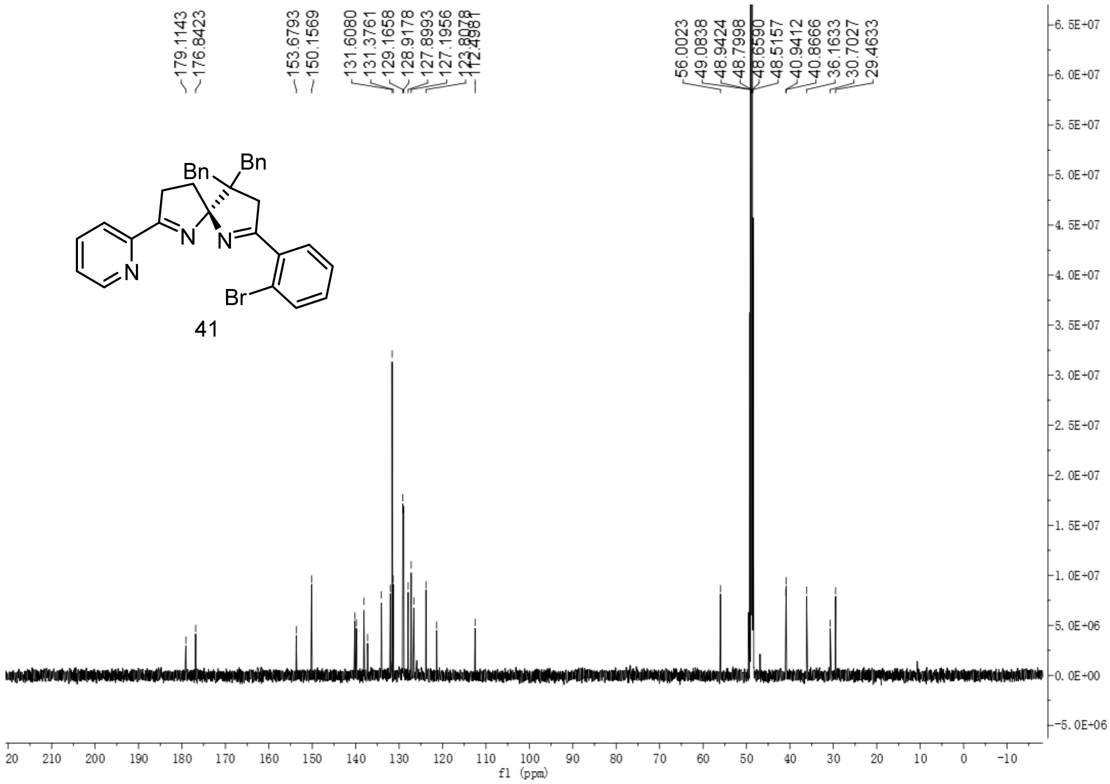
179.1143
176.8423
153.6793
150.1569
131.6080
131.3761
129.1658
128.9178
127.8993
127.1956
123.8078
112.4981
56.0023
49.0838
48.9424
48.7998
48.6590
48.5157
40.9412
40.8666
36.1633
30.7027
29.4633
Bn
Bn
N
N
N
Br
41
f1 (ppm)

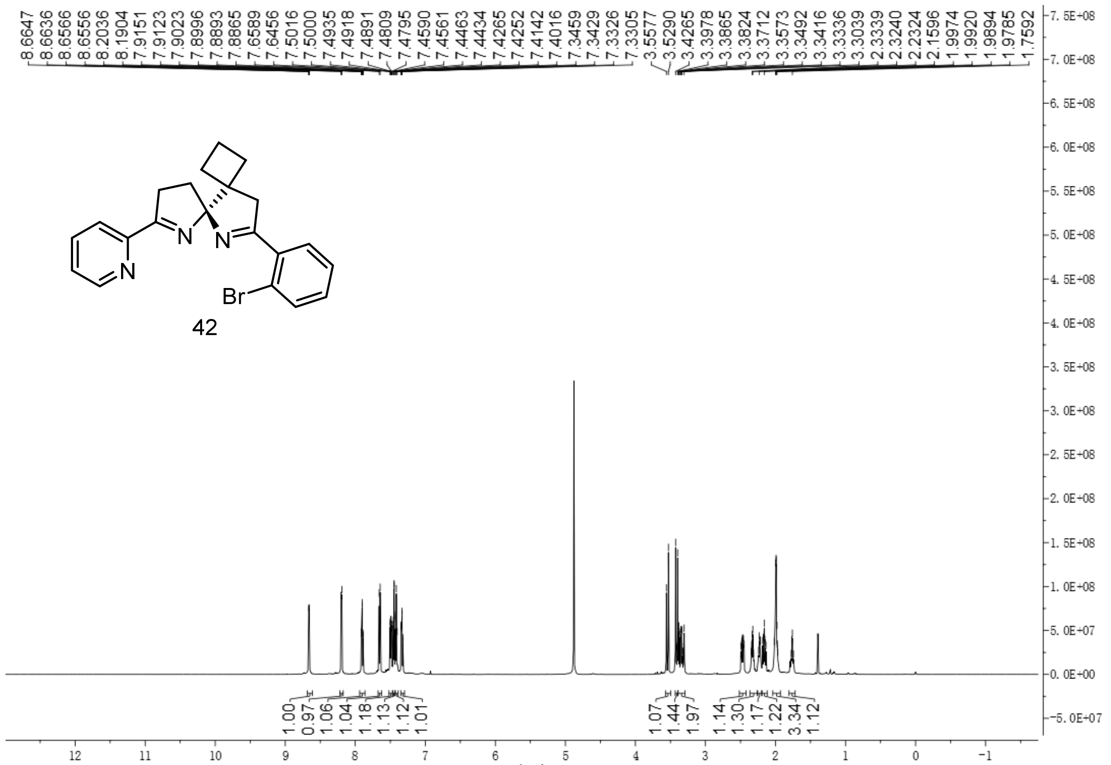

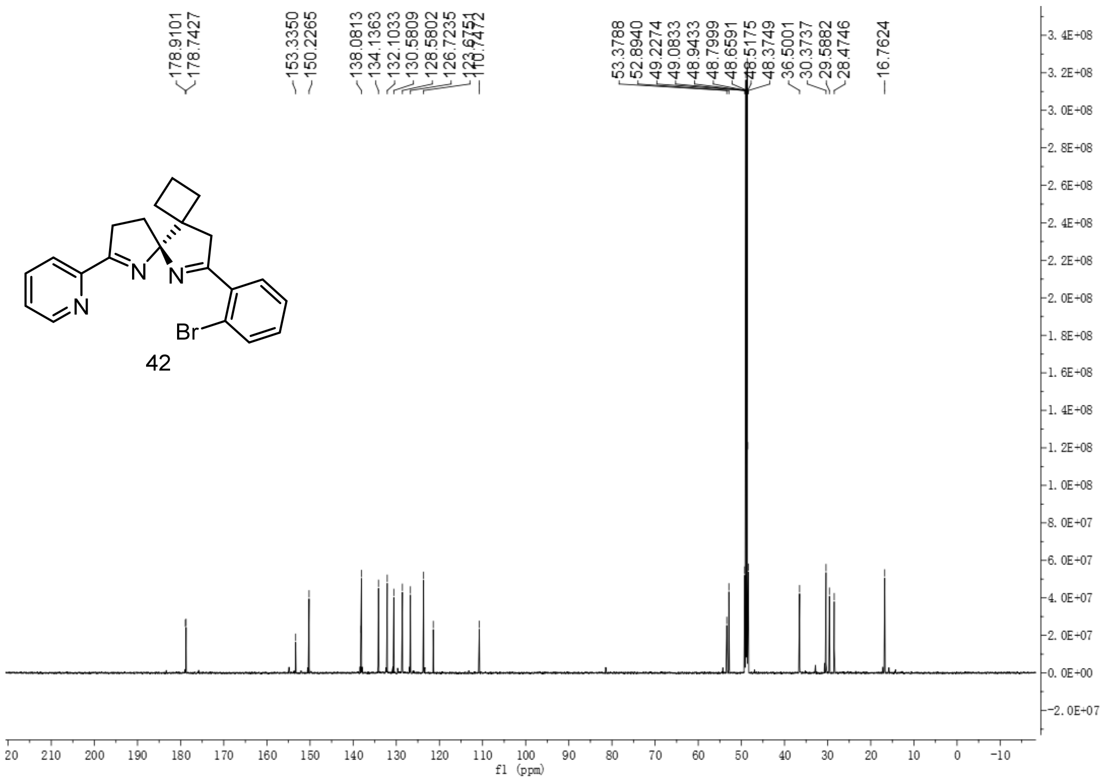

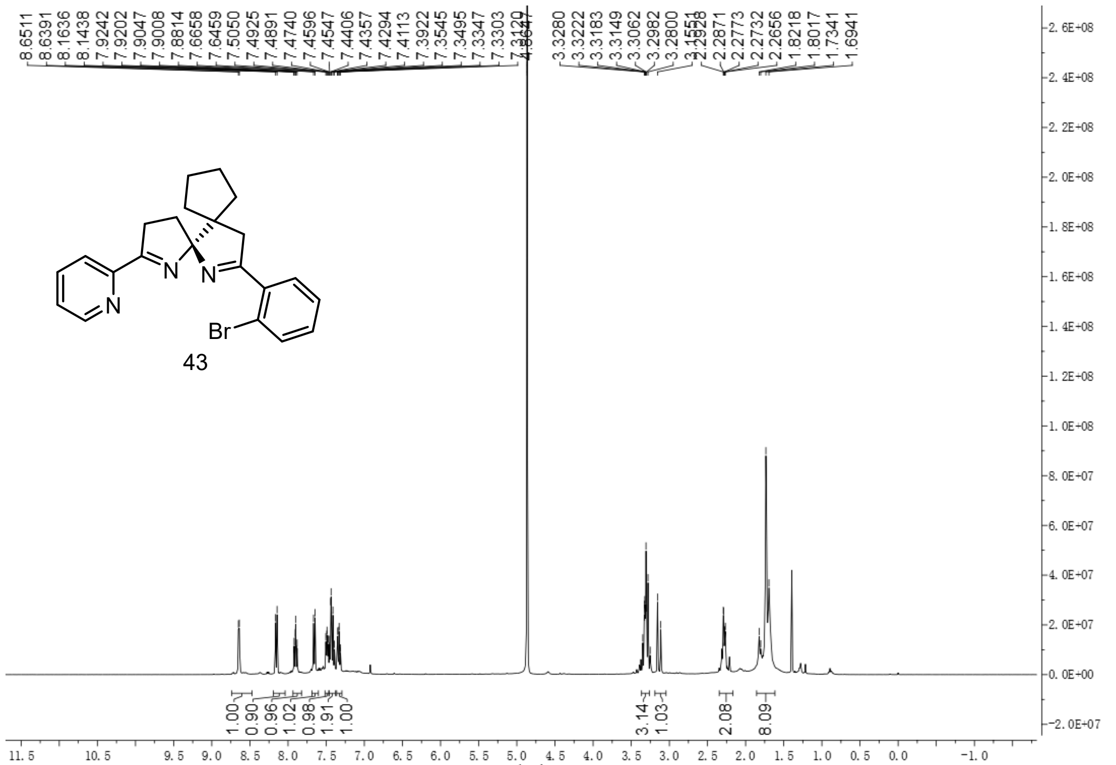

43

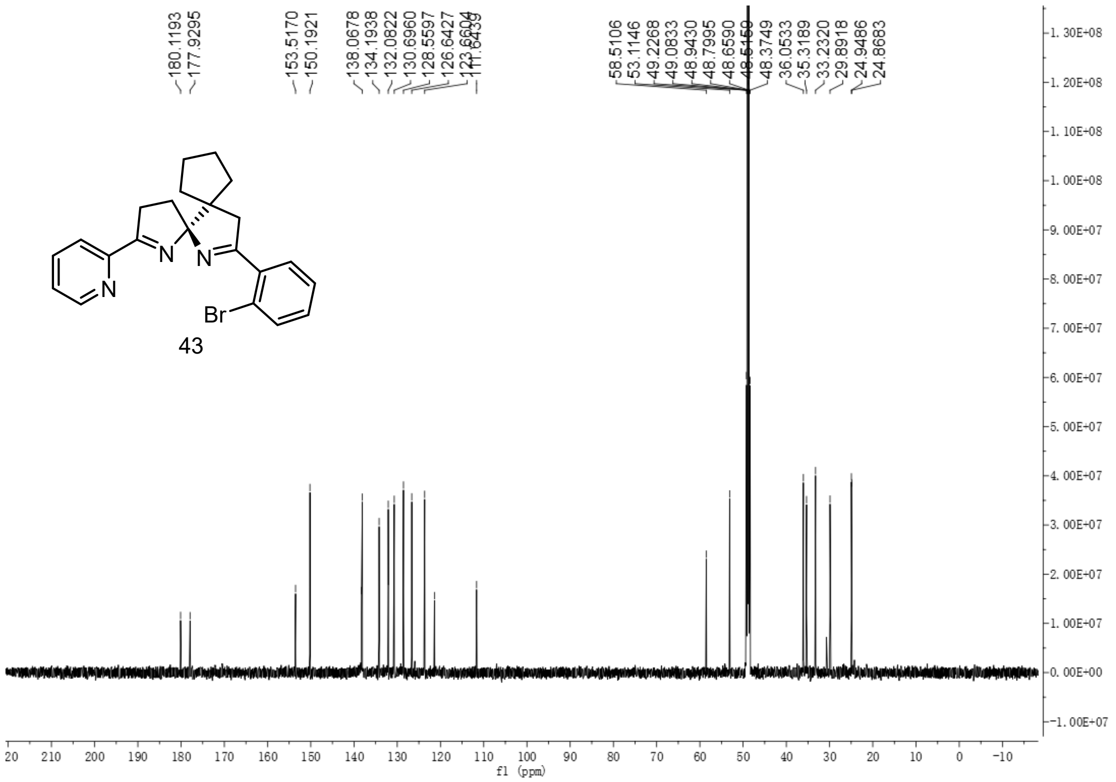

43

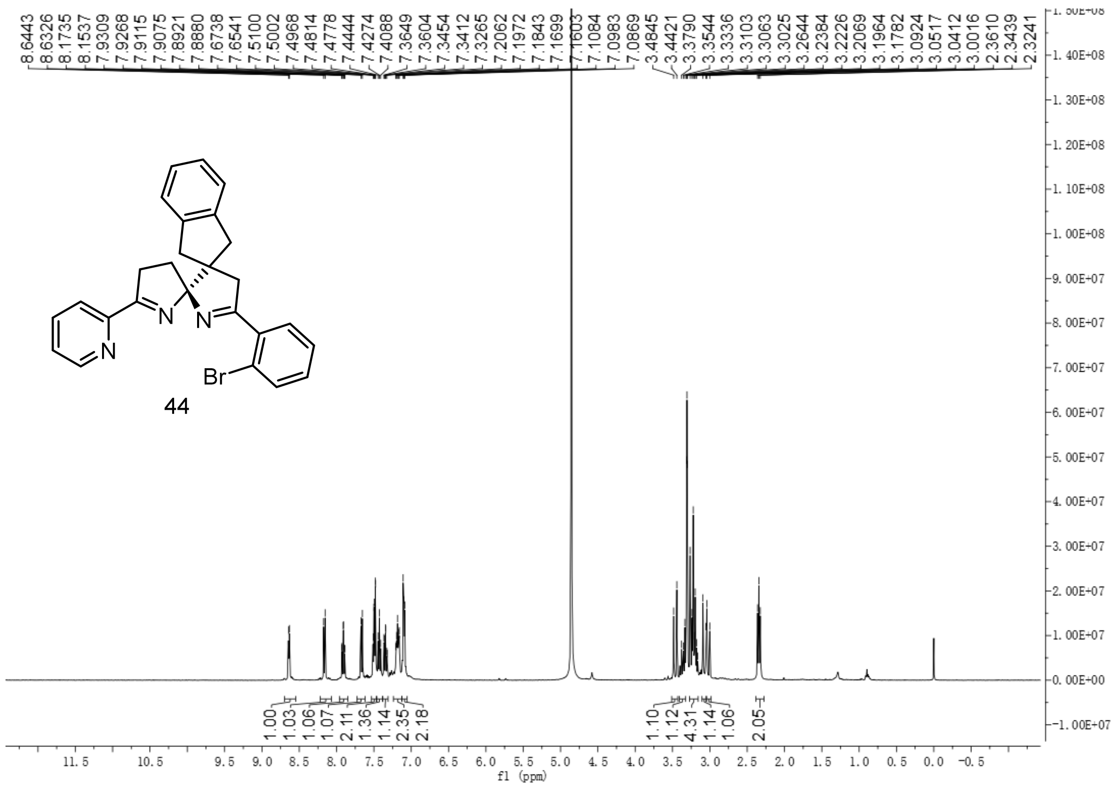

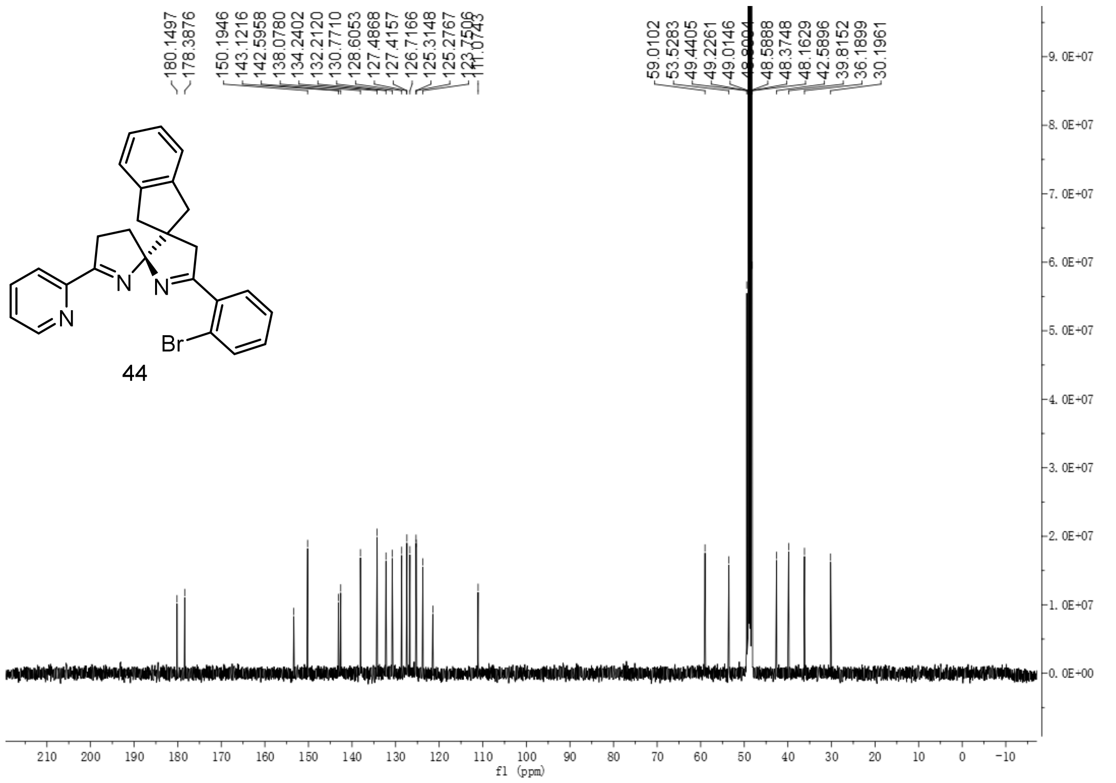

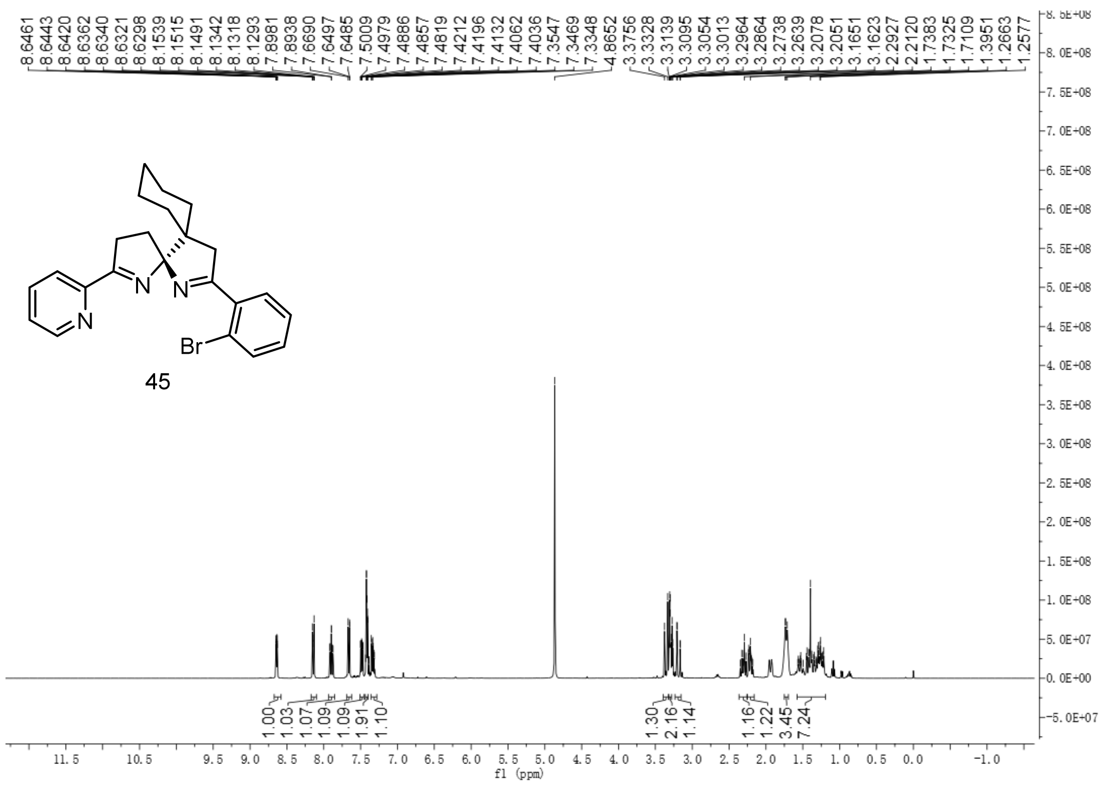
8.6461
8.6443
8.6420
8.6362
8.6340
8.6321
8.6298
8.1539
8.1515
8.1491
8.1342
8.1318
8.1293
7.8981
7.8938
7.6690
7.6497
7.6485
7.5009
7.4979
7.4886
7.4857
7.4819
7.4212
7.4196
7.4132
7.4062
7.4036
7.3547
7.3469
7.3348
4.8652
3.3756
3.3328
3.3139
3.3095
3.3054
3.3013
3.2964
3.2864
3.2738
3.2639
3.2078
3.2051
3.1651
3.1623
2.2927
2.2120
1.7383
1.7325
1.7109
1.3951
1.2663
1.2577
N
N
N
Br
45
1.00
1.03
1.07
1.09
1.09
1.91
1.10
1.30
2.16
1.14
1.16
1.22
3.45
7.24
f1 (ppm)

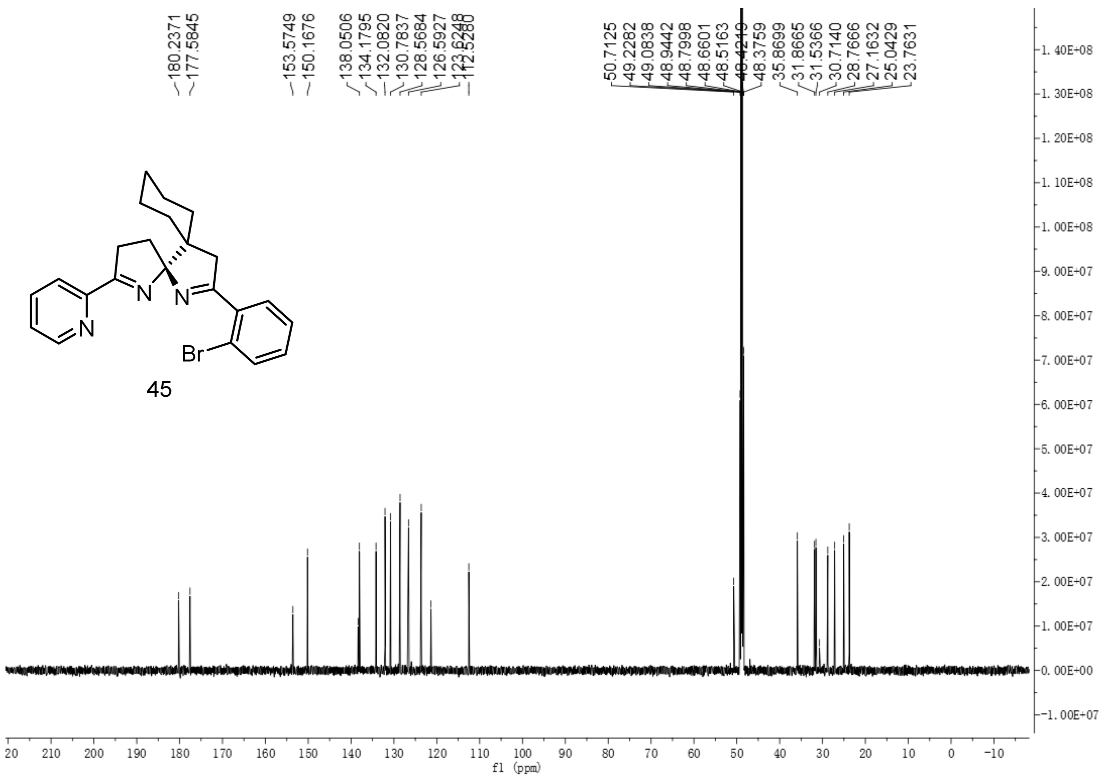
180.2371
177.5845
153.5749
150.1676
138.0506
134.1795
132.0820
130.7837
128.5684
126.5927
112.5280
50.7125
49.2282
49.0838
48.9442
48.7998
48.6601
48.5163
48.3759
35.8699
31.8665
31.5366
30.7140
28.7666
27.1632
25.0429
23.7631
N
N
N
Br
45
f1 (ppm)

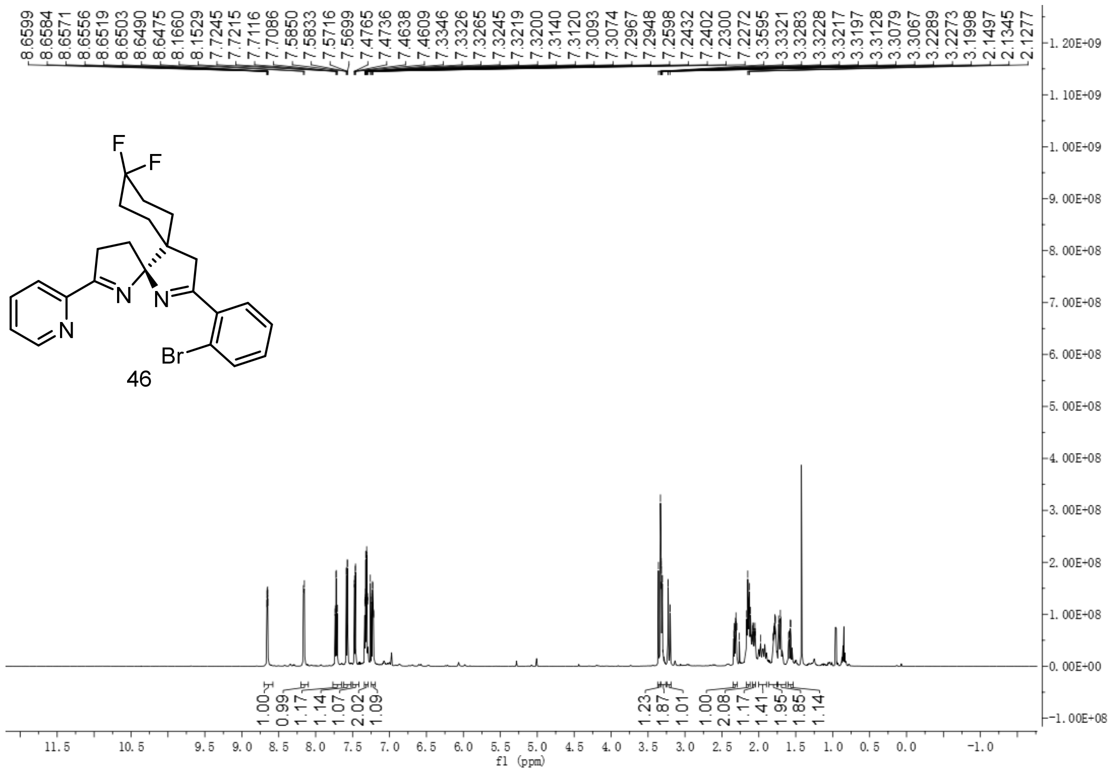

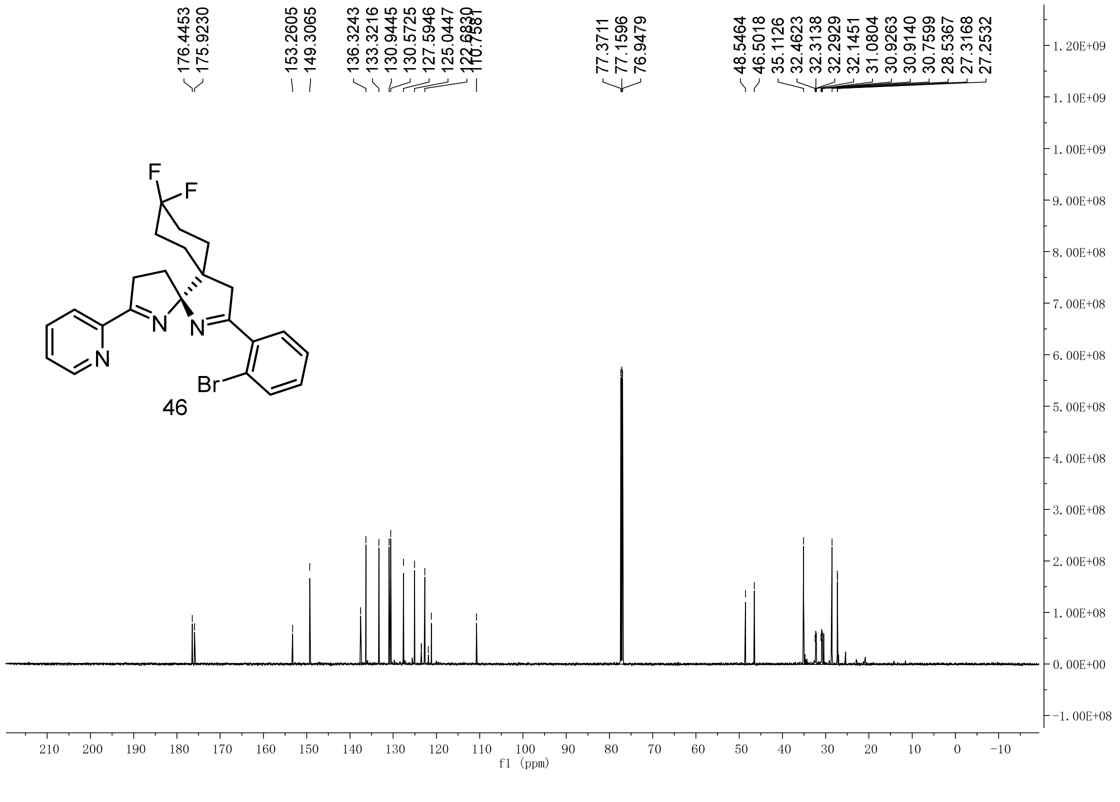

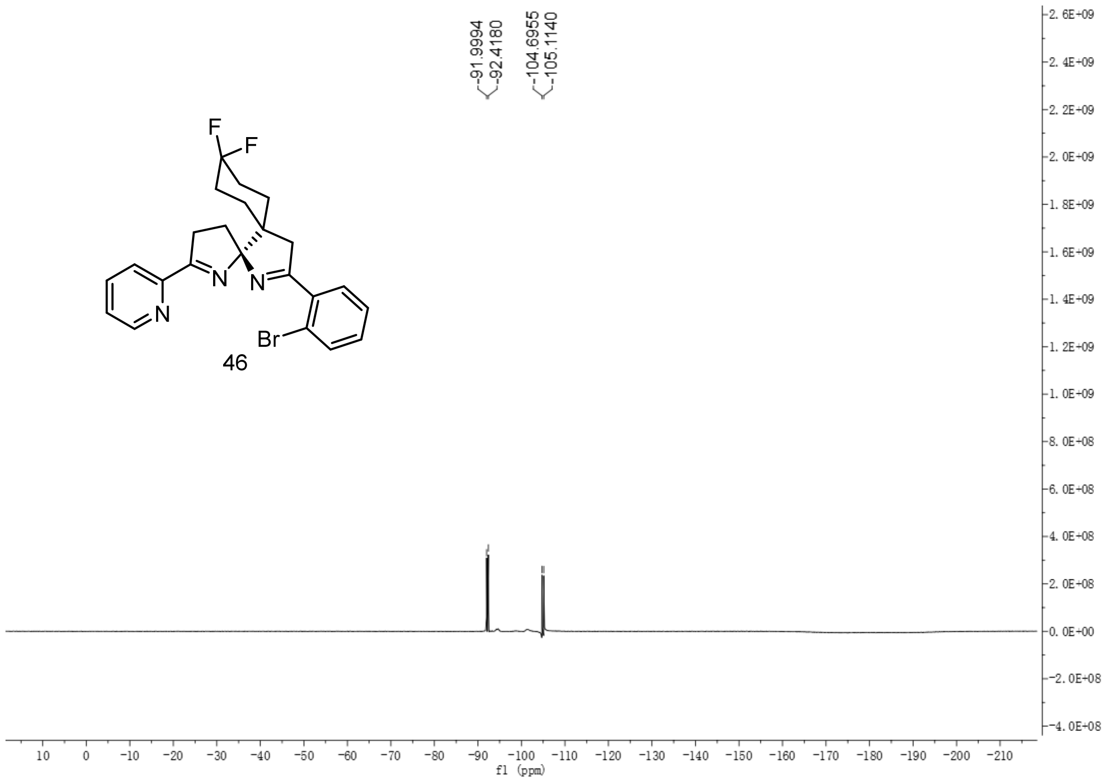
-91.9994
-92.4180
-104.6955
-105.1140
46
f1 (ppm)

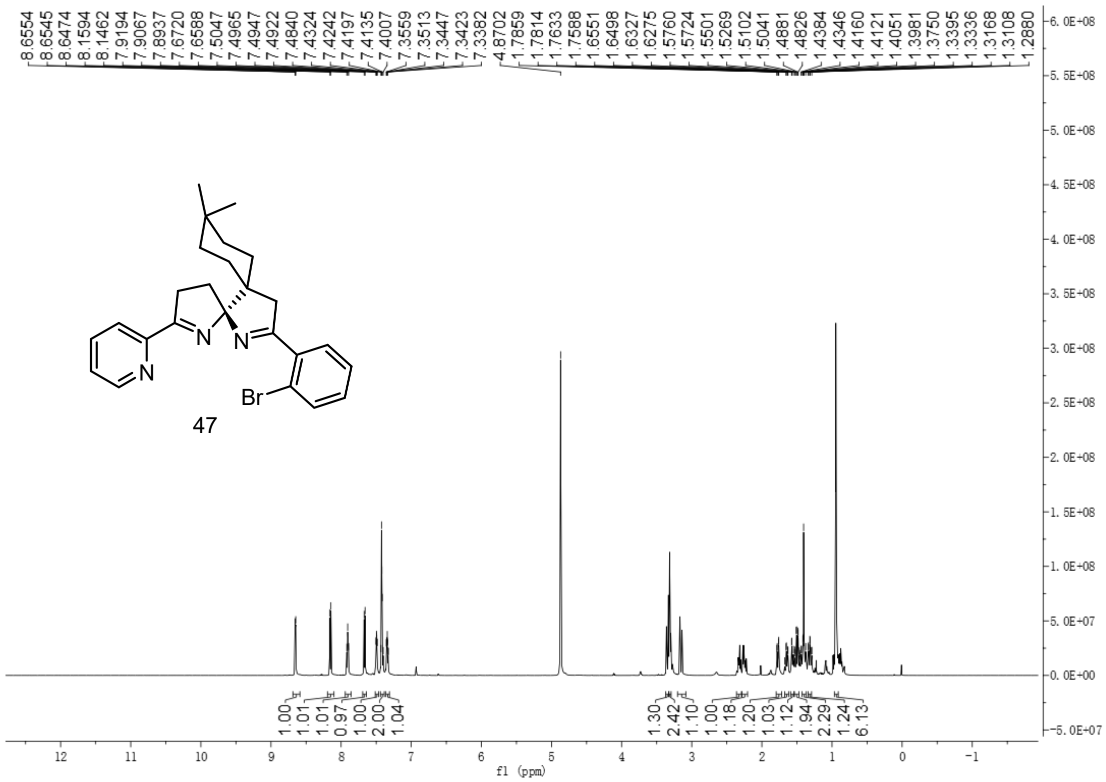
8.6554
8.6545
8.6474
8.1594
8.1462
7.9194
7.9067
7.8937
7.6720
7.6588
7.5047
7.4965
7.4947
7.4922
7.4840
7.4324
7.4242
7.4197
7.4135
7.4007
7.3559
7.3513
7.3447
7.3423
7.3382
4.8702
1.7859
1.7814
1.7633
1.7588
1.6551
1.6498
1.6327
1.6275
1.5760
1.5724
1.5501
1.5269
1.5102
1.5041
1.4881
1.4826
1.4384
1.4346
1.4160
1.4121
1.4051
1.3981
1.3750
1.3395
1.3336
1.3168
1.3108
1.2880
47
1.00
1.01
0.97
1.00
2.00
1.04
1.30
2.42
1.10
1.00
1.18
1.20
1.03
1.12
1.94
2.29
1.24
6.13
f1 (ppm)

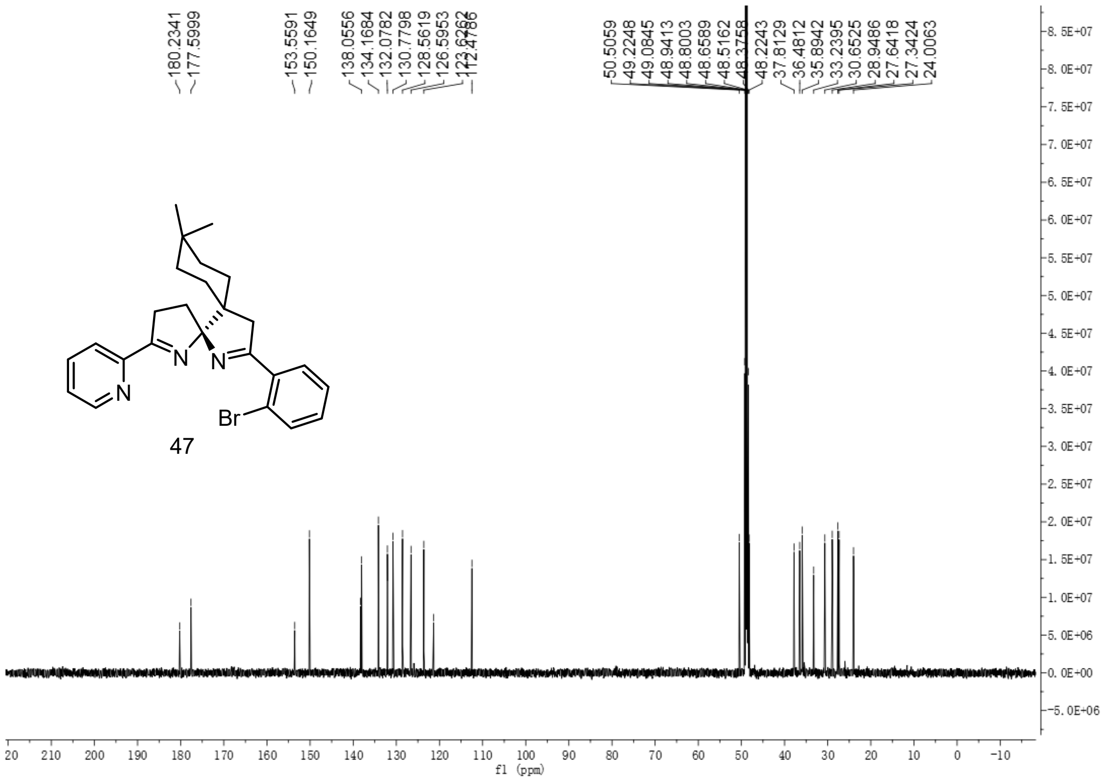

47

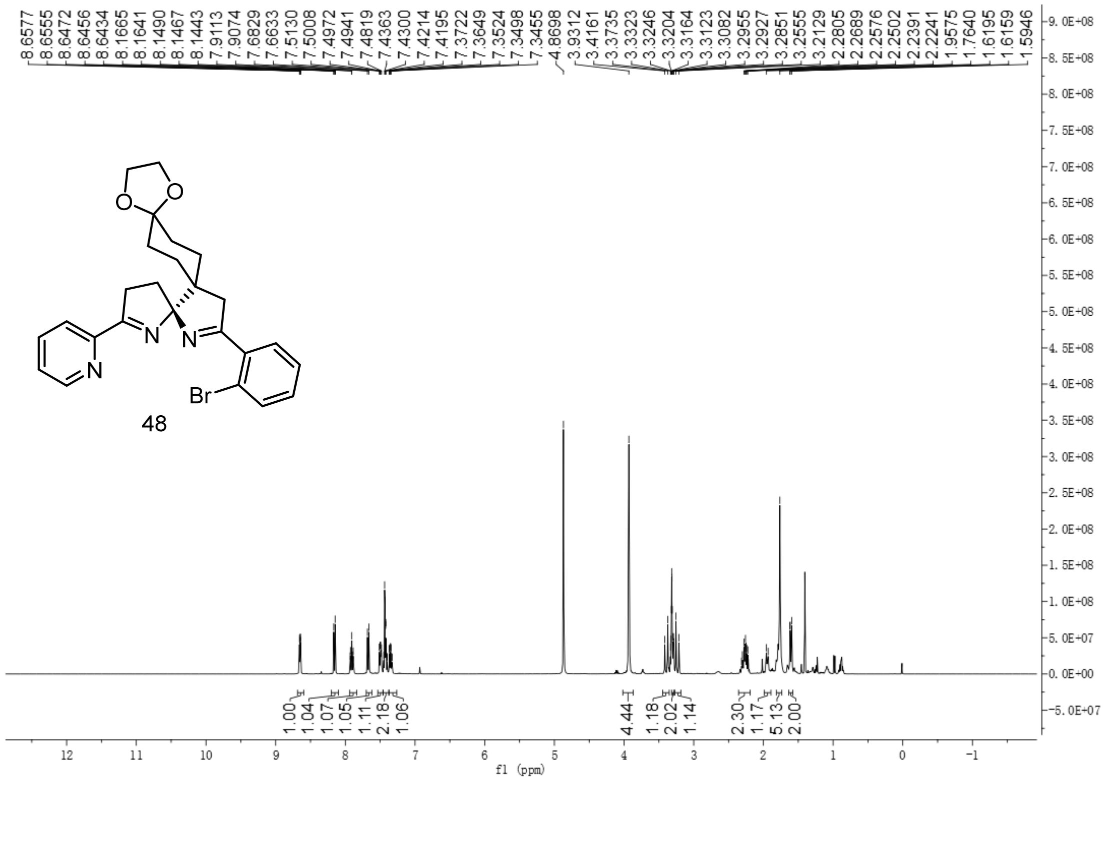

48

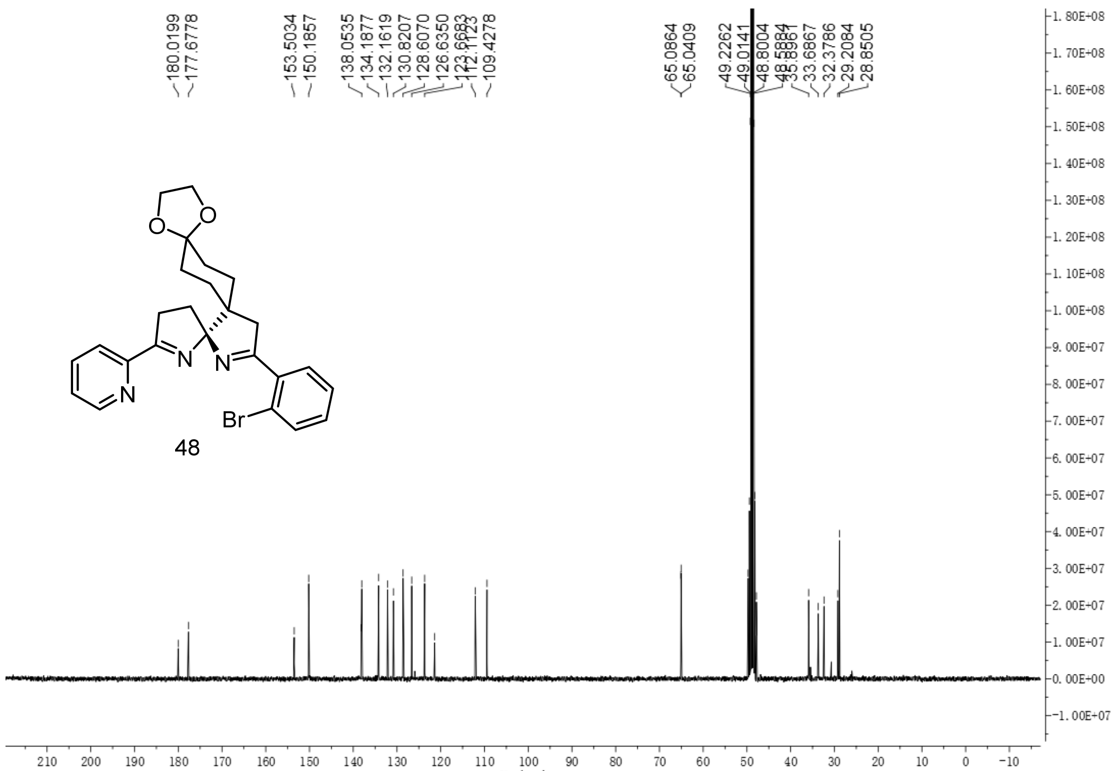

180.0199
177.6778
153.5034
150.1857
138.0535
134.1877
132.1619
130.8207
128.6070
126.6350
109.4278
65.0864
65.0409
49.2262
49.0141
48.8004
33.6867
32.3786
29.2084
28.8505
48
fl (ppm)

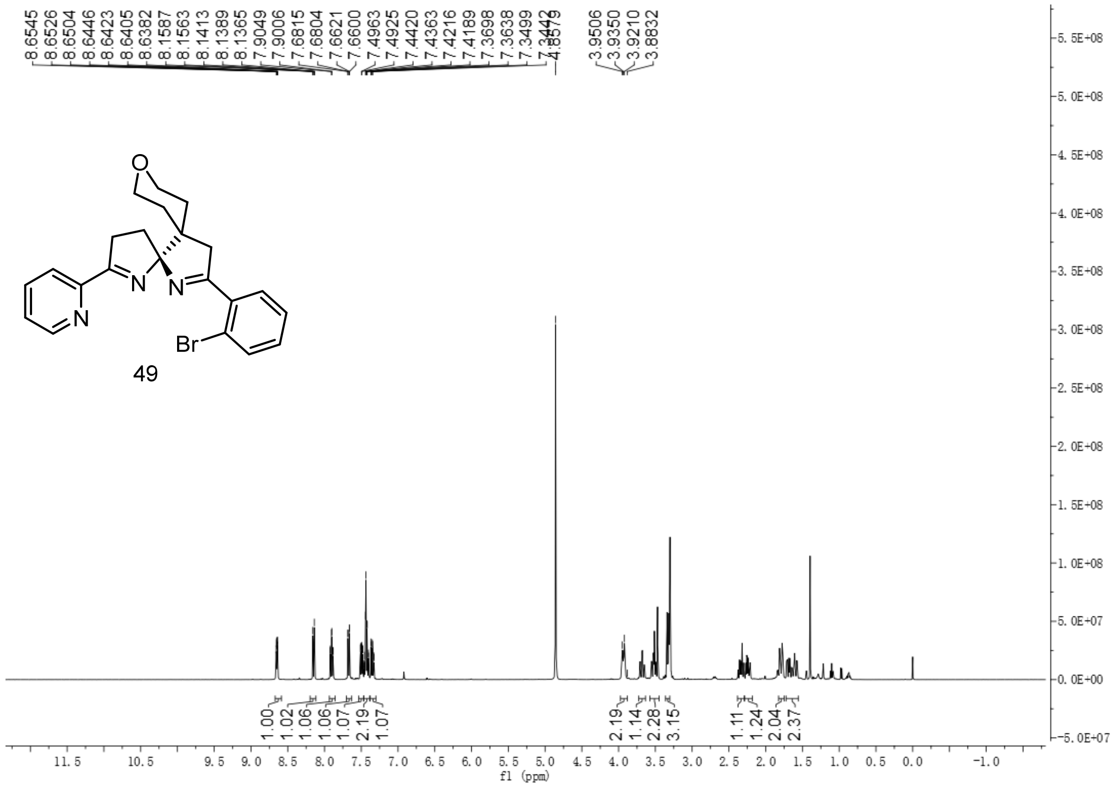

8.6545
8.6526
8.6504
8.6446
8.6423
8.6405
8.6382
8.1587
8.1563
8.1413
8.1389
8.1365
7.9049
7.9006
7.6815
7.6804
7.6621
7.6600
7.4963
7.4925
7.4420
7.4363
7.4216
7.4189
7.3698
7.3638
7.3499
3.9506
3.9350
3.9210
3.8832
49
1.00
1.02
1.06
1.06
1.07
2.19
1.07
2.19
1.14
2.28
3.15
1.11
1.24
2.04
2.37
fl (ppm)

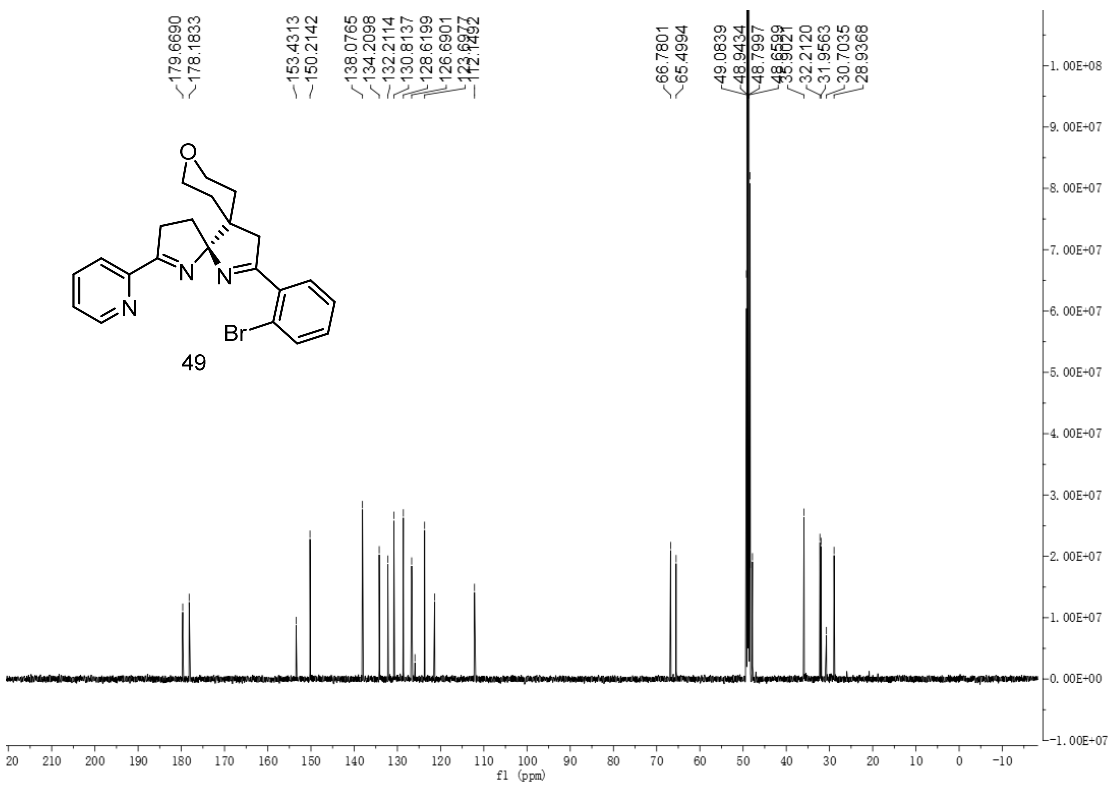
179.6690
178.1833
153.4313
150.2142
138.0765
134.2098
132.2114
130.8137
128.6199
126.6901
123.6977
112.1492
66.7801
65.4994
49.0839
48.9434
48.7997
48.6599
35.9021
32.2120
31.9563
30.7035
28.9368
O
N
N
N
Br
49
1.00E+08
9.00E+07
8.00E+07
7.00E+07
6.00E+07
5.00E+07
4.00E+07
3.00E+07
2.00E+07
1.00E+07
0.00E+00
−1.00E+07
20 210 200 190 180 170 160 150 140 130 120 110 100 90 80 70 60 50 40 30 20 10 0 −10
f1 (ppm)

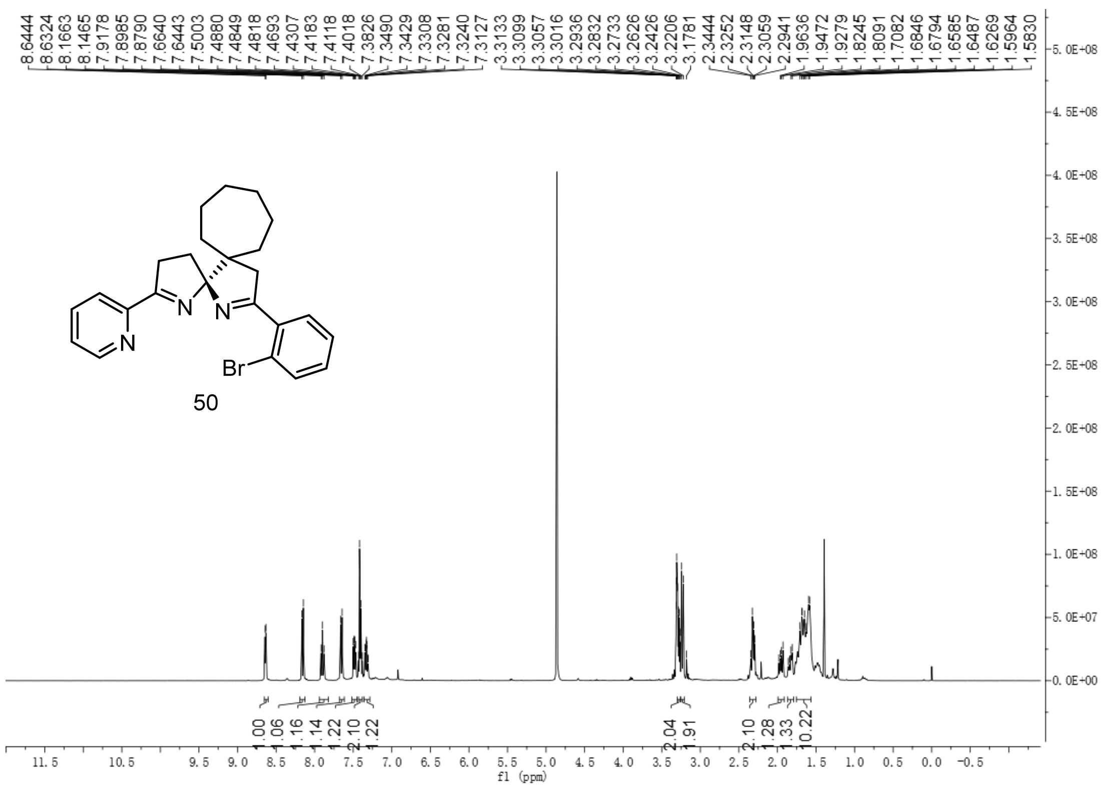
8.6444
8.6324
8.1665
8.1465
7.9178
7.8985
7.8790
7.6640
7.6443
7.5003
7.4880
7.4849
7.4818
7.4693
7.4307
7.4183
7.4118
7.4018
7.3826
7.3490
7.3429
7.3308
7.3281
7.3240
7.3127
3.3133
3.3099
3.3057
3.3016
3.2936
3.2832
3.2733
3.2626
3.2426
3.2206
3.1781
2.3444
2.3252
2.3148
2.3059
2.2941
1.9636
1.9472
1.9279
1.8245
1.8091
1.7082
1.6846
1.6794
1.6585
1.6487
1.6269
1.5964
1.5830
N
N
N
Br
50
5.0E+08
4.5E+08
4.0E+08
3.5E+08
3.0E+08
2.5E+08
2.0E+08
1.5E+08
1.0E+08
5.0E+07
0.0E+00
1.00
1.06
1.16
1.14
1.22
2.10
1.22
2.04
1.91
2.10
1.28
1.33
10.22
11.5 10.5 9.5 9.0 8.5 8.0 7.5 7.0 6.5 6.0 5.5 5.0 4.5 4.0 3.5 3.0 2.5 2.0 1.5 1.0 0.5 0.0 −0.5
f1 (ppm)

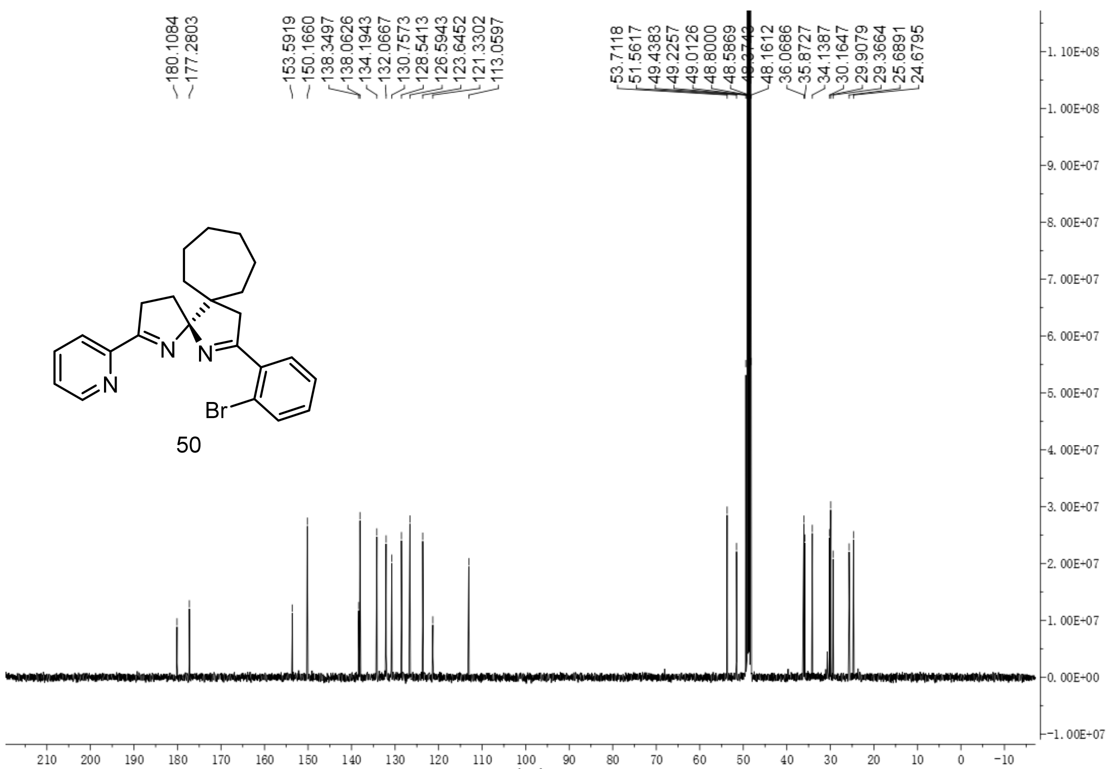
180.1084
177.2803
153.5919
150.1660
138.3497
138.0626
134.1943
132.0667
130.7573
128.5413
126.5943
123.6452
121.3302
113.0597
53.7118
51.5617
49.4383
49.2257
49.0126
48.8000
48.5869
48.1612
36.0686
35.8727
34.1387
30.1647
29.9079
29.3664
25.6891
24.6795
N
N
N
Br
fl (ppm)

50

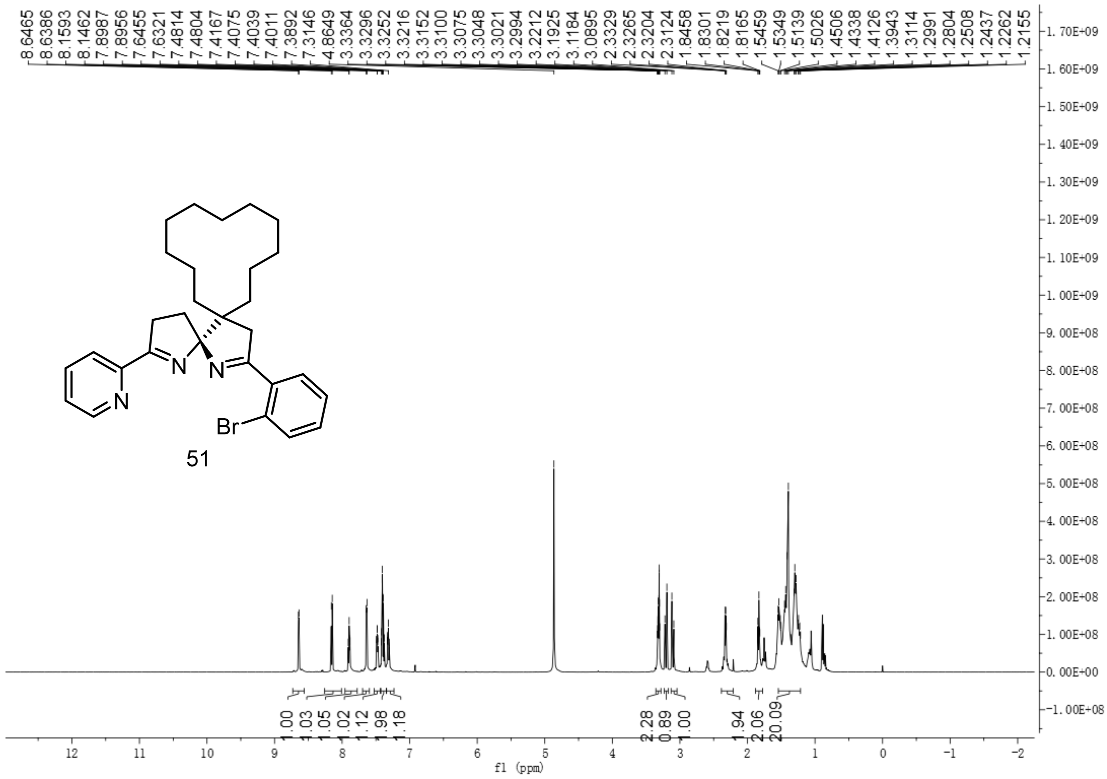
8.6465
8.6386
8.1593
8.1462
7.8987
7.8956
7.6455
7.6321
7.4814
7.4804
7.4167
7.4075
7.4039
7.4011
7.3892
7.3146
4.8649
3.3364
3.3296
3.3252
3.3216
3.3152
3.3100
3.3075
3.3048
3.3021
3.2994
3.2212
3.1925
3.1184
3.0895
2.3329
2.3265
2.3204
2.3124
1.8458
1.8301
1.8219
1.8165
1.5459
1.5349
1.5139
1.5026
1.4506
1.4338
1.4126
1.3943
1.3114
1.2991
1.2804
1.2508
1.2437
1.2262
1.2155
N
N
N
Br
1.00
1.03
1.05
1.02
1.12
1.98
1.18
2.28
0.89
1.00
1.94
2.06
20.09
fl (ppm)

51

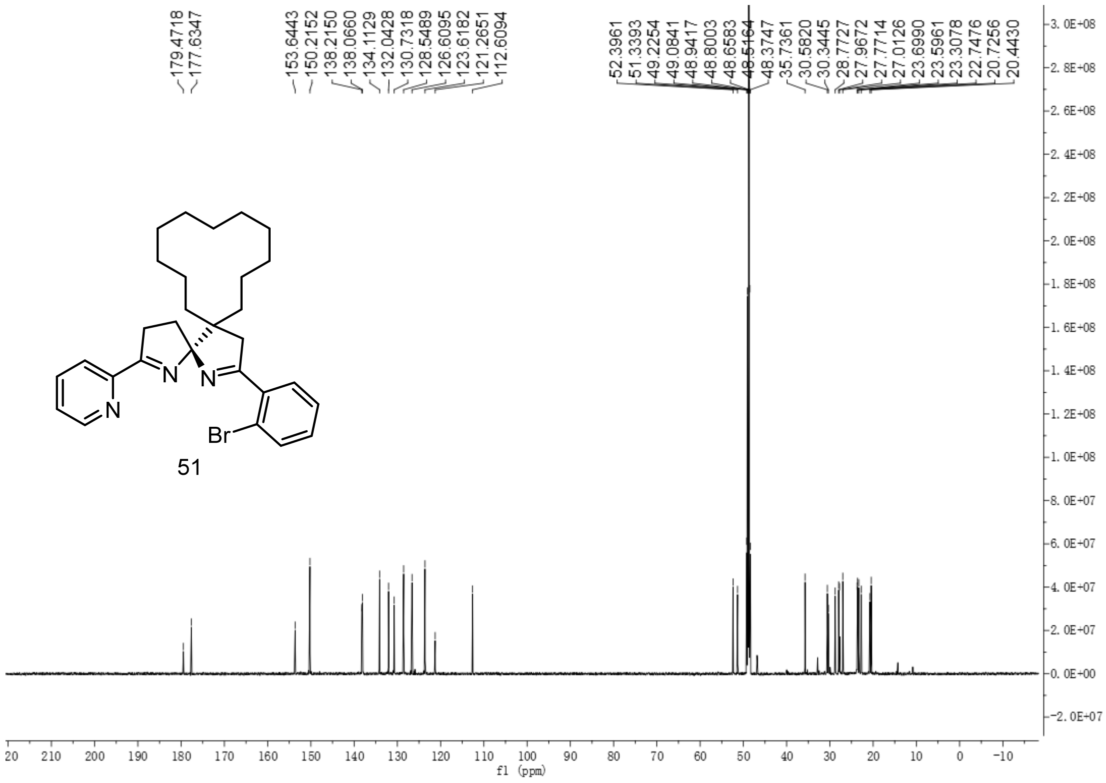

179.4718
177.6347
153.6443
150.2152
138.2150
138.0660
134.1129
132.0428
130.7318
128.5489
126.6095
123.6182
121.2651
112.6094
52.3961
51.3393
49.2254
49.0841
48.9417
48.8003
48.6583
48.5164
48.3747
35.7361
30.5820
30.3445
28.7727
27.9672
27.7714
27.0126
23.6990
23.5961
23.3078
22.7476
20.7256
20.4430
N
N
N
Br
51
f1 (ppm)

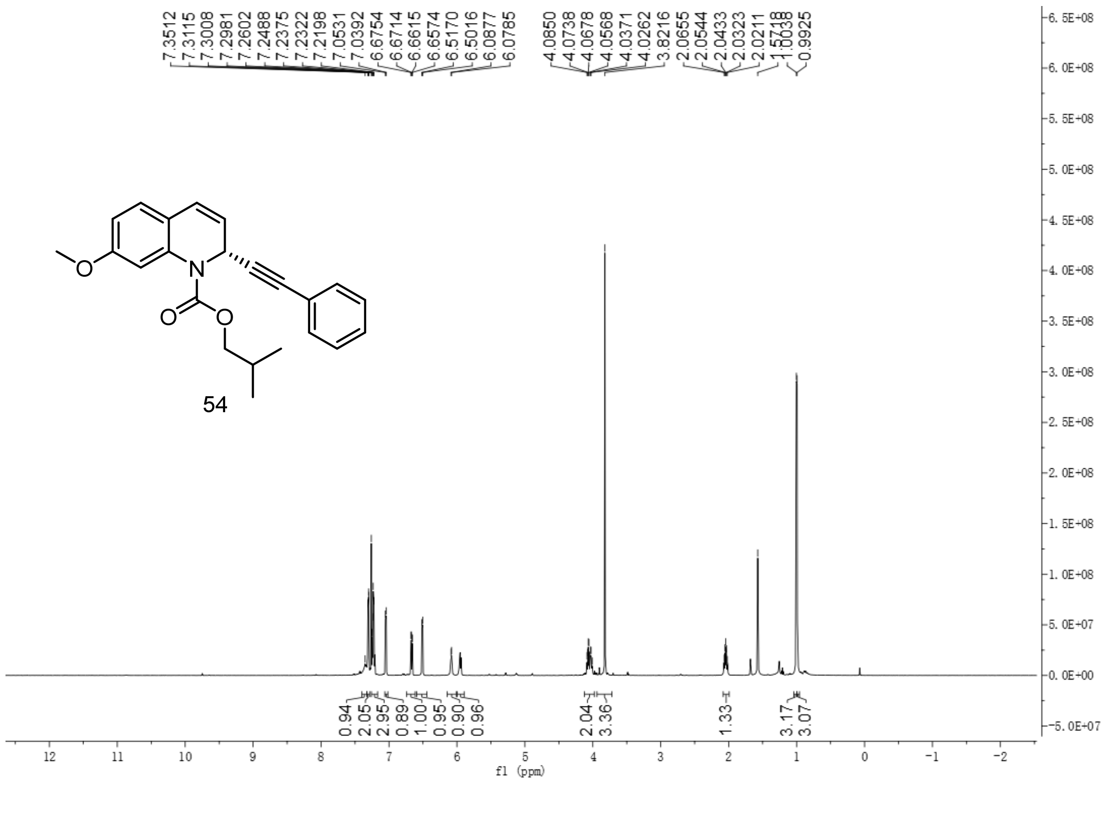

7.3512
7.3115
7.3008
7.2981
7.2602
7.2488
7.2375
7.2322
7.2198
7.0531
7.0392
6.6754
6.6714
6.6615
6.6574
6.5170
6.5016
6.0877
6.0785
4.0850
4.0738
4.0678
4.0568
4.0371
4.0262
3.8216
2.0655
2.0544
2.0433
2.0323
2.0211
1.5718
1.0038
0.9925
O
N
O
O
54
0.94
2.05
2.95
0.89
1.00
0.95
0.90
0.96
2.04
3.36
1.33
3.17
3.07
f1 (ppm)

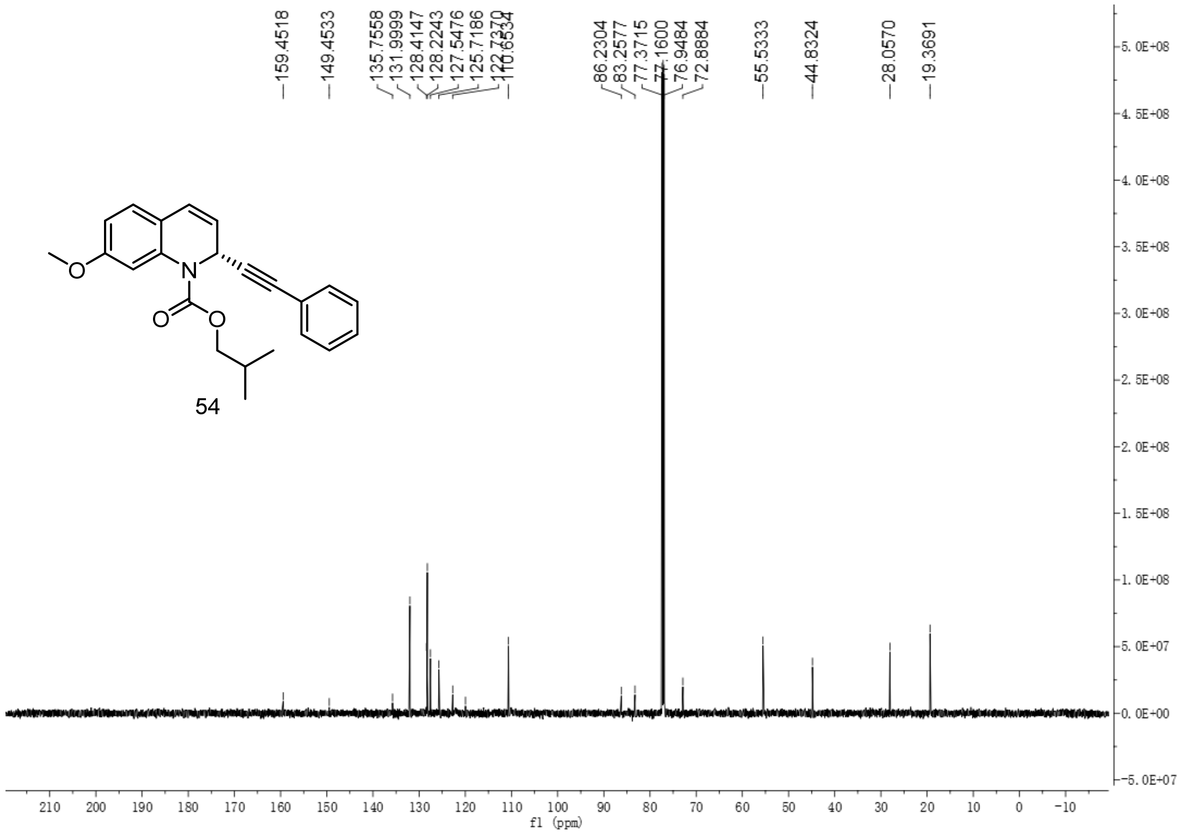

159.4518
149.4533
135.7558
131.9999
128.4147
128.2243
127.5476
125.7186
122.7370
110.6534
86.2304
83.2577
77.3715
77.1600
76.9484
72.8884
55.5333
44.8324
28.0570
19.3691
N
O
O
O
54
f1 (ppm)

9. References

(1) P.Rabe, L.Barra, J. Rinkel, R. Riclea, C. Citron, T. Klapschinski, A. Janusko, J. Dickschat, *Angew. Chem. Int. Ed.* **2015**, *54*, 13448–13451.

(2) C. Huang, Z. Li, J. Song, H. Xu, *Angew. Chem. Int. Ed.* **2021**, *60*, 11237–11241.

(3) C. Hsu, H. Lin, X. Hou, R. Tapales, C. Shih, S. Miñoza, Y. Tsai, Z. Tsai, C. Chan, H. Liao, *Am. Chem. Soc*. **2023**, *145*, 19049−19059.

(4) N. Gesmundo, D. Nicewicz, *Beilstein J. Org. Chem*. **2014**, *10*, 1272–1281.

(5) J. Davies, L. Angelini, M. Alkhalifah, L. Sanz, N. Sheikh, D. Leonori, *Synthesis* **2018**, *50*, 821-830.

(6) H. Xu, J. He, J. Shi, L. Tan, D. Qiu, X. Luo, Y. Li, *J. Am. Chem. Soc*. **2018**, *140*, 3555−3559.

(7) X. Shen, C. Huang, X. Yuan, S. Yu, *Angew. Chem. Int. Ed.* **2021**, *60*, 9672–9679.

(8) Y. Liao, Y. Ran, G. Liu, P. Liu, X. Liu, *Org. Chem. Front.* **2020**, *7*, 3638–3647.

(9) S. Li, H. Du, P. Davies, W. Shu, Doi: 10.26434/chemrxiv-2023-z1fq5.

(10) M. Andresini, L. Degannaroand, R. Luisi, Beilstein *J. Org. Chem.* **2021**, *17*, 203–209.

(11) L. Liu, Q. Zhang, C. Wang, *Org. Lett.* **2022**, *24*, 5913−5917.

(12) M. Pappoppula, F. Cardoso, B. Garrett, A. Aponick, *Angew. Chem. Int. Ed*. **2015**, *54*, 15202–15206.
